# Functional analysis reveals driver cooperativity and novel mechanisms in endometrial carcinogenesis

**DOI:** 10.15252/emmm.202217094

**Published:** 2023-08-17

**Authors:** Matthew Brown, Alicia Leon, Katarzyna Kedzierska, Charlotte Moore, Hayley L Belnoue‐Davis, Susanne Flach, John P Lydon, Francesco J DeMayo, Annabelle Lewis, Tjalling Bosse, Ian Tomlinson, David N Church

**Affiliations:** ^1^ Cancer Genomics and Immunology Group, Wellcome Centre for Human Genetics University of Oxford Oxford UK; ^2^ Oxford NIHR Comprehensive Biomedical Research Centre, Oxford University Hospitals NHS Foundation Trust Oxford UK; ^3^ Department of Pathology Leiden University Medical Center Leiden The Netherlands; ^4^ Gastrointestinal Stem Cell Biology Laboratory, Wellcome Centre for Human Genetics University of Oxford Oxford UK; ^5^ Department of Otorhinolaryngology, Head and Neck Surgery LMU Klinikum Munich Germany; ^6^ German Cancer Consortium (DKTK), Partner Site Munich Germany; ^7^ Department of Molecular and Cellular Biology Baylor College of Medicine Houston TX USA; ^8^ Reproductive and Developmental Biology Laboratory National Institute of Environmental Health Sciences Research Triangle Park NC USA; ^9^ Department of Life Sciences, College of Health, Medicine and Life Sciences Brunel University London Uxbridge UK; ^10^ Institute of Genetics and Cancer The University of Edinburgh Edinburgh UK; ^11^ Oxford Cancer Centre, Churchill Hospital Oxford University Hospitals Foundation NHS Trust Oxford UK

**Keywords:** Driver genes, Endometrial cancer, Fbxw7, Functional models, GEMM, Cancer, Urogenital System

## Abstract

High‐risk endometrial cancer has poor prognosis and is increasing in incidence. However, understanding of the molecular mechanisms which drive this disease is limited. We used genetically engineered mouse models (GEMM) to determine the functional consequences of missense and loss of function mutations in *Fbxw7*, *Pten* and *Tp53*, which collectively occur in nearly 90% of high‐risk endometrial cancers. We show that *Trp53* deletion and missense mutation cause different phenotypes, with the latter a substantially stronger driver of endometrial carcinogenesis. We also show that *Fbxw7* missense mutation does not cause endometrial neoplasia on its own, but potently accelerates carcinogenesis caused by *Pten* loss or *Trp53* missense mutation. By transcriptomic analysis, we identify LEF1 signalling as upregulated in *Fbxw7/FBXW7*‐mutant mouse and human endometrial cancers, and in human isogenic cell lines carrying *FBXW7* mutation, and validate LEF1 and the additional Wnt pathway effector TCF7L2 as novel FBXW7 substrates. Our study provides new insights into the biology of high‐risk endometrial cancer and suggests that targeting LEF1 may be worthy of investigation in this treatment‐resistant cancer subgroup.

## Graphical abstract

The consequences of common endometrial driver mutations were studied in mice and found to exert different functional effects alone and in combination.

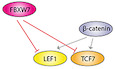

**The paper explained**

**Problem**

Endometrial cancer (EC) is the most common gynaecological malignancy in the developed world. Mechanistic insights into high‐risk EC may help identify novel therapeutic targets against this poor prognosis subgroup.

**Results**

Using genetically modified mouse models (GEMM) we show that driver mutations common in high‐risk EC exert different effects alone and in combination. *Trp53* missense mutation reliably induces EC, while *Trp53* loss does not. Cancer‐associated missense mutation of the ubiquitin ligase *Fbxw7* does not cause EC in isolation, but potently accelerates EC caused by *Pten* loss or *Trp53* missense mutation. *Fbxw7*‐mutant mouse EC display enrichment of a gene signature of the Wnt pathway effector Lef1, and the same signature is evident in human EC and human isogenic cell lines with *FBXW7* mutation. Using immunoprecipitation, we confirm LEF1 and the related Wnt effector TCF7L2 as novel FBXW7 substrates, thus identifying a novel mechanism of FBXW7 mediated tumour suppression.

**Impact**

This study sheds new lights on the mechanistic basis of EC and suggests that targeting of the Wnt pathway may be worthy of investigation in EC carrying *FBXW7* mutations.

## Introduction

Endometrial cancer (EC) is the most common gynaecological malignancy in the developed world, with more than 150,000 cases each year in Europe and the United States (Sung *et al*, 2021). While most are low‐grade endometrioid tumours of early stage with favourable prognosis, the 10–20% of ECs classified as high‐risk have considerably worse outcomes (Creasman *et al*, 2006; León‐Castillo *et al*, 2020; Crosbie *et al*, 2022). This group includes high‐grade (grade 3) endometrioid tumours, cases of non‐endometrioid histologies including serous, clear‐cell and undifferentiated histology, and de‐differentiated carcinosarcomas; metaplastic tumours derived from epithelial progenitors (Toboni *et al*, 2021).

Tumour sequencing studies, including those performed by The Cancer Genome Atlas (TCGA) have characterised the somatic mutational landscape of EC and its variation between subgroups (Gallo *et al*, 2012; Cancer Genome Atlas Research Network *et al*, 2013; Zhao *et al*, 2013). These have confirmed the high mutation frequency of known drivers such as *PTEN* and *TP53* in endometrioid and non‐endometrioid tumours respectively, and identified over 30 other significantly recurrently mutated genes (Gallo *et al*, 2012; Cancer Genome Atlas Research Network *et al*, 2013; Zhao *et al*, 2013; Martincorena *et al*, 2017; Bailey *et al*, 2018; Martínez‐Jiménez *et al*, 2020). Among the most commonly mutated of these, particularly in non‐endometrioid tumours, is *FBXW7*—the substrate recognition component of an SCF complex responsible for the ubiquitylation and degradation of multiple oncogenic substrates, including cyclin E, mTOR and Jun (Welcker & Clurman, 2008). Mechanistically, *FBXW7* recognises substrates through interaction of arginine residues in its WD40 domain beta propellors with a conserved Cdc4 phosphodegron (CPD) sequence (Orlicky *et al*, 2003; Hao *et al*, 2007). Cancer‐associated *FBXW7* mutations are often heterozygous substitutions of these residues, rather than the truncating mutations predominant among other tumour suppressors (Davis & Tomlinson, 2012). This finding has been attributed to the fact that FBXW7 exists as a homodimer, meaning that these missense mutations exert dominant negative effects (Davis *et al*, 2014a). However, whether this is the case in EC is unknown.

The functional consequences of manipulation of several genes identified as EC drivers has been investigated using genetically engineered mouse models (GEMM). Conditional uterine deletion of *Pten* reliably caused endometrial cancer in mice, and this phenotype was accelerated by concomitant *Trp53* deletion (Daikoku *et al*, 2008). While this study did not report the effects of *Trp53* deletion alone, another study which used an alternative Cre recombinase showed this caused EC with long latency (typically > 65 weeks; Wild *et al*, 2012). The effects of deletion of *Fbxw7* in the mouse endometrium were recently reported (Cuevas *et al*, 2019). While deletion of *Fbxw7* alone did not induce neoplasia by 52 weeks age, co‐deletion with *Pten* caused aggressive tumours resembling carcinosarcomas in all cases by 40–67 weeks age (Cuevas *et al*, 2019). These tumours showed frequent Trp53 mutation or p53‐mutant immunostaining, suggesting functional interplay between the Pten‐PI3K, Trp53 and Fbxw7 pathways (Cuevas *et al*, 2019).

While these preclinical studies have substantially advanced our understanding of EC biology, several gaps remain. The functional consequences of *Fbxw7* and *Trp53* missense mutations typical in human cancer are currently uncharacterised. The effects of combined defects in *Fbxw7* and *Trp53* common in high‐risk human EC await definition. And the substrates dysregulated as a result of endometrial *Fbxw7* mutation *in vivo* are unknown. Given that *FBXW7* missense (but not truncating) mutations are common in normal endometrial glands (Moore *et al*, 2020) and that its substrates appear to vary by tissue (Davis *et al*, 2014a), addressing these is critical if FBXW7 is to be a target for endometrial cancer prevention and therapy.

In this study, we sought to do this by the generation and phenotyping of GEMM with endometrial expression of a cancer hotspot *Fbxw7* mutation alone, in combination with *Pten* deletion, and in combination with either deletion or mutation of *Trp53*.

## Results

### *FBXW7* hotspot mutations, *PTEN* and *TP53* mutation are common in high‐risk endometrial cancer

**Figure 1:**
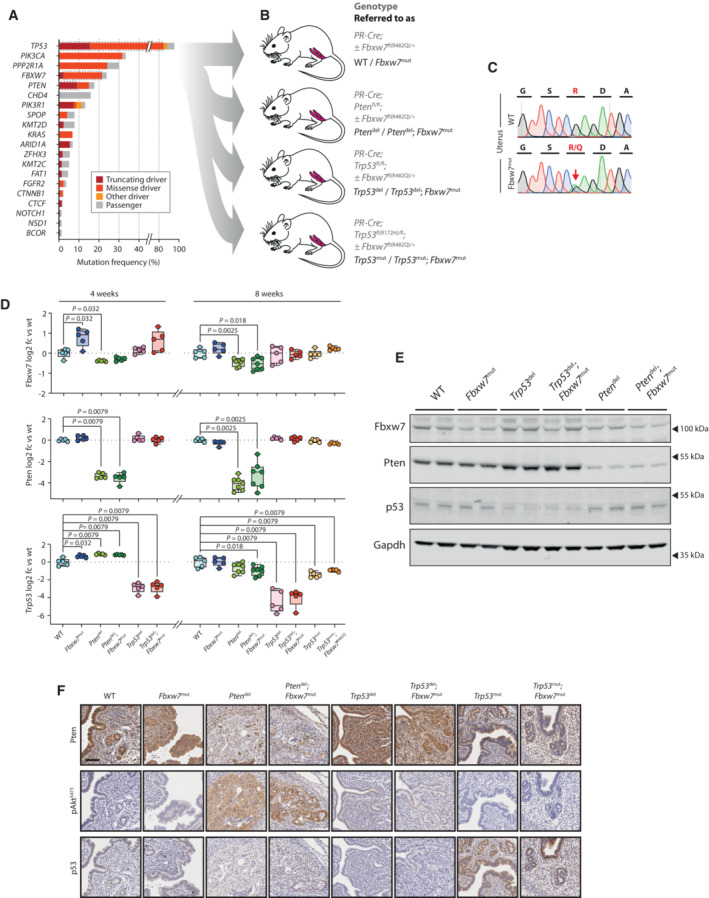
Functional analysis of high‐risk endometrial cancer drivers in combination reveals interactions between Fbxw7, Pten and p53 Frequency and type of somatic mutations in top 20 driver genes in high‐risk endometrial cancer (EC) from TCGA UCEC and UCS cohorts. Details of mutation classification are provided in Materials and Methods.Genetically engineered mouse models (GEMM) used in this study comprising conditional knock‐in of heterozygous *Fbxw7* ^R482Q^ missense mutation (corresponding to human hotspot *FBXW7* ^R479Q^), homozygous *Pten* loss, homozygous *Trp53* loss and heterozygous *Trp53* ^R172H^ missense mutation (corresponding to human hotspot *TP53* ^R175H^) alone and in combination. Full genotypes are provided in grey text, with abbreviations used in this study in black text. Further details are provided in Table 1.Sanger sequencing of cDNA from indicated tissues showing expression of *Fbxw7* ^R482Q^ knock‐in allele in mouse uterus.Real‐time reverse transcription quantitative PCR (RT–qPCR) showing Fbxw7, Pten and Trp53 expression in mouse uteri at 4 and 8 weeks postnatal age (NB *Trp53* ^R172H^ uteri were not analysed at the 4 week timepoint). Box extends from 25^th^ to 75^th^ percentiles, with line at median, whiskers indicate minimum and maximum values, and dots individual values (*n* = 5 samples per group with exception of *Pten* ^del^ and *Pten* ^del^, *Fbxw7* ^mut^ genotypes at 8 weeks which contain *n* = 7 samples each). Statistical comparison between WT controls and other genotypes was performed by unadjusted, unpaired Mann–Whitney test; *P < 0.05, **P < 0.01.Western blot of mouse uteri at 8 weeks postnatal age (NB *Trp53* ^R172H^ uteri were not analysed by Western blotting). Values indicate densitometric units (mean ± SD) normalised to control group of WT samples in case of Fbxw7 protein and both WT and *Fbxw7* ^mut^ samples in case of Pten and p53 proteins. Image is representative of duplicate technical replicates. Statistical comparison between controls and other groups was performed by unadjusted, unpaired Mann–Whitney test; and are shown next to densitometry results where statistically significant.Immunohistochemical (IHC) analysis of mouse uteri at 8 weeks postnatal age. Images are representative of minimum of 3 biological replicates for each genotype and protein. Scale bar indicates 100 μm. Source data are available online for this figure.

We first examined *FBXW7*, *TP53* and *PTEN* mutation frequency and type relative to other IntOgen EC drivers (Martínez‐Jiménez *et al*, 2020) in the TCGA uterine corpus endometrial cancer (UCEC; Cancer Genome Atlas Research Network, 2013) and carcinosarcoma (UCS; Cherniack *et al*, 2017) cohorts, focusing on the copy number high UCEC subgroup and carcinosarcomas—hereafter referred to as high‐risk endometrial cancer—in view of their exaggerated morbidity and mortality (Crosbie *et al*, 2022) (note that this definition excludes clear cell and undifferentiated tumours, which have poor prognosis but were excluded from the TCGA analyses). As expected, *TP53* was the most frequently mutated driver (84.0% cases), with *PIK3CA* (32.0%), *PPP2R1A* (24.2%) and *FBXW7* (21.5%), the next most commonly mutated genes. Interestingly, despite its association with low‐grade disease, *PTEN* was the 5^th^ most commonly mutated driver (15.1% cases) in this high‐risk subset (Fig 1A). Known and predicted oncogenic mutations in *TP53* and *PTEN* included recurrent hotspot missense mutations and protein‐truncating variants (PTVs), with preponderance of hotspots in *TP53* (146 of 184; 79.3%) and PTVs in *PTEN* (20 of 33; 60.6%). In contrast, nearly 90% (42 of 47) of oncogenic *FBXW7* mutations were hotspot missense alterations – typically arginine residues in the WD40 domain—suggesting strong selection for these over PTVs in high‐risk EC (Fig 1A). This was supported by cross‐cancer comparison, which revealed that the proportion of *FBXW7* missense driver mutations to PTVs in high‐risk EC was significantly greater than all other cancer types in which *FBXW7* is mutated in more than 5% cases (*P* < 1.0e−04 to 7.2e−03, Fisher exact test) (Appendix Fig S1). Oncogenic mutations in *FBXW7* in this cohort of high‐risk endometrial cancers tended to mutual exclusivity with those in *TP53* (OR = 0.53, unadjusted *P* = 0.12) and particularly *PTEN* (OR = 0.32, unadjusted *P* = 0.067).

### Variable effects of high‐risk endometrial cancer drivers alone and in combination in GEMM

We sought to understand the mechanisms of tumorigenesis caused by EC‐associated *FBXW7*, *PTEN*, and *TP53* mutations through functional analysis of GEMM. We bred mice expressing Cre recombinase from the progesterone receptor locus (PR‐Cre; Soyal *et al*, 2005; which drives recombination in the endometrium and female reproductive tract from 2 weeks post‐natal age) with mice carrying *Pten* ^fl^ or *Trp53* ^fl^ conditional knockout alleles and/or *Trp53* ^fl(R172H)^ (corresponding to human *TP53* ^R175H^) and *Fbxw7* ^fl(R482Q)^ (corresponding to human *FBXW7* ^R479Q^) conditional knock‐in alleles (Marino *et al*, 2000; Suzuki *et al*, 2001; Olive *et al*, 2004; Davis *et al*, 2011 *)* carrying EC hotspot missense mutations to generate control and experimental mice (Table 1 and Fig 1B) with the following alterations in the uterus:

- Cre expression only (WT)
- Knock‐in of *Fbxw7* hotspot missense mutation (*Fbxw7* ^mut^)
- Pten deletion (*Pten* ^del^)
- Pten deletion plus *Fbxw7* hotspot missense mutation (*Pten* ^del^; *Fbxw7* ^mut^)
- p53 deletion (*Trp53* ^del^)
- p53 deletion plus *Fbxw7* hotspot missense mutation (*Trp53* ^del^; *Fbxw7* ^mut^)
- p53 hotspot missense mutation (*Trp53* ^mut^)
- p53 hotspot missense and Fbxw7 hotspot missense mutations (*Trp53* ^mut^; *Fbxw7* ^mut^)

**Table 1:** Genetically engineered mouse models (GEMM) used in study.

Allelic composition^a^	Genetic modification post‐recombination	Predicted alteration in uterus	Referred to as
PR^cre/+^	Unaltered	Nil	WT
PR^cre/+^; *Fbxw7* ^fl(R482Q)/+^	*Fbxw7* ^R482Q/+^	Knock‐in of heterozygous Fbxw7 hotspot mutation	Fbxw7^mut^
PR^Cre/+^; *Pten* ^fl/fl^	*Pten* ^del/del^	Pten deletion	Pten^del^
PR^Cre/+^; *Pten* ^fl/fl^; *Fbxw7* ^fl(R482Q)/+^	*Pten* ^del/del^; *Fbxw7* ^R482Q/+^	Pten deletion plus heterozygous Fbxw7 hotspot mutation	Pten^del^; Fbxw7^mut^
PR^Cre/+^; *Trp53* ^fl/fl^	*Trp53* ^del/del^	p53 deletion	p53^del^
PR^Cre/+^; *Trp53* ^fl/fl^; *Fbxw7* ^fl(R482Q)/+^	*Trp53* ^del/del^; *Fbxw7* ^R482Q/+^	p53 deletion plus heterozygous Fbxw7 hotspot mutation	p53^del^; Fbxw7^mut^
PR^Cre/+^; *Trp53* ^fl(R172H)/fl^	*Trp53* ^R172H/del^	Knock‐in of heterozygous Trp53 hotspot mutation	p53^mut^
PR^Cre/+^; *Trp53* ^fl(R172H)/fl^; *Fbxw7* ^fl(R482Q)/+^	*Trp53* ^R172H/del^; *Fbxw7* ^R482Q/+^	Knock‐in of heterozygous Trp53 and Fbxw7 hotspot mutations	p53^mut^; Fbxw7^mut^

^a^ All alleles are wild‐type unless explicitly stated otherwise.

Females were culled at four and eight weeks (4w, 8w) for phenotyping and also aged for survival analysis. Analysis of uteri at 4w and 8w confirmed allelic recombination, expression of *Fbxw7* ^R482Q^ and *Trp53* ^R172H^ mutations and reduction in Pten and Trp53 mRNA and protein in *Pten* ^del^ and *Trp53* ^del^ mice respectively (Fig 1B–F; Appendix Fig S2A and B). This also revealed apparent interactions between *Fbxw7*, *Pten* and *Trp53* at the level of transcription. *Fbxw7* ^mut^ caused an increase in global *Fbxw7* expression (*P* < 0.05, Mann–Whitney test) which was lost with concomitant *Pten* ^del^ but not *Trp53* ^del^. *Fbxw7* ^mut^ and *Pten* ^del^ uteri showed increased *Trp53* expression at 4w, although by 8w this effect was lost or reversed (Fig 1D). We also noted possible interactions at the post‐transcriptional level. *Trp53* ^del^ uteri had increased Pten protein on immunoblotting (Fig 1E), despite similar *Pten* expression, while *Tp53* ^mut^ uteri showed substantially increased p53 immunostaining to controls despite similar *Trp53* expression (Fig 1F).

**Figure 2:**
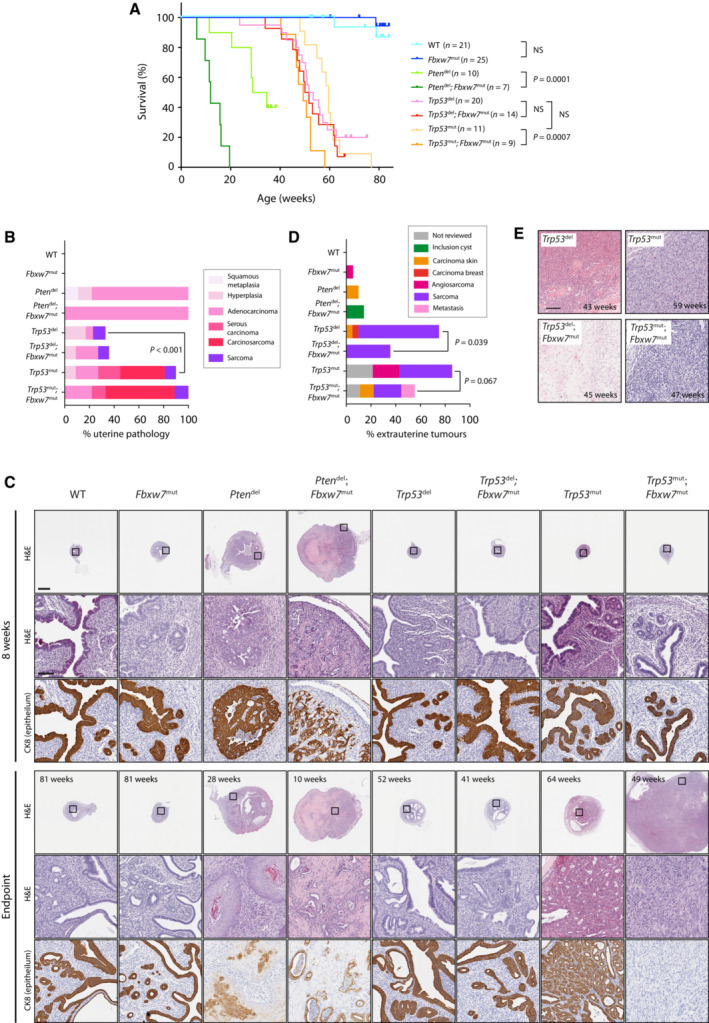
*Fbxw7* hotspot mutation accelerates Pten and Trp53‐driven endometrial cancer but does not accelerate extrauterine tumour development Kaplan–Meier curves showing survival of genetically engineered mouse models (GEMM) of indicated genotypes and group sizes. Statistical comparison between groups was performed by unadjusted log‐rank test.Prevalence of uterine pathology in GEMM shown in (A) at study endpoint as graded by expert pathologist. Statistical comparison between the proportion of mice with epithelial endometrial malignancy (ie carcinoma or carcinosarcoma) in groups was made by unadjusted Fisher exact test.Low and high power images of GEMM uteri at 8 weeks age and at study endpoint after H&E staining or IHC for cytokeratin 8 (CK8) (epithelia). Scale bars in low and high magnification panels indicate 1,000 and 100 μm respectively. Images are representative of a minimum of 5 mice per genotype per timepoint, with exception of wild‐type and *Fbxw7* ^mut^ mice at 8 weeks age where 3 mice were analysed.Prevalence and type of external tumours in GEMM shown in (A) at study endpoint as graded by expert pathologist. Statistical comparison of the proportion of mice with external tumours of any histotype between groups was made by Fisher exact test.H&E‐stained sections of high‐grade external sarcomas in *Trp53*del, *Trp53* ^del^; *Fbxw7*mut, *Trp53* ^mut^ and *Trp53* ^mut^; *Fbxw7* ^mut^ mice. Images are representative of minimum of 3 mice examined for each genotype. Scale bar indicates 100 μm. Source data are available online for this figure.

Consistent with previous work (Daikoku *et al*, 2008), Pten deletion caused endometrial cancer as early as 4 weeks age (Appendix Fig S3), with mice requiring sacrifice for uterine tumours from 11 weeks age (Fig 2A–C). A single *Pten* ^del^ mouse developed an extrauterine tumour in the form of an inclusion cyst containing a squamous cell carcinoma (Fig 2D). In contrast, *Trp53* ^del^ mice showed no evidence of endometrial neoplasia at early timepoints, but instead developed external tumours requiring sacrifice from 24 weeks age, most of which were found to be soft tissue sarcomas on pathologist review (Fig 2C–E). One *Trp53* ^del^ female culled for an external tumour at 57 weeks age was found to also have an endometrial carcinoma on pathological review, and another two *Trp53* ^del^ mice culled for external tumours at 51 and 68 weeks age had uterine sarcomas (Fig 2A). While some *Trp53* ^mut^ females also required sacrifice for external tumours, an appreciable fraction were culled for abdominal distension or genital bleeding, found to be secondary to uterine tumours on necropsy. Although survival of *Trp53* ^mut^ mice was not significantly different to that of *Trp53* ^del^ animals, and pathology review revealed similar frequency of extrauterine tumours (predominantly sarcomas) to *Trp53* ^del^ mice (Fig 2A, D, and E), the frequency of endometrial carcinoma in *Trp53* ^mut^ mice was far greater, with 8 of 11 (72.7%) developing epithelial malignancies during the study (*P* = 3e−04 vs. *Trp53* ^del^, Fisher exact test) (Fig 2A–C). Interestingly, *Fbxw7* ^mut^ mice showed no evidence of endometrial neoplasia up to the study endpoint, although a single animal developed an external angiosarcoma at 72 weeks age (Fig 2A–C).

We speculated that the tumorigenic effect of a cancer‐associated *Fbxw7* missense mutation may be unmasked when combined with another driver. Consistent with this prediction, *Fbxw7* ^mut^ dramatically accelerated endometrial tumorigenesis and reduced survival in *Pten* ^del^ mice, with *Pten* ^del^; *Fbxw7* ^mut^ compound mutants requiring sacrifice as early as 6 weeks for large, high‐grade endometrial tumours (Fig 2A–C; Appendix Fig S3). *Fbxw7* ^mut^ also significantly accelerated endometrial neoplasia and reduced survival of *Trp53* ^mut^ mice, but interestingly not *Trp53* ^del^ mice (*P*_INTERACTION_ = 0.13) (Fig 2A–C). Intriguingly, in contrast to its tumour promotion in the uterus in *Trp53* ^mut^ females, the addition of *Fbxw7* ^mut^ resulted in significantly lower frequency of extrauterine tumours in *Trp53* ^del^ mice (*P* = 0.039, Fisher exact test), and to a lesser extent in *Trp53* ^mut^ mice, though the latter was not statistically significant (Fig 2D).

We analysed endometrial tumours from the GEMM in detail. Expert gynaecological pathology review of endometrial cancers from *Pten* ^del^ and *Pten* ^del^; *Fbxw7* ^mut^ mice revealed most were adenocarcinomas of endometrioid histotype; with none displaying serous or carcinoscarcomatous morphology, in contrast to the recent study of Cuevas *et al* (2019) (Fig 2B). *Pten* ^del^ and particularly *Pten* ^del^; *Fbxw7* ^mut^ endometrial cancers displayed downregulation of the endometrial tumour suppressor Foxa2 (Appendix Fig S4), and frequent abnormal p53 immunostaining (Appendix Fig S5). The few endometrial carcinomas which occurred in *Trp53* ^del^, and *Trp53* ^del^; *Fbxw7* ^mut^ animals were of endometrioid histotype, although one tumour displayed sarcomatoid appearances insufficient for formal diagnosis of carcinosarcoma. In contrast, *Trp53* ^mut^ (3 of 11) and particularly *Trp53* ^mut^; *Fbxw7* ^mut^ females (5 of 9) developed carcinosarcoma, characterised by typical morphology and loss of the epithelial marker cytokeratin 8 (Krt8; Fig 2B and C). The difference in carcinosarcoma prevalence between *Pten* ^del^ ± *Fbxw7* ^mut^ and *Trp53* ^mut^ ± *Fbxw7* ^mut^ mice was statistically significant (*P* = 0.0043, Fisher exact test). *Trp53* ^mut^ mice also developed serous endometrial cancers in several cases, though the difference in prevalence with *Pten* ^del^ mice was not statistically significant. *Trp53* ^mut^ and *Trp53* ^mut^; *Fbxw7* ^mut^ endometrial tumours also often displayed abnormal p53 immmunostaining, with variable patterns observed (Appendix Fig S5).

### Transcriptomic profiling of murine and human tumours, human isogenic cell lines and in silico analysis identifies Wnt pathway effectors LEF1 and TCF7L2 as candidate FBXW7 substrates

**Figure 3:**
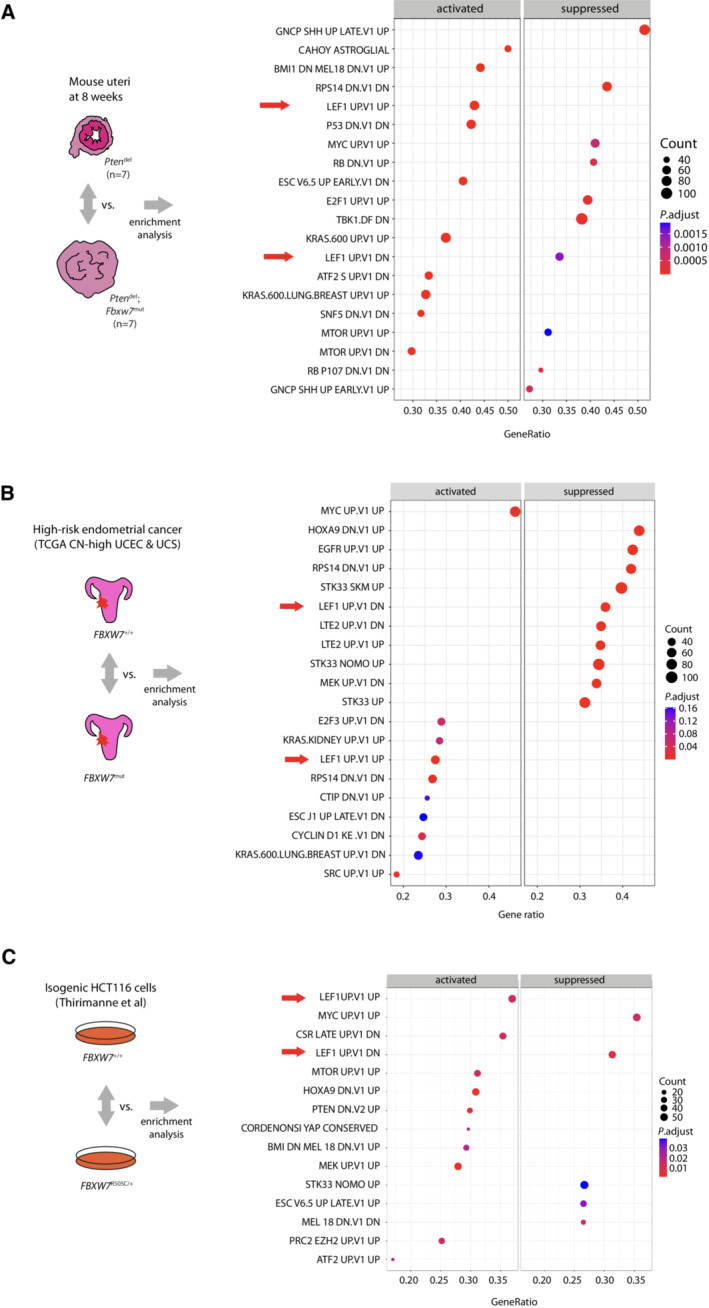
*Fbxw7* hotspot mutation drives enrichment of LEF1 signalling in GEMM uteri and human cancer cells (Left panel) Schematic illustrating gene expression profiling of mouse uterine samples of indicated genotypes and group sizes at 8 weeks age, with groups compared by enrichment analysis. (Right panel) Dot plot indicating enrichment of MSigDB C6 oncogenic gene sets in *Pten* ^del^; *Fbxw7* ^mut^ uteri vs. *Pten* ^del^ uteri. Arrows indicate enrichment of LEF1 signatures.(Left panel) Schematic illustrating comparison of high‐risk endometrial cancers from UCEC and UCS cohorts (see Results for definition) by enrichment analysis according to pathogenic *FBXW7* missense mutation status. (Right panel) Dot plot indicating enrichment of MSigDB C6 signatures in *FBXW7* mutant tumours. Arrows indicate enrichment of LEF1 signatures.(Left panel) Schematic illustrating comparison of isogenic parental *FBXW7* wild‐type (*FBXW7* ^+/+^) and heterozygous WD40 hotspot missense mutant (*FBXW7* ^R505C/+^) isogenic HCT116 cells reported by Thirimanne *et al* (2022) by enrichment analysis following pre‐ranking. (Right panel) Dot plot indicating enrichment of MSigDB C6 oncogenic sets in *FBXW7* ^R505C/+^ cells. Arrows indicate enrichment of LEF1 signatures. Source data are available online for this figure.

We investigated the mechanisms of *Fbxw7* ^mut^ ‐driven tumorigenesis in the GEMM uteri. Gene expression profiling and gene set enrichment analysis (GSEA) revealed no differentially‐expressed genes (DEGs), and a small number of significantly enriched gene sets between *Fbxw7* ^mut^ and wild‐type uteri at 8 weeks age, paralleling the minimal perturbation of uterine *Fbxw7* and *Trp53* expression at this timepoint (Datasets [Link], [Link]; Appendix Fig S6). In contrast, analysis of *Pten* ^del^ and *Pten* ^del^; *Fbxw7* ^mut^ compound mutant uteri revealed 788 DEGs (FDR < 0.05) and multiple enriched gene sets (Fig 3A; Datasets [Link], [Link]; Appendix Fig S7). These included those corresponding to epithelial‐mesenchymal transition (EMT) (the most enriched hallmark gene set), dysregulation of p53 signalling (consistent with the increased p53 protein on immunostaining, although we note that enrichment of p53 gene sets is not specific for alterations in p53 itself), signalling by known FBXW7 substrate Myc, and multiple other oncogenic cellular signalling pathways (Fig 3A). Among these, LEF1 signalling caught our attention, owing to its strong upregulation, its critical role in endometrial gland development (adenogenesis; Mericskay *et al*, 2004; Shelton *et al*, 2012) and its role as an effector in the Wnt pathway, which is recurrently dysregulated in cancer. We confirmed upregulation of Lef1 targets and significant DEGs Mmp13 and Wisp1 (Ccn4) in *Pten* ^del^; *Fbxw7* ^mut^ compound mutant uteri by qRT–PCR, (Appendix Fig S8A and B), and excluded differential infiltration of neoplastic uteri by Lef1‐expressing lymphocytes as a confounder (Appendix Fig S9A and B).

**Figure 4:**
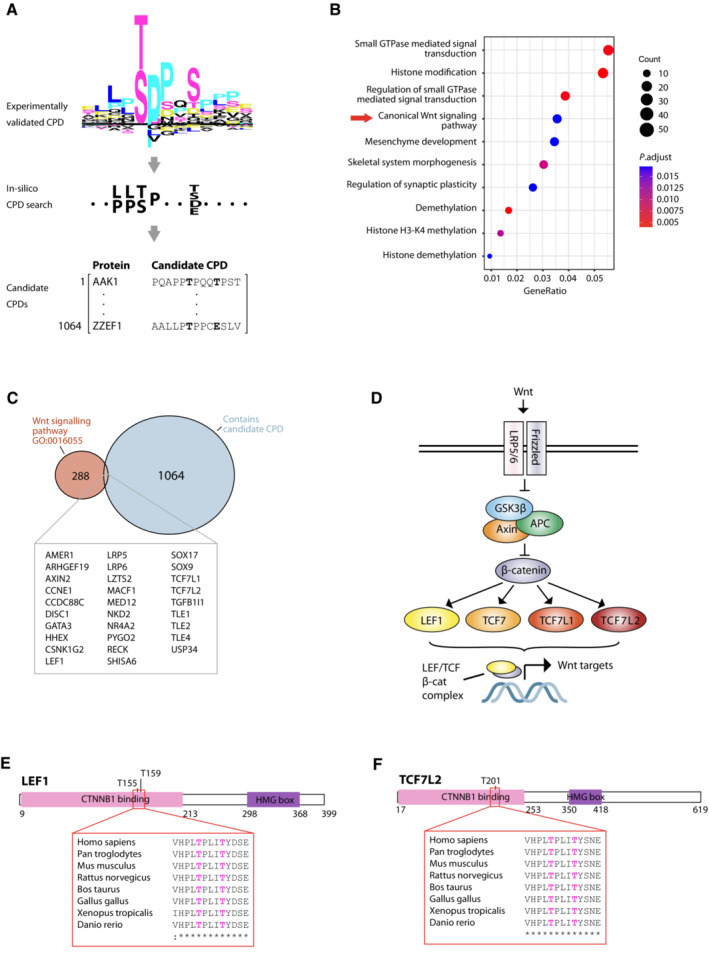
*In silico* identification of candidate *FBXW7* targets reveals enrichment of Wnt pathway components including LEF1 and TCF7L2 Schematic indicating workflow for *in silico* identification of candidate FBXW7 targets carrying putative conserved phosphodegrons (CPDs). Experimentally validated FBXW7 CPDs (top, provided as Dataset EV8) were used to define a consensus motif (middle) for search against the human proteome to identify candidate FBXW7 targets (bottom).Dot plot showing enrichment analysis of 1,064 putative FBXW7 substrates against GO biological processes.Venn diagram showing overlap between putative FBXW7 substrates identified by *in silico* analysis and Wnt pathway components curated by the Gene Ontology Consortium (GO:0016055).Simplified representation of Wnt signalling pathway. Following stimulation by Wnt ligand, the GSK3β/Axin/APC destruction complex is inhibited and β‐catenin can translocate to the nucleus where it complexes with TCF/LEF family transcription factors to regulate Wnt target gene expression.Schematic of putative FBXW7 substrate LEF1 indicating position and conservation of candidate CPD, and phosphorylation sites corresponding to the ‘0’ and ‘+4’ positions curated in PhosphoSitePlus database.Corresponding schematic for putative FBXW7 substrate TCF7L2. Source data are available online for this figure.

We sought to confirm the association of *FBXW7* mutation with LEF1 signalling in human samples. GSEA of high‐risk endometrial cancers from TCGA (CN high UCEC and UCS) revealed enrichment of LEF1 signalling in cases with *FBXW7* missense driver mutation (Fig 3B; Dataset EV7). Similarly, GSEA of RNAseq data from a recent study (Thirimanne *et al*, 2022) of isogenic human colon cancer cells with wild‐type or oncogenic missense mutant *FBXW7* (*FBXW7* ^R505C^) identified LEF1 signalling as the most enriched gene set in *FBXW7* ^R505C^ cells, supporting a causal relationship. LEF1 signalling was also significantly enriched in isogenic cells with *FBXW7* deletion, though this effect was weaker (Fig 3C; Appendix Fig S10). Interestingly, while as in the GEMM tumours *FBXW7* mutation was associated with enrichment of MYC signalling in the human endometrial cancers, this was not the case in the colon cancer cells, and dysregulation of p53 gene sets was less evident in both, possibly owing to different p53 targets in mouse and human, or mutations in *TP53* and other genes in its pathway in the human samples (Fig 3A–C). To explore the mechanistic basis of the LEF1 pathway enrichment with *FBXW7* mutation, we performed an *in silico* search for novel FBXW7 targets using a CPD sequence derived from experimentally validated substrates (Materials and Methods; Fig 4A; Dataset EV8). The 1,064 candidates (Dataset EV9) contained significant overrepresentation of Wnt pathway components, including LEF1 and other transcriptional effectors TCF7L1 and TCF7L2 (Fig 4B–D), but not β‐catenin – noteworthy given previous data suggesting β‐catenin is an FBXW7 substrate (Jiang *et al*, 2016). Importantly, the putative CPD in LEF1 and TCF7L2 was highly conserved, consistent with a functional role (Fig 4E and F).

### LEF1 and TCF7L2 are novel FBXW7 substrates expressed in proliferative endometrial stem cells and dysregulated by *Fbxw7/FBXW7* hotspot mutation

**Figure 5:**
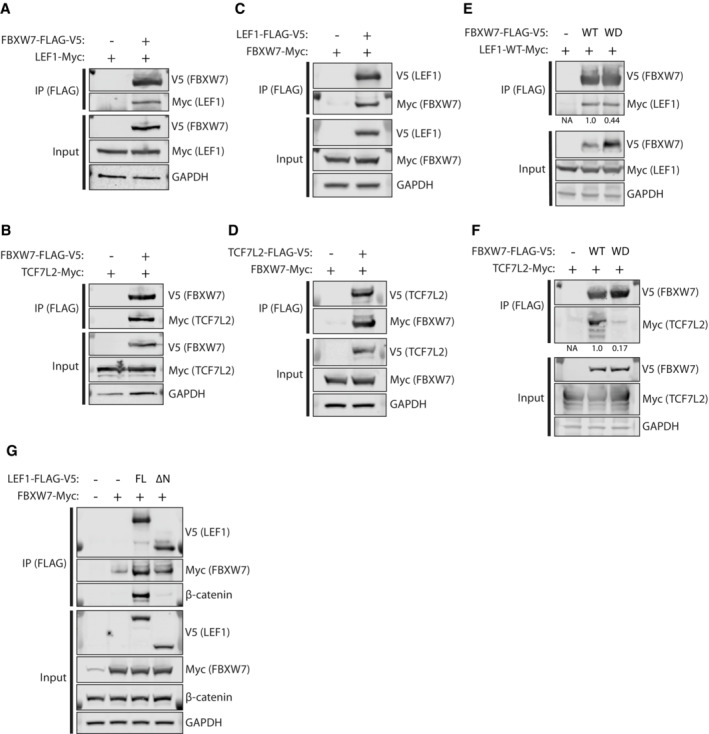
LEF1 and TCF7L2 are novel FBXW7 substrates whose interaction is disrupted by FBXW7 WD40 hotspot mutation HEK293T cells were transfected with constructs encoding FLAG‐V5‐tagged FBXW7α and Myc‐tagged LEF1. FLAG‐V5‐tagged FBXW7 α was immunoprecipitated (IP) from cell extracts with anti‐FLAG antibody before immunoblotting for tagged proteins as indicated (upper panels). Lower panels show inputs.HEK293T cells were transfected with constructs encoding FLAG‐V5‐tagged FBXW7α and Myc‐tagged TCF7L2. IP was performed as in (A) before immunoblotting for tagged proteins as indicated.Reciprocal experiment to (A) in which FLAG‐V5‐tagged LEF1 was pulled down by IP.Reciprocal experiment to (B) in which FLAG‐V5‐tagged TCF7L2 was pulled down by IP.Complementary experiment to (A) in which HEK293T cells were transfected with either FLAG‐V5‐tagged wild‐type FBXW7α (WT) or a substrate‐binding mutant (WD40), in which three arginine residues within one of the FBXW7 WD40 repeats have been mutated. Numbers below upper bands indicate densitometric quantification of the ratio of LEF1‐Myc to FBXW7 α‐V5 following IP by anti‐FLAG antibody, with WT normalised to 1.Complementary experiment to (B), of similar design to (E) but using Myc‐tagged TCF7L2 rather than LEF1.HEK293T cells were transfected with Myc‐tagged FBXW7α and either FLAG‐V5‐tagged full length LEF1 (FL) or truncated LEF1 in which the N terminal domain required for β‐catenin binding has been deleted. Immunoblotting for indicated proteins was performed following IP by anti‐FLAG antibody. Data information: All images shown are representative of a minimum of two independent experiments; densitometry results indicate mean of three independent experiments. Source data are available online for this figure.

We investigated interactions between FBXW7 and these putative novel targets. LEF1 and TCF7L2 were detected in immunoprecipitates following FBXW7 pulldown in HEK293T cells (Fig 5A and B), and a reciprocal experiment confirmed presence of FBXW7 in LEF1 and TCF7L2 immunoprecipitates (Fig 5C and D). Analysis of the interaction of LEF1 with WD40 domain mutant FBXW7, defective in substrate binding, was complicated by robust and reproducibly greater levels of WD40 mutant than wild‐type FBXW7 protein following transfection (Fig 5E). However, densitometric quantification confirmed the amount of LEF1 relative to immunoprecipitated FBXW7 was reproducibly lower with WD40 mutant than wild type FBXW7 (see values under band) (Fig 5E). Interestingly, wild type and WD40 domain mutant FBXW7 protein levels were similar when co‐transfected with TCF7L2, and the reduction in the interaction of TCF7L2 with the WD40 domain‐mutant FBXW7 was more obvious (Fig 5F). The proven FBXW7‐LEF1 interaction, and the absence of a candidate FBXW7 CPD in β‐catenin in the *in silico* analysis caused us to speculate that the previously reported FBXW7–β‐catenin interaction could in fact be mediated indirectly via LEF1 rather than through direct interaction. In keeping with this hypothesis, immunoprecipitates of full length LEF1 contained both FBXW7 and β‐catenin, while those from a truncated LEF1 lacking the β‐catenin‐interacting N terminal domain contained FBXW7 but not β‐ catenin (Fig 5G).

**Figure 6:**
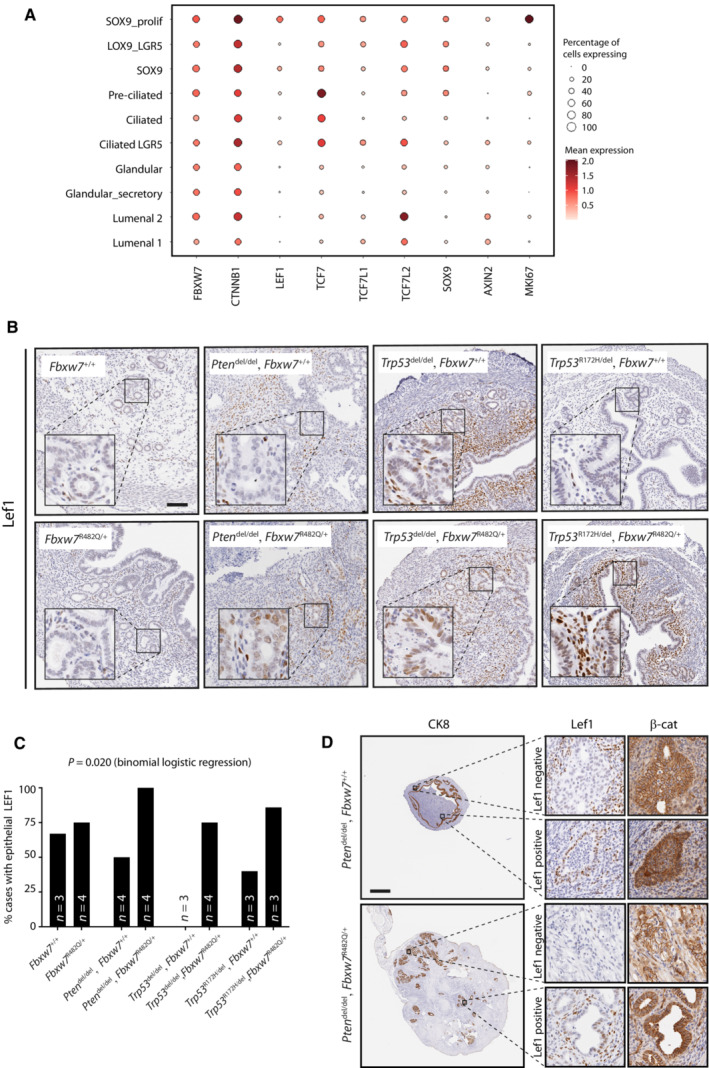
LEF1 is highly expressed in proliferative endometrial stem cells and dysregulated by Fbxw7 hotspot mutation Dot plot showing log2‐transformed expression of FBXW7, LEF1 and other wnt pathway genes in endometrial epithelial cell subsets defined by single cell RNAseq (scRNAseq) in Garcia‐Alonso *et al* (2021).Representative LEF1 immunohistochemistry in GEMM uteri from mice of indicated genotypes at 8 weeks age. For each of the four columns (i.e genotypes), upper and lower images are taken from littermate females housed in the same cage. Images are representative of 3–4 mice as shown in (C). Scale bar indicates 100 μm.Percentage of mice with epithelial LEF1 expression in endometria at 8 weeks age according to genotype. *P* value was derived from binomial logistic regression with epithelial LEF1 expression as the dependent variable.Immunohistochemistry for LEF1 and β‐catenin from endometrial carcinomas in *Pten* ^del^ ± *Fbxw7* ^mut^ mice. High power images show representative staining in LEF1 negative and positive glands. Scale bar indicates 1,000 μm. Source data are available online for this figure.

As noted above, LEF1/Lef1 is essential for adenogenesis in several tissues, including the endometrium (van Genderen *et al*, 1994; Shelton *et al*, 2012). We investigated its role in endometrial physiology using human scRNAseq data from a recent study (Garcia‐Alonso *et al*, 2021). LEF1 expression was greatest in proliferating cells defined by the stem cell marker SOX9 (Fig 6A), signatures of which are enriched in human endometrial cancers (Garcia‐Alonso *et al*, 2021). Epithelial Lef1 expression in GEMM endometria at 8 weeks age was more common in *Fbxw7* ^mut^ mice than controls (*P* = 0.02, logistic regression) (Fig 6B and C), with ectopic expression outside the gland base stem/progenitor cell niche (Syed *et al*, 2020) (e.g. luminal positivity in bottom right image in Fig 6B), and associated nuclear β‐catenin localisation (Morgan *et al*, 2019) (Fig 6D). Interestingly, analysis of high‐grade human endometrial cancers of no specific molecular profile (NSMP) and p53 mutant subtypes and murine endometrial cancers from *Pten* ^del^ and *Trp53* ^mut^ GEMM at experimental endpoint revealed near‐universal epithelial LEF1/Lef1 expression, irrespective of *FBXW7*/*Fbxw7* mutation (Appendix Figs S11A and B, and S12A and B), suggesting that *FBXW7/Fbxw7* wild‐type tumours may employ alternative mechanisms of Lef1 upregulation, as has been suggested by earlier studies of mouse and human tumours (Shelton *et al*, 2012; Ruz‐Caracuel *et al*, 2021).

Unfortunately, infertility of *Pten* ^del^; *Fbxw7* ^mut^ females (owing to the rapid onset of endometrial neoplasia) precluded breeding to generate GEMM with concomitant Lef1 deletion, meaning that we were unable to examine whether the tumour phenotype was rescued by Lef1 loss. Similarly, our attempts to define the effects of *FBXW7* mutation and PTEN loss in normal human endometrial cells proved unsuccessful, as hEM3 cells targeted with *FBXW7* mutation failed to expand from single cell colonies.

## Discussion

In this study, we used GEMM to define the functional consequences of high‐risk endometrial cancer driver mutations, and human tumours and isogenic cell lines to confirm our key results. We found that while *Fbxw7* hotspot missense mutation fails to induce endometrial neoplasia on its own, it significantly accelerates endometrial tumorigenesis caused by *Pten* deletion or *Trp53* hotspot mutation, with many tumours showing characteristic features of human carcinosarcomas in the latter case. Interestingly, *Fbxw7* mutation did not potentiate extrauterine tumorigenesis caused by *Trp53* deletion, consistent with tissue‐specific variation in the selective advantage it confers in malignancy. Taking an agnostic approach, we identified enrichment of a gene signature corresponding to the Wnt pathway effector LEF1 in *Fbxw7* mutant mouse endometrial cancers. We confirmed this association in human endometrial cancers and used isogenic human colorectal cancer cells to provide strong evidence that this relationship is causal. Finally, we confirmed LEF1 and its LEF/TCF family member TCF7L2 as novel FBXW7 substrates by immunoprecipitation. Our results provide new insights into the biology of high‐risk endometrial cancer and suggest that strategies targeting the Wnt pathway may be worthy of investigation in endometrial tumours with *FBXW7* mutations.

Cuevas *et al* (2019) recently reported that co‐deletion of Pten and Fbxw7 in the mouse endometrium using an alternative *BAC‐Sprr2f‐Cre* recombinase caused EMT and carcinosarcomas with complete penetrance following somatic acquisition of *Trp53* mutations (Cuevas *et al*, 2019). We found similar enrichment of EMT signatures in neoplastic uteri in our *Pten* ^del^; *Fbxw7* ^mut^ mice; however none of these animals developed carcinosarcoma. This may relate to the reduced latency of tumorigenesis in our model, or differing effects of *Fbxw7* loss and heterozygous missense mutation, as has been demonstrated in other cancer types (King *et al*, 2013). While formal comparison of *Fbxw7* missense mutation and loss was beyond the scope of this study, it is interesting to note that *Trp53* missense mutation was a far stronger driver of endometrial tumorigenesis than *Trp53* loss in our GEMM. The development of high‐grade endometrial tumours with features of carcinosarcomas in *Trp53* ^mut^ mice, and their potentiation by *Fbxw7* mutation is consistent with a central role of *TP53* and *FBXW7* in human carcinosarcoma. Furthermore, the failure of *Fbxw7* ^mut^ to accelerate extrauterine tumours caused by *Trp53* loss or mutation supports the hypothesis that Fbxw7 mutation confers greater selective advantage in endometrial malignancy, though this is currently unproven.

The recent discovery that cancer driver mutations are common in normal tissue (Moore *et al*, 2020) poses important questions about the steps required for malignant transformation. Our initial surprise at the failure of heterozygous *Fbxw7* mutation to induce endometrial neoplasia was lessened by the detection of potentially compensatory alterations in gene expression. Upregulation of Fbxw7 expression would be expected to increase the amount of functional Fbxw7 homodimers, thus maintaining physiological levels of substrates, while induction of a p53 response is a well‐characterised block on tumorigenesis (Chen *et al*, 2005). Whether this is the case in normal human endometrial glands with *FBXW7* mutation is unknown. Interestingly, *Fbxw7* ^mut^ ‐ induced *Fbxw7* upregulation was not detected in the background of *Pten* loss, raising the possibility that Pten may function in a feedback loop, the loss of which contributes to tumorigenesis. Further examination of this, and the mechanisms by which the p53 response is bypassed in mouse and human tumours appears merited.

Since its early characterisation as a ubiquitin ligase for cyclin E (Koepp, 2001; Moberg *et al*, 2001; Strohmaier *et al*, 2001), FBXW7 has been shown to regulate a large and increasing number of substrates (Welcker & Clurman, 2008; Davis *et al*, 2014b). While many have proven oncogenic function, it is currently unclear which drive tumorigenesis in the various cancer types in which *FBXW7* is recurrently mutated, as these may vary by tissue (Davis *et al*, 2014a). LEF1 is a Wnt pathway effector and transcriptional activator essential for endometrial adenogenesis (Shelton *et al*, 2012). LEF1 is frequently overexpressed in murine and human endometrial cancers and has also been shown to be a key mediator of EMT (Medici *et al*, 2006; Freihen *et al*, 2020; Ruz‐Caracuel *et al*, 2021). Taking these observations together with our data, it appears plausible that failure of *Fbxw7* missense‐mutant endometrial cancers to degrade Lef1 may contribute to tumorigenesis and EMT in our GEMM. As we were unable to test whether Lef1 deletion attenuated tumorigenesis in our mice, defining this will be a priority, particularly given that Lef1 ablation in a murine intestinal tumour model unexpectedly increased tumour development (Heino *et al*, 2021). Whether TCF7L2 dysregulation contributes to *FBXW7*‐mutant endometrial cancer is less clear. TCF7L2 can act as an activator or repressor of Wnt targets depending on cellular context (Struewing *et al*, 2010), and has been characterised as a tumour suppressor in colorectal cancer (Wenzel *et al*, 2020). Understanding the tissue‐specific variation in LEF1 and TCF7L2, along with other FBXW7 substrates will be important topics for future investigation.

Our study has several strengths. These include the large number of alleles examined, our use of clinically relevant hotspot missense *Fbxw7* and *Trp53* mutations, detailed tumour phenotyping including expert pathological review, agnostic approach to identify novel candidate FBXW7 substrates which were formally confirmed by co‐immunoprecipitation, and confirmation of LEF1 signature in human endometrial cancers with *FBXW7* mutation. It also has limitations. Our focus on endometrial tumours meant we did not undertake detailed characterisation of extrauterine lesions beyond basic histopathological analysis. Transcriptomic analyses were of bulk tissue, and it will be important to define the effects of genetic manipulation within the epithelial compartment using single‐cell sequencing and spatial transcriptomic approaches. Logistical considerations caused us to employ a single Cre recombinase and to focus on a single *Fbxw7* hotspot missense mutation. It will be important to define whether recombination in non‐epithelial cells with PR‐Cre influenced our results and to examine the impact of substitution of other WD40 propellor‐tip residues (eg R465, R505) in endometria. Similarly, the *Trp53* R172H allele used in this study was chosen for pragmatic reasons rather than its prevalence in high‐risk endometrial cancer (6% of oncogenic *TP53* point mutations in high‐risk EC in the TCGA studies) and it will be of interest to examine the effects of orthologues of more common mutations such as *TP53* R273H (14% of oncogenic *TP53* point mutations in TCGA). As noted above, we were unable to test whether LEF1 loss ameliorated tumour development in *Pten* ^del^; *Fbxw7* ^mut^ mice. Development of inducible endometrial Cre recombinases would permit this, and the analysis of other genetic alterations in combination, and should be a priority for the field. Finally, our attempts to introduce *FBXW7* missense mutation into immortalised human endometrial cells were unsuccessful, and it will be of interest to develop methods to permit this in cells or organoids in the future.

In summary, we show that while *Fbxw7* hotspot missense mutation is insufficient to cause endometrial neoplasia in isolation, it potently accelerates tumorigenesis caused by *Pten* deletion or *Trp53* mutation, potentially by failure to degrade the Wnt pathway effector Lef1. Given the morbidity and mortality of high‐risk endometrial cancer (León‐Castillo *et al*, 2020; Crosbie *et al*, 2022), further investigation of this pathway as a therapeutic target appears worthwhile.

## Materials and Methods

The study confirms to all relevant ethical regulations of the University of Oxford.

### Human cancers

Publicly available TCGA data for uterine corpus endometrial cancer (UCEC) and uterine carcinosarcoma (UCS) were downloaded from CBioportal (https://www.cbioportal.org). UCEC data were filtered to include only high‐risk copy number high (CN‐high) cases. Mutations were classified as oncogenic based on the criteria used by TCGA which incorporates data from OncoKB (Chakravarty *et al*, 2017), CIViC (Griffith *et al*, 2017), MyCancerGenome (https://www.mycancergenome.org), Cancer Hotspots (Chang *et al*, 2018) and 3D Cancer Hotspots (Gao *et al*, 2017). TCGA RNAseq data were downloaded from the Genomic Data Commons Data portal (https://portal.gdc.cancer.gov), details of processing and analysis are provided below. High‐grade (grade 3) endometrial cancers were identified from the databases of the PORTEC2 trial and a prospective cohort of endometrial cancers from the Medisch Spectrum Twente (MST), Enschede (NL). Molecular subtyping of tumours was done as previously reported (Vermij *et al*, 2023). Ethical approval for analysis of samples was provided by the Leiden‐Den Haag‐Delft medical ethics committee. Participants in the PORTEC 2 trial provided informed consent for sample analysis, for the MST cohort a waiver for informed consent for anonymised analysis of samples was given by the ethics committee. All experiments conformed to the principles set out in the WMA Declaration of Helsinki and the Department of Health and Human Services Belmont Report. Inclusion and exclusion criteria for the PORTEC2 trial have been reported previously. The MST real‐world cohort did not apply specific inclusion or exclusion criteria. Cases were selected for analysis based on known *FBXW7* mutation status and availability of FFPE slides for immunohistochemistry. No randomisation was performed.

### Mice

Mouse breeding and experiments were performed at the Functional Genomics Facility, Wellcome Centre for Human Genetics, University of Oxford, under UK Home Office Project Licence PPL PDF0B94C3, by appropriately trained, PIL licenced researchers (PILs I8E73D7F0 and ID4B695EE).

Details of generation of Fbxw7^fl(R482Q)^ conditional knock‐in mice have been reported previously (Davis *et al*, 2011, 2014a). Pten^tm2Mak^ (*Pten* ^fl^) mice (Suzuki *et al*, 2001; MGI: 2182005) were imported from the laboratory of Bass Hassan (University of Oxford). *Trp53* ^tm1Brn^ (Trp53^fl^) mice (Marino *et al*, 2000; MGI:1931011) and B6.129S4(Cg)‐Trp53tm2.1Tyj/J (*Trp53* ^R172H^) mice (Olive *et al*, 2004; MGI:3039264) were imported from the laboratory of Xin Lu (University of Oxford). *Pgr* ^tm2(cre)Lyd^ (PR‐Cre) mice (Soyal *et al*, 2005) were imported from laboratory of Franco de Mayo (Baylor University). All mice were backcrossed onto a C57BL/6J background for at least 6 generations before use in experimental breeding. Details of primers used for genotyping are provided in Dataset EV10. Mice were housed at 18–24°C, relative humidity 30–70% under a 12 h light/dark cycle (07:00 to 19:00 light, 19:00 to 07:00 dark), with drinking water and chow provided ad libitum. Experimental females were checked daily for general health and tumour development, and sacrificed by Schedule 1 approved method at humane endpoint (hunching, bleeding, weight loss ≥ 15% from baseline, external tumour ≥ 10 mm). Experimental uteri and external tumours were harvested, before snap freezing in liquid nitrogen, embedding in Optimal Cutting Temperature Compound (OCT) (Agar Scientific) and/or fixation in 10% neutral buffered formalin (NBF) depending on sample.

### Tissue preparation and molecular/pathological analysis of mouse and human samples

DNA for routine genotyping was extracted from ear snips using the Hot Sodium Hydroxide and Tris (HotSHOT) method. Extraction of DNA and RNA from snap‐frozen tissues was performed using the Qiagen DNeasy and RNAeasy Plus kit respectively (Qiagen, Hilden, Germany) according to manufacturer's protocol and quantified by NanoDrop spectrophotometer (ThermoFisher, Waltham, MA, USA) or by Qubit (ThermoFisher). Confirmation of Cre‐mediated recombination of conditional alleles was done by PCR using primers detailed in Dataset EV10 (reaction conditions available on request). First strand synthesis of RNA was performed by High Capacity cDNA kit (Applied Biosystems, Waltham, MA, USA) using random primers as per manufacturer's instructions. cDNA was quantified by real time, reverse transcription PCR (RT–PCR) using TaqMan primer‐probes (ThermoFisher) on the QuantStudio 6 Flex system (Applied Biosystems). Tissue lysates were made by disruption of frozen mouse samples in RIPA buffer (ThermoFisher Scientific) containing protease (Roche, Basel, Switzerland) and phosphatase inhibitors (Sigma‐Aldrich, St Louis, MO, USA) at manufacturer's recommended concentrations, using a rotor‐stator homogeniser. Lysates were cleared by centrifugation at 16,000 *g* for 20 min at 4°C, with supernatant quantified using the CBX assay (G Biosciences, St Louis, MO, USA) according to manufacturer's instructions. Mouse samples for immunoblotting were heated to 70°C with NuPage LDS Sample Buffer (Invitrogen), loaded on NuPage Bis‐Tris gels (Invitrogen) and separated by electrophoresis before transfer to PVDF membrane. Membranes were then blocked for 1 h at room temperature (RT) in 5% milk diluted in tris‐buffered saline containing 0.1% Tween (TBST) and washed in TBST before incubation with primary antibody in 5% milk or bovine serum albumin (BSA) (Sigma Aldrich) overnight at 4°C. Membranes were then washed, and incubated with IRDye secondary antibody before imaging using a LI‐COR Odyssey CLx system (LI‐COR, Lincoln, NE, USA). Antibodies and concentrations used for immunoblotting are provided in Dataset EV11. Mouse samples for histological analysis were fixed in NBF at room temperature (RT) for < 24 h before processing and paraffin embedding. 4–5 μm sections were cut by microtome, and H&E staining performed by standard techniques. Pathological grading of H&E‐stained uteri and tumours was performed by two expert gynaecological pathologists (AL, TB) blinded to experimental genotype. Slides for immunohistochemistry were deparaffinised in xylene and rehydrated through graded alcohol series. Antigen retrieval was performed by boiling slides in 10 mM citrate buffer (pH 6.0) for 5 min at 120°C in a pressure cooker. Slides were allowed to cool and washed before peroxidase quench in 3% hydrogen peroxide (Sigma Aldrich) for 10 min, followed by further washes (2× in water and 1× in TBS) and blocking by incubation at RT for 1 h with 5% normal goat serum (Vector Laboratories, Newark, CA, USA). Slides were then incubated with primary antibody for 1 h at RT or overnight at 4°C and washed prior to addition of SignalStain Boost IHC Detection Reagent (Rabbit or Mouse; Cell Signalling Technology, Danvers, MA, USA) for 45 min at RT (details in Dataset EV10). Development of the slides was performed using 3–3′ diaminobenzidine (DAB) (Vector Laboratories) according to manufacturer's instructions. Slides were counterstained with haematoxylin, dehydrated and mounted with coverslips. Stained slides were scanned using Aperio CS2 Slide Scanner and viewed by Aperio ImageScope (version 12.4.3.5008; Leica Biosystems, Wetzlar, Germany).

LEF1 IHC on human endometrial cancers was performed on 4 μm FFPE slides in the Histopathology department of Leiden University Medical Centre (LUMC). Unstained slides were rehydrated via graded ethanol series, followed by endogenic peroxidase activity blocking (0.3% Methanol/H2O2) and antigen retrieval using a microwave oven procedure in 10 mmol/L Tris‐EDTA buffer, pH9.0 for 10 min. Tissue sections were incubated overnight with primary antibodies against LEF1 at 4°. A 30 min incubation with a secondary antibody (Poly‐HRP‐GAM/R/R; DPV0110HRP; ImmunoLogic) was then performed before visualisation with DAB (K3468, DAKO) haematoxylin counterstaining. Staining was reviewed and scored by an expert gyn pathologist (TB).

### Transcriptomic analysis

Total RNA samples from mouse uteri at 8 weeks age were analysed by TapeStation 4200 to determine sample RNA Integrity Number (RIN). Samples with a RIN ≥ 7 were analysed by Clariom™ S Expression Assays (Affymetrix, Santa Clara, CA, USA) at the Oxford Genomics Centre, Wellcome Centre for Human Genetics, University of Oxford. Data was obtained in the form of sample level, probe set intensities values, which were clustered by principal component analysis to confirm expected grouping. Data were then RMA normalised using the oligo package for R, and probe sets were annotated to their representative gene using the annotation file provided by the manufacturer. Differentially expressed gene (DEG) analysis was performed using the limma package for R. Gene set enrichment analysis (GSEA; Subramanian *et al*, 2005) was performed and results visualised using the ClusterProfiler (Yu *et al*, 2012) package in R.

TCGA RNAseq data were downloaded from the Genomic Data Commons (GDC: https://portal.gdc.cancer.gov), normalised and log2 transformed prior to GSEA using ClusterProfiler (Yu *et al*, 2012).

Differential gene expression from comparison of isogenic HCT116 wild‐type and FBXW7 hotspot mutant cells was downloaded from the supplementary data from Thirimanne *et al* (2022). DEGs were used as input for GSEA after pre‐ranking using ClusterProfiler.

### *In silico* CPD search

Experimentally validated FBXW7 phosphodegron sequences were curated from search of published literature using PubMed (Koepp, 2001; Welcker *et al*, 2004; Sundqvist *et al*, 2005; Mao *et al*, 2008; Galli *et al*, 2010; Liu *et al*, 2010; Inuzuka *et al*, 2011; Fukushima *et al*, 2012; Giráldez *et al*, 2014; Koo *et al*, 2015; Kourtis *et al*, 2015; Lv *et al*, 2015; Maskey *et al*, 2015; Chen *et al*, 2016; Kharat *et al*, 2016; Zhang *et al*, 2016; Zhao *et al*, 2016; Song *et al*, 2017; Wang *et al*, 2017; Dataset EV7). Phosphodegrons were tabulated, plotted using Seq2Logo web interface https://services.healthtech.dtu.dk/service.php?Seq2Logo‐2.0 (Thomsen & Nielsen, 2012), and used to derive a ‘validated’ CPD [LP][LP][TS]P..[TSDE] for searching against canonical human sequences listed in SwissProt (Bairoch & Apweiler, 1996) and Ensembl (Cunningham *et al*, 2022) using Bioconductor (Huber *et al*, 2015). Details of gene names, number of CPD matches, CPD match position, and match sequence were extracted, and compiled to generate a list of candidate substrates. Enrichment analysis of results against curated gene sets corresponding to biological processes was performed using ClusterProfiler (Yu *et al*, 2012).

### Co‐immunoprecipitation of tagged plasmids

Of the plasmids used in this work: pDONR223_FBXW7_WT was a gift from Jesse Boehm, William Hahn and David Root (Addgene plasmid #81795; http://n2t.net/addgene:81795; RRID: Addgene 81795; Kim *et al*, 2016), TCF7L2_pLX307 was a gift from William Hahn and Sefi Rosenbluh (Addgene plasmid #98373; http://n2t.net/addgene:98373; RRID: Addgene 98373; Rosenbluh *et al*, 2016), gateway entry vector containing the LEF1 CDS generated by the Human ORFeome Collection (Accession #EU446873) was obtained from the Cellular High Throughput Screening Group, Target Discovery Institute, University of Oxford, pMH‐MYC was a gift from Michael Huen (Addgene plasmid #101765; http://n2t.net/addgene:101765; RRID: Addgene 101765; An *et al*, 2017), pcDNA3.1‐3xFLAG‐V5‐ccdB was a gift from Susan Lindquist and Mikko Taipale (Addgene plasmid #87064; http://n2t.net/addgene:87064; RRID: Addgene 87064; Taipale *et al*, 2012).

A Gateway entry vector, pDONR221‐TCF7L2, was generated by BP recombination cloning of the TCF7L2 CDS from TCF7L2 pLX307 in to the pDONR221 vector (Invitrogen). Entry vectors were recombined into destination vectors pMH‐Myc and pcDNA3.1‐3xFLAG‐V5‐ccdB, to generate plasmids for transfection, using the LR Clonase II enzyme mix (Invitrogen, Waltham, MA, USA), according to the manufacturer's instructions. Where required, site directed mutagenesis was performed using the QuickChange Lightning Kit (Stratagene, La Jolla, CA, USA) on the entry vectors prior to LR recombination, according to manufacturer's instructions. HEK293T immortalised embryonic kidney cells (RRID:CVCL_0063) were confirmed mycoplasma‐free by regular commercial testing and maintained in DMEM with 10% FBS and 1% penicillin and streptomycin (Sigma Aldrich). Cells for transfection were plated at 5 × 10^6^ cells per 10 cm dish in antibiotic‐free medium. After 24 h, cultures were transfected with 10ug total plasmid using 30 μl FuGENE HD transfection reagent (Promega, Madison, WI, US) according to manufacturer's protocol. Transfection efficiency was confirmed the following day by visualisation of GFP by microscopy, and cells split into 2 × 10 cm plates and allowed to grow for a further 24 h before lysis in 500 μl benzonase lysis buffer (20 mM Tris pH 7.5; 40 mM KCl; 2 mM MgCl2; 10% (v/v) glycerol, 0.5% (v/v) IGEPAL‐CA‐630; 50 U/ml Benzonase (25 U/μl); 1× cOmplete EDTA‐free protease inhibitor (Roche); 0.5× phosphatase inhibitor cocktail 2 (Sigma‐Aldrich); 0.5× phosphatase inhibitor cocktail 3 (Sigma‐Aldrich)) on ice for 10 min. KCl was then added to 450 mM final concentration and samples rotated for 30 min at 4°C before clarification and protein quantification by CB‐X assay (G‐Biosciences St Louis, MO, USA). Clarified lysates were diluted to KCl concentration of 150 mM by addition of No‐salt equilibration buffer” (20 mM Tris–HCl pH 7.5; 10% (v/v) glycerol; 0.5 mM dithiothreitol (DTT); 0.5 mM ethylenediaminetetraacetic acid (EDTA); 1× cOmplete EDTA‐free protease inhibitor; 0.5× phosphatase inhibitor cocktail 2; 0.5× phosphatase inhibitor cocktail 3). 2 mg of protein was pre‐cleared by incubation with mouse IgG1 isotype control magnetic beads followed by anti‐FLAG M2 magnetic beads for 2 h. Bead‐substrate complexes were washed five times in wash buffer (20 mM Tris–HCl (pH 7.5); 100 mM KCl; 10% (v/v) glycerol; 0.5 mM DTT; 1X cOmplete EDTA‐free protease inhibitor; 0.5x phosphatase inhibitor cocktail 2; 0.5x phosphatase inhibitor cocktail 3) at 4°C and resuspended in 30 μl LDS elution buffer (50 mM glycine pH 2.8, 1× NuPAGE LDS Sample Buffer, 1× NuPAGE Sample Reducing Agent) before heating at 70°C for 10 min. The supernatant was retained and loaded onto 4–12% Bolt Bis‐Tris mini‐protein gels (Invitrogen) for electrophoretic separation and transfer to PVDF membrane before immunoblotting as described above. Primary and secondary antibodies used are listed in Dataset EV10.

### Statistical analysis

Sample sizes were not predetermined and animals were not randomised. With the exception of pathological grading, researchers were not blinded to experimental genotype. Data were analysed using GraphPad Prism (GraphPad, San Diego, CA, USA) or R (R Core Team, 2022). Statistical tests used for comparison of groups are indicated in the text or figure legends in all cases. Survival curves were generated according to the Kaplan–Meier method and groups compared by the log‐rank test. Testing for Fbxw7*Trp53 interaction in survival analysis was performed by analysis of the cross‐product term in a Cox proportional hazards model including mutation status of both genes (*Fbxw7* ^+/+^ or *Fbxw7* ^R482Q/+^ and *Trp53* ^del/del^ or *Trp53* ^R172H/del^). All statistical tests were two‐sided. Statistical significance was accepted at *P* < 0.05 or FDR < 0.05 depending on the analysis.

## Author contributions

**Matthew Brown:** Conceptualization; resources; data curation; formal analysis; validation; investigation; visualization; methodology; project administration; writing – review and editing. **Alicia Leon:** Formal analysis; investigation; writing – review and editing. **Katarzyna Kedzierska:** Formal analysis; investigation; writing – review and editing. **Charlotte Moore:** Data curation; investigation; writing – review and editing. **Hayley L Belnoue‐Davis:** Resources; writing – review and editing. **Susanne Flach:** Investigation; writing – review and editing. **John P Lydon:** Resources; writing – review and editing. **Francesco J DeMayo:** Resources; writing – review and editing. **Annabelle Lewis:** Resources; writing – review and editing. **Tjalling Bosse:** Formal analysis; supervision; validation; writing – review and editing. **Ian Tomlinson:** Resources; supervision; funding acquisition; writing – review and editing. **David N Church:** Conceptualization; resources; data curation; formal analysis; supervision; funding acquisition; validation; investigation; visualization; methodology; writing – original draft; project administration; writing – review and editing.

## Disclosure and competing interests statement

The authors declare that they have no conflict of interest.

## For more information

Author website: https://www.well.ox.ac.uk/research/research‐groups/church‐group.
Endometrial cancer driver mutations: https://www.intogen.org/search?cancer=UCEC.
Endometrial cancer information and patient support: https://peachestrust.org.

## Supporting information



Appendix

Dataset EV1

Dataset EV2

Dataset EV3

Dataset EV4

Dataset EV5

Dataset EV6

Dataset EV7

Dataset EV8

Dataset EV9

Dataset EV10

Dataset EV11

Source Data for Appendix

Source Data for Figure 1

Source Data for Figure 2

Source Data for Figure 3

Source Data for Figure 4

Source Data for Figure 5

Source Data for Figure 6

## Data Availability

Microarray data from this study have been deposited in GEO under accession number GSE232356: https://www.ncbi.nlm.nih.gov/geo/query/acc.cgi?acc=GSE232356.
