# Supplementary material for: Functional analysis reveals driver cooperativity and novel mechanisms in endometrial carcinogenesis
Source: EMBO Mol Med. 2023 Aug 17;15(10):e17094. doi: 10.15252/emmm.202217094 (PMC10565641; doi:10.15252/emmm.202217094)
Supplement: Supplementary file 1 — Appendix [file EMMM-15-e17094-s020.pdf]

Supplementary figures to Brown et al.

Functional analysis in mice reveals driver cooperativity and novel mechanisms in endometrial carcinogenesis

List of supplementary figures

Appendix Figure. S1. Comparison of missense to truncating *FBXW7* driver mutation frequency across cancer types

Appendix Figure S2. PCR showing targeted alleles and uterine recombination

Appendix Figure S3. Rapid development of carcinoma with uterine Pten deletion

Appendix Figure S4. Foxa2 downregulation in GEMM endometrial carcinomas

Appendix Figure S5. Abnormal p53 staining in endometrial tumours

Appendix Figure S6. Volcano plot showing differentially expressed genes (DEGs) between wild-type and *Fbxw7*^{R482Q} mutant uteri at 8 weeks age

Appendix Figure S7. Volcano plot showing differentially expressed genes (DEGs) between *Pten*^{del} and *Pten*^{del}, *Fbxw7*^{R482Q} mutant uteri at 8 weeks age

Appendix Figure S8. Upregulation of Wnt target genes in *Pten*^{del}, *Fbxw7*^{mut} uteri

Appendix Figure S9. T cell infiltrate in endometrial cancers from *Pten*^{del} and *Pten*^{del}; *Fbxw7*^{mut} mice

Appendix Figure S10. Enrichment analysis of FBXW7 deleted isogenic colorectal cell lines

Appendix Figure S11. Epithelial LEF1 expression in high-grade human endometrial cancers of no specific molecular profile (NSMP) and p53-abnormal molecular subgroups according to *FBXW7* mutation status

Appendix Figure S12. Epithelial LEF1 expression in endometrial cancers from GEMM at experimental endpoint

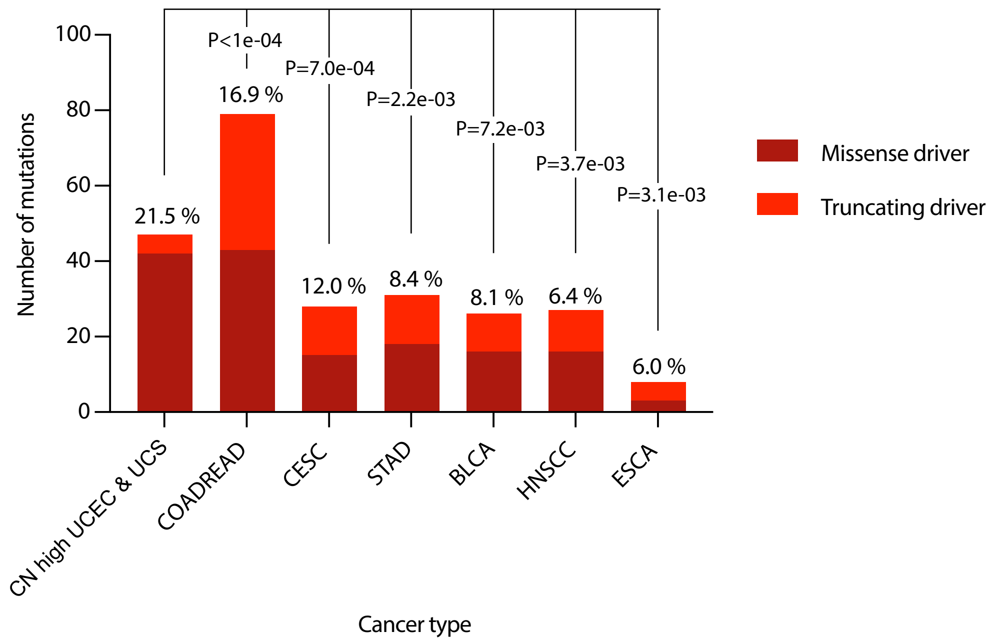

Appendix Figure. S1. Comparison of missense to truncating *FBXW7* driver mutation frequency across cancer types

Absolute number of missense and truncating (nonsense, frameshift, splice site) *FBXW7* driver mutations are shown for high-risk, copy-no (CN) high endometrial and carcinosarcoma (UCEC & UCS), colon and rectal cancer (COADREAD), cervical squamous cell carcinoma (CESC), stomach adenocarcinoma (STAD), bladder carcinoma (BLCA), head and neck squamous cell carcinoma (HNSCC) and oesophageal adenocarcinoma (ESCA) from the TCGA study. Cancer types with *FBXW7* mutation frequency >5% were selected for comparison – mutation frequency (also includes non-driver mutations) for each is shown above bars. *P* values show results of comparison of individual cancer types with CN-high endometrial and carcinosarcomas by Fisher exact test.

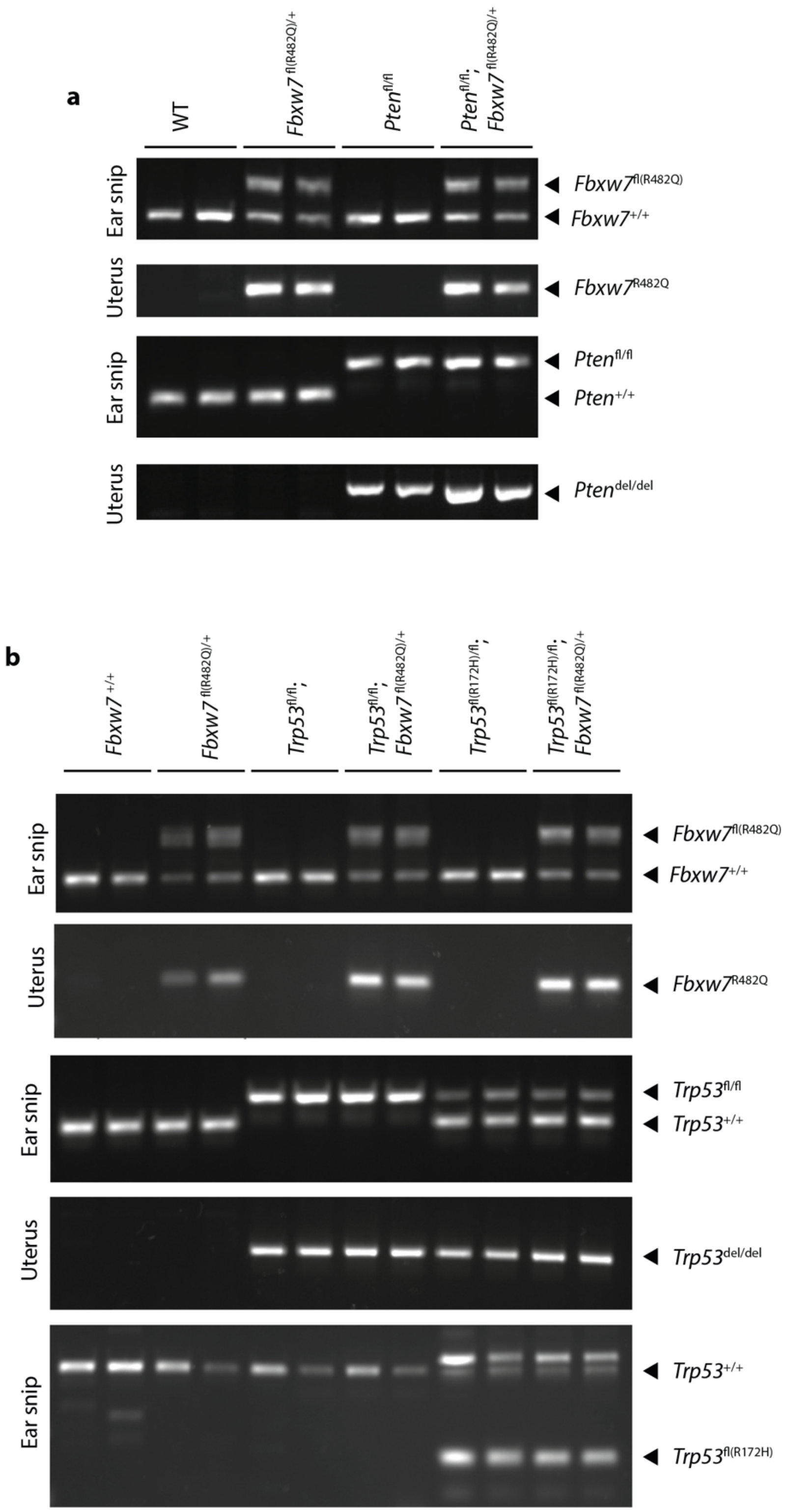

Appendix Figure S2. PCR showing targeted alleles and uterine recombination

DNA extracted from ear snips and uteri from mice of indicated genotypes carrying conditional *Fbxw7* and *Pten* alleles (**a**) and carrying conditional *Fbxw7* and *Trp53* alleles (**b**) was amplified by PCR using primer pairs (Methods) specific for *Fbxw7*$^{fl(R482Q)}$, *Pten*^{fl}, *Trp53*^{fl} and *Trp5*$^{fl(R172H)}$ conditional alleles and recombined *Fbxw7*^{R482Q} allele (primers for recombined *Trp53*^{R172H} allele are not available).

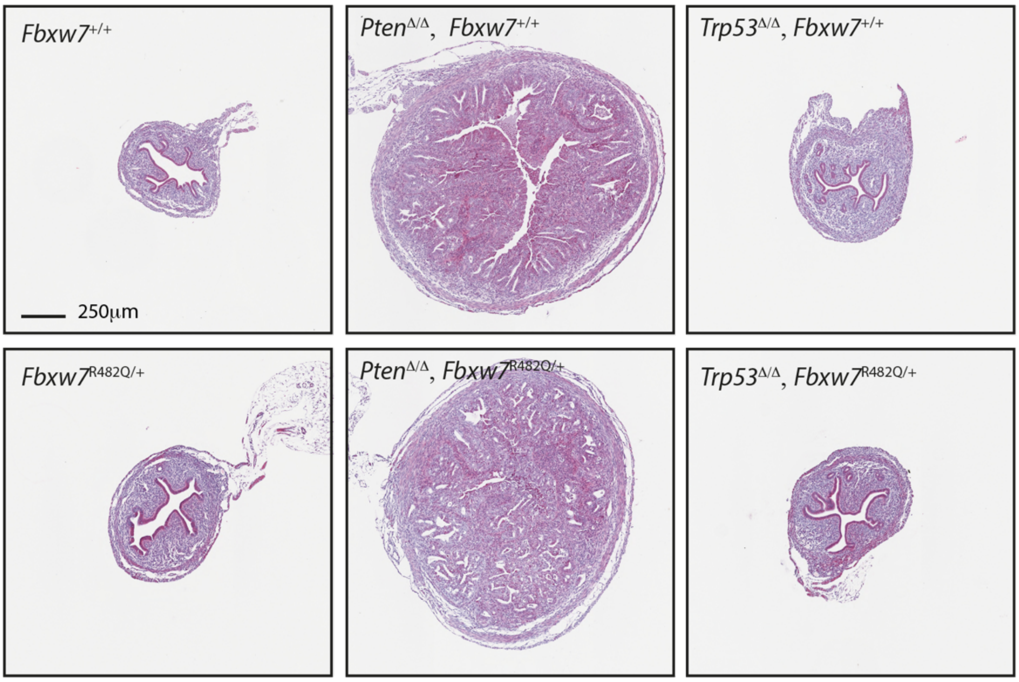

Appendix Figure S3. Rapid development of carcinoma with uterine Pten deletion
Representative haematoxylin and eosin (H&E) stained sections of uteri from mice of indicated genotypes at four weeks post-natal age. *Tp53*$^{R172/\Delta}$ animals were not analysed at this timepoint.

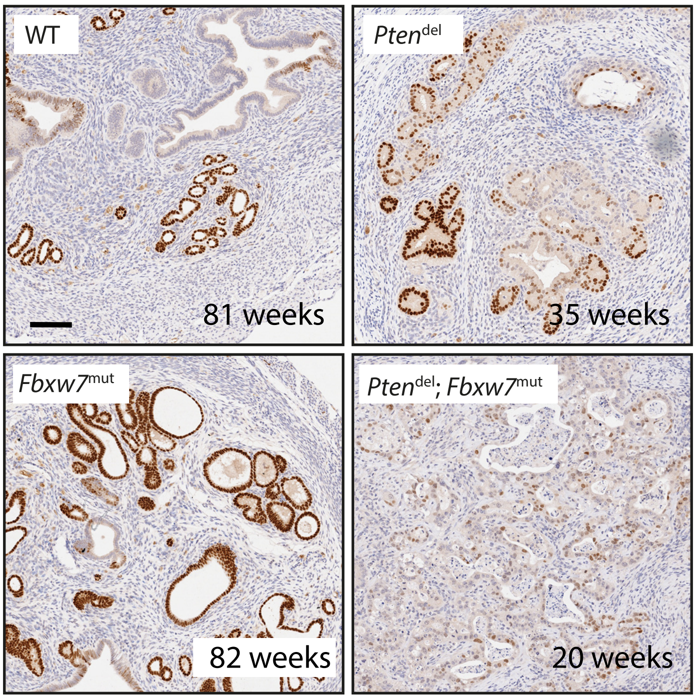

Appendix Figure S4. Foxa2 downregulation in GEMM endometrial carcinomas
Representative images of Foxa2 immunohistochemistry on endometria from mice of indicated genotypes aged for survival analysis. Scale bar indicates 100um.

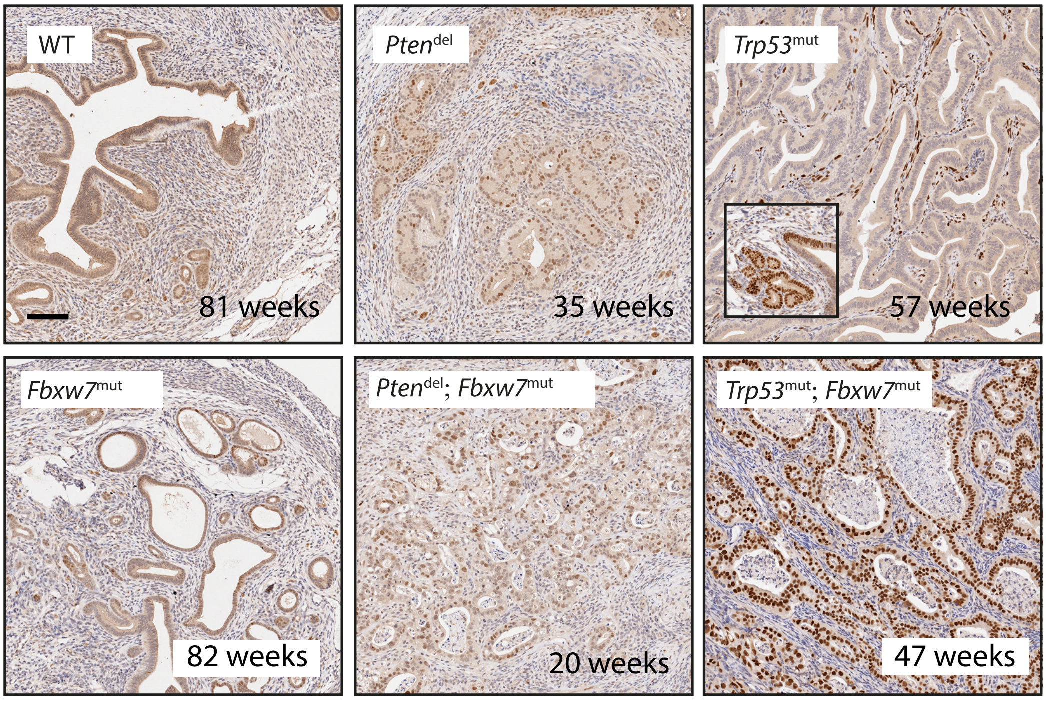

Appendix Figure S5. Abnormal p53 staining in endometrial tumours

p53 immunostaining on endometria from mice of indicated genotypes aged for survival analysis. Inset in *Trp53*^{mut} tumour in upper right image shows area with focal abnormal p53 staining in region of morphologically normal uterus from same mouse. Scale bar indicates 100um.

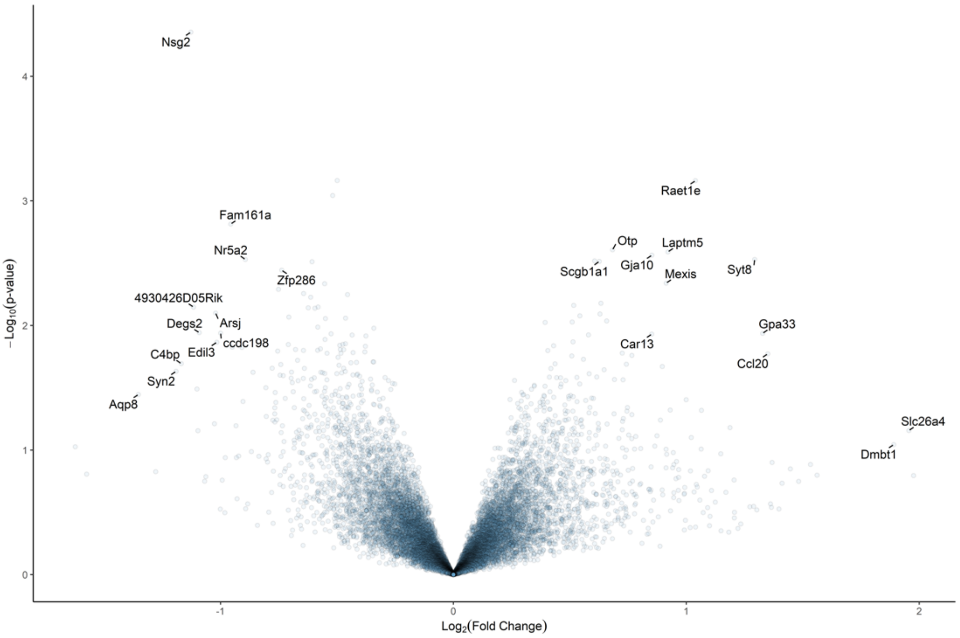

Appendix Figure S6. Volcano plot showing differentially expressed genes (DEGs) between wild-type and *Fbxw7*^{R482Q} mutant uteri at 8 weeks age

DEG analysis was performed following gene expression profiling of wild-type and *Fbxw7*^{R482Q}-mutant uteri (n=7 both groups).

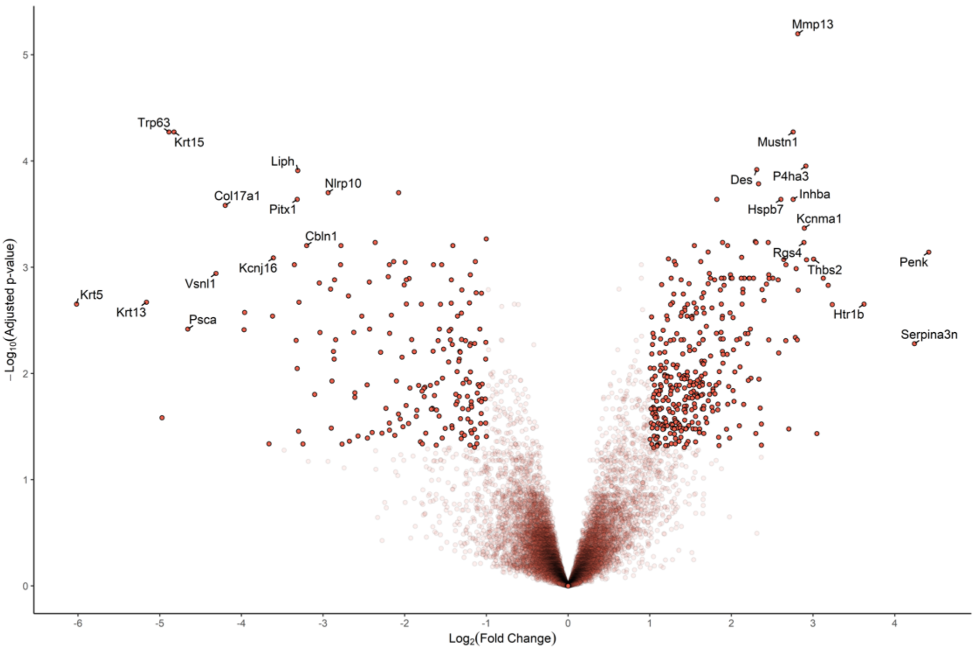

Appendix Figure S7. Volcano plot showing differentially expressed genes (DEGs) between *Pten*^{del} and *Pten*^{del}, *Fbxw7*^{R482Q} mutant uteri at 8 weeks age

DEG analysis was performed following gene expression profiling of *Pten*^{del} and *Pten*^{del}, *Fbxw7*^{R482Q} mutant uteri (n=7 both groups).

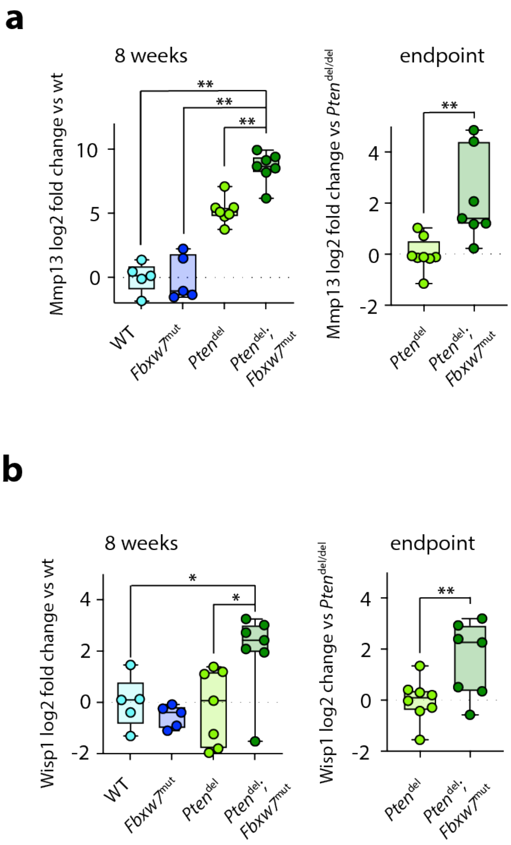

Appendix Figure S8. Upregulation of Wnt target genes in *Pten*^{del}, *Fbxw7*^{mut} uteri

RT-qPCR analysis showing expression of LEF1 target genes Mmp13 (**a**) and Wisp1 (**b**) in uteri from mice of indicated genotypes at 8 weeks age and at experimental endpoint.

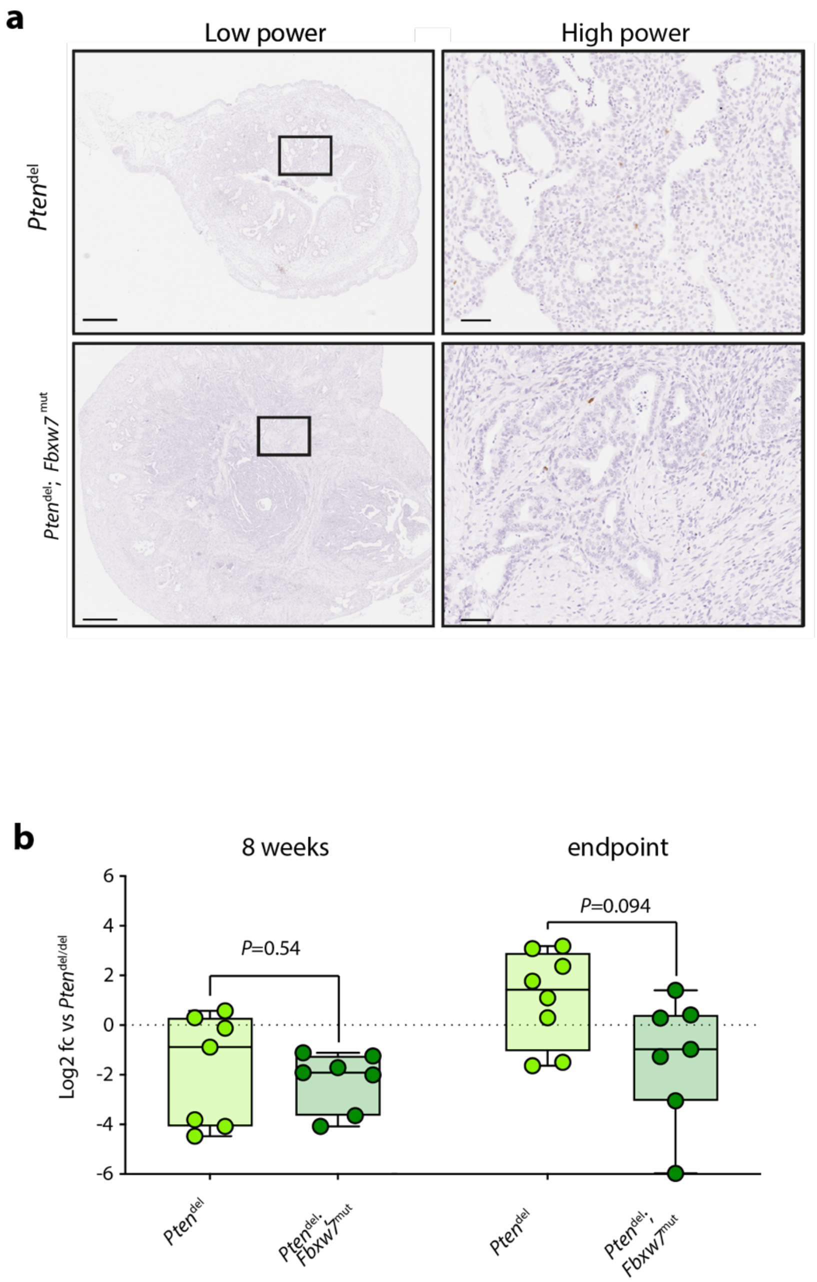

Appendix Figure S9. T cell infiltrate in endometrial cancers from *Pten*^{del} and *Pten*^{del}; *Fbxw7*^{mut} mice

a. Representative low and high magnification images of CD8 immunohistochemistry from uteri of mice of indicated genotypes at 8 weeks postnatal age. Scale bars in left panels indicate 500um, those in right panels indicate 60um. **b.** RT-qPCR analysis for Cd3e expression in uteri from mice of indicated genotypes at 8 weeks postnatal age, and at experimental endpoint. Results are shown as fold change relative to all samples from *Pten*^{del} mice. Horizontal line within boxes indicates sample median, upper and lower box borders correspond to 1.5x above and below the interquartile range, whiskers indicate minimum and maximum values. Statistical comparison between groups was performed by Mann-Whitney test.

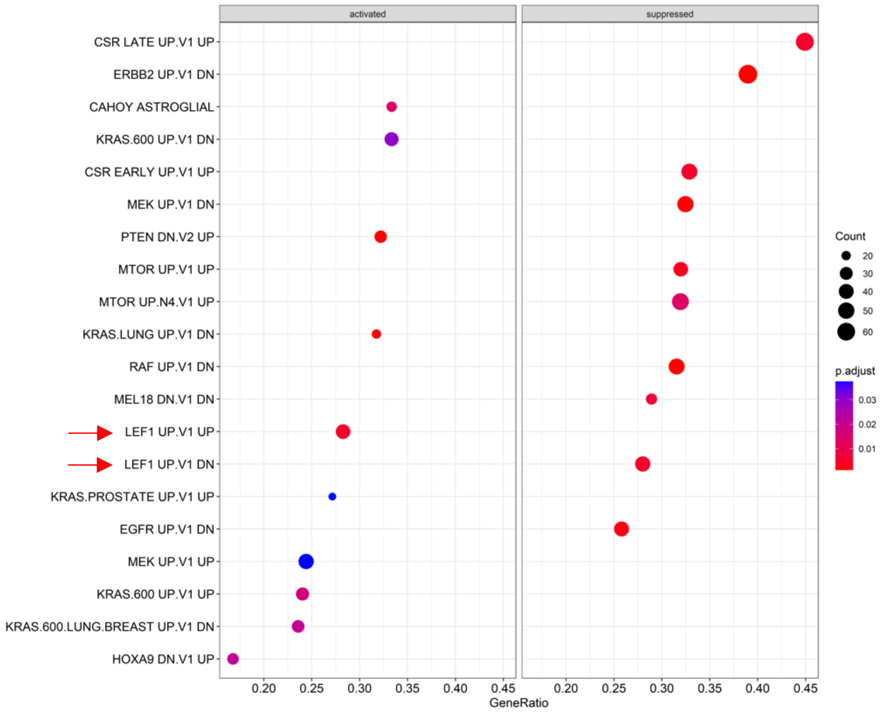

Appendix Figure S10. Enrichment analysis of FBXW7 deleted isogenic colorectal cell lines
Dot plot indicating enrichment of Gene Ontology (GO) biological processes (BP) in *FBXW*$^{del/del}$ vs *FBXW7*$^{+/+}$ HCT116 cells generated by Thirimanne and colleagues (Ref 29).

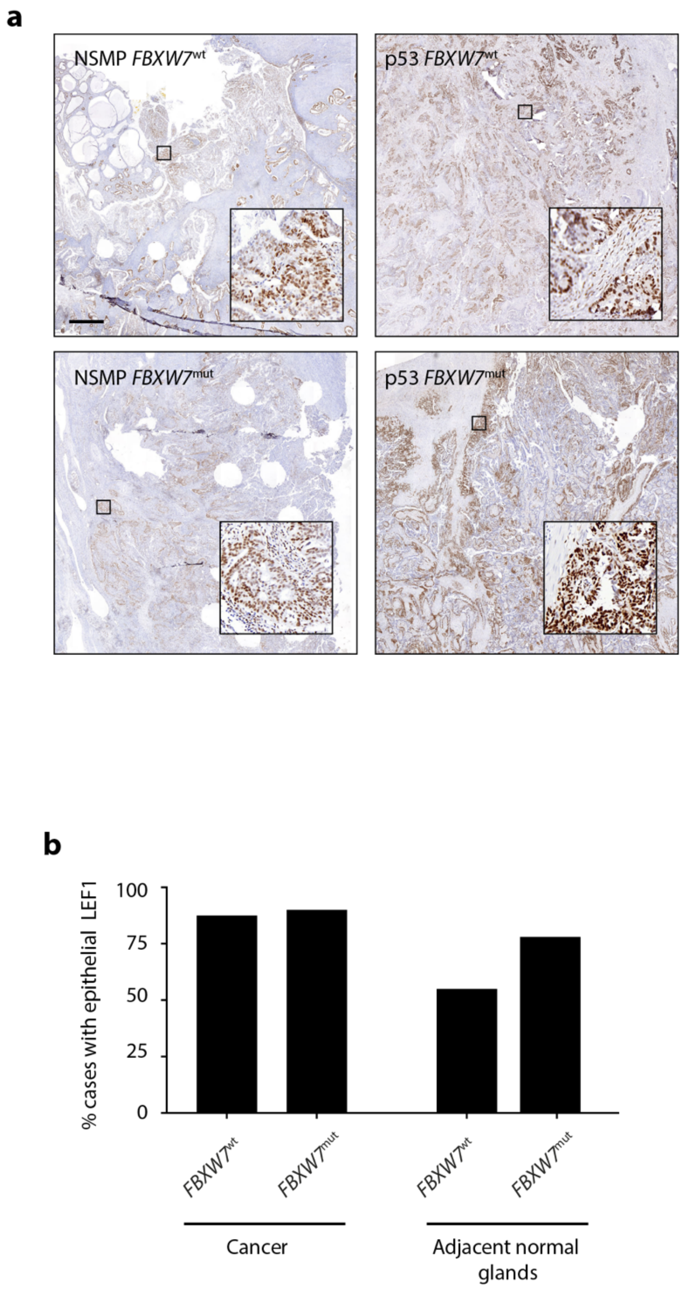

Appendix Figure S11. Epithelial LEF1 expression in high-grade human endometrial cancers of no specific molecular profile (NSMP) and p53-abnormal molecular subgroups according to *FBXW7* mutation status

a. Representative images of LEF1 immunohistochemistry on grade 3 human endometrial cancers of indicated molecular subgroups according to *FBXW7* mutation status. Scale bar indicates 1mm **b.** Proportion of tumours with epithelial LEF1 expression in malignant and adjacent normal epithelium according to *FBXW7* mutation status.

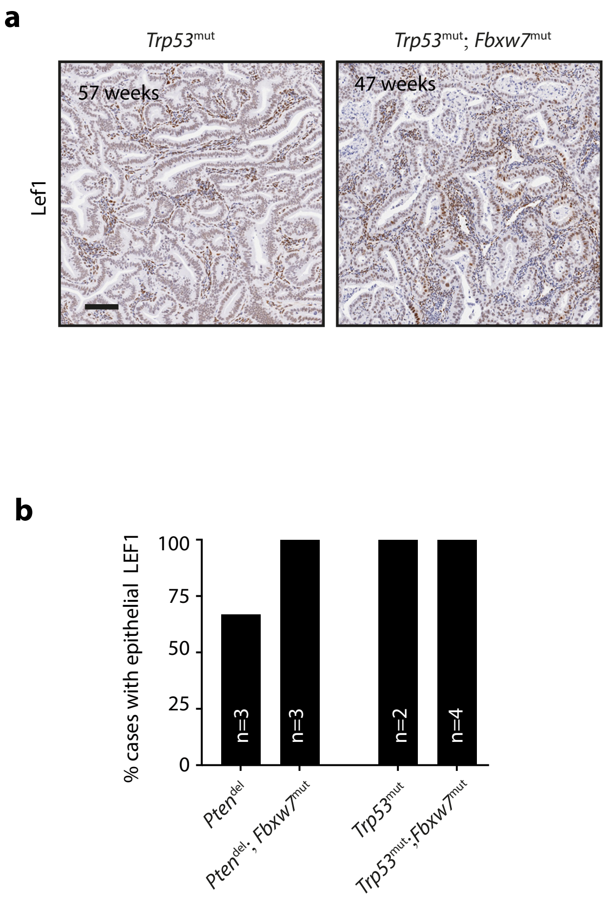

Appendix Figure S12. Epithelial LEF1 expression in endometrial cancers from GEMM at experimental endpoint

a. LEF1 immunohistochemistry on endometrial cancers from GEMM of indicated genotypes at experimental endpoint **b.** Prevalence of epithelial LEF1 expression in endometrial tumours from mice of indicated genotypes at experimental endpoint (6-60 weeks age).
