# Supplementary figures and images for: Functional analysis reveals driver cooperativity and novel mechanisms in endometrial carcinogenesis (part 1 of 2)
Source: EMBO Mol Med. 2023 Aug 17;15(10):e17094. doi: 10.15252/emmm.202217094 (PMC10565641; doi:10.15252/emmm.202217094)

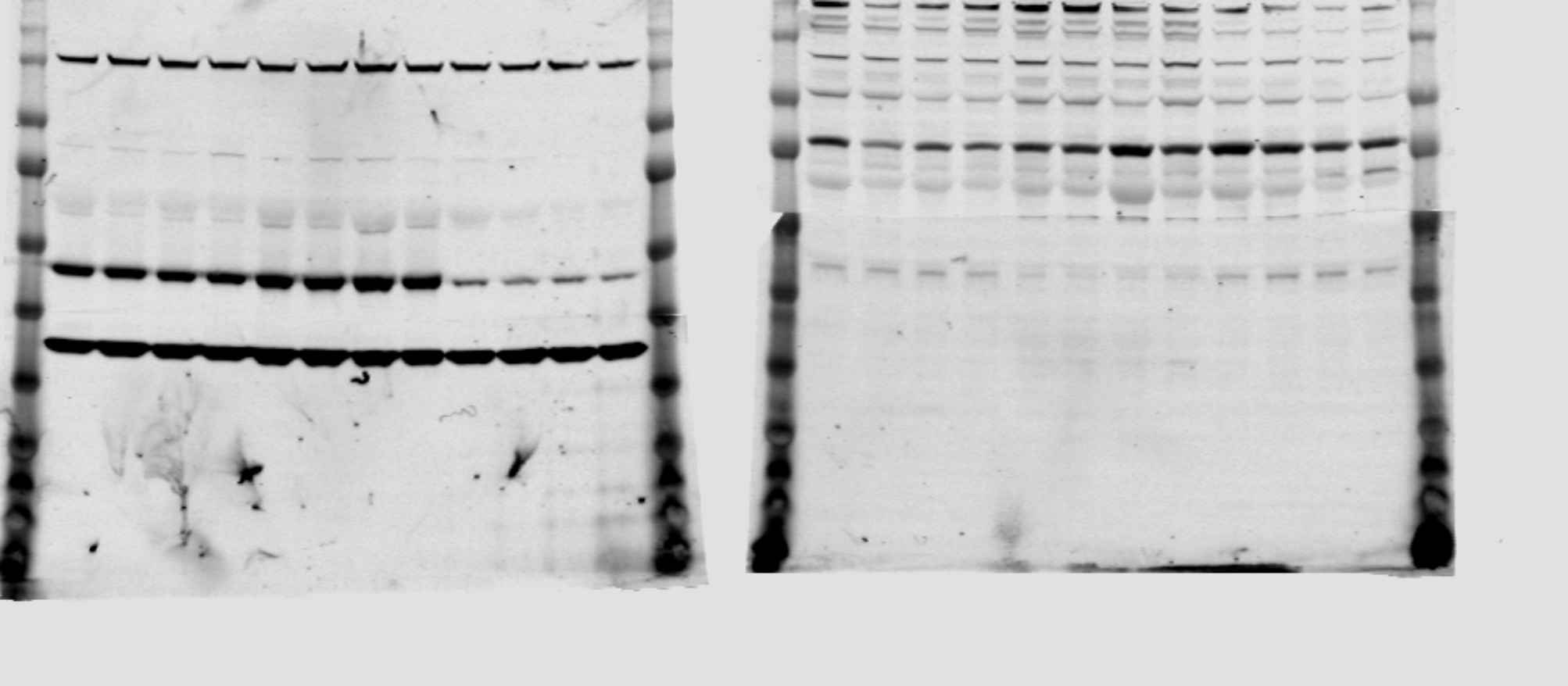

Supplement: Supplementary file 14 — Source Data for Figure 1 [file EMMM-15-e17094-s008.zip › EMM-2022-17094_source_data_figure_1/figure_1E/1E FBXW7.tif]

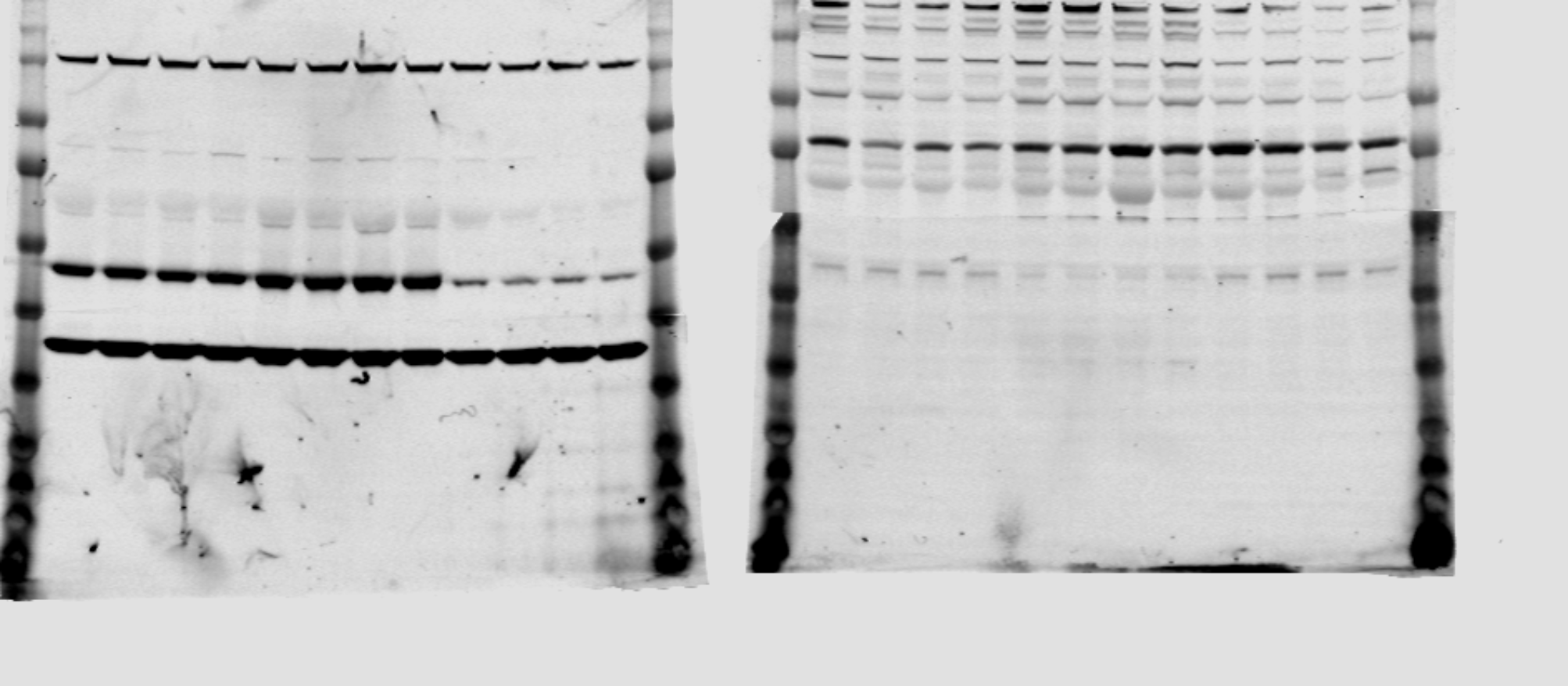

Supplement: Supplementary file 14 — Source Data for Figure 1 [file EMMM-15-e17094-s008.zip › EMM-2022-17094_source_data_figure_1/figure_1E/1E Trp53.tif]

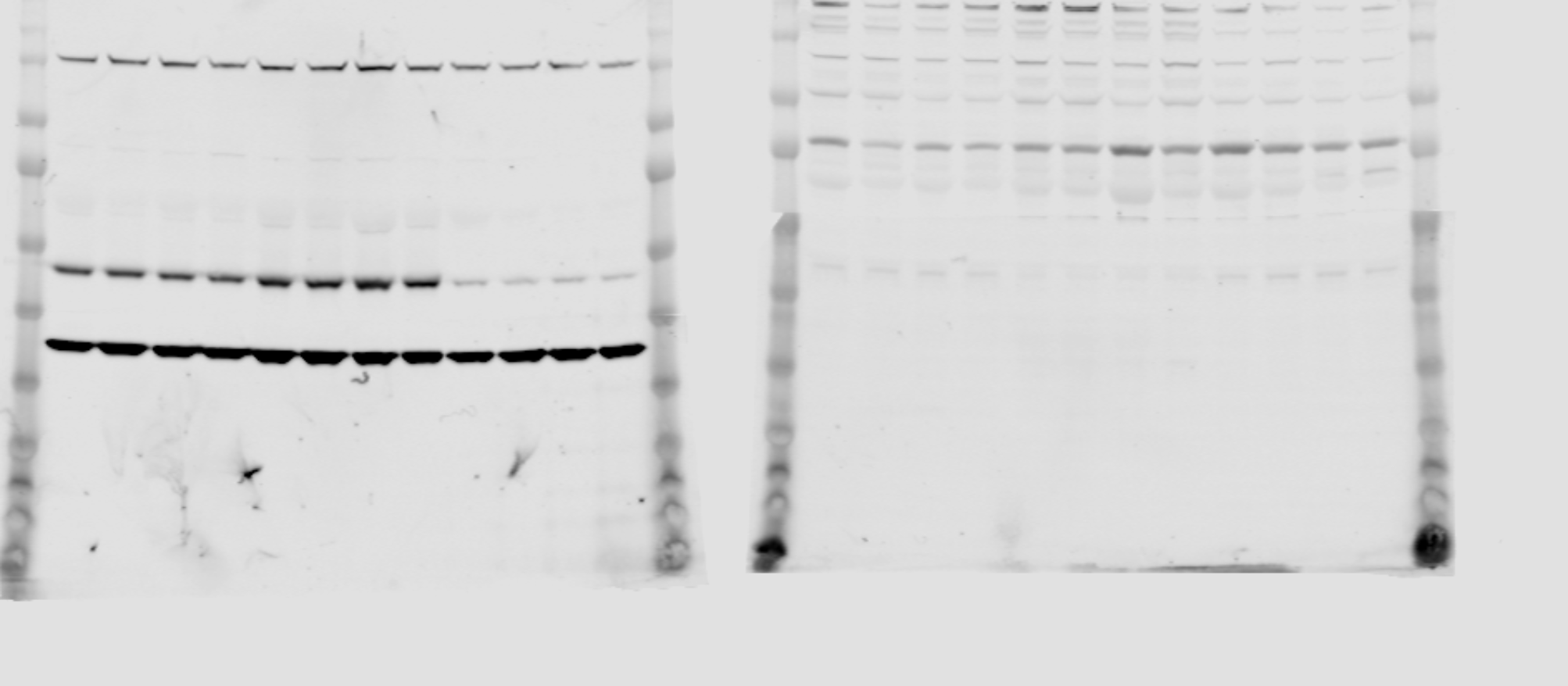

Supplement: Supplementary file 14 — Source Data for Figure 1 [file EMMM-15-e17094-s008.zip › EMM-2022-17094_source_data_figure_1/figure_1E/1E PTEN.tif]

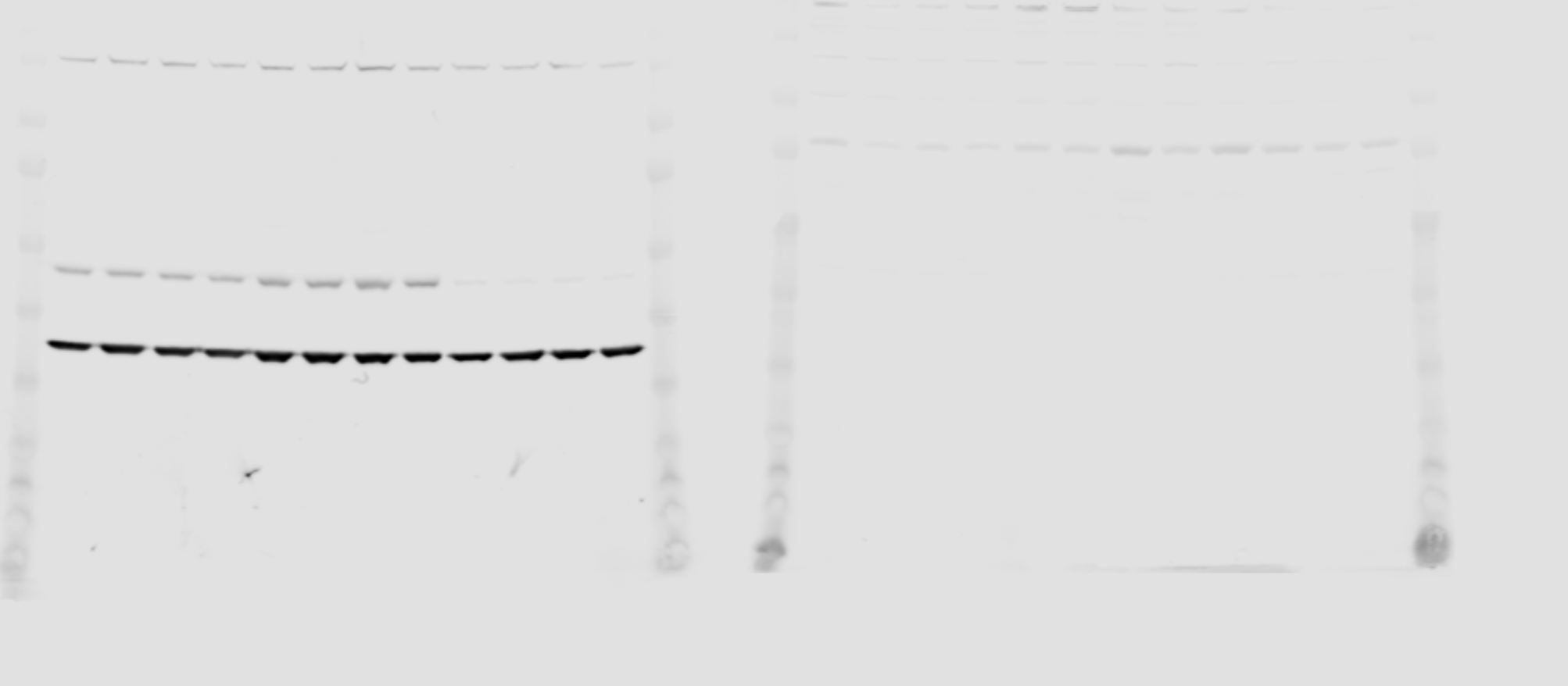

Supplement: Supplementary file 14 — Source Data for Figure 1 [file EMMM-15-e17094-s008.zip › EMM-2022-17094_source_data_figure_1/figure_1E/1E GAPDH.tif]

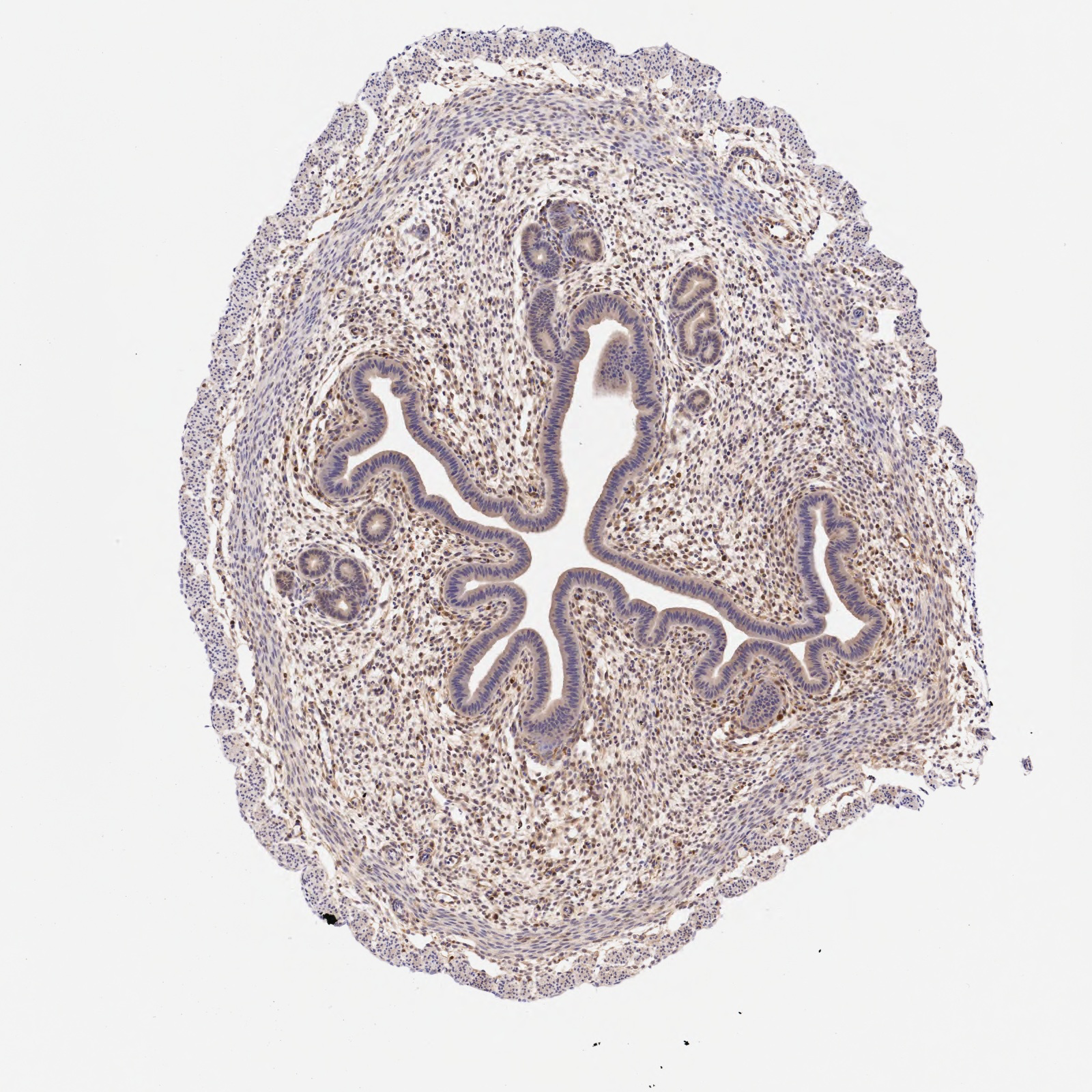

Supplement: Supplementary file 14 — Source Data for Figure 1 [file EMMM-15-e17094-s008.zip › EMM-2022-17094_source_data_figure_1/figure_1F/1F trp53mut_fbxw7mut_tprp_11.3i_pten_10x.jpg]

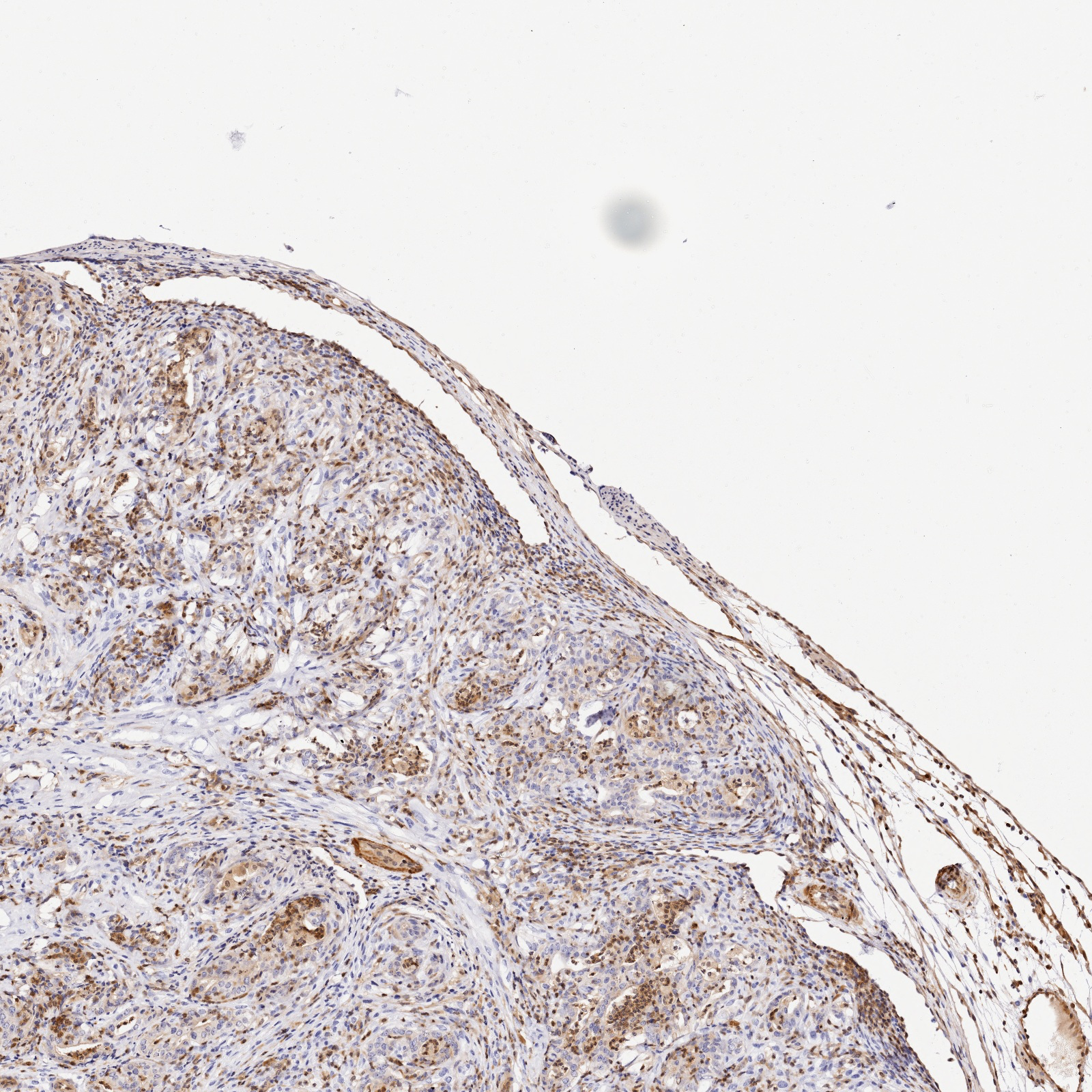

Supplement: Supplementary file 14 — Source Data for Figure 1 [file EMMM-15-e17094-s008.zip › EMM-2022-17094_source_data_figure_1/figure_1F/1F pten_fbxw7mut_tbnw_23_1d_pten_@10x.jpg]

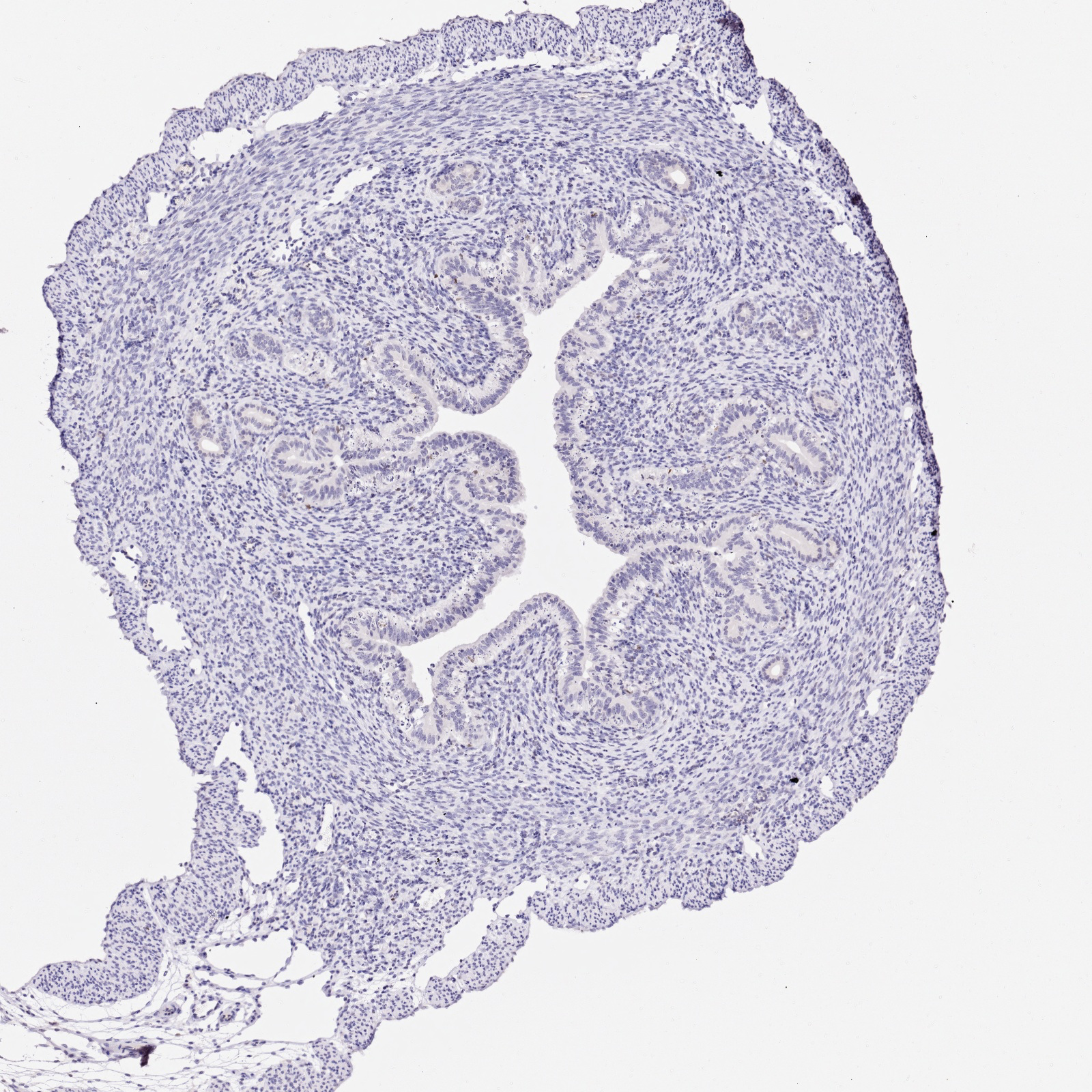

Supplement: Supplementary file 14 — Source Data for Figure 1 [file EMMM-15-e17094-s008.zip › EMM-2022-17094_source_data_figure_1/figure_1F/1F trp53del_fbxw7mut_tbow_13.3f_p53_@10x.jpg]

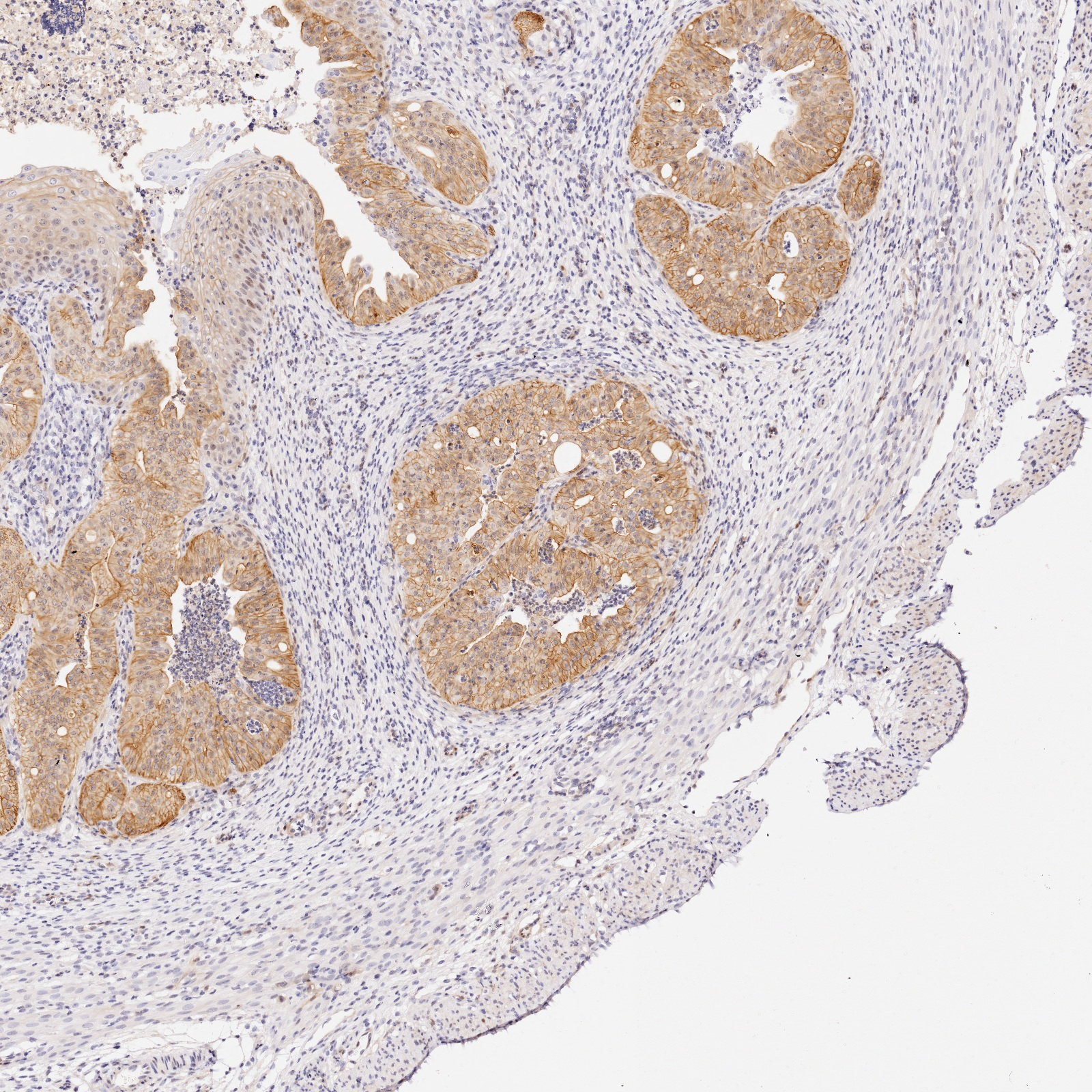

Supplement: Supplementary file 14 — Source Data for Figure 1 [file EMMM-15-e17094-s008.zip › EMM-2022-17094_source_data_figure_1/figure_1F/1F pten_tbnw_15.1f_pAkt_@10x.jpg]

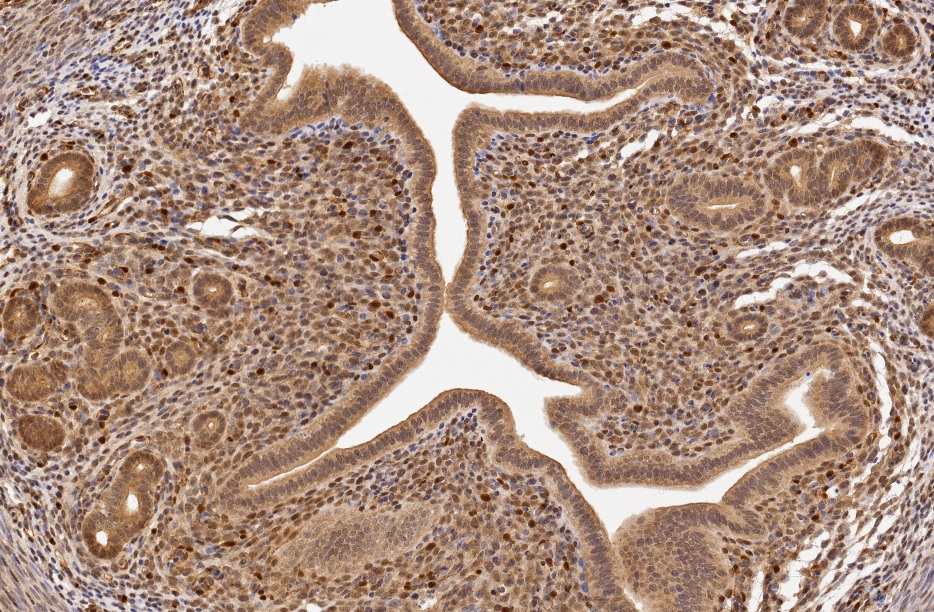

Supplement: Supplementary file 14 — Source Data for Figure 1 [file EMMM-15-e17094-s008.zip › EMM-2022-17094_source_data_figure_1/figure_1F/1F trp53del_tbow_13.3e_pten_@10x.jpg]

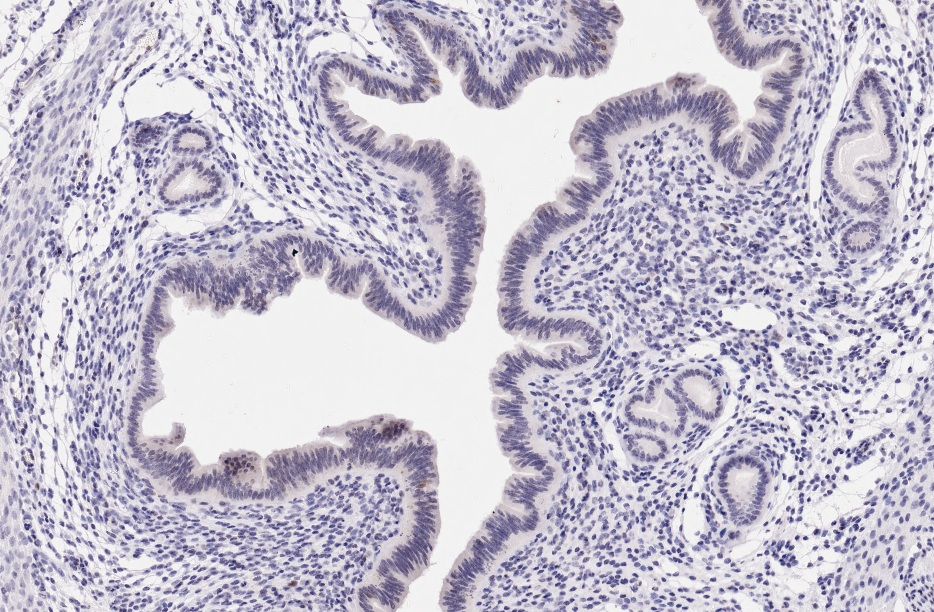

Supplement: Supplementary file 14 — Source Data for Figure 1 [file EMMM-15-e17094-s008.zip › EMM-2022-17094_source_data_figure_1/figure_1F/1F wt_tbpw_8.2f_pAkt_@10x.jpg]

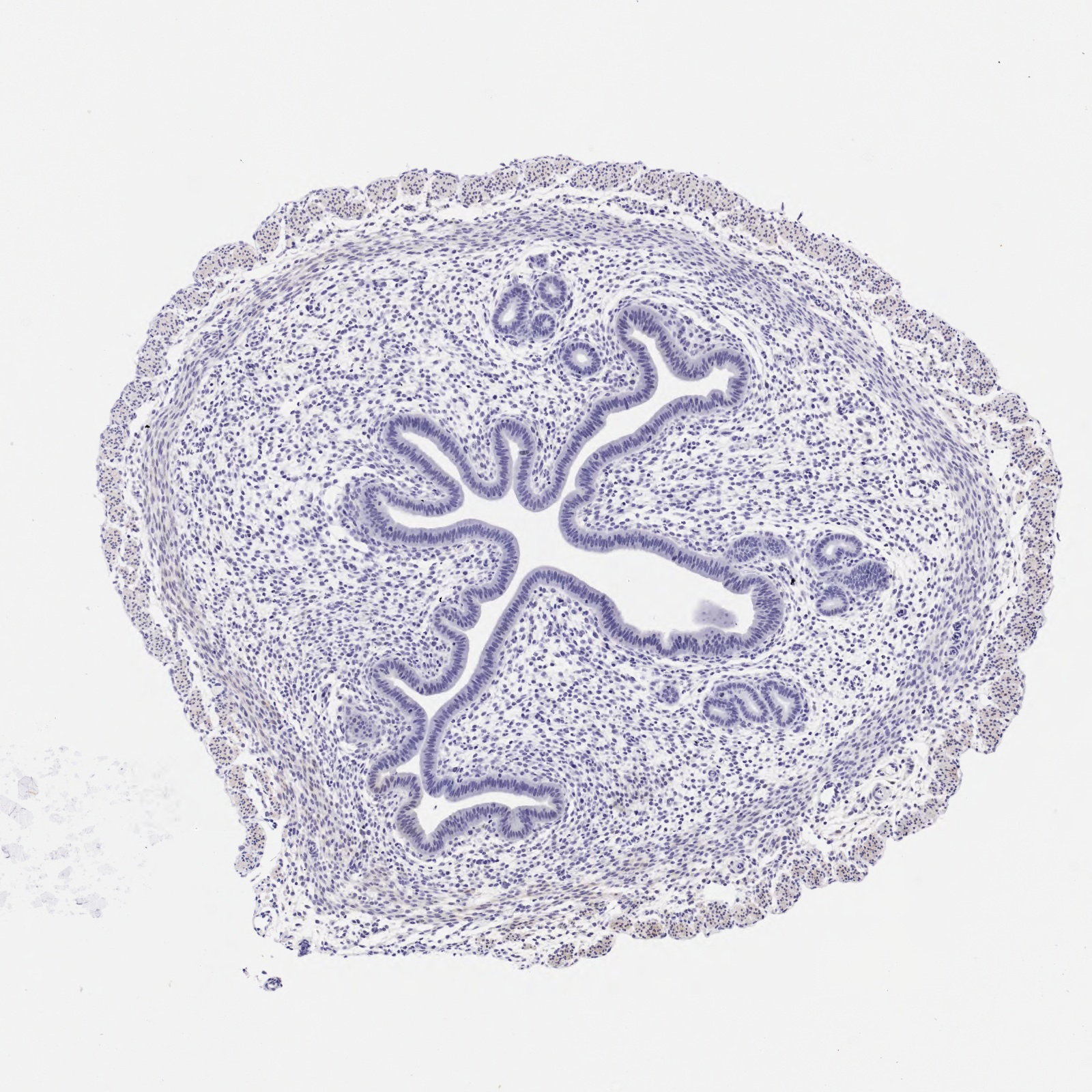

Supplement: Supplementary file 14 — Source Data for Figure 1 [file EMMM-15-e17094-s008.zip › EMM-2022-17094_source_data_figure_1/figure_1F/1F trp53mut_fbxw7mut_tprp_11.3i_pakt_10x.jpg]

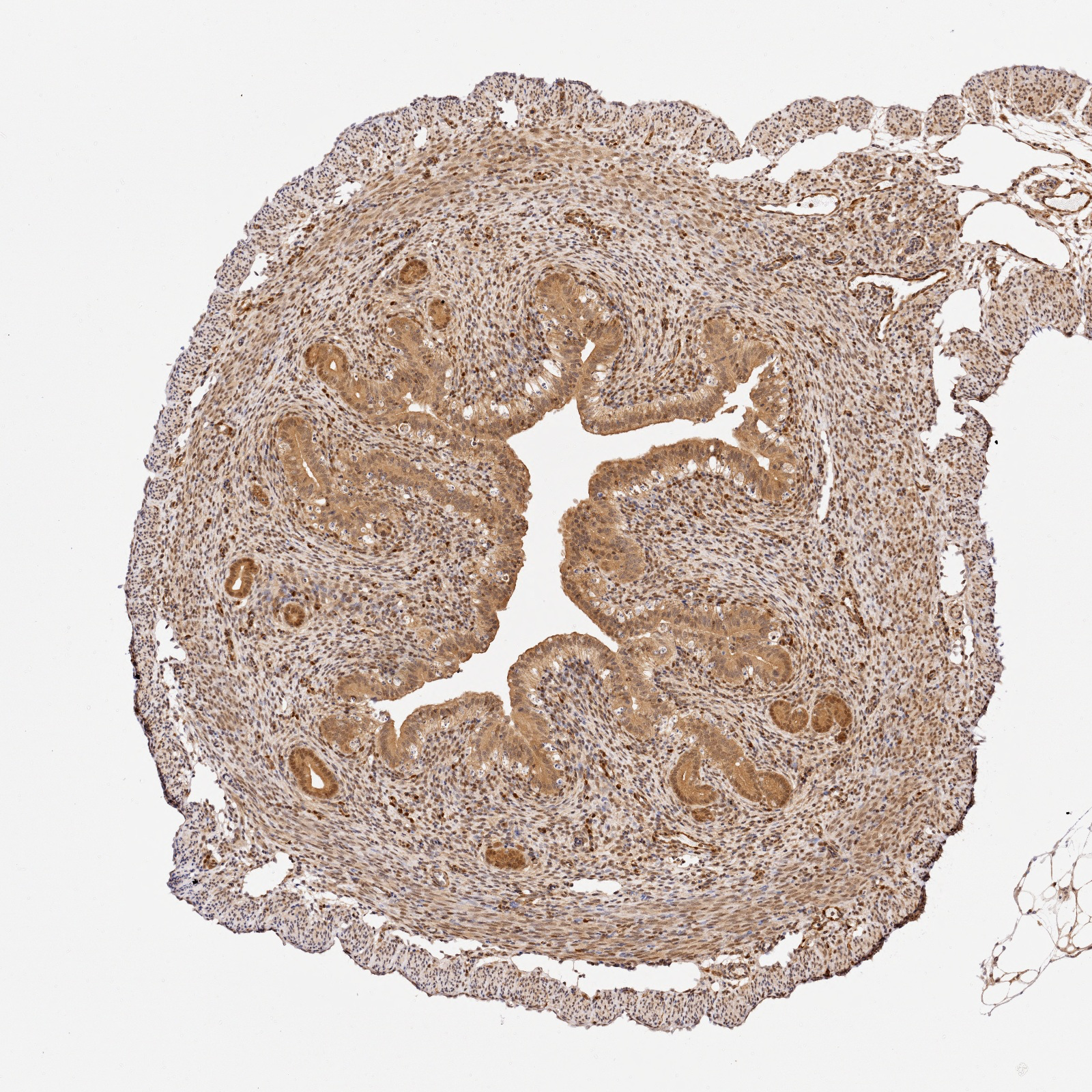

Supplement: Supplementary file 14 — Source Data for Figure 1 [file EMMM-15-e17094-s008.zip › EMM-2022-17094_source_data_figure_1/figure_1F/1F trp53del_fbxw7mut_tbow_13.3f_r482q_pten_@10x.jpg]

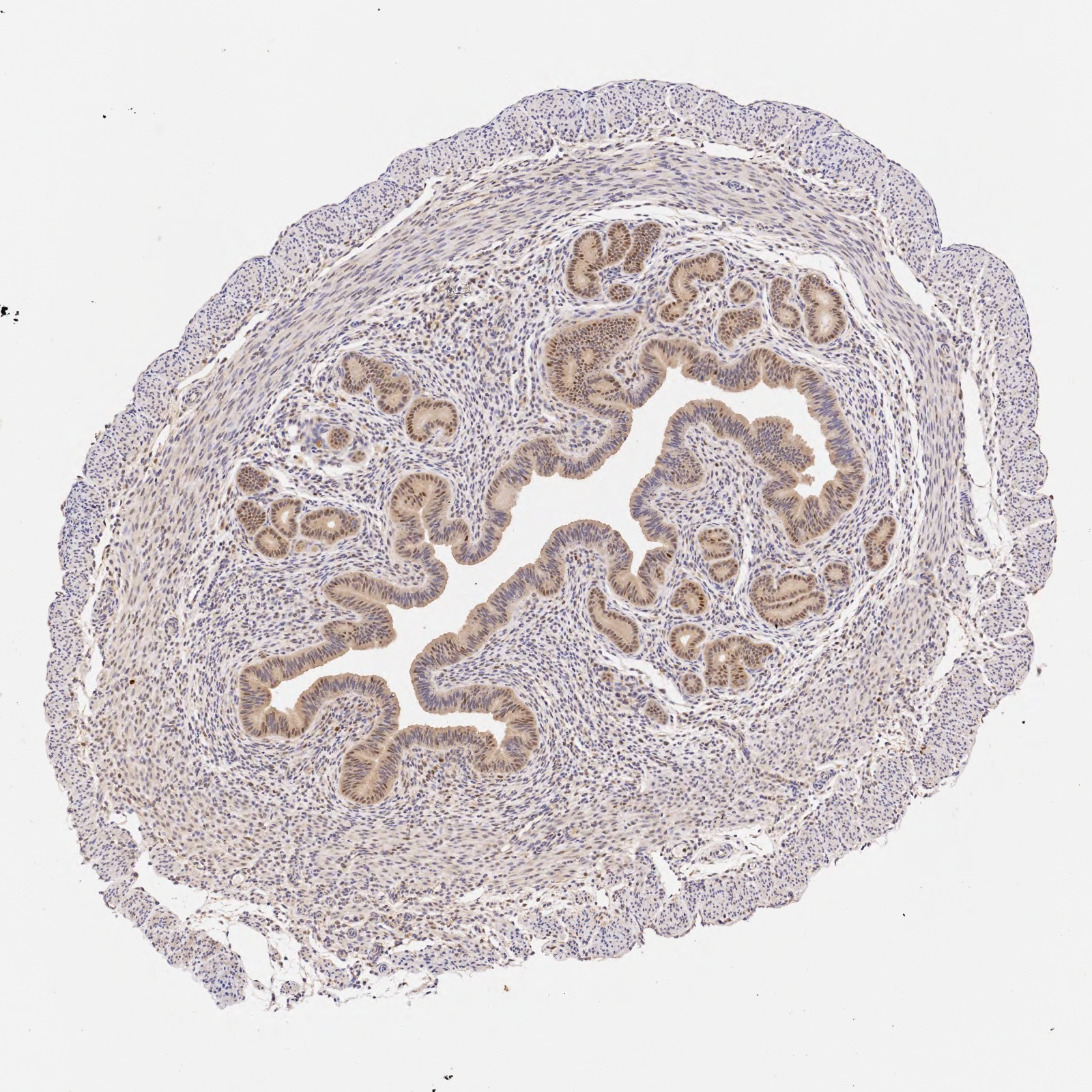

Supplement: Supplementary file 14 — Source Data for Figure 1 [file EMMM-15-e17094-s008.zip › EMM-2022-17094_source_data_figure_1/figure_1F/1F trp53mut_tprp_11.3h_p53_10x.jpg]

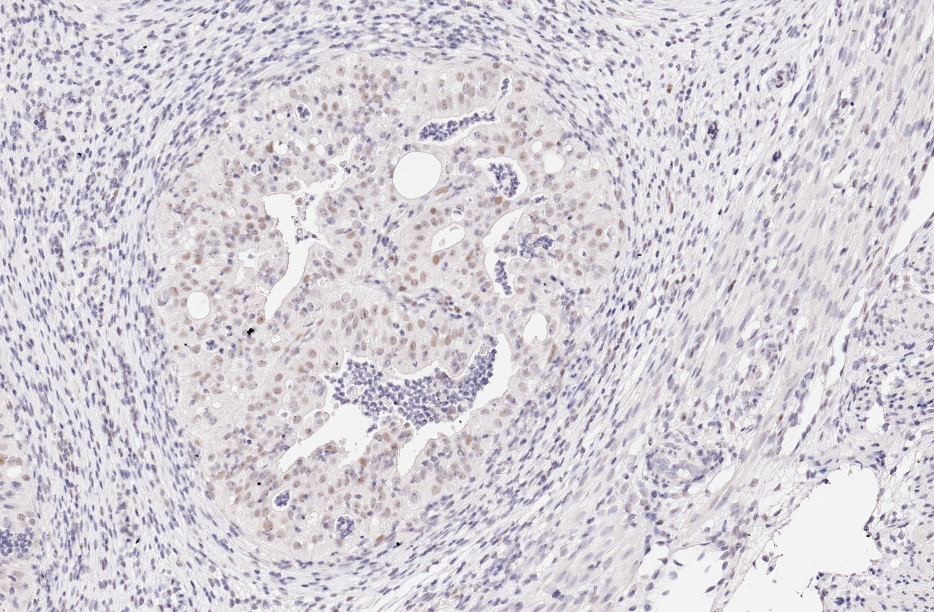

Supplement: Supplementary file 14 — Source Data for Figure 1 [file EMMM-15-e17094-s008.zip › EMM-2022-17094_source_data_figure_1/figure_1F/1F pten_tbnw_15.1f_p53_@10x.jpg]

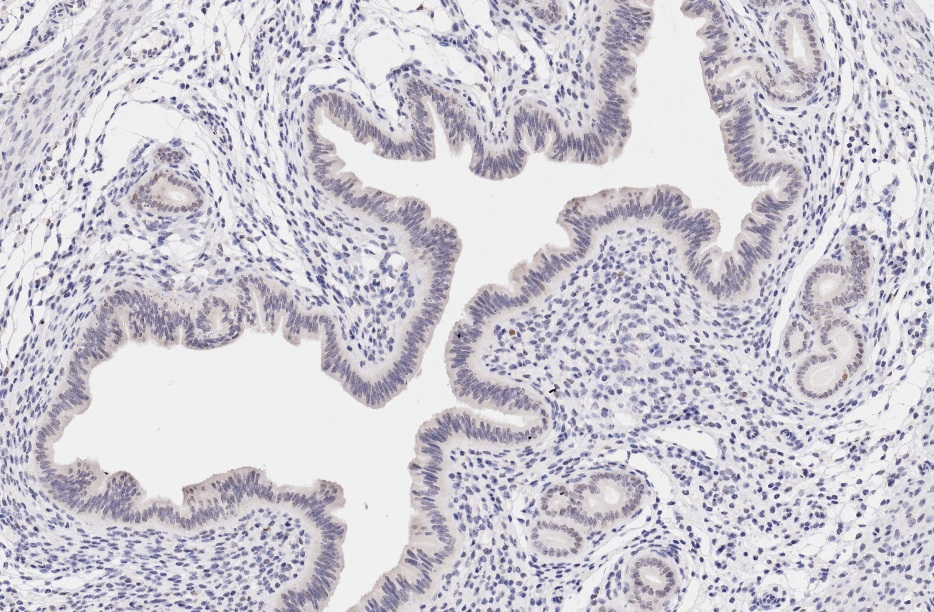

Supplement: Supplementary file 14 — Source Data for Figure 1 [file EMMM-15-e17094-s008.zip › EMM-2022-17094_source_data_figure_1/figure_1F/1F wt_tbpw_8.2f_p53_@10x.jpg]

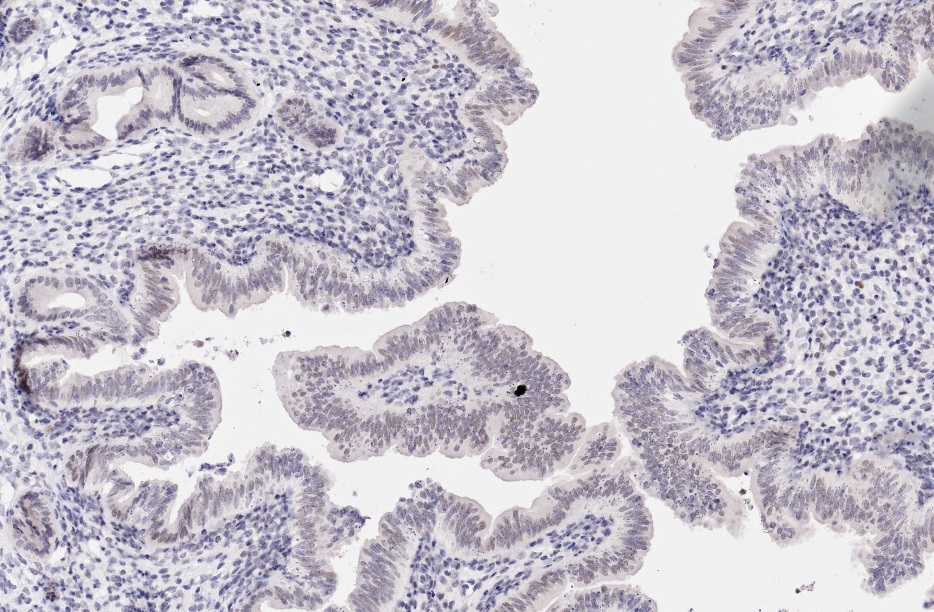

Supplement: Supplementary file 14 — Source Data for Figure 1 [file EMMM-15-e17094-s008.zip › EMM-2022-17094_source_data_figure_1/figure_1F/1F fbxw7mut_tbpw_8.2g_p53_@10x.jpg]

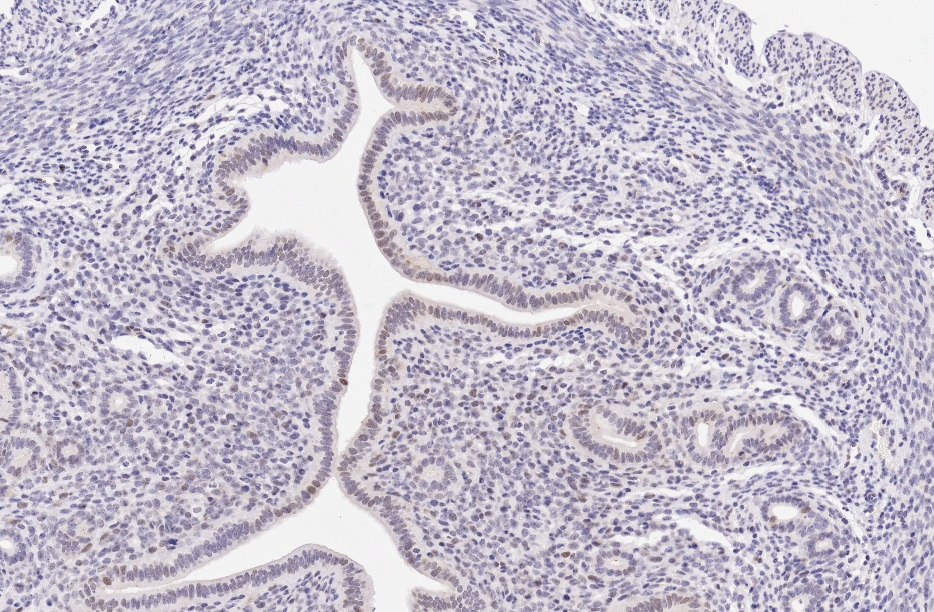

Supplement: Supplementary file 14 — Source Data for Figure 1 [file EMMM-15-e17094-s008.zip › EMM-2022-17094_source_data_figure_1/figure_1F/1F trp53del_tbow_13.3e_pAkt@10x.jpg]

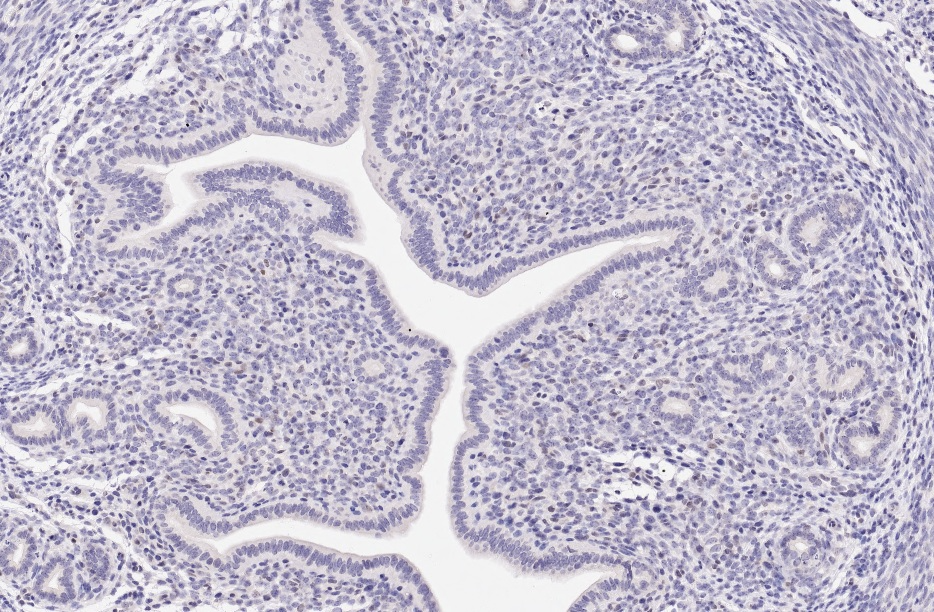

Supplement: Supplementary file 14 — Source Data for Figure 1 [file EMMM-15-e17094-s008.zip › EMM-2022-17094_source_data_figure_1/figure_1F/1F trp53del_tbow_13.3e_p53_@10x.jpg]

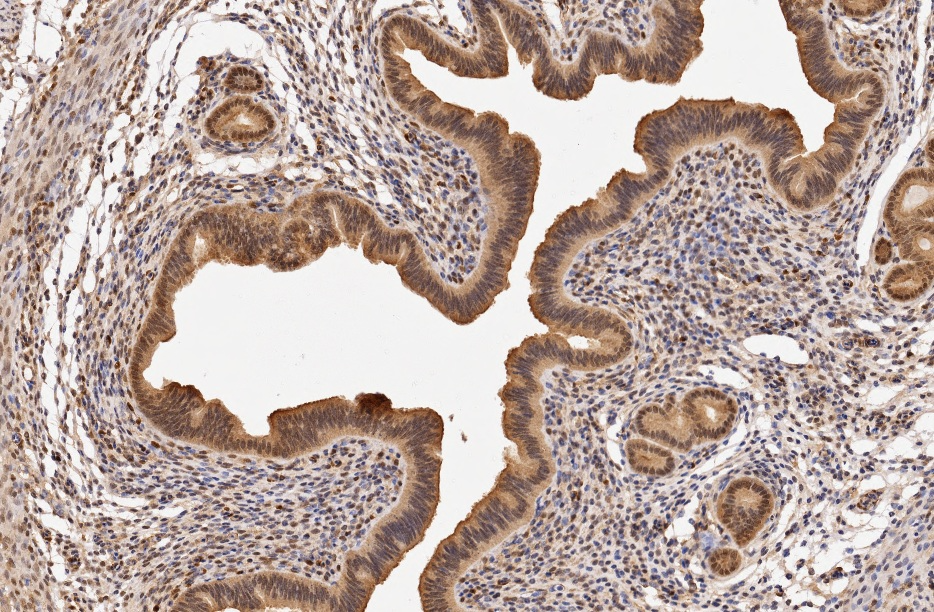

Supplement: Supplementary file 14 — Source Data for Figure 1 [file EMMM-15-e17094-s008.zip › EMM-2022-17094_source_data_figure_1/figure_1F/1F wt_tbpw_8.2f_pten_@10x.jpg]

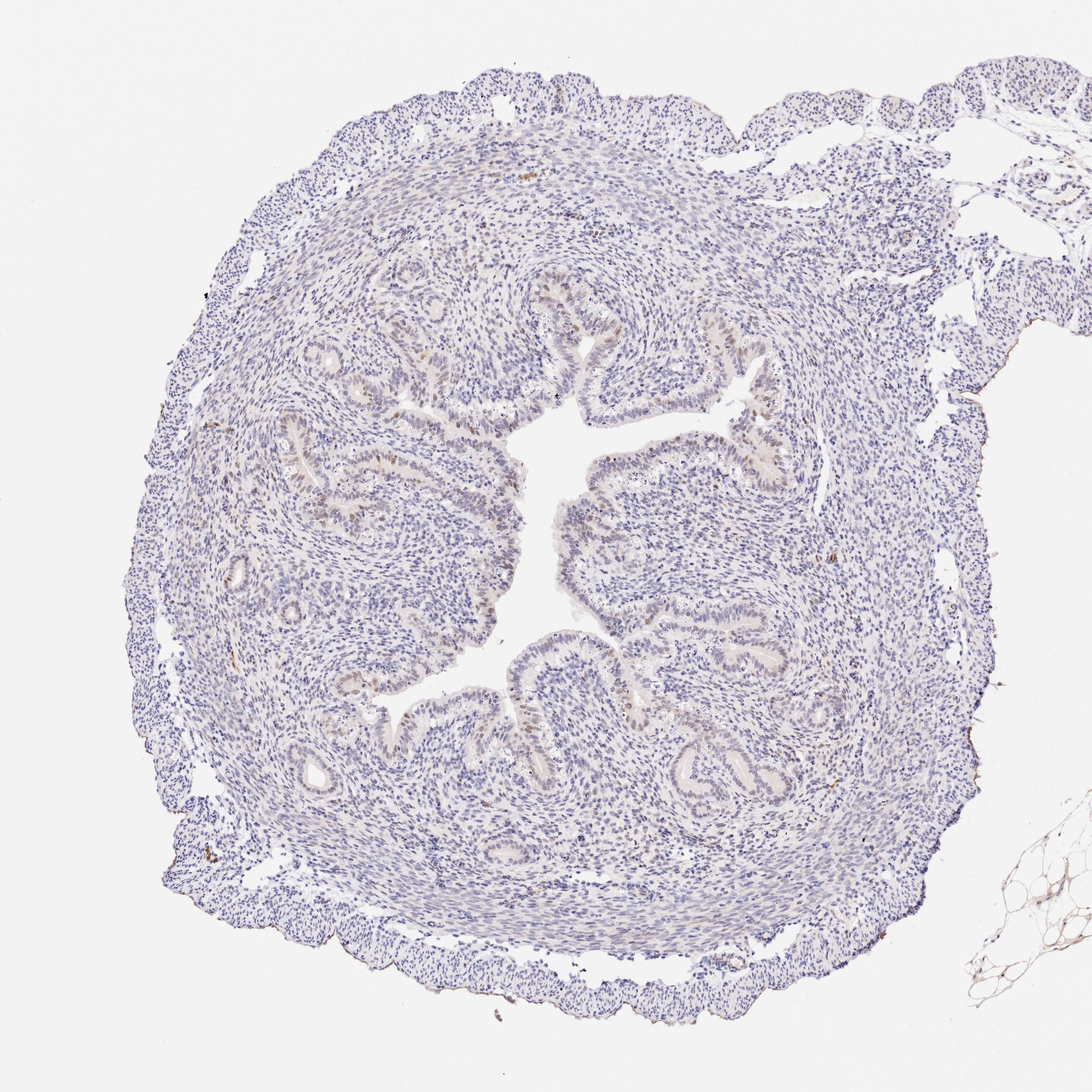

Supplement: Supplementary file 14 — Source Data for Figure 1 [file EMMM-15-e17094-s008.zip › EMM-2022-17094_source_data_figure_1/figure_1F/1F trp53del_fbxw7mut_tbow_13.3f_pAkt_@10x.jpg]

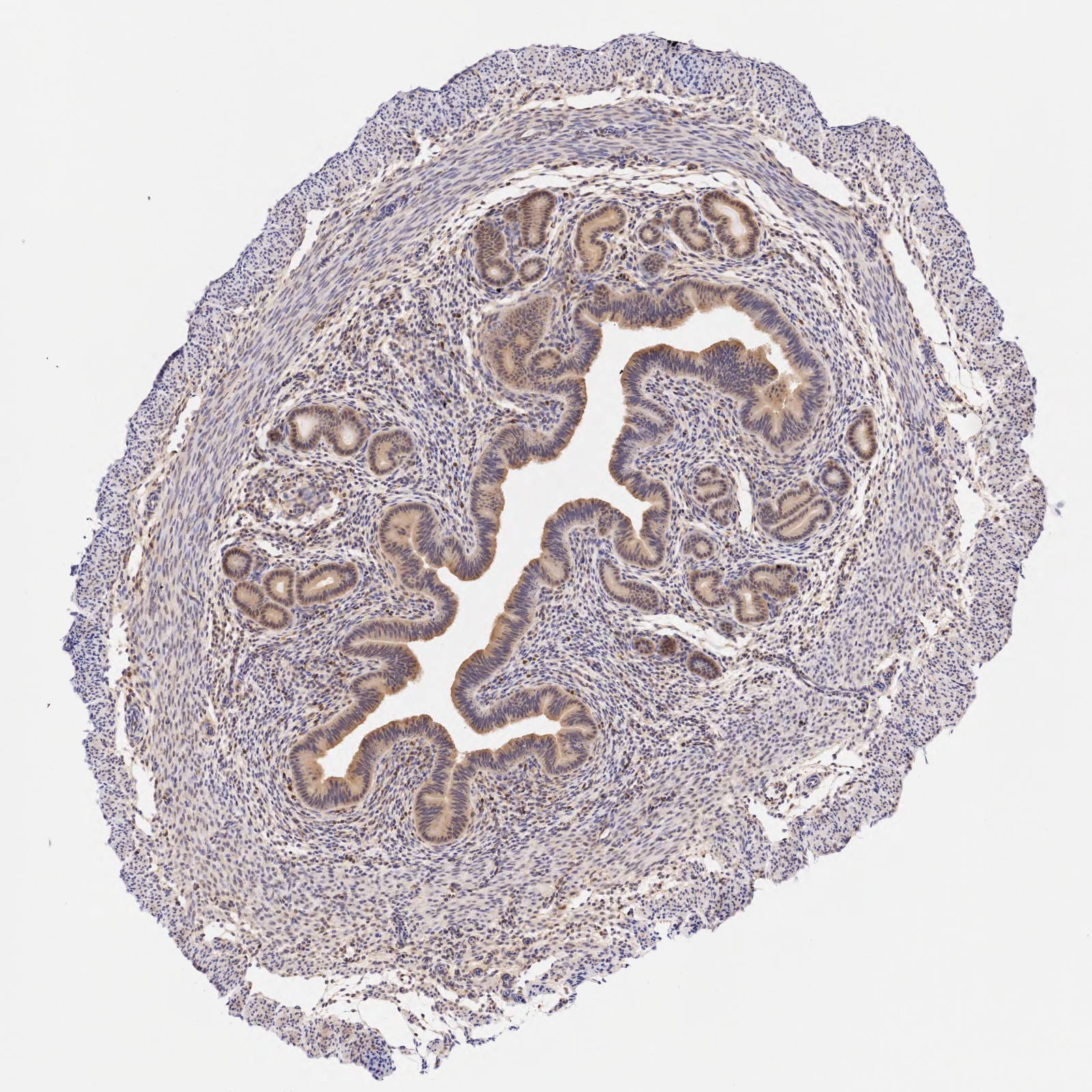

Supplement: Supplementary file 14 — Source Data for Figure 1 [file EMMM-15-e17094-s008.zip › EMM-2022-17094_source_data_figure_1/figure_1F/1F trp53mut_tprp_11.3h_pten_10x.jpg]

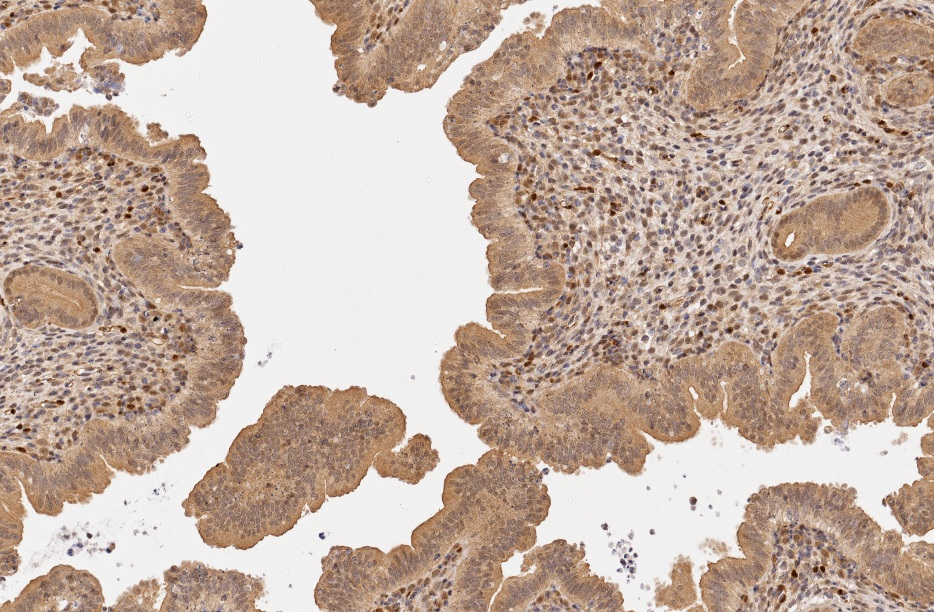

Supplement: Supplementary file 14 — Source Data for Figure 1 [file EMMM-15-e17094-s008.zip › EMM-2022-17094_source_data_figure_1/figure_1F/1F fbxw7mut_tbpw_8.2g_pten_@10x.jpg]

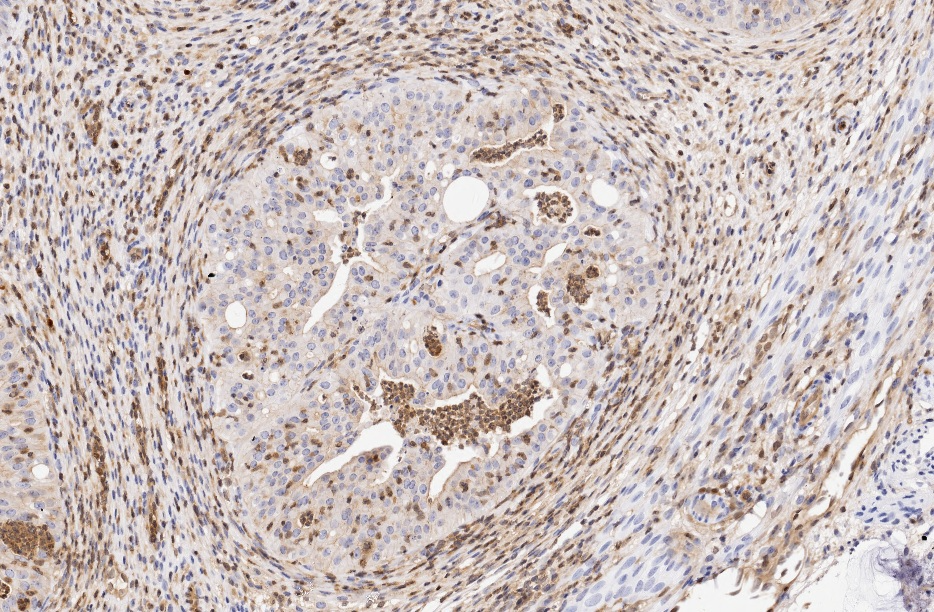

Supplement: Supplementary file 14 — Source Data for Figure 1 [file EMMM-15-e17094-s008.zip › EMM-2022-17094_source_data_figure_1/figure_1F/1F pten_tbnw_15.1f_pten_@10x.jpg]

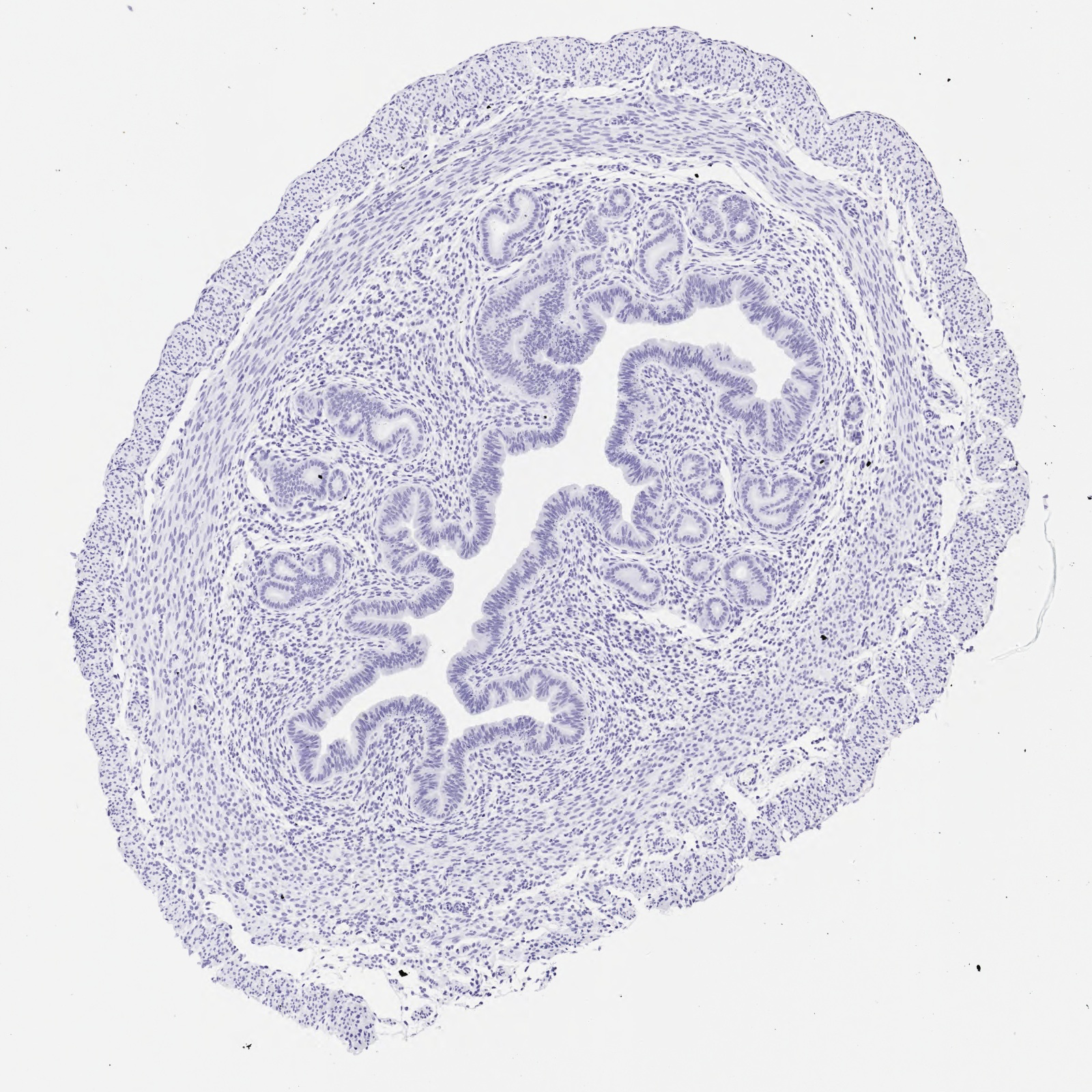

Supplement: Supplementary file 14 — Source Data for Figure 1 [file EMMM-15-e17094-s008.zip › EMM-2022-17094_source_data_figure_1/figure_1F/1F trp53mut_tprp_11.3h_pakt_10x.jpg]

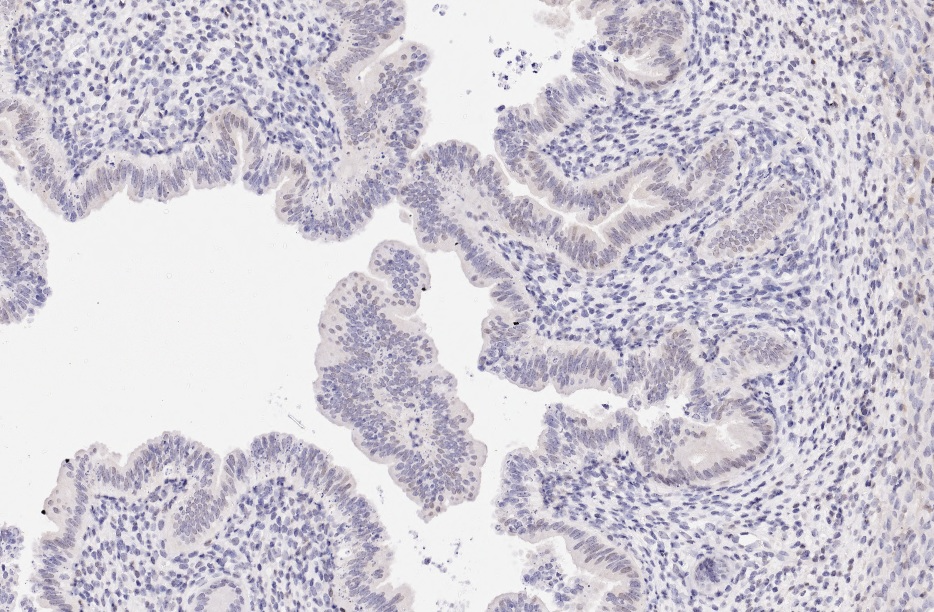

Supplement: Supplementary file 14 — Source Data for Figure 1 [file EMMM-15-e17094-s008.zip › EMM-2022-17094_source_data_figure_1/figure_1F/1F fbwx7mut_tbpw_8.2g_pAkt_@10x.jpg]

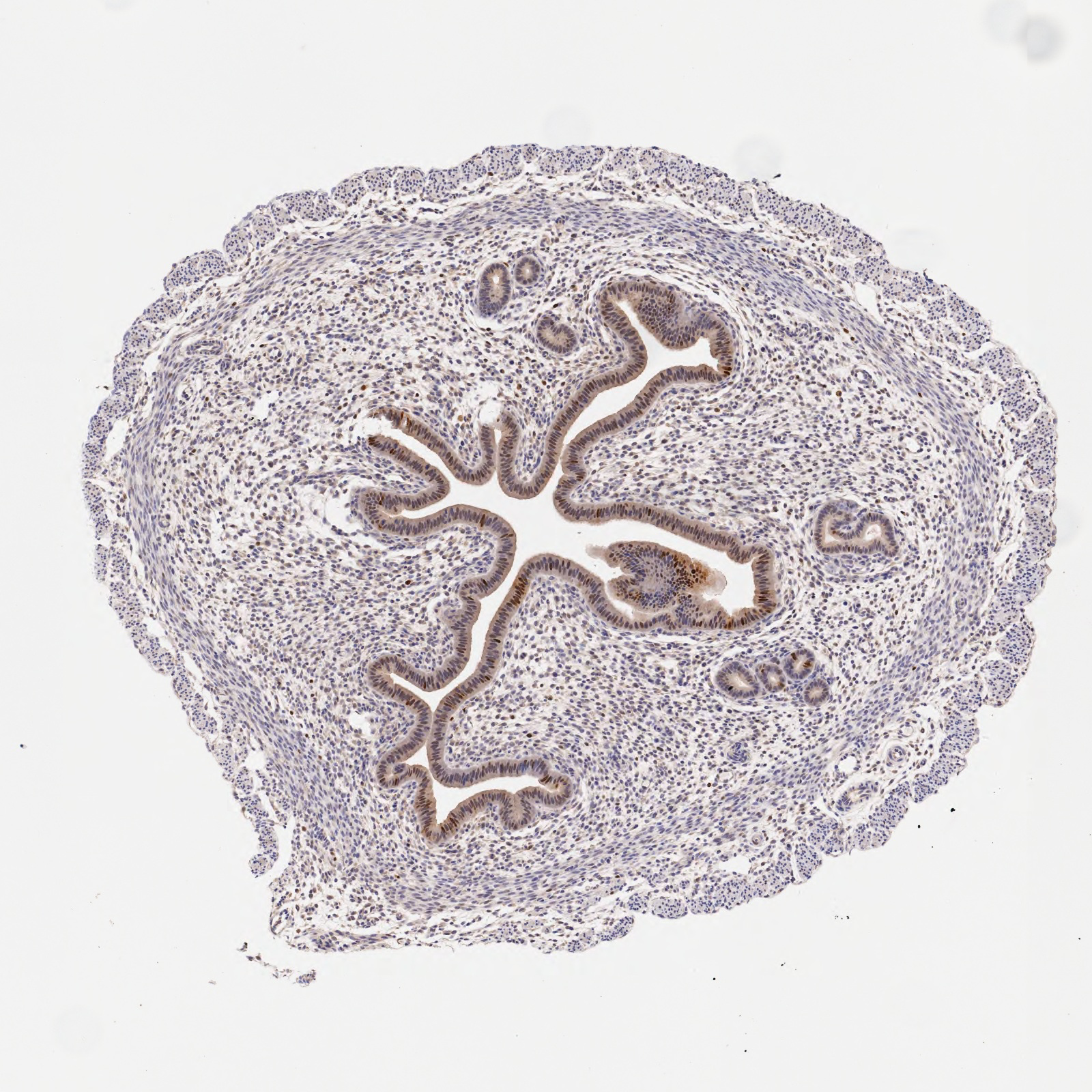

Supplement: Supplementary file 14 — Source Data for Figure 1 [file EMMM-15-e17094-s008.zip › EMM-2022-17094_source_data_figure_1/figure_1F/1F trp53mut_fbxw7mut_tprp_11.3i_p53_10x.jpg]

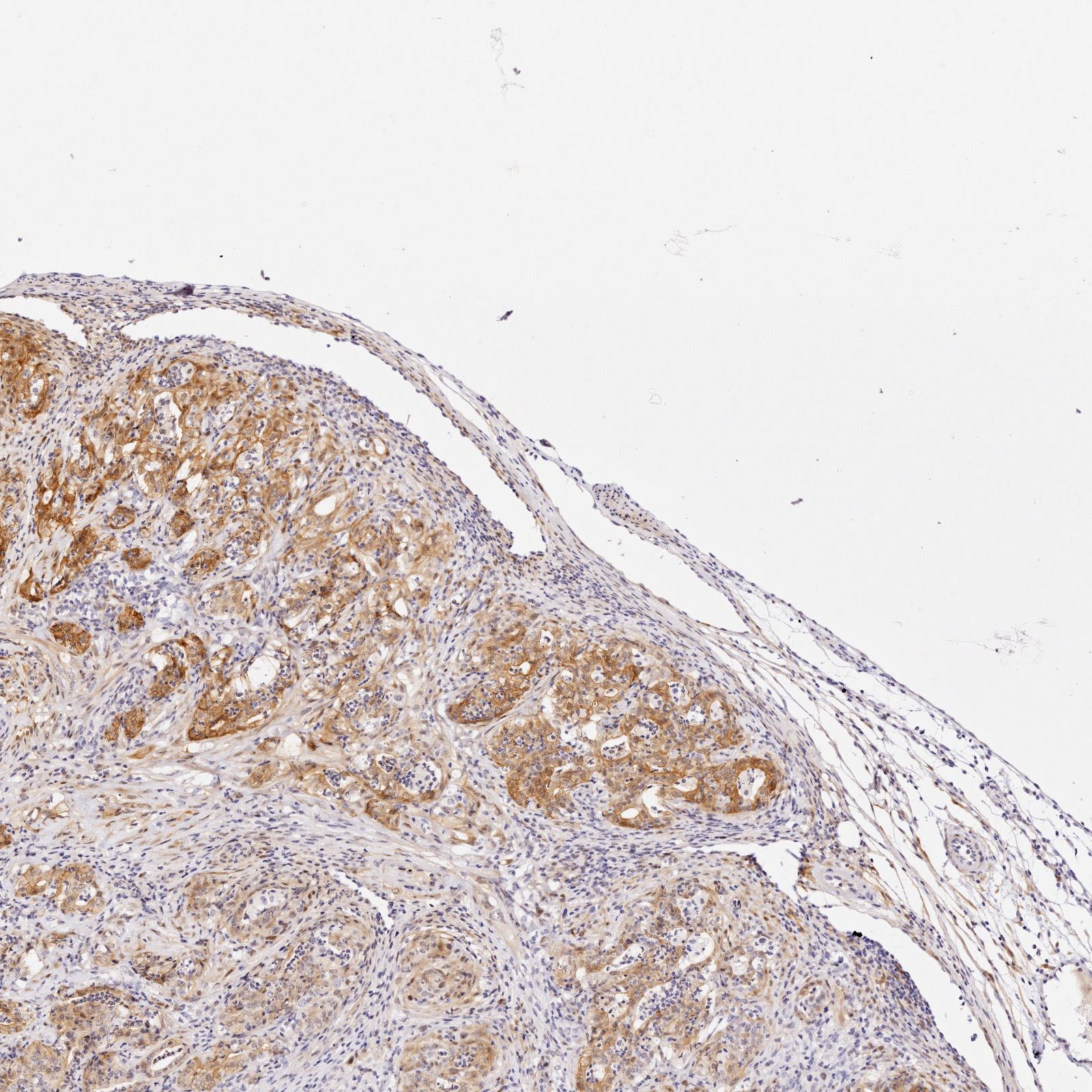

Supplement: Supplementary file 14 — Source Data for Figure 1 [file EMMM-15-e17094-s008.zip › EMM-2022-17094_source_data_figure_1/figure_1F/1F pten_fbxw7mut_tbnw_23_1d_pAkt_@10x.png]

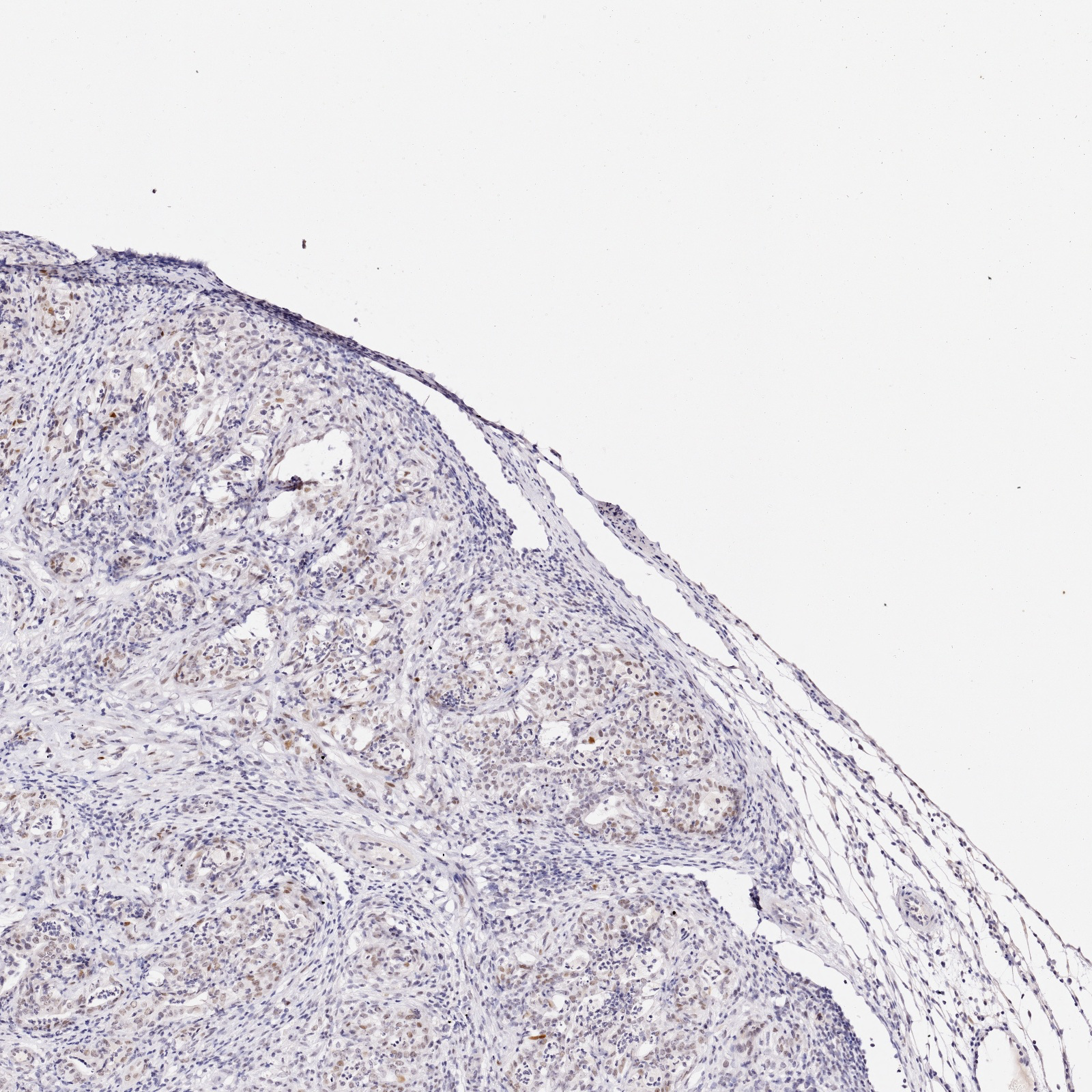

Supplement: Supplementary file 14 — Source Data for Figure 1 [file EMMM-15-e17094-s008.zip › EMM-2022-17094_source_data_figure_1/figure_1F/1F pten_fbxw7mut_tbnw_23_1d_p53_@10x.jpg]

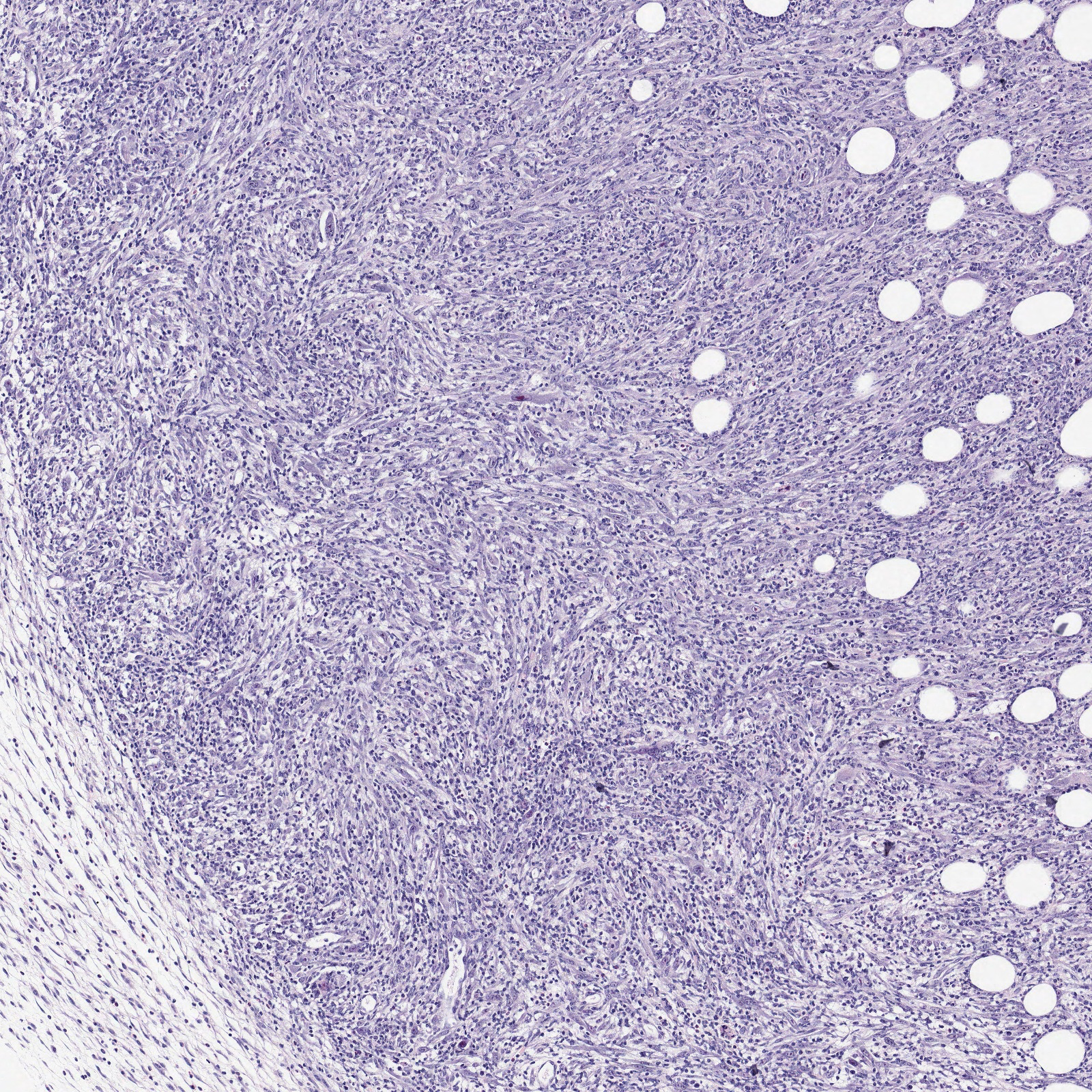

Supplement: Supplementary file 15 — Source Data for Figure 2 [file EMMM-15-e17094-s003.zip › EMM-2022-17094_source_data_figure_2/figure_2E/tprp_6.1d_r172_r482q_he_10x.jpg]

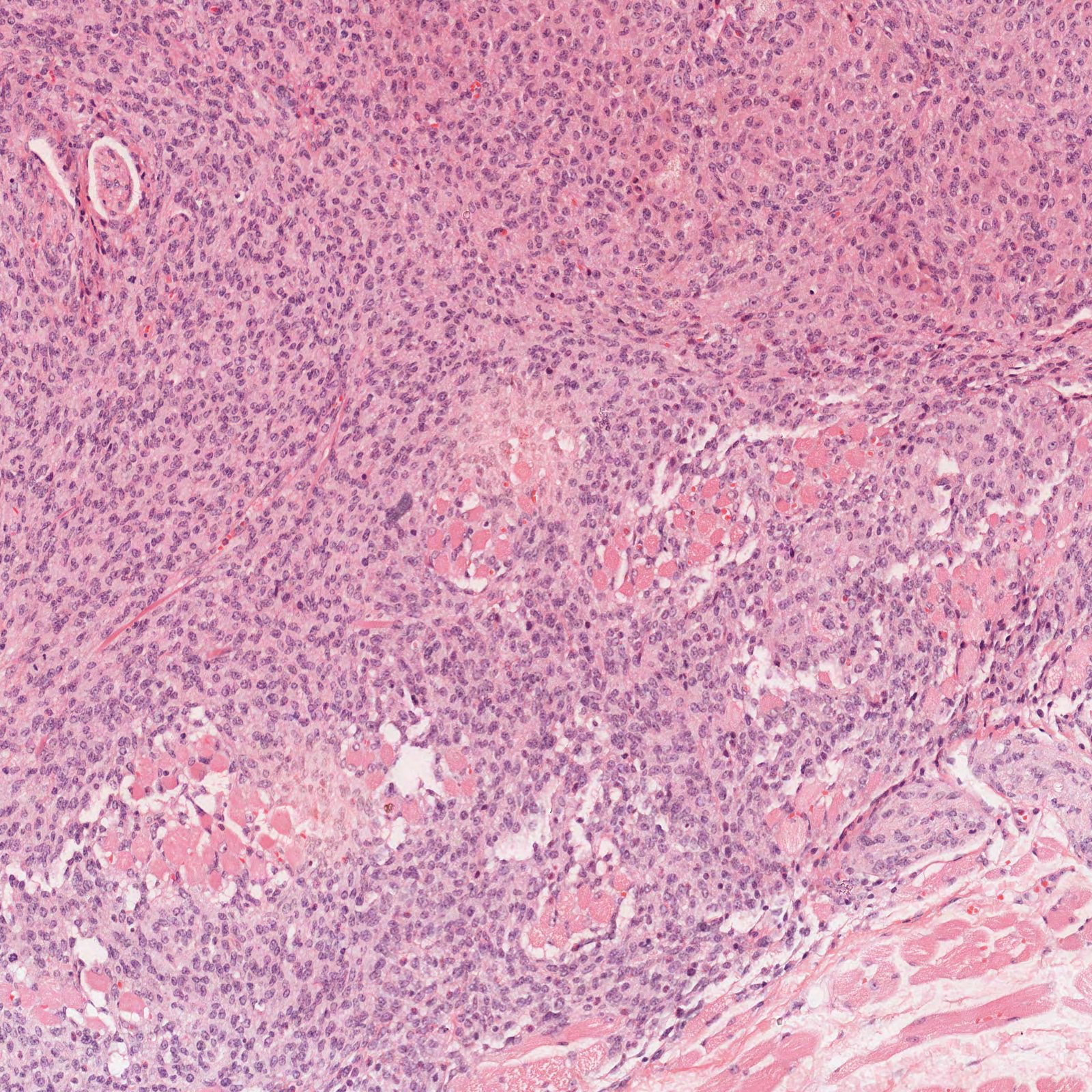

Supplement: Supplementary file 15 — Source Data for Figure 2 [file EMMM-15-e17094-s003.zip › EMM-2022-17094_source_data_figure_2/figure_2E/tbow11.2e_p53_tumour_20x.jpg]

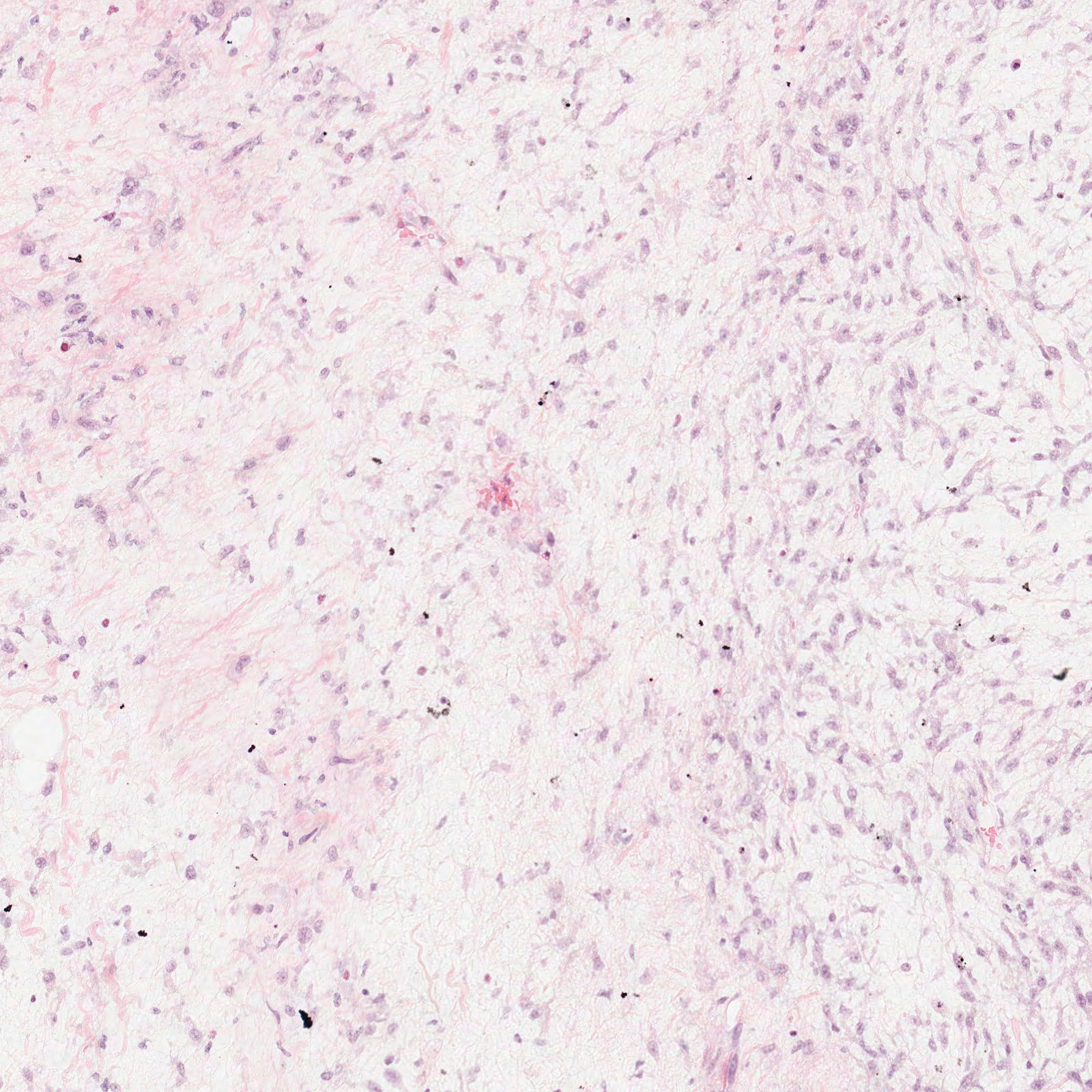

Supplement: Supplementary file 15 — Source Data for Figure 2 [file EMMM-15-e17094-s003.zip › EMM-2022-17094_source_data_figure_2/figure_2E/tbow12.1e_p53r482q_tumour_20x.jpg]

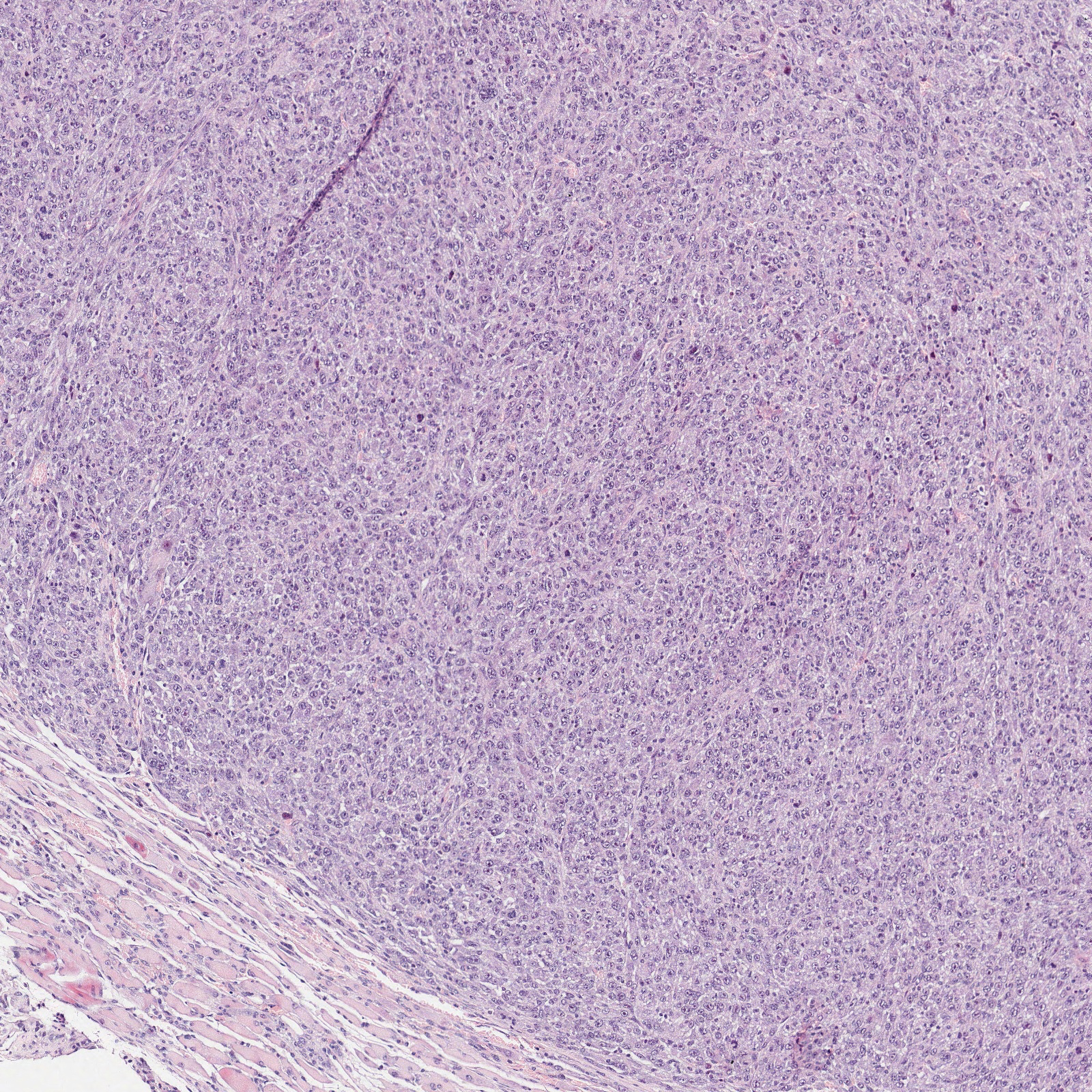

Supplement: Supplementary file 15 — Source Data for Figure 2 [file EMMM-15-e17094-s003.zip › EMM-2022-17094_source_data_figure_2/figure_2E/tprp1.3e_r172h_r482q_he_10x.jpg]

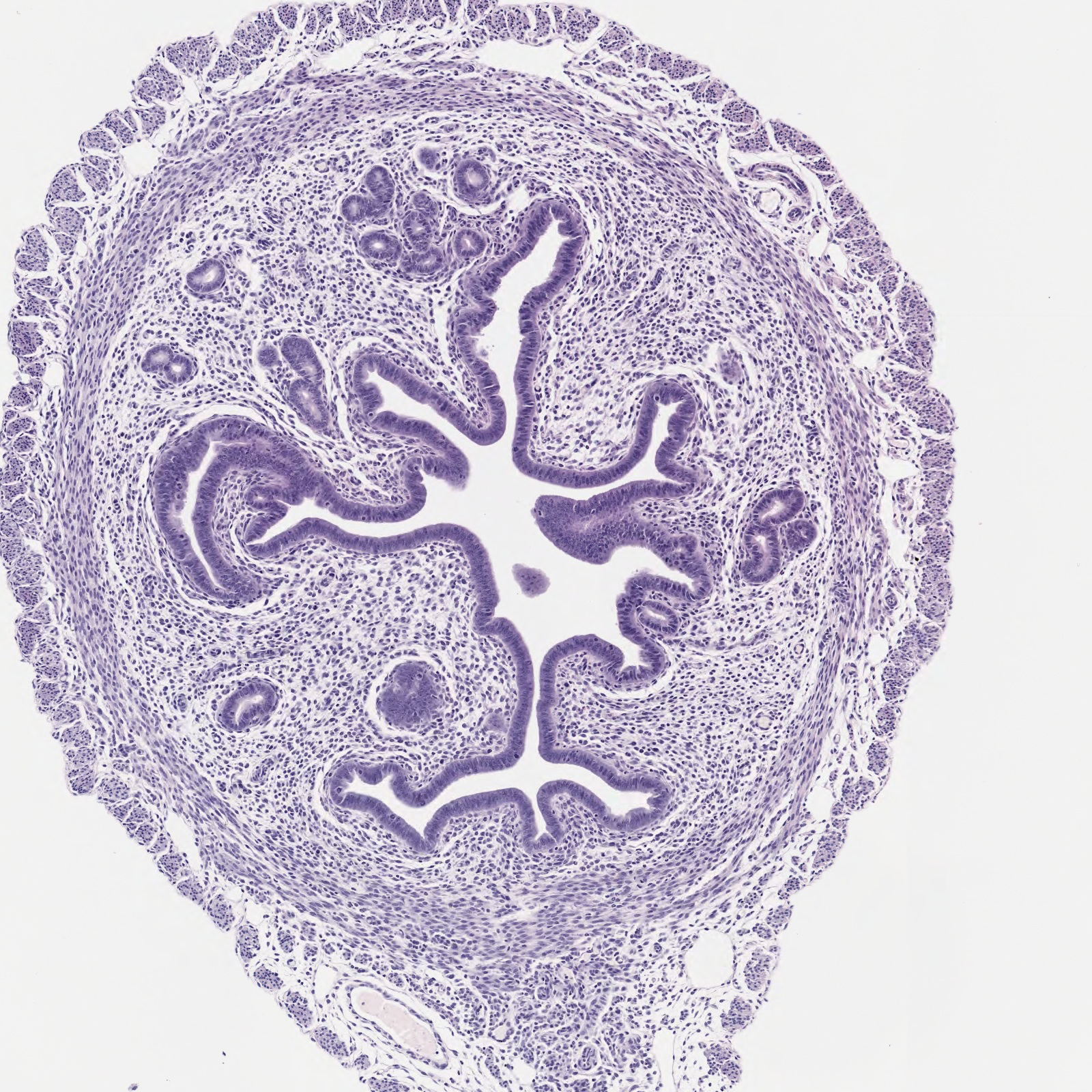

Supplement: Supplementary file 15 — Source Data for Figure 2 [file EMMM-15-e17094-s003.zip › EMM-2022-17094_source_data_figure_2/figure_2C/8 weeks/trp53mut_fbxw7mut_tprp_11.3i_r172h_r482q_he_10x.jpg]

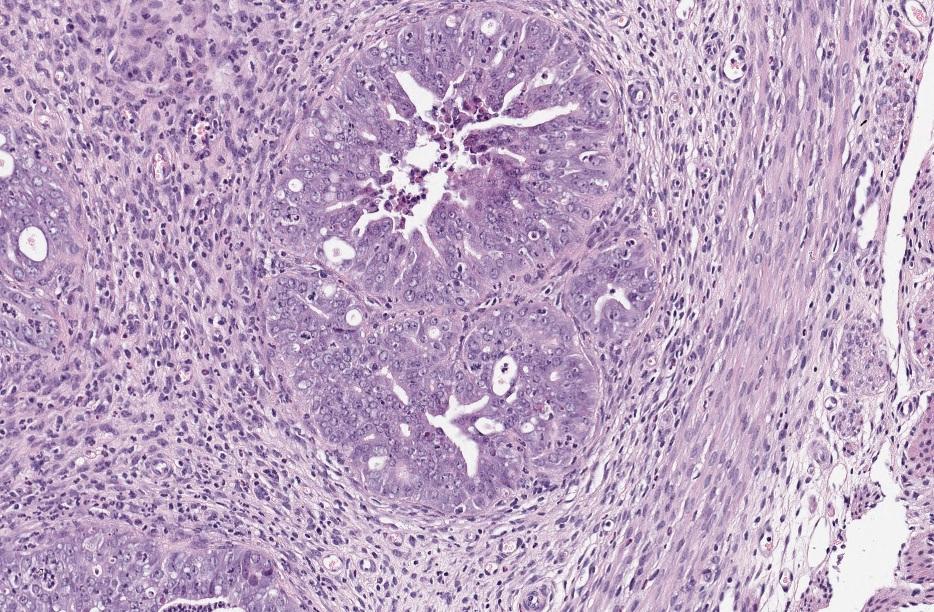

Supplement: Supplementary file 15 — Source Data for Figure 2 [file EMMM-15-e17094-s003.zip › EMM-2022-17094_source_data_figure_2/figure_2C/8 weeks/pten_tbnw_15.1f_pten_h&e_@10x.jpg]

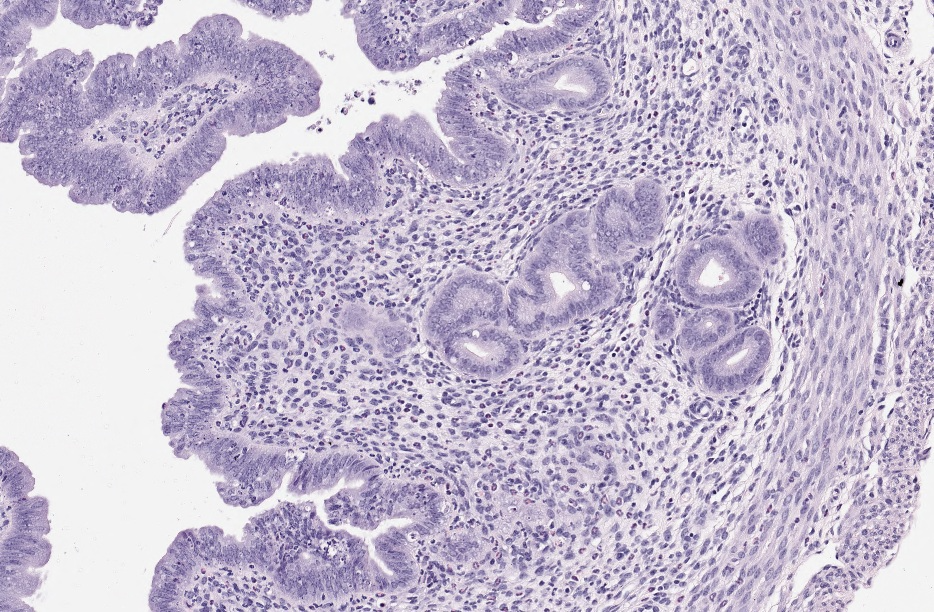

Supplement: Supplementary file 15 — Source Data for Figure 2 [file EMMM-15-e17094-s003.zip › EMM-2022-17094_source_data_figure_2/figure_2C/8 weeks/fbxw7mut_tbpw_8.2g_r482q_h&e_@10x.jpg]

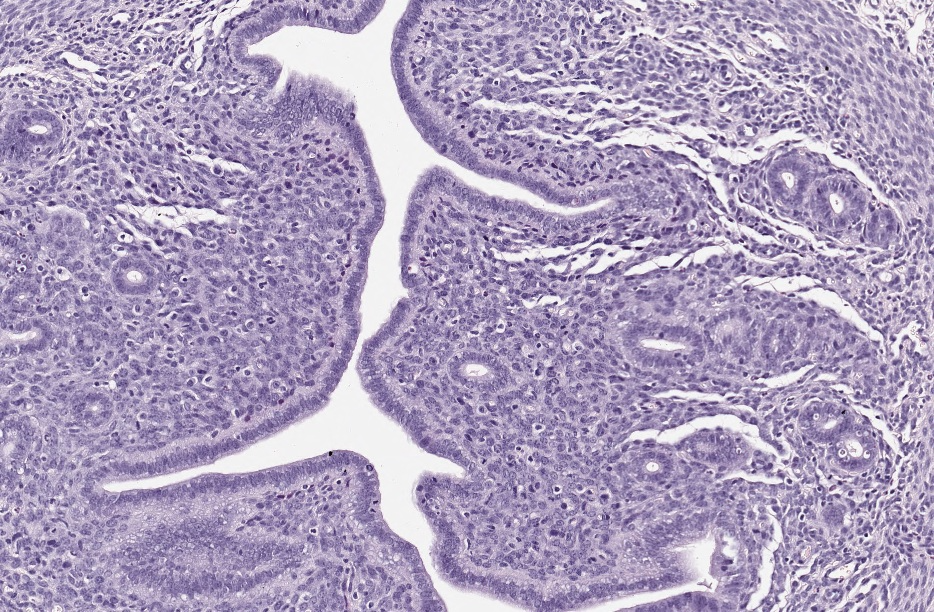

Supplement: Supplementary file 15 — Source Data for Figure 2 [file EMMM-15-e17094-s003.zip › EMM-2022-17094_source_data_figure_2/figure_2C/8 weeks/trp53del_tbow_13.3e_tp53del_h&e_@10x.jpg]

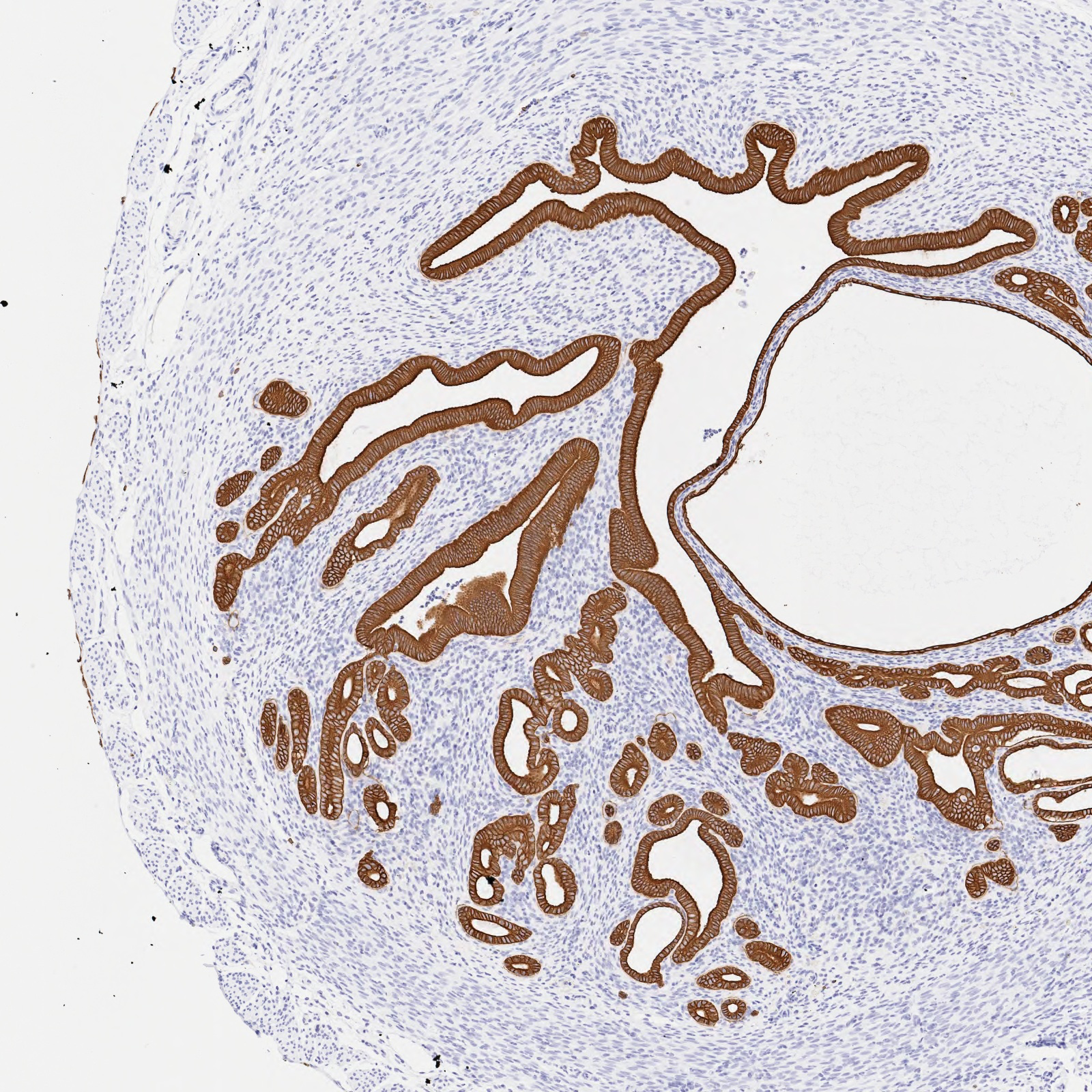

Supplement: Supplementary file 15 — Source Data for Figure 2 [file EMMM-15-e17094-s003.zip › EMM-2022-17094_source_data_figure_2/figure_2C/8 weeks/tbpw_4.1c_wt_survival_ck_10x.jpg]

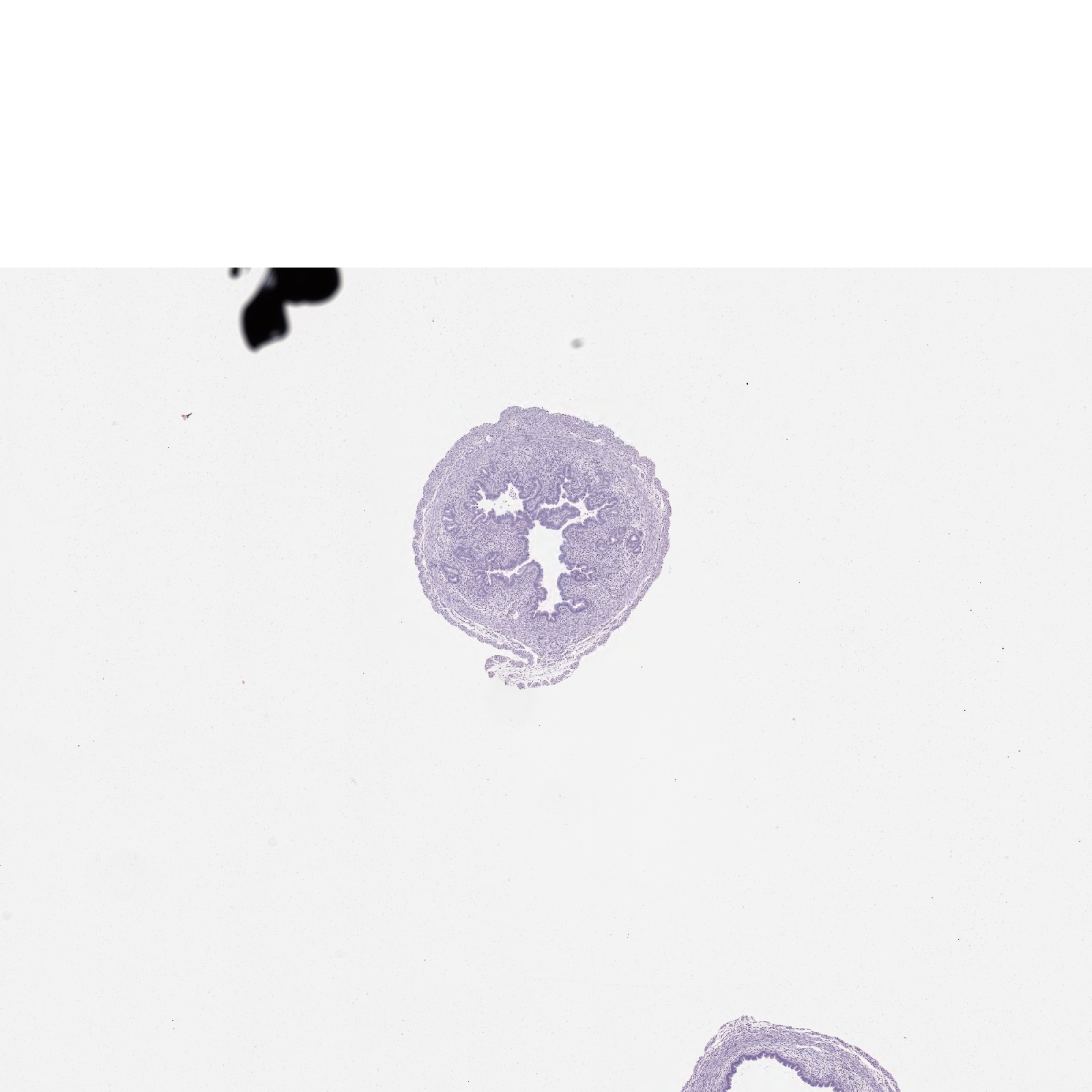

Supplement: Supplementary file 15 — Source Data for Figure 2 [file EMMM-15-e17094-s003.zip › EMM-2022-17094_source_data_figure_2/figure_2C/8 weeks/fbxw7mut_tbpw_8.2g_r482q_h&e_@2x.jpg]

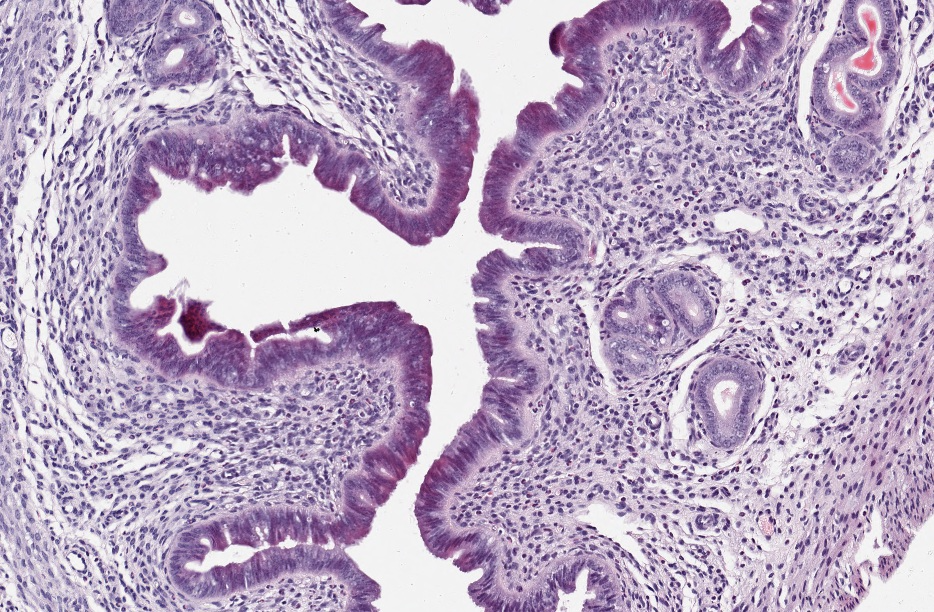

Supplement: Supplementary file 15 — Source Data for Figure 2 [file EMMM-15-e17094-s003.zip › EMM-2022-17094_source_data_figure_2/figure_2C/8 weeks/wt_tbpw_8.2f_wt_h&e_@10x.jpg]

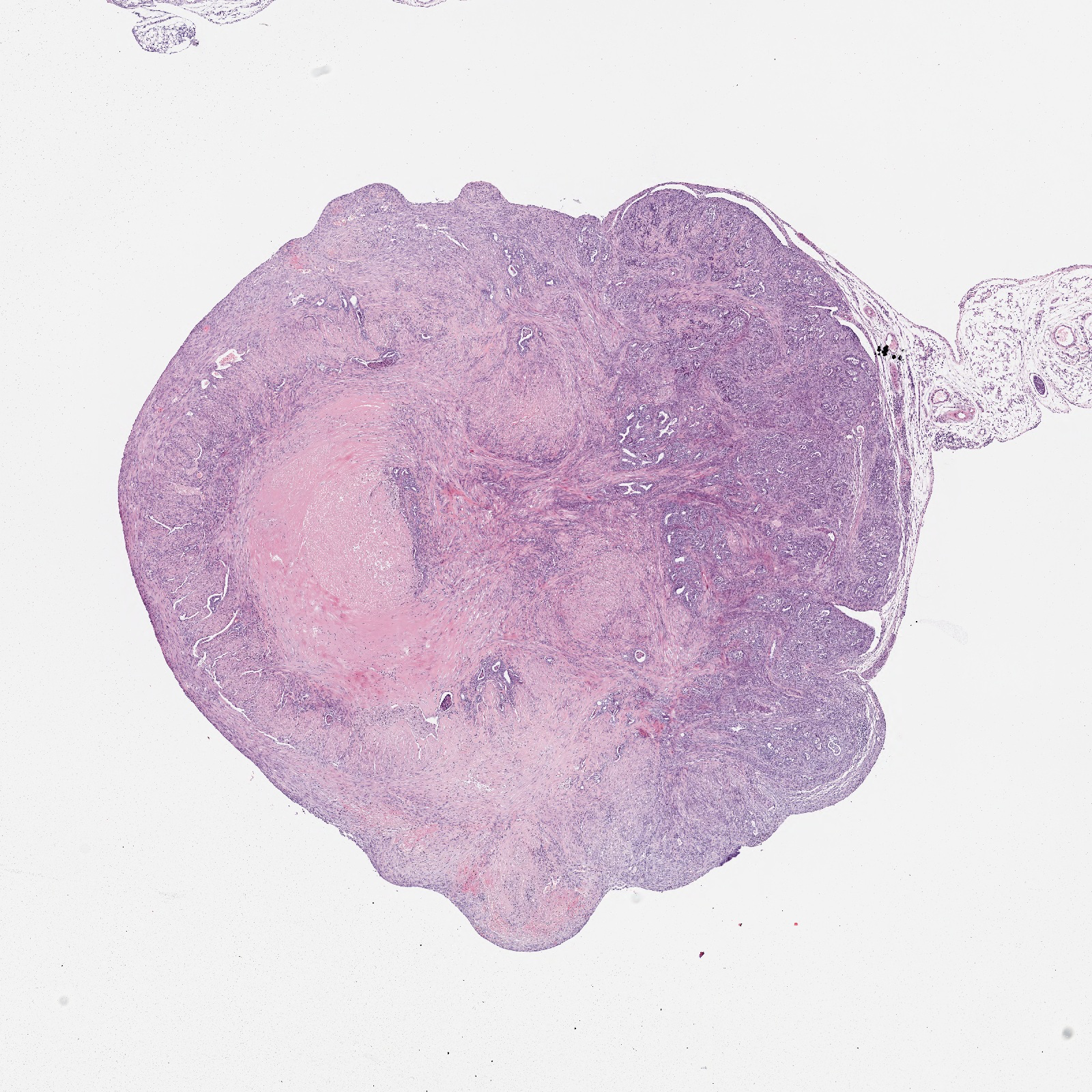

Supplement: Supplementary file 15 — Source Data for Figure 2 [file EMMM-15-e17094-s003.zip › EMM-2022-17094_source_data_figure_2/figure_2C/8 weeks/ptendel_fbxw7mut_tbnw_23_1d_ptendelr482q_h&e_@2x.jpg]

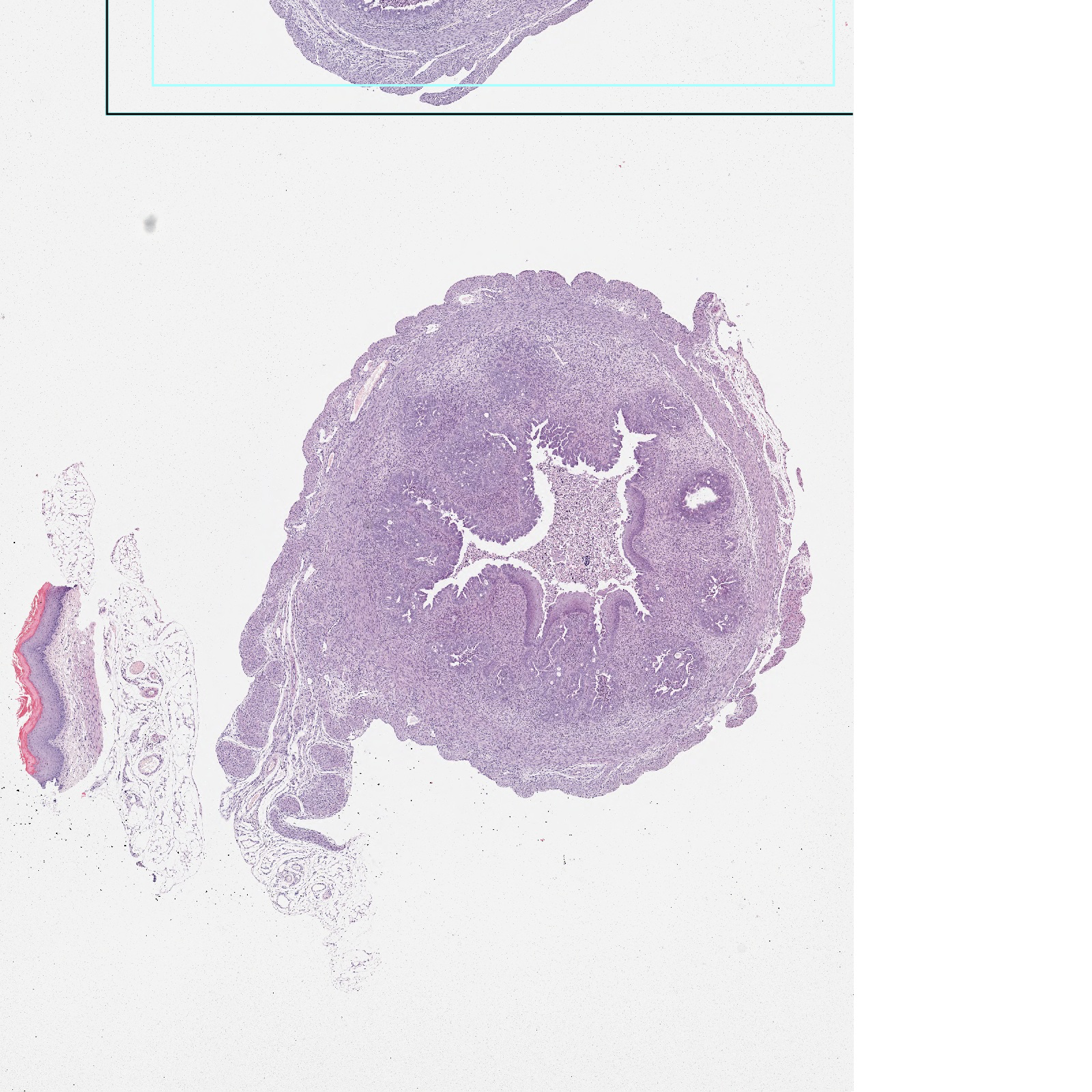

Supplement: Supplementary file 15 — Source Data for Figure 2 [file EMMM-15-e17094-s003.zip › EMM-2022-17094_source_data_figure_2/figure_2C/8 weeks/pten_tbnw_15.1f_pten_h&e_@2x.jpg]

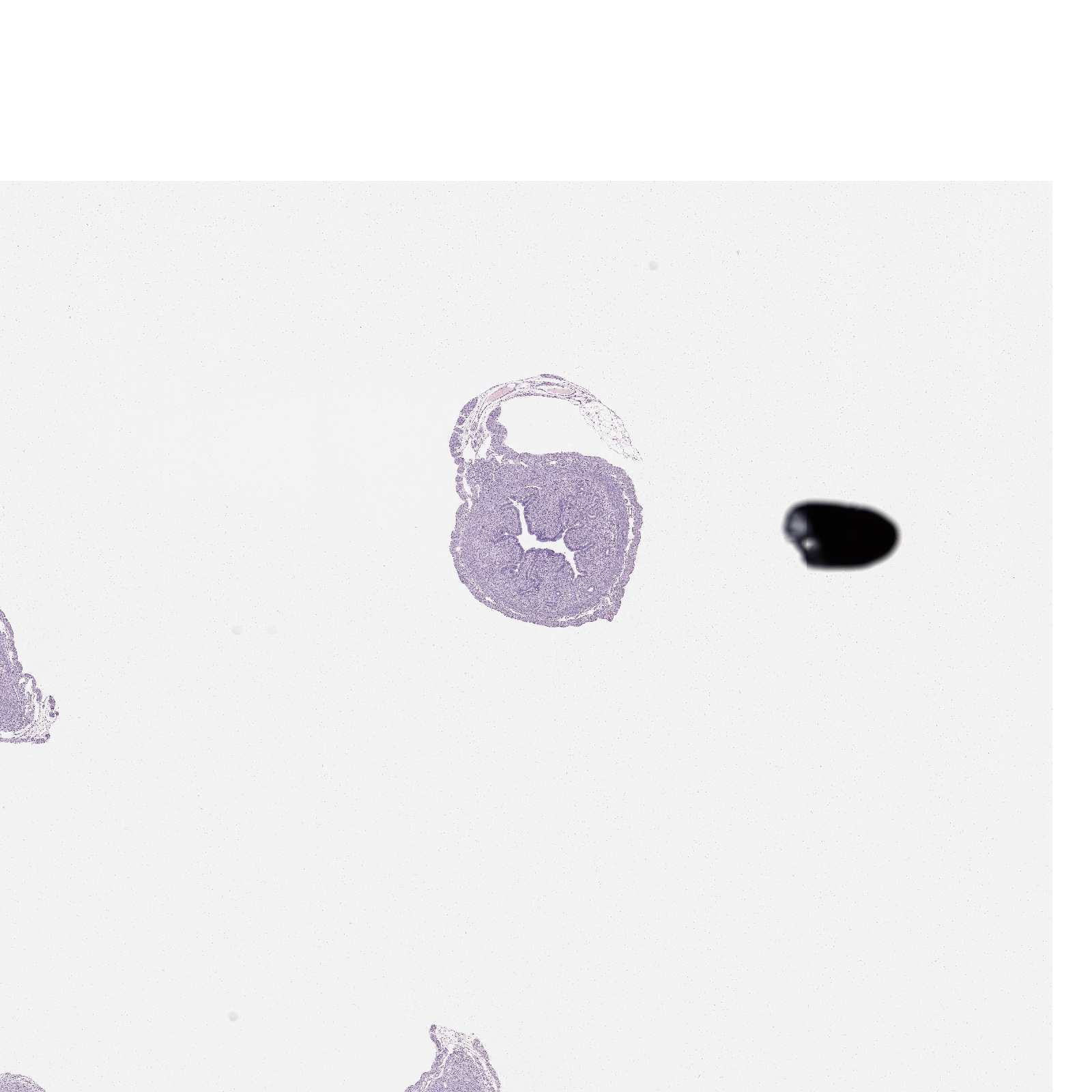

Supplement: Supplementary file 15 — Source Data for Figure 2 [file EMMM-15-e17094-s003.zip › EMM-2022-17094_source_data_figure_2/figure_2C/8 weeks/trp53del_fbxw7mut_tbow_13.3f_trp53del_r482q_h&E_@2x.jpg]

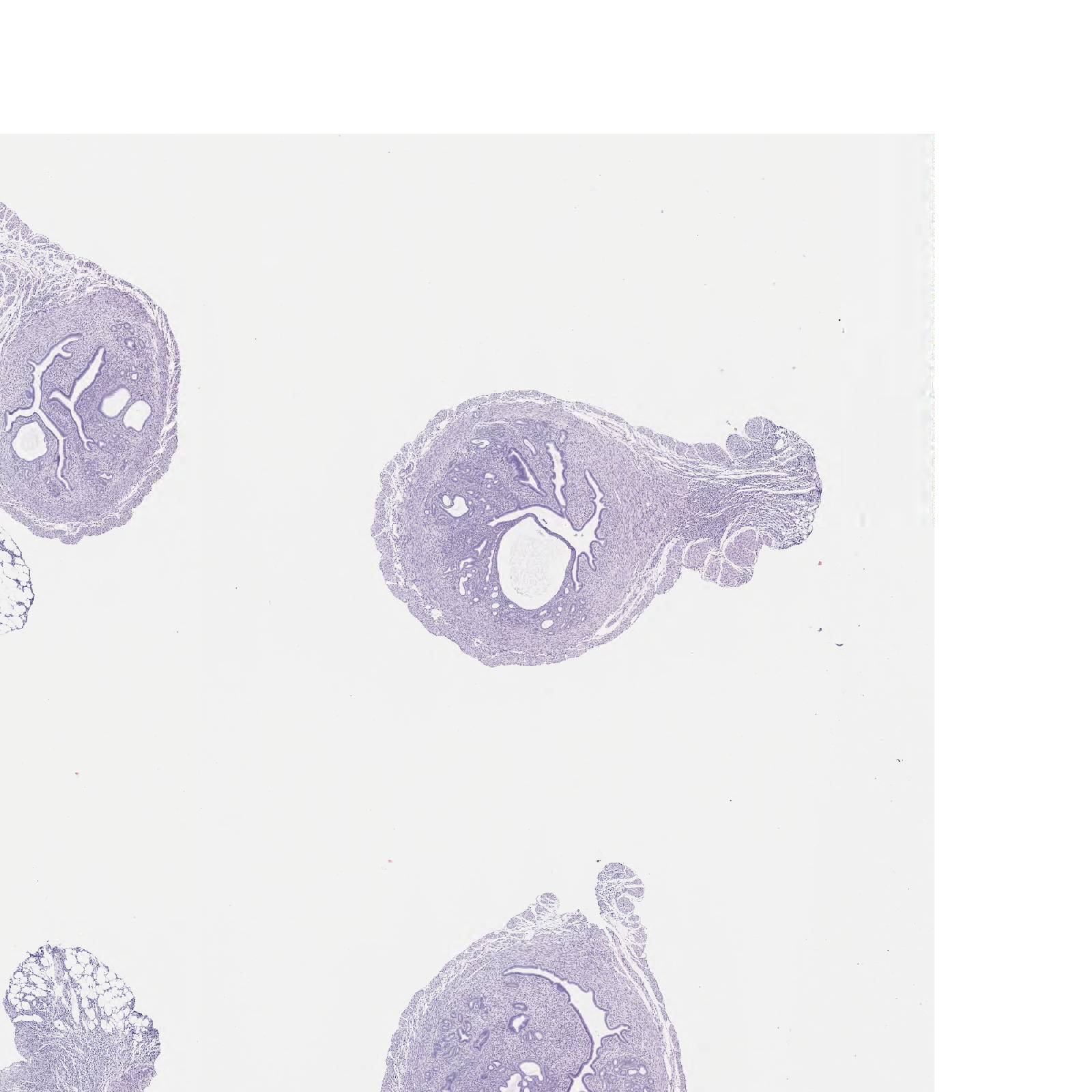

Supplement: Supplementary file 15 — Source Data for Figure 2 [file EMMM-15-e17094-s003.zip › EMM-2022-17094_source_data_figure_2/figure_2C/8 weeks/tbpw_4.1c_wt_survival_he_2x.jpg]

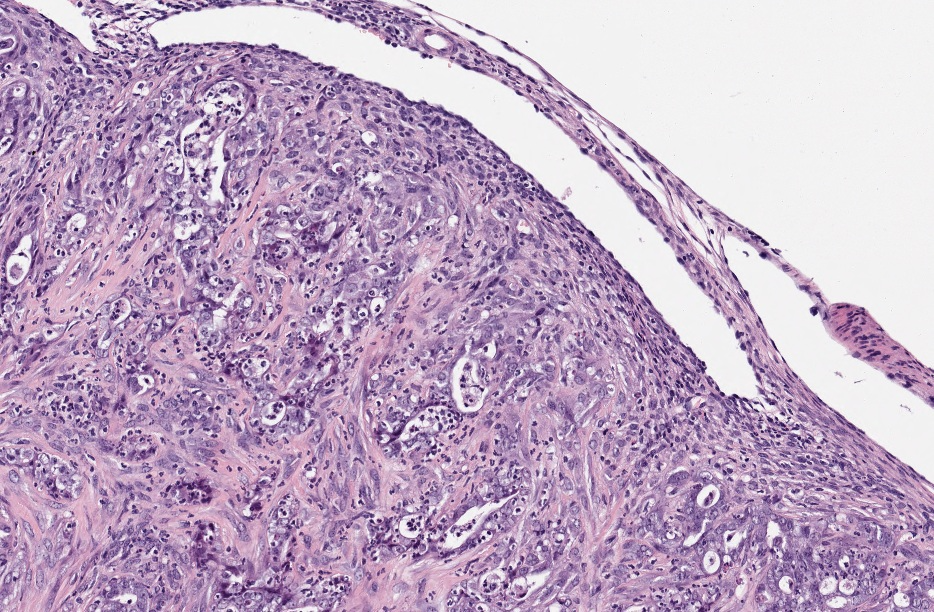

Supplement: Supplementary file 15 — Source Data for Figure 2 [file EMMM-15-e17094-s003.zip › EMM-2022-17094_source_data_figure_2/figure_2C/8 weeks/ptendel_fbxw7mut_tbnw_23_1d_ptendelr482q_h&e_@10x.jpg]

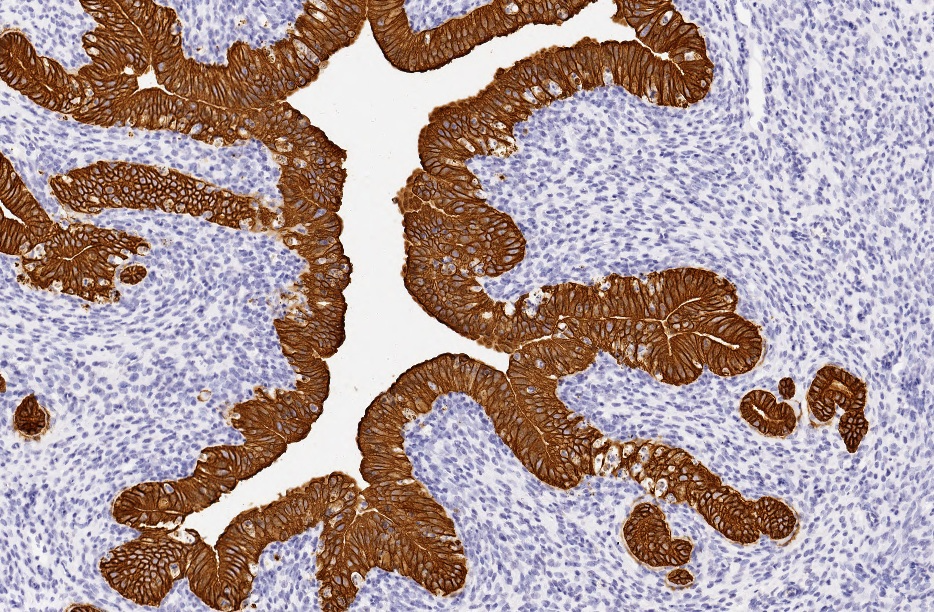

Supplement: Supplementary file 15 — Source Data for Figure 2 [file EMMM-15-e17094-s003.zip › EMM-2022-17094_source_data_figure_2/figure_2C/8 weeks/trp53del_fbxw7mut_tbow_13.3f_trp53del_r482q_ck8_@10x.jpg]

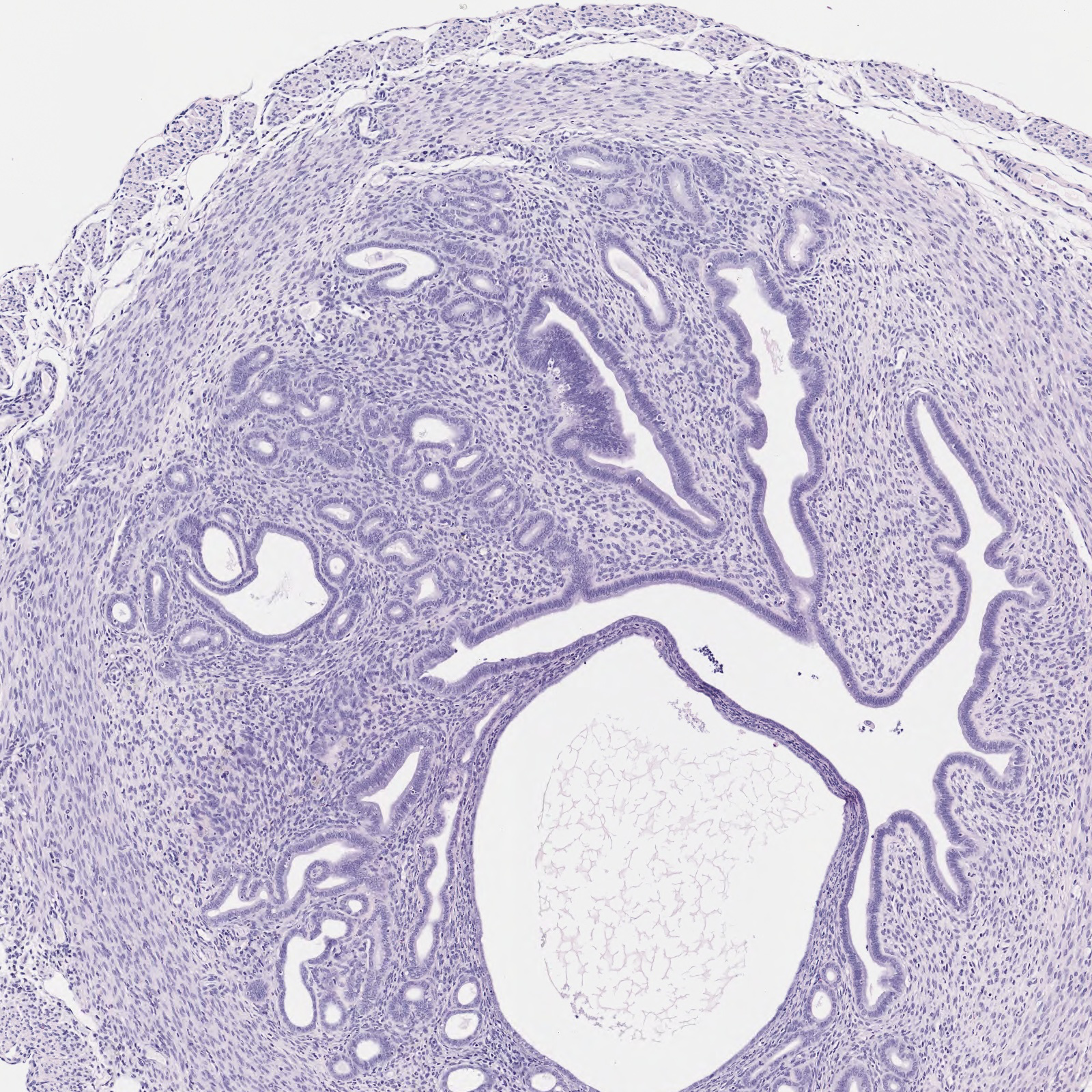

Supplement: Supplementary file 15 — Source Data for Figure 2 [file EMMM-15-e17094-s003.zip › EMM-2022-17094_source_data_figure_2/figure_2C/8 weeks/tbpw_4.1c_wt_survival_he_10x.jpg]

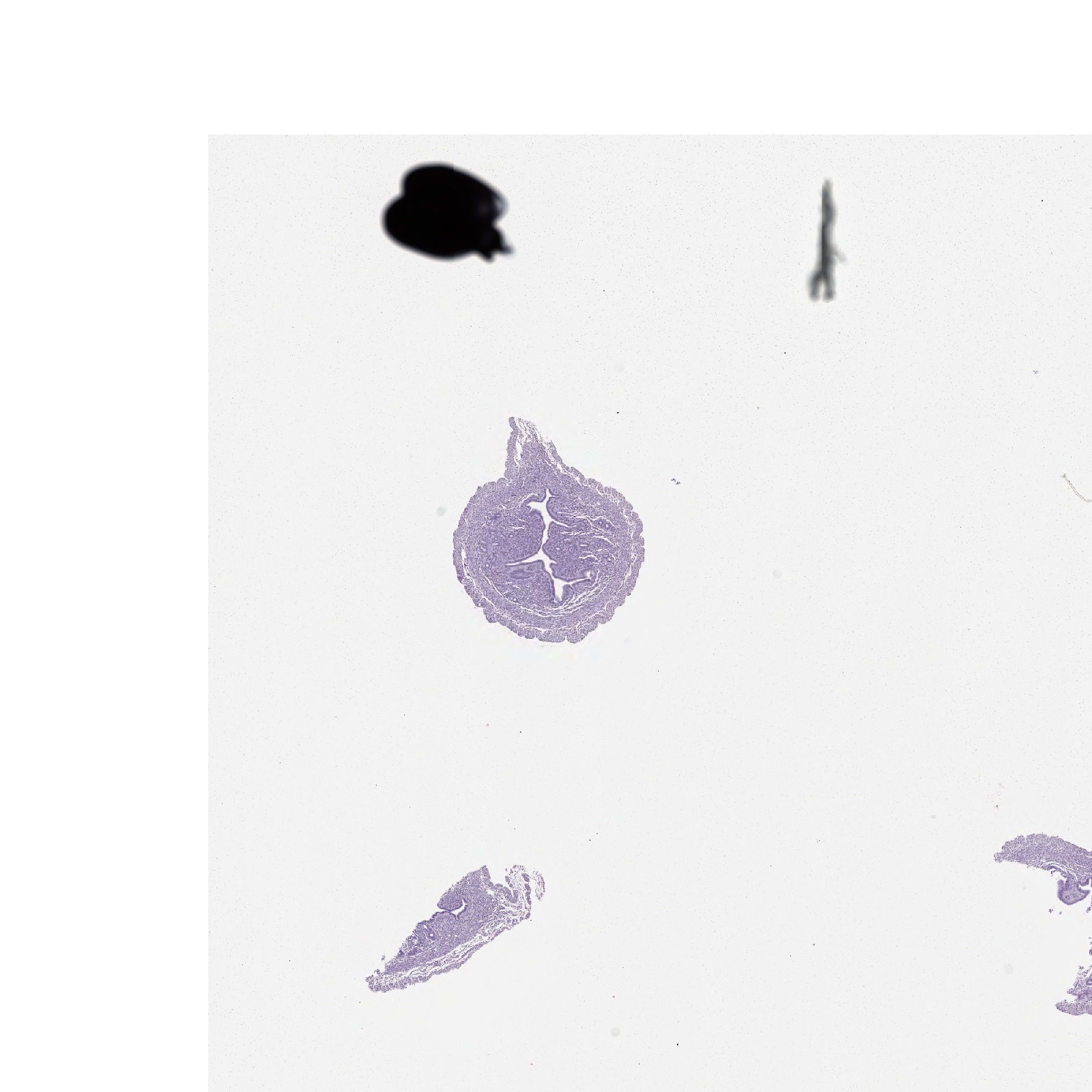

Supplement: Supplementary file 15 — Source Data for Figure 2 [file EMMM-15-e17094-s003.zip › EMM-2022-17094_source_data_figure_2/figure_2C/8 weeks/trp53del_tbow_13.3e_tp53del_h&e_@2x.jpg]

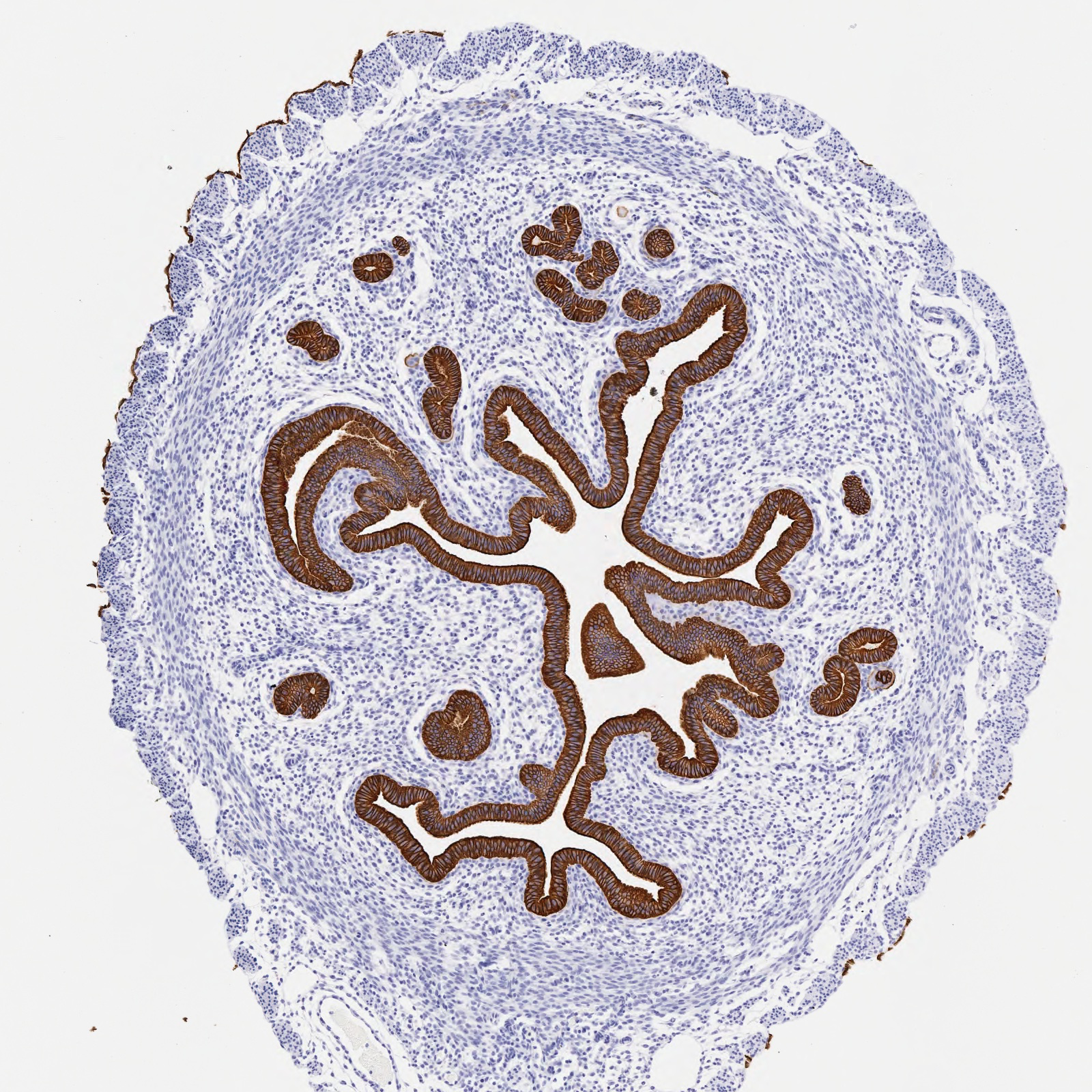

Supplement: Supplementary file 15 — Source Data for Figure 2 [file EMMM-15-e17094-s003.zip › EMM-2022-17094_source_data_figure_2/figure_2C/8 weeks/trp53mut_fbxw7mut_tprp_11.3i_r172h_r482q_ck_10x.jpg]

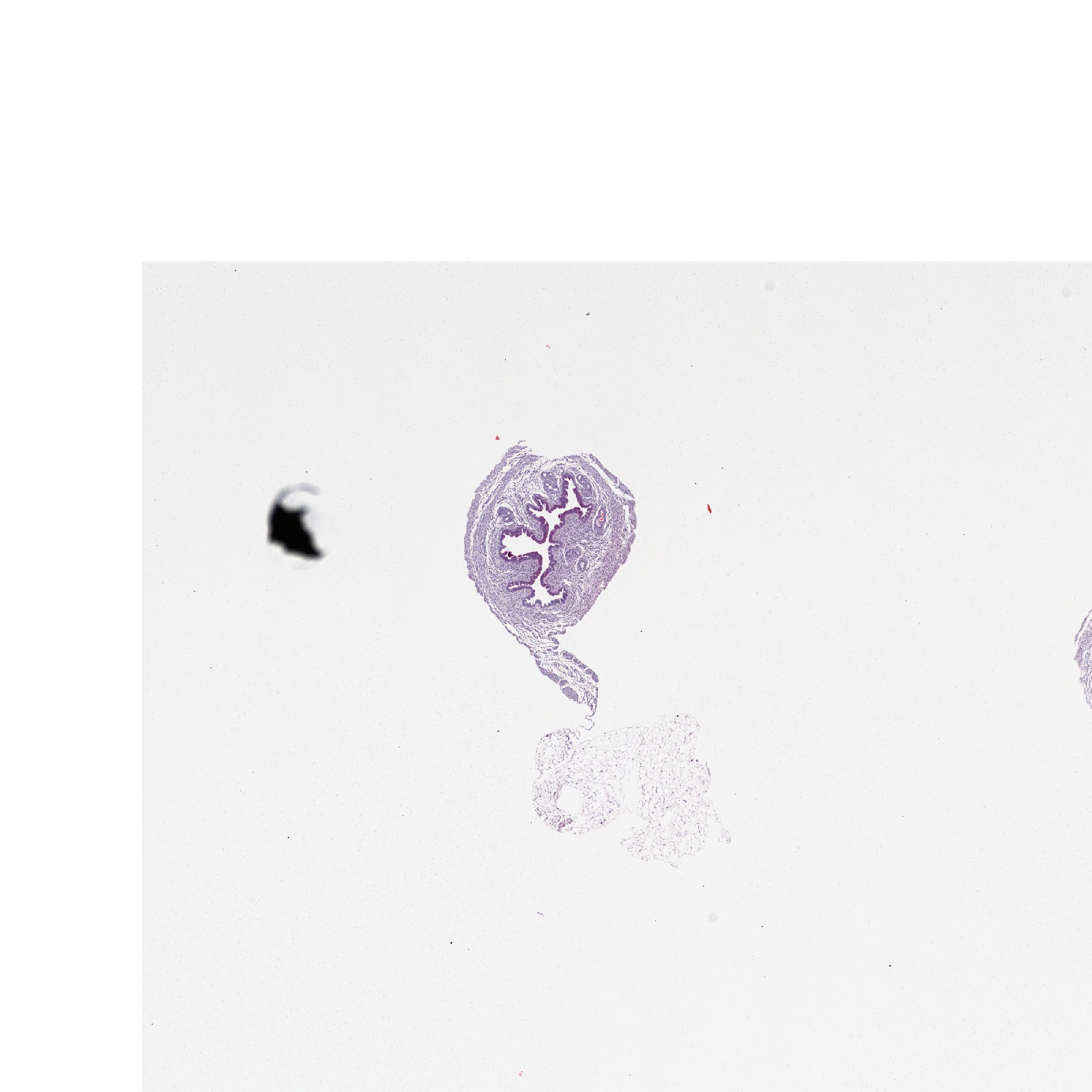

Supplement: Supplementary file 15 — Source Data for Figure 2 [file EMMM-15-e17094-s003.zip › EMM-2022-17094_source_data_figure_2/figure_2C/8 weeks/wt_tbpw_8.2f_wt_h&e_@2x.jpg]

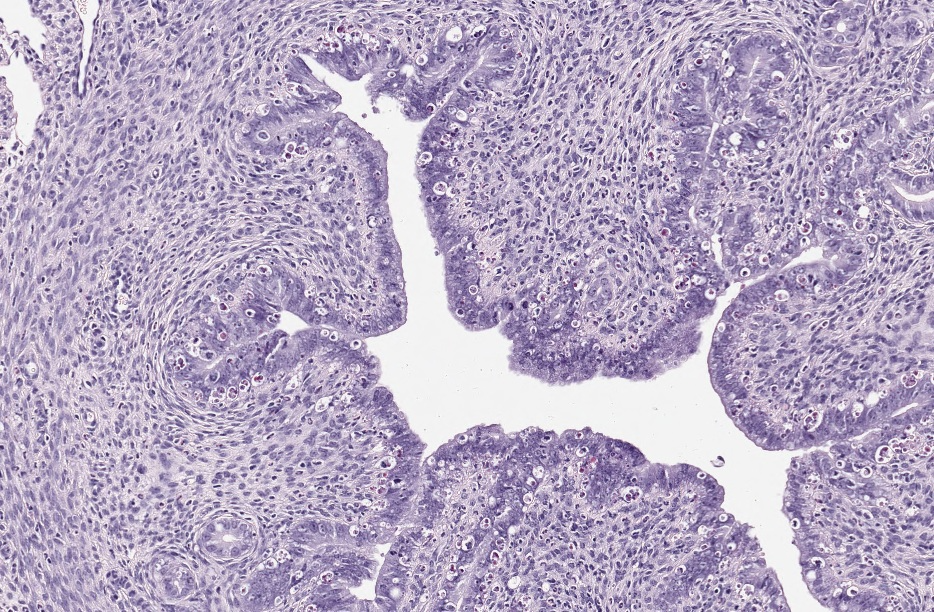

Supplement: Supplementary file 15 — Source Data for Figure 2 [file EMMM-15-e17094-s003.zip › EMM-2022-17094_source_data_figure_2/figure_2C/8 weeks/trp53del_fbxw7mut_tbow_13.3f_trp53del_r482q_h&E_@10x.jpg]

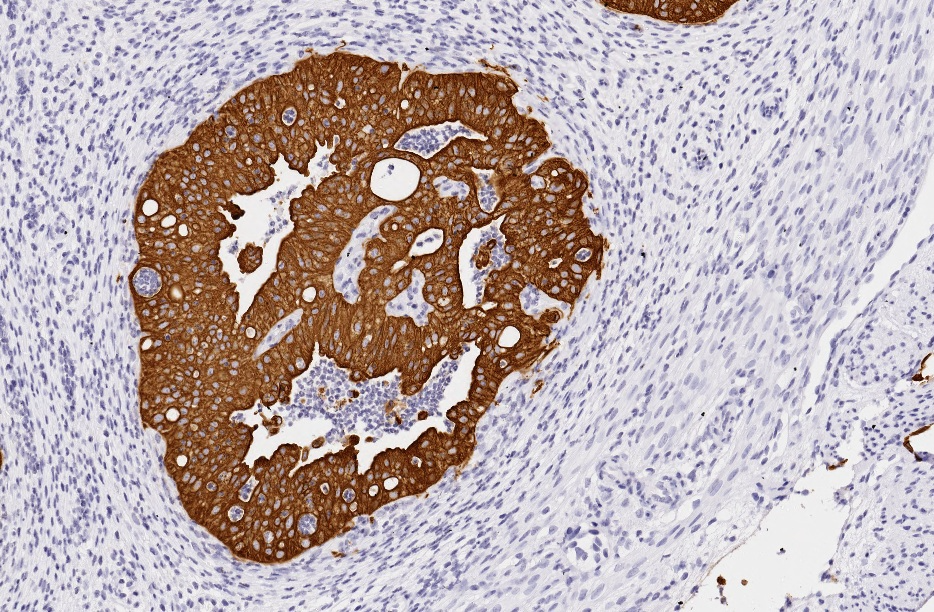

Supplement: Supplementary file 15 — Source Data for Figure 2 [file EMMM-15-e17094-s003.zip › EMM-2022-17094_source_data_figure_2/figure_2C/8 weeks/pten_tbnw_15.1f_pten_ck8_@10x.jpg]

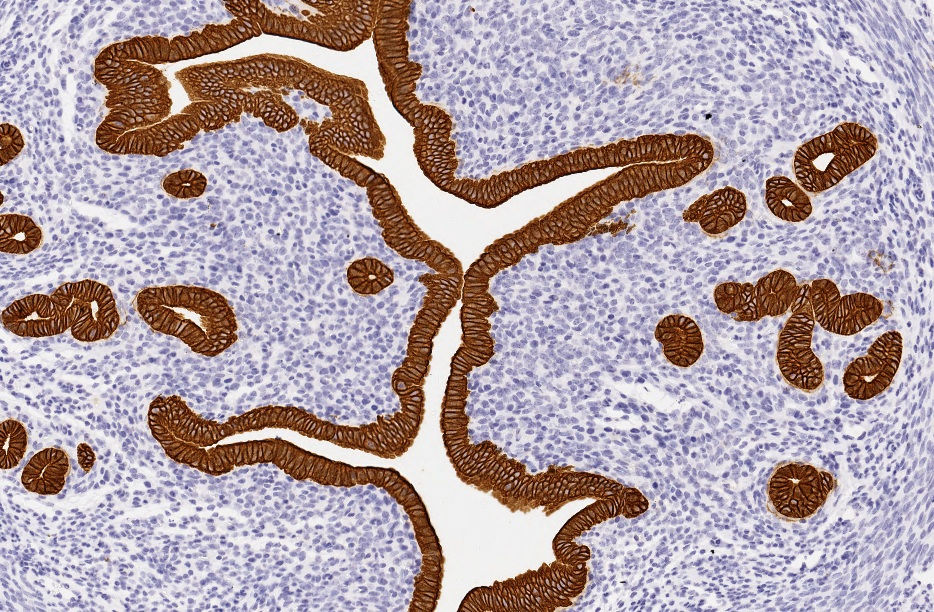

Supplement: Supplementary file 15 — Source Data for Figure 2 [file EMMM-15-e17094-s003.zip › EMM-2022-17094_source_data_figure_2/figure_2C/8 weeks/trp53del_tbow_13.3e_tp53del_ck8_@10x.jpg]

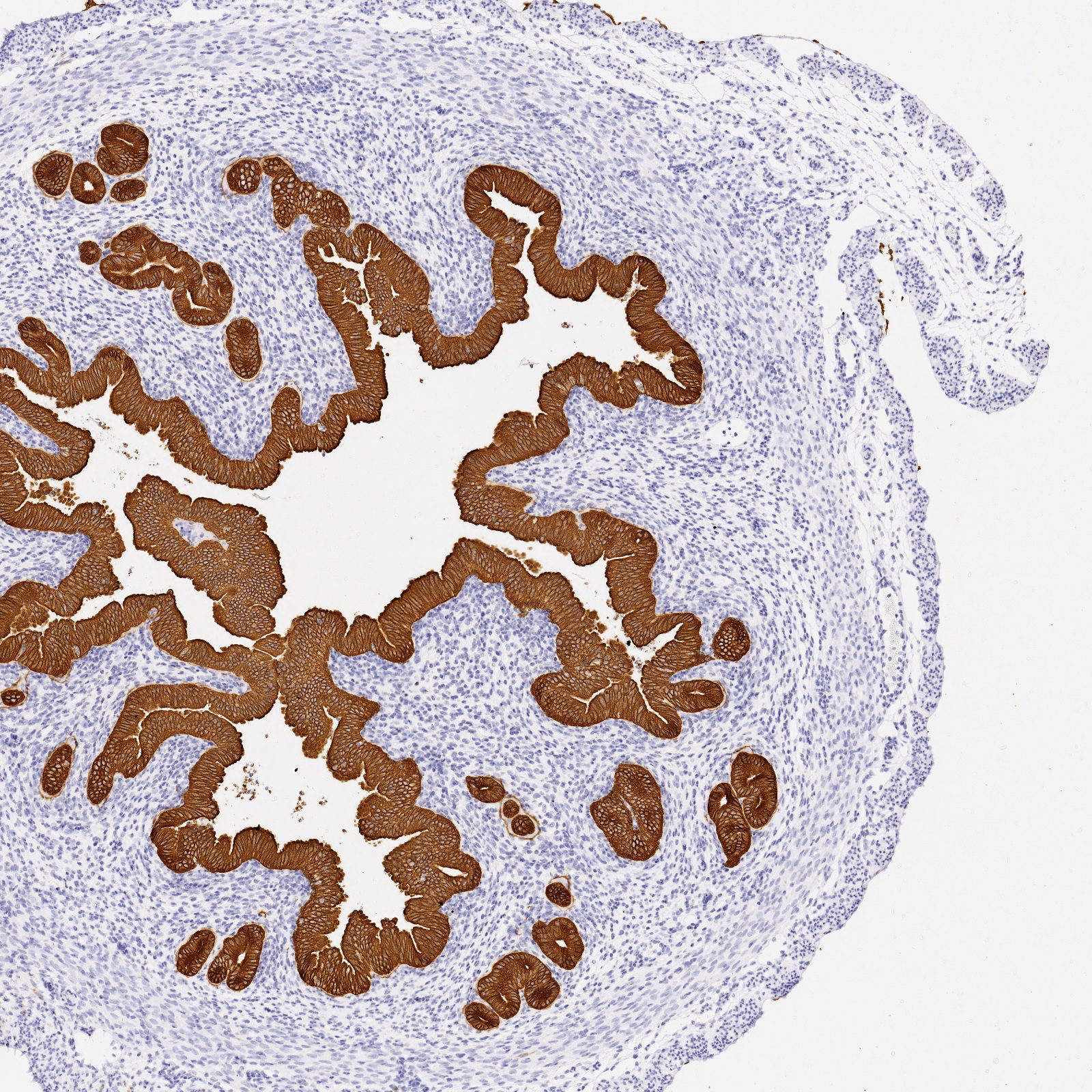

Supplement: Supplementary file 15 — Source Data for Figure 2 [file EMMM-15-e17094-s003.zip › EMM-2022-17094_source_data_figure_2/figure_2C/8 weeks/fbxw7mut_tbpw_8.2g_r482q_ck8_@10x.jpg]

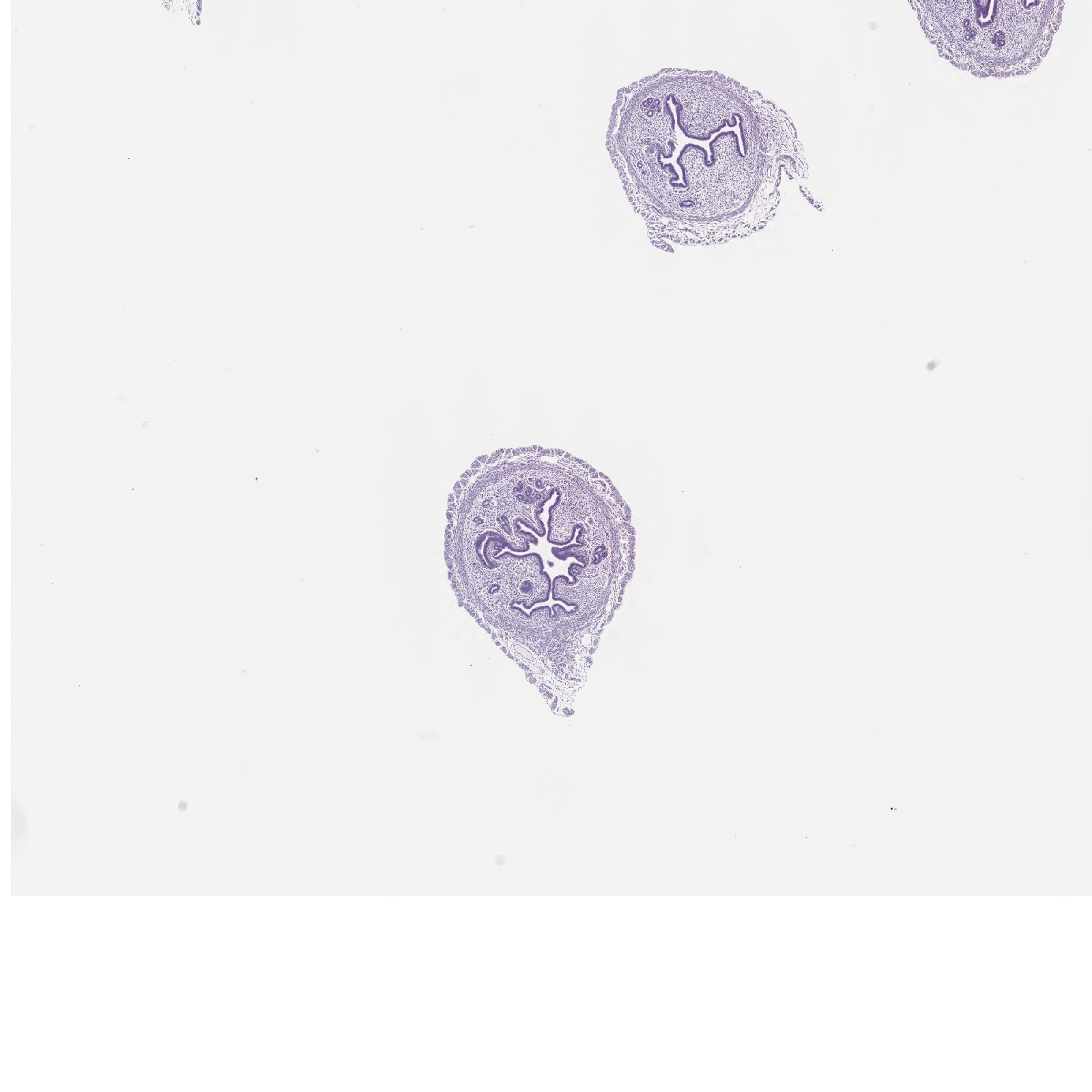

Supplement: Supplementary file 15 — Source Data for Figure 2 [file EMMM-15-e17094-s003.zip › EMM-2022-17094_source_data_figure_2/figure_2C/8 weeks/trp53mut_fbxw7mut_tprp_11.3i_r172h_r482q_he_2x.jpg]

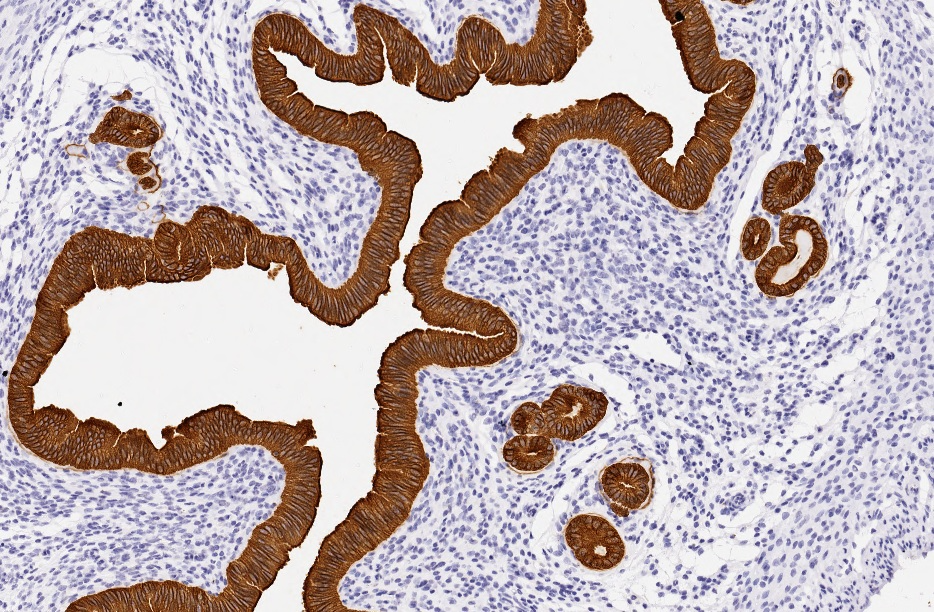

Supplement: Supplementary file 15 — Source Data for Figure 2 [file EMMM-15-e17094-s003.zip › EMM-2022-17094_source_data_figure_2/figure_2C/8 weeks/wt_tbpw_8.2f_wt_ck8_@10x.jpg]

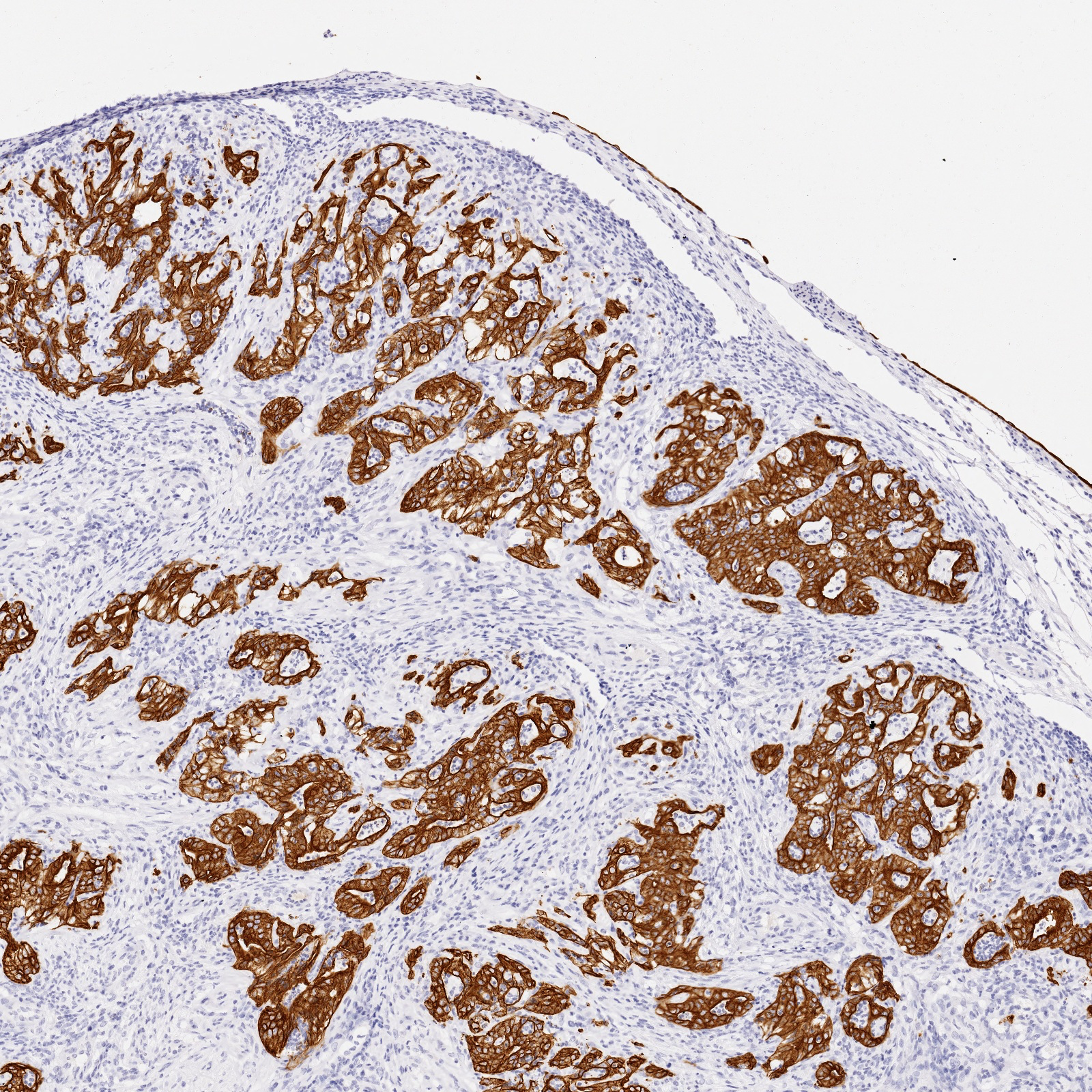

Supplement: Supplementary file 15 — Source Data for Figure 2 [file EMMM-15-e17094-s003.zip › EMM-2022-17094_source_data_figure_2/figure_2C/8 weeks/ptendel_fbxw7mut_tbnw_23_1d_ptendelr482q_ck_@10x.jpg]

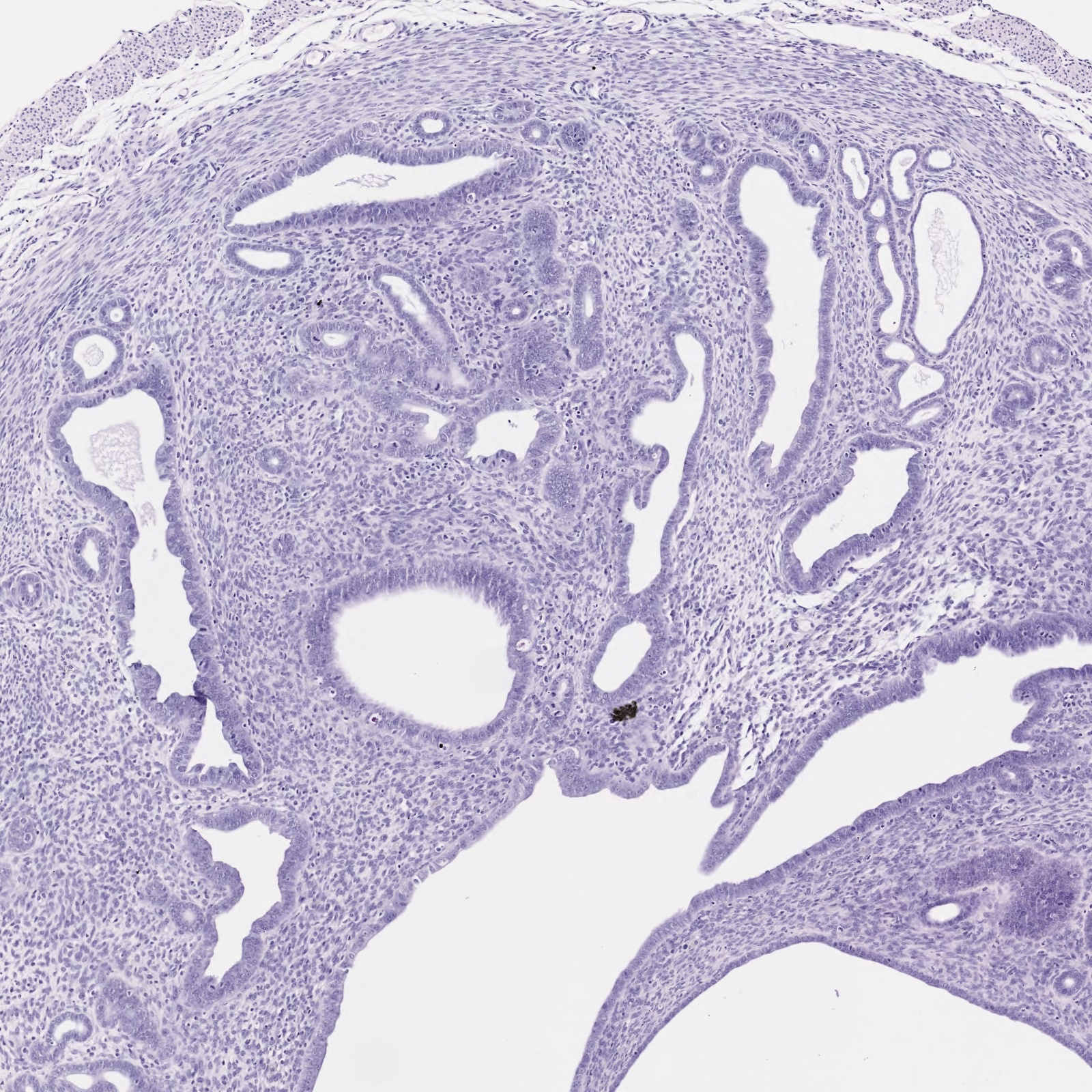

Supplement: Supplementary file 15 — Source Data for Figure 2 [file EMMM-15-e17094-s003.zip › EMM-2022-17094_source_data_figure_2/figure_2C/survival/trp53del_fbxw7mut_tbow_11.1b_trp53del_r482q_@survival_he_10x.jpg]

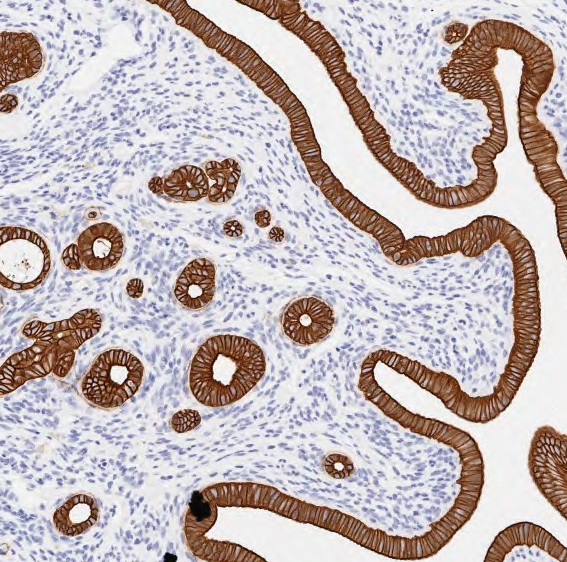

Supplement: Supplementary file 15 — Source Data for Figure 2 [file EMMM-15-e17094-s003.zip › EMM-2022-17094_source_data_figure_2/figure_2C/survival/fbxw7mut_tbpw_5.1e_r482q_@survival_ck_10x_cropped_200mm.png]

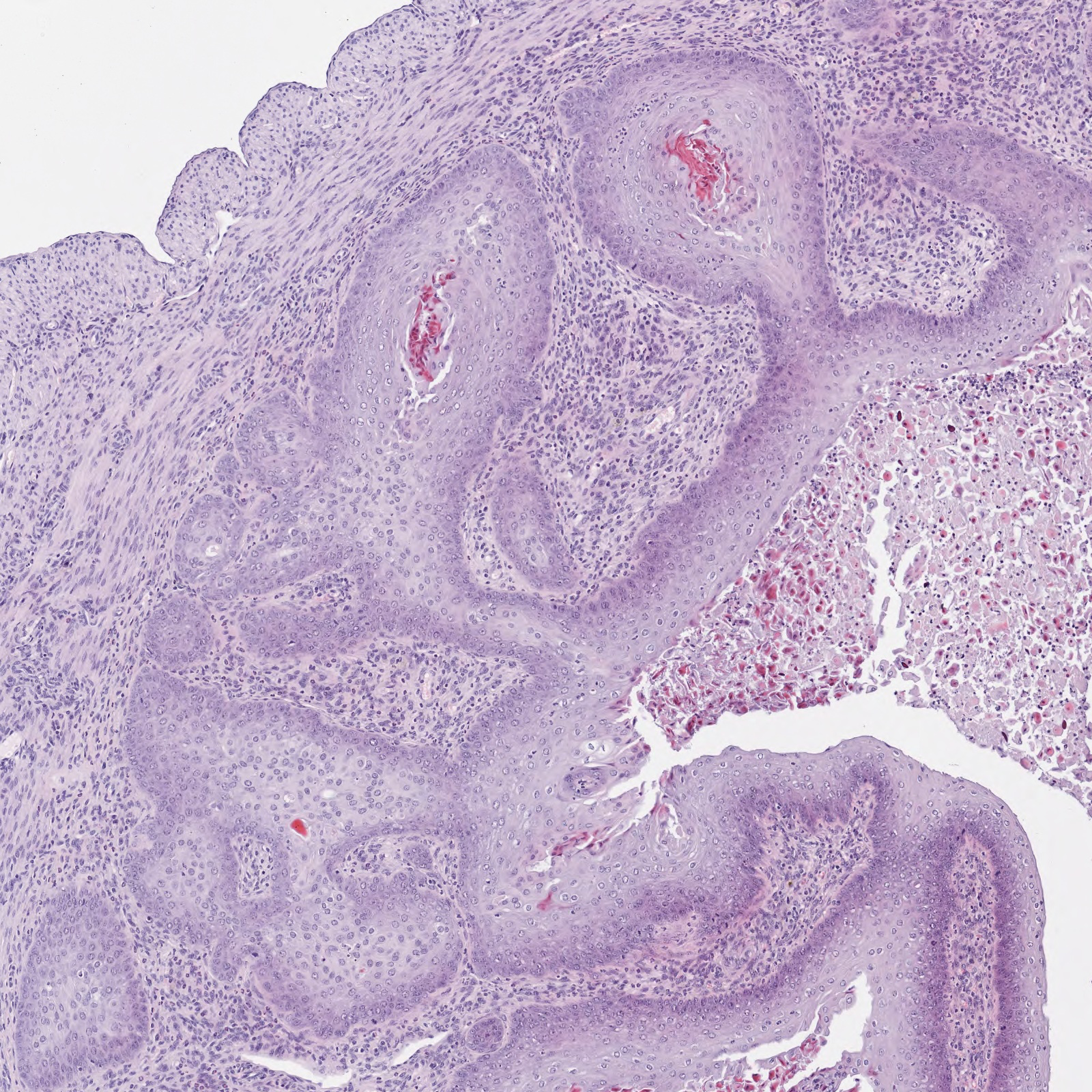

Supplement: Supplementary file 15 — Source Data for Figure 2 [file EMMM-15-e17094-s003.zip › EMM-2022-17094_source_data_figure_2/figure_2C/survival/ptendel_tbnw_1.2i_pten_@survival_he_10x.jpg]

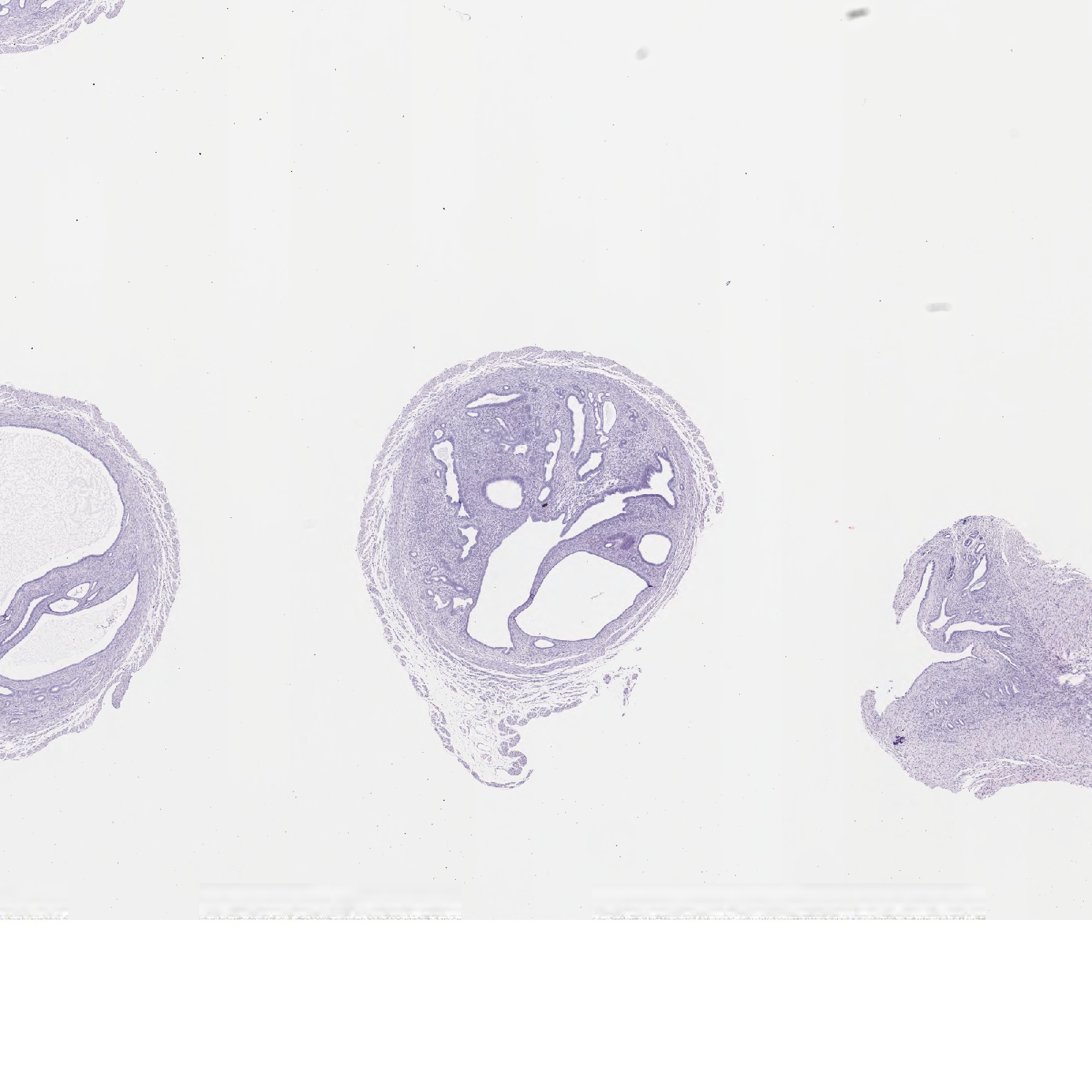

Supplement: Supplementary file 15 — Source Data for Figure 2 [file EMMM-15-e17094-s003.zip › EMM-2022-17094_source_data_figure_2/figure_2C/survival/trp53del_fbxw7mut_tbow_11.1b_trp53del_r482q_@survival_he_2x.jpg]

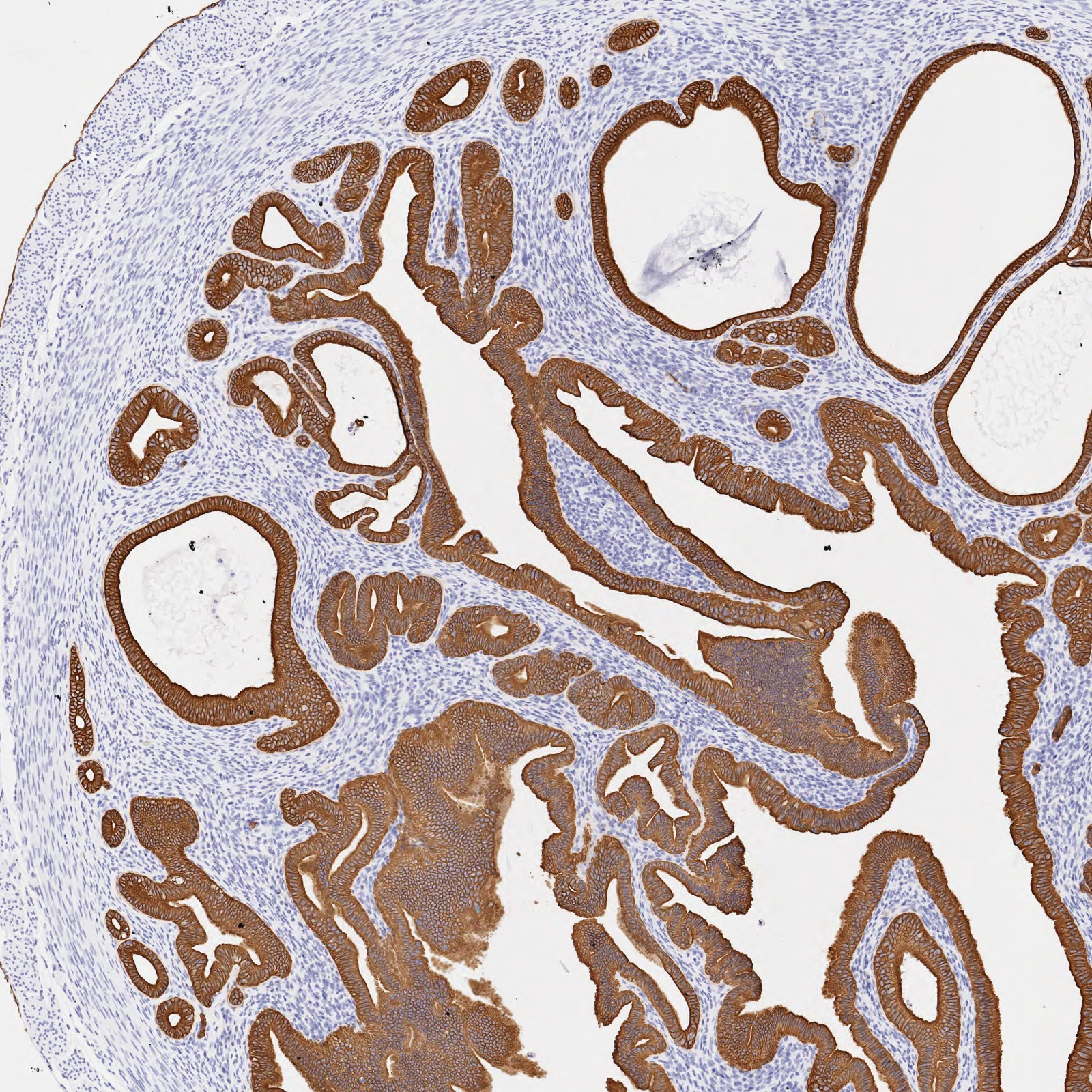

Supplement: Supplementary file 15 — Source Data for Figure 2 [file EMMM-15-e17094-s003.zip › EMM-2022-17094_source_data_figure_2/figure_2C/survival/trp53del_tbow_11.1a_trp53del_@survival_ck_10x.jpg]

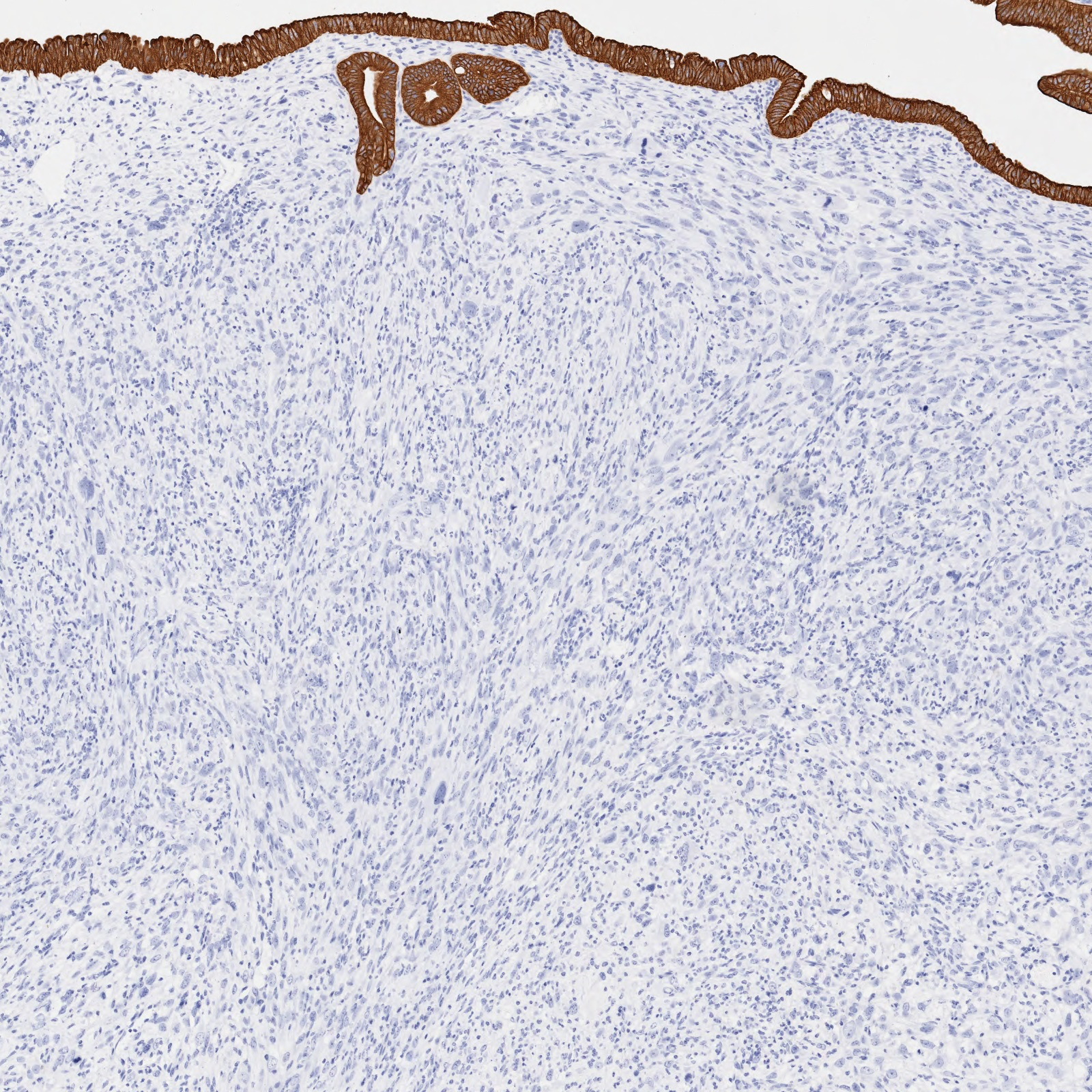

Supplement: Supplementary file 15 — Source Data for Figure 2 [file EMMM-15-e17094-s003.zip › EMM-2022-17094_source_data_figure_2/figure_2C/survival/trp53del_fbxw7mut_tprp_5.2h_r172h_r482q_@survival_ck_10x.jpg]

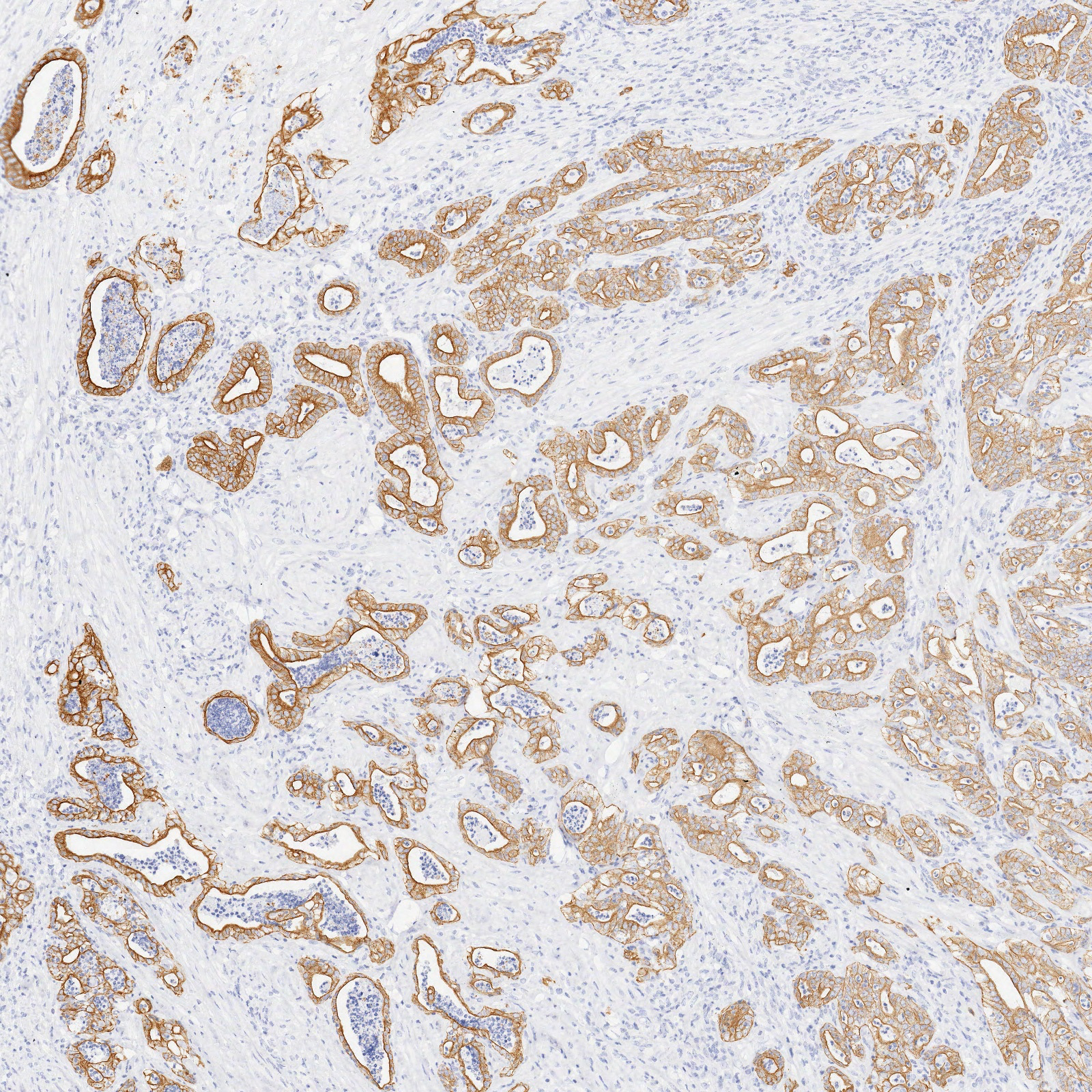

Supplement: Supplementary file 15 — Source Data for Figure 2 [file EMMM-15-e17094-s003.zip › EMM-2022-17094_source_data_figure_2/figure_2C/survival/ptendel_fbxw7mut_tbnw_1.2g_pten_r482q_@survival_ck_10x.jpg]

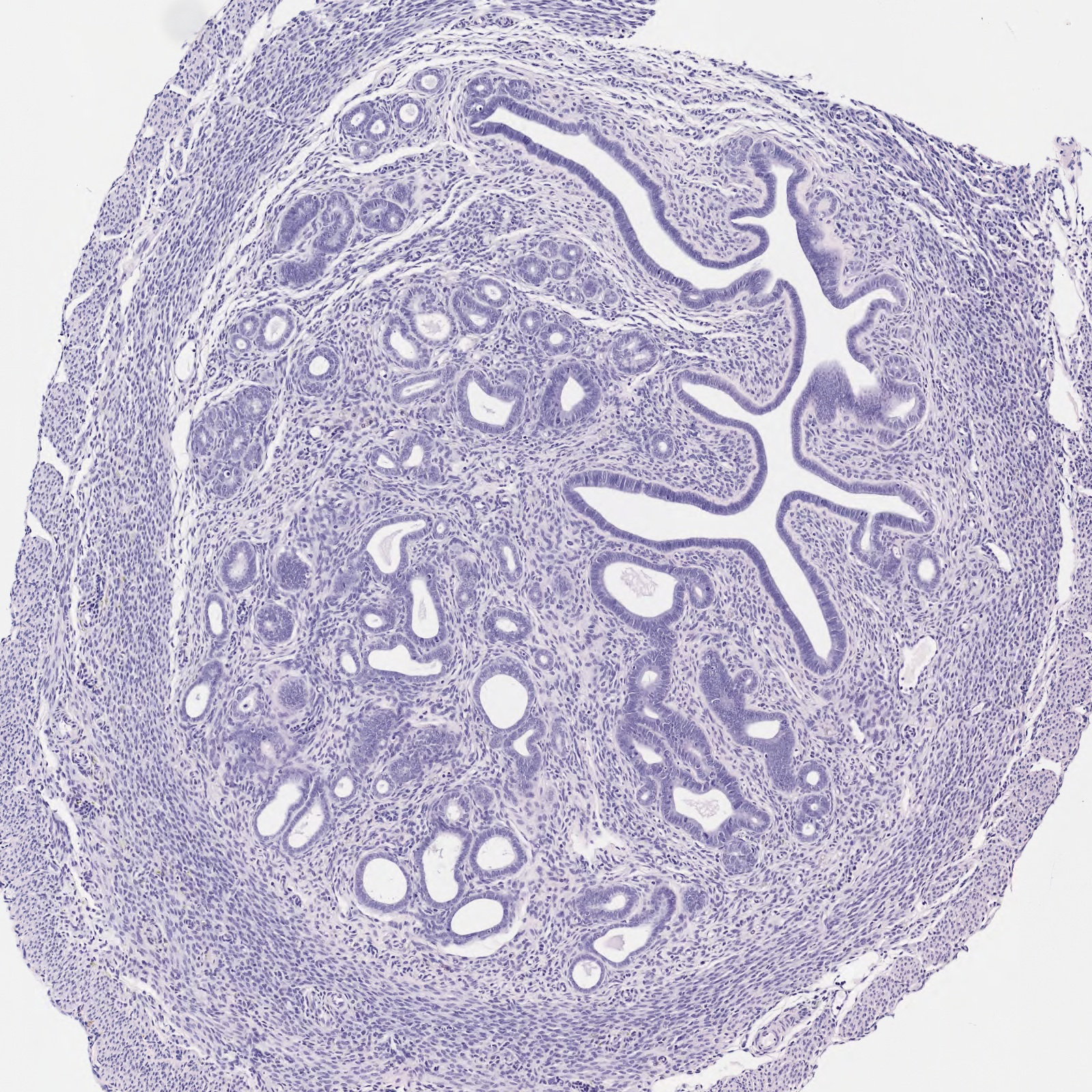

Supplement: Supplementary file 15 — Source Data for Figure 2 [file EMMM-15-e17094-s003.zip › EMM-2022-17094_source_data_figure_2/figure_2C/survival/fbxw7mut_tbpw_5.1e_r482q_@survival_he_10x.jpg]

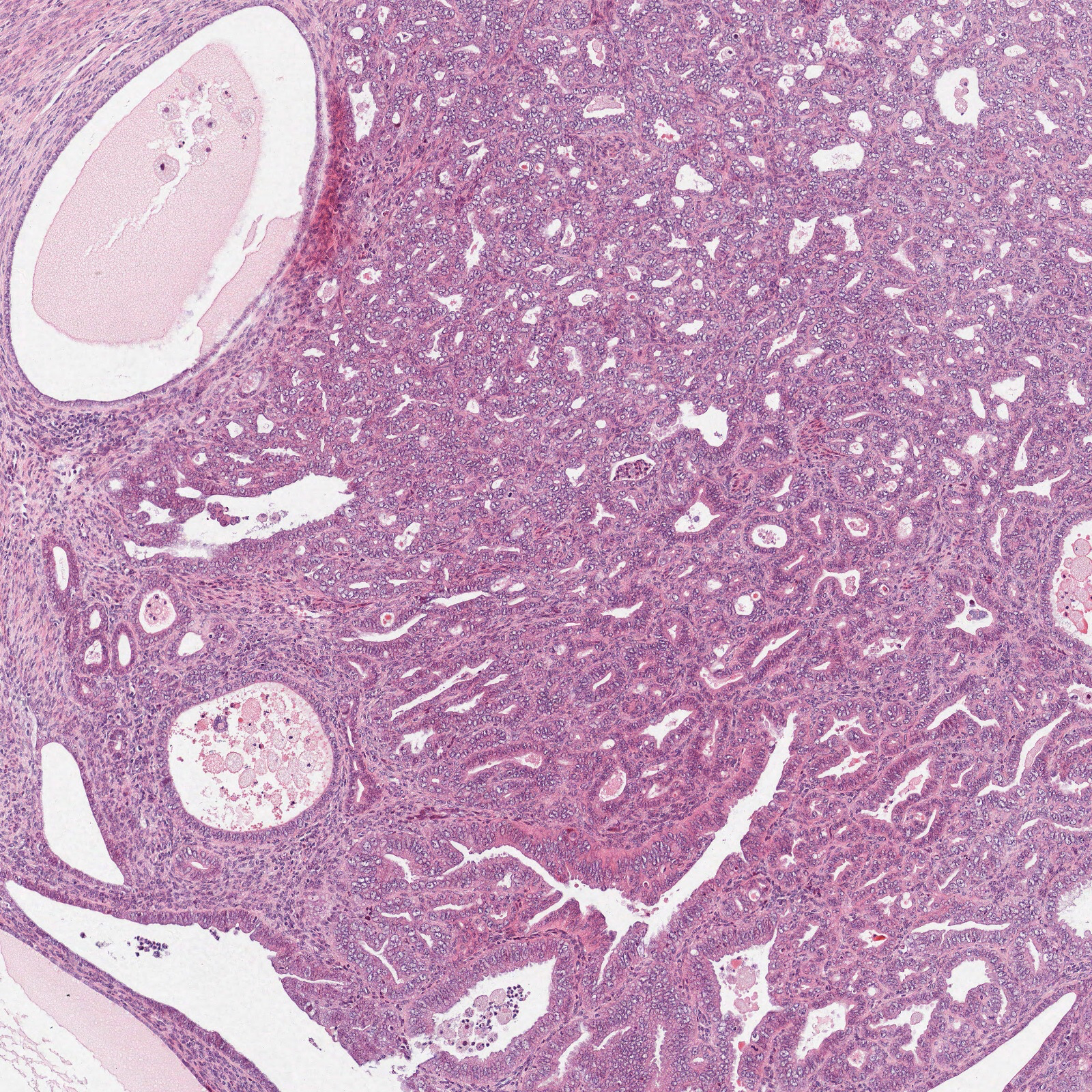

Supplement: Supplementary file 15 — Source Data for Figure 2 [file EMMM-15-e17094-s003.zip › EMM-2022-17094_source_data_figure_2/figure_2C/survival/trp53mut_tprp_1.3d_r172h_@survival_he_10x.jpg]

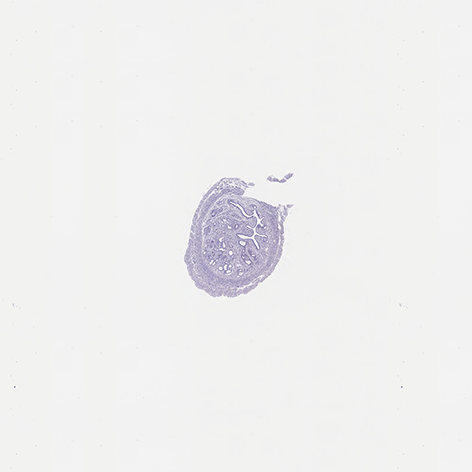

Supplement: Supplementary file 15 — Source Data for Figure 2 [file EMMM-15-e17094-s003.zip › EMM-2022-17094_source_data_figure_2/figure_2C/survival/fbxw7mut_tbpw_5.1e_r482q_@survival_he_2x_20mm_@600dpi.jpg]

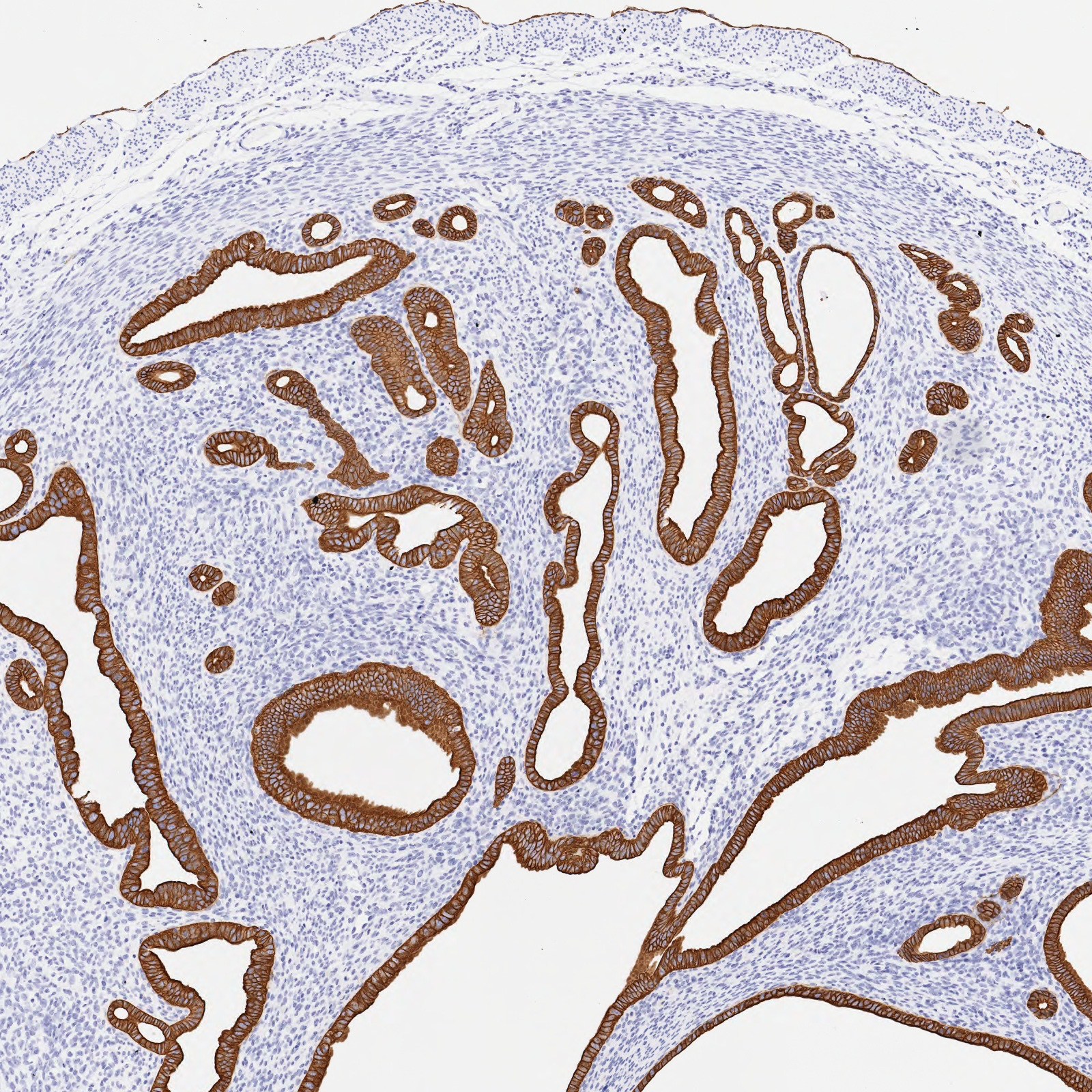

Supplement: Supplementary file 15 — Source Data for Figure 2 [file EMMM-15-e17094-s003.zip › EMM-2022-17094_source_data_figure_2/figure_2C/survival/trp53del_fbxw7mut_tbow_11.1b_trp53del_r482q_@survival_ck_10x.jpg]

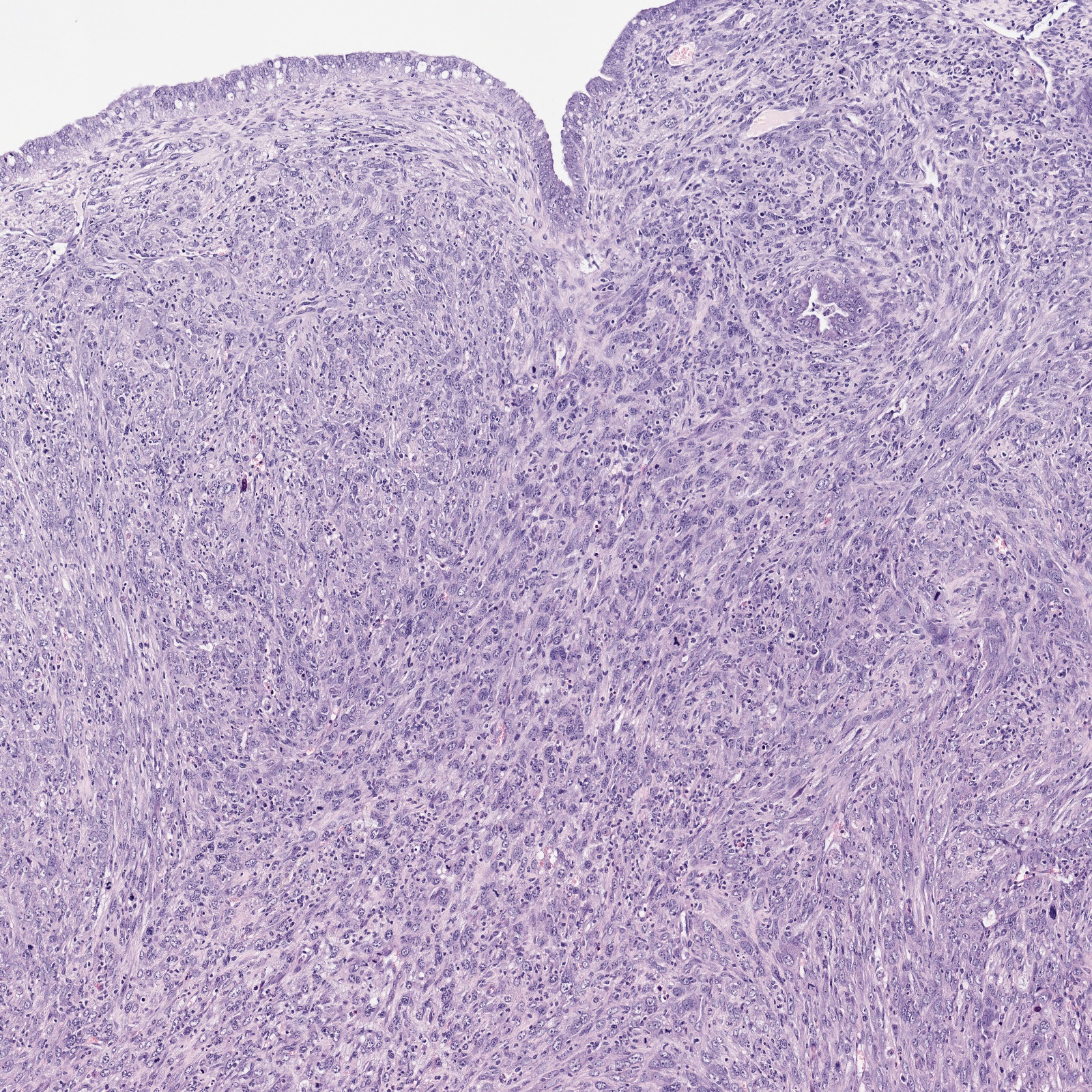

Supplement: Supplementary file 15 — Source Data for Figure 2 [file EMMM-15-e17094-s003.zip › EMM-2022-17094_source_data_figure_2/figure_2C/survival/trp53del_fbxw7mut_tprp_5.2h_r172h_r482q_@survival_he_10x.jpg]

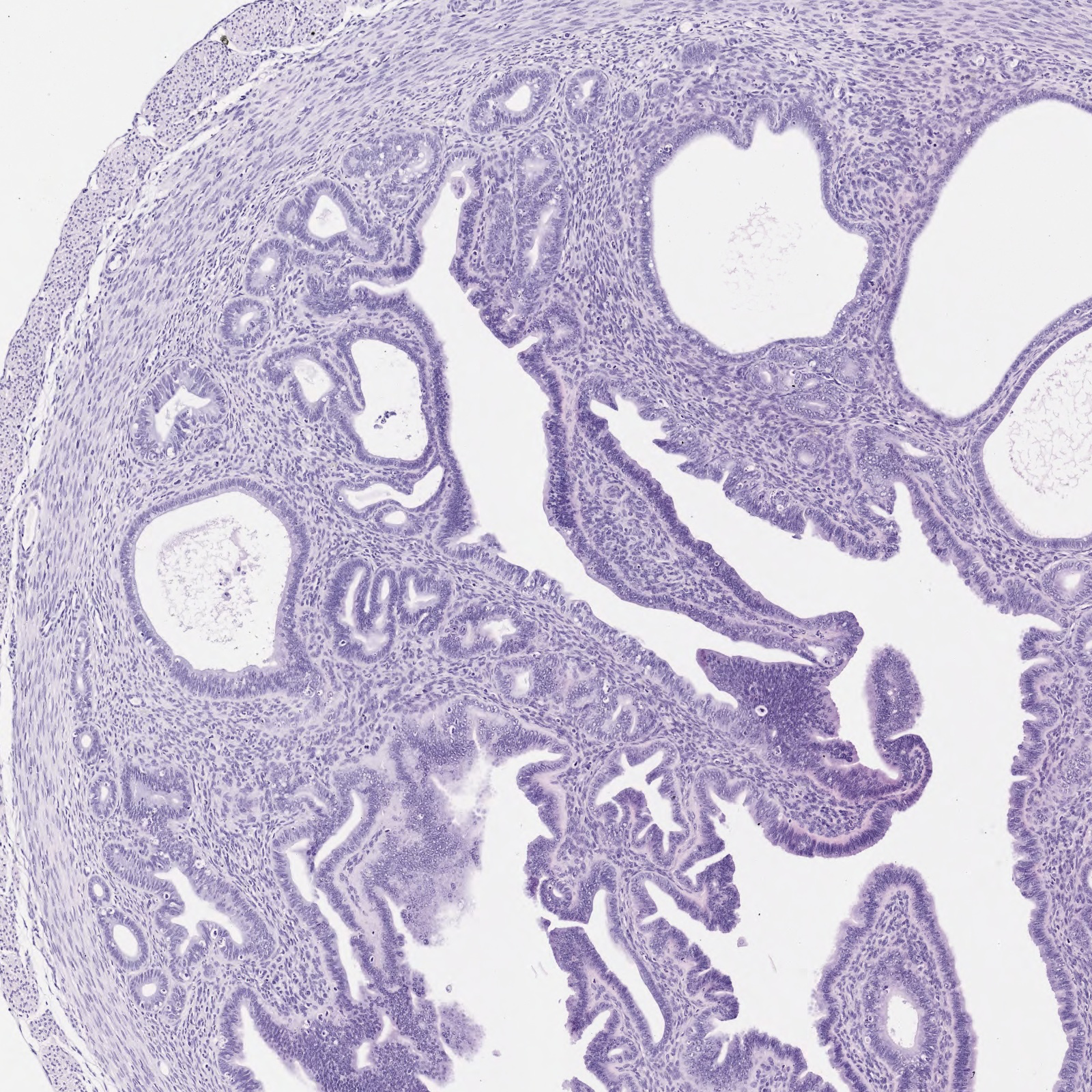

Supplement: Supplementary file 15 — Source Data for Figure 2 [file EMMM-15-e17094-s003.zip › EMM-2022-17094_source_data_figure_2/figure_2C/survival/trp53del_tbow_11.1a_trp53del_@survival_he_10x.jpg]

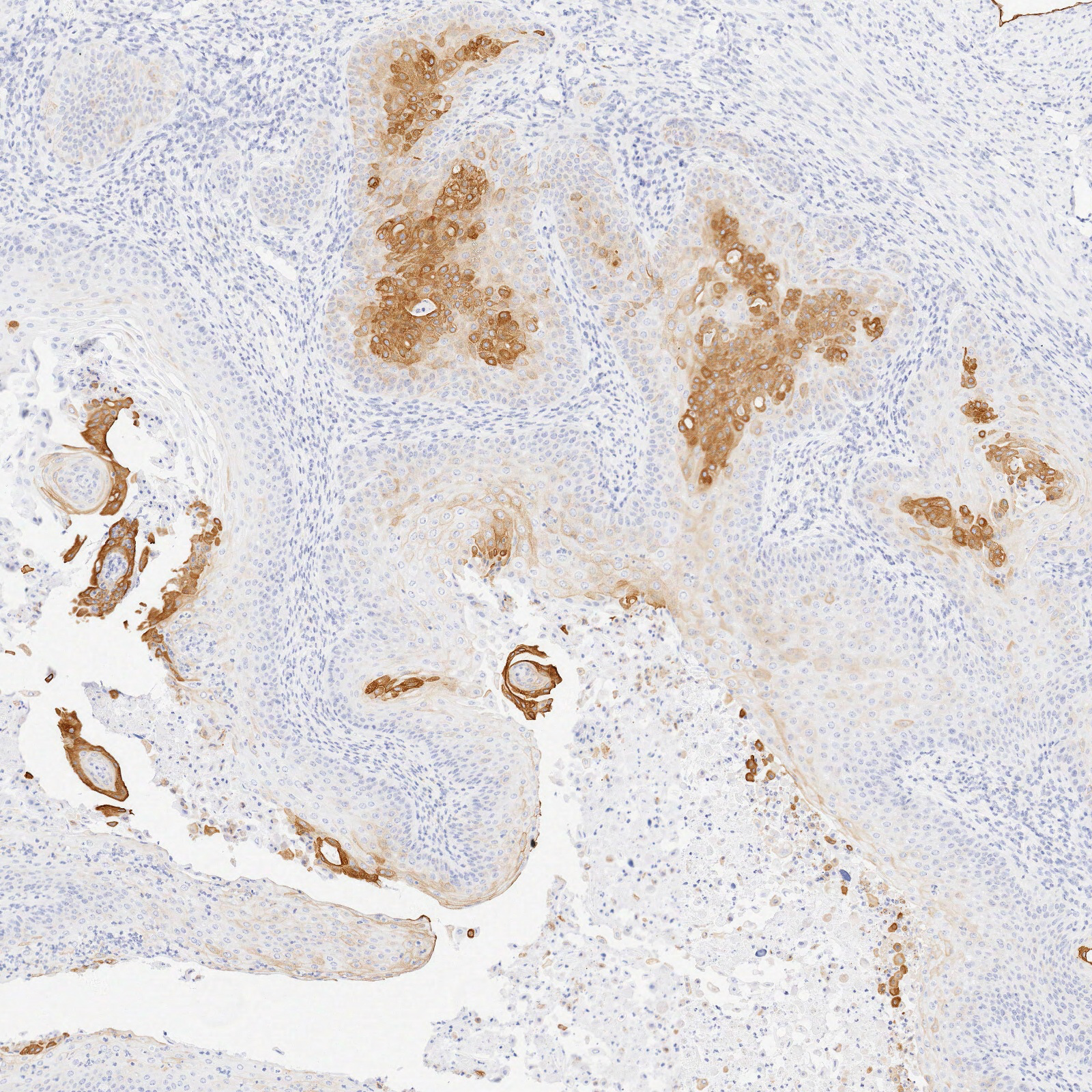

Supplement: Supplementary file 15 — Source Data for Figure 2 [file EMMM-15-e17094-s003.zip › EMM-2022-17094_source_data_figure_2/figure_2C/survival/ptendel_tbnw_1.2i_pten_@survival_ck_10x.jpg]

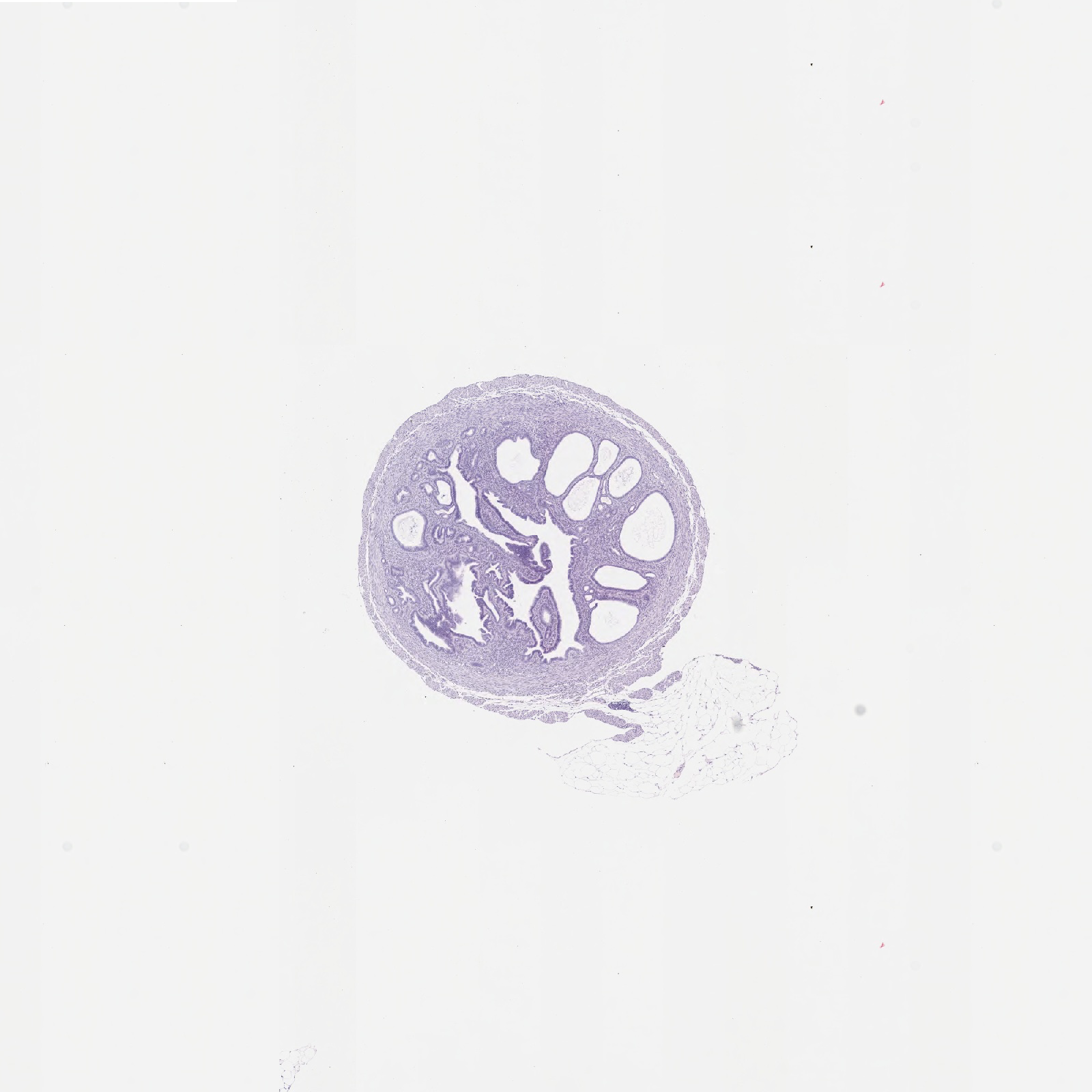

Supplement: Supplementary file 15 — Source Data for Figure 2 [file EMMM-15-e17094-s003.zip › EMM-2022-17094_source_data_figure_2/figure_2C/survival/trp53del_tbow_11.1a_trp53del_@survival_he_2x.jpg]

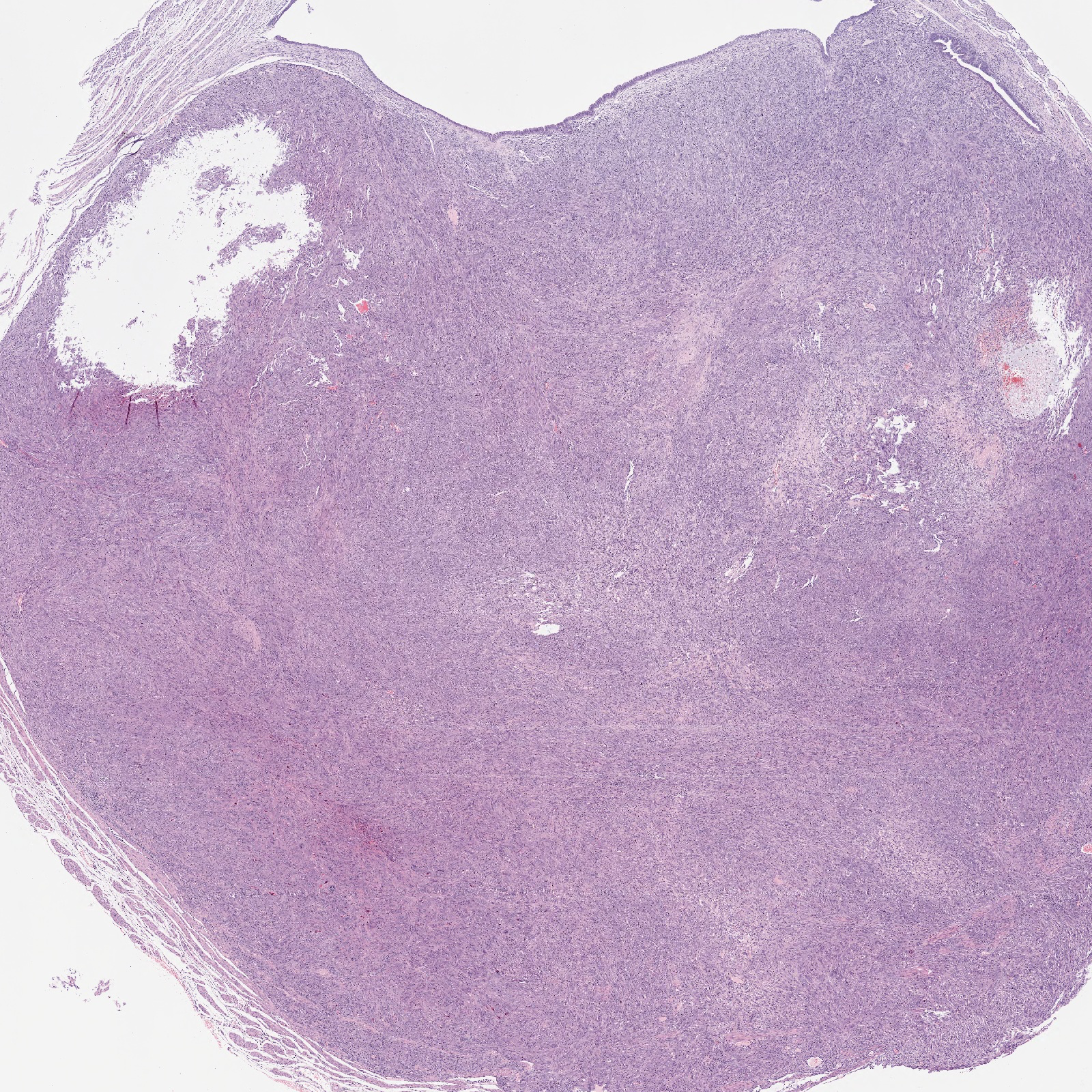

Supplement: Supplementary file 15 — Source Data for Figure 2 [file EMMM-15-e17094-s003.zip › EMM-2022-17094_source_data_figure_2/figure_2C/survival/trp53del_fbxw7mut_tprp_5.2h_r172h_r482q_@survival_he_2x.jpg]

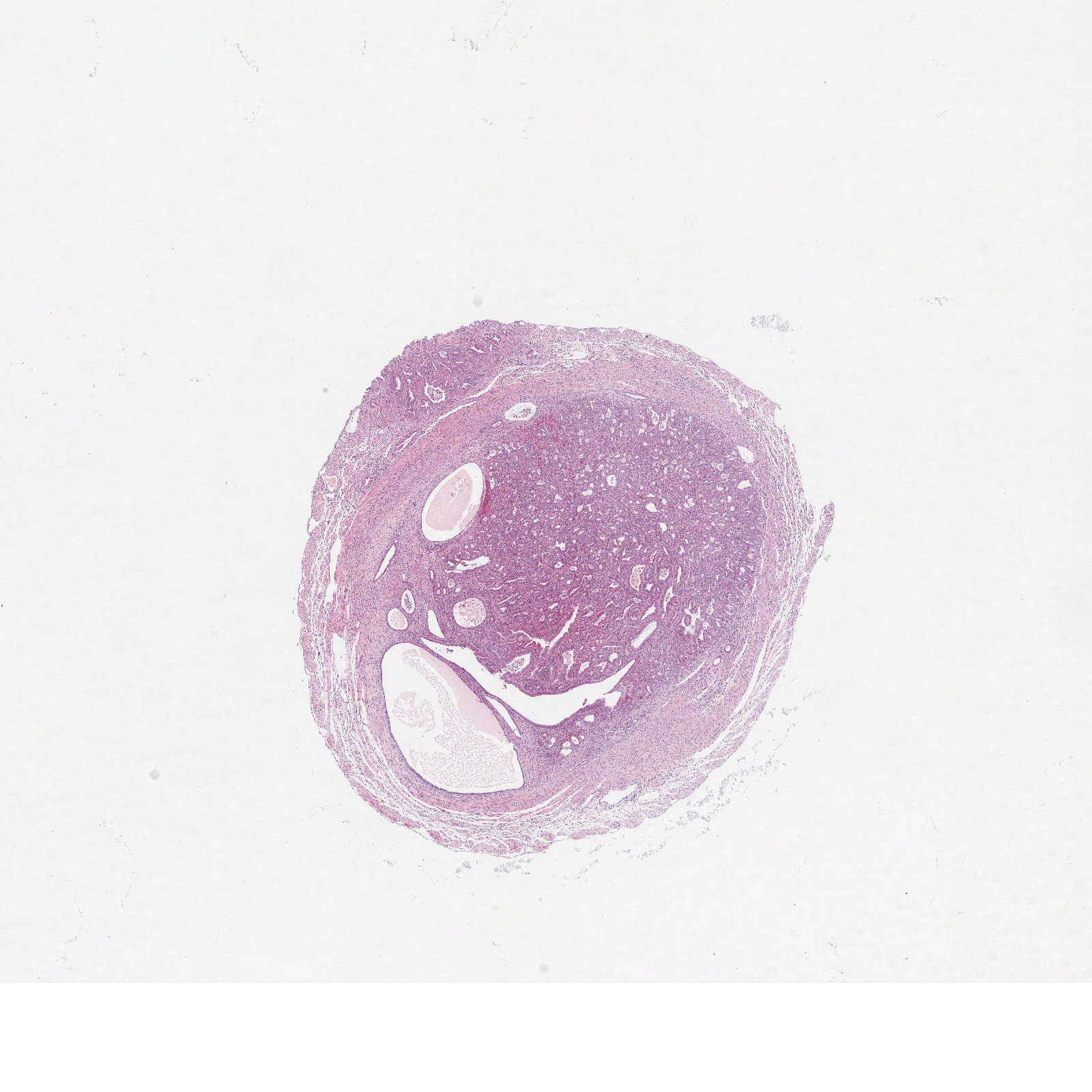

Supplement: Supplementary file 15 — Source Data for Figure 2 [file EMMM-15-e17094-s003.zip › EMM-2022-17094_source_data_figure_2/figure_2C/survival/trp53mut_tprp_1.3d_r172h_@survival_he_2x.jpg]

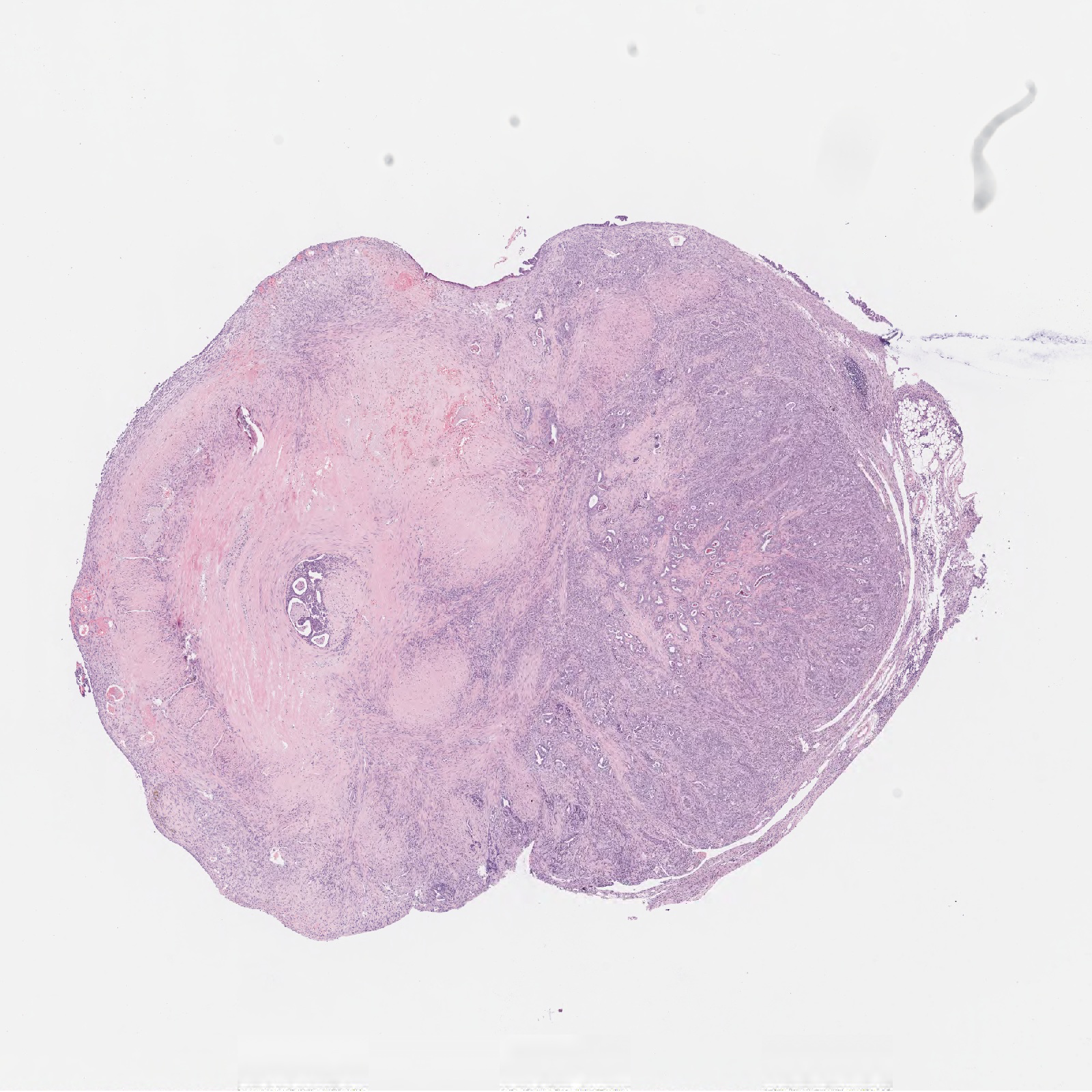

Supplement: Supplementary file 15 — Source Data for Figure 2 [file EMMM-15-e17094-s003.zip › EMM-2022-17094_source_data_figure_2/figure_2C/survival/ptendel_fbxw7mut_tbnw_1.2g_pten_r482q_@survival_he_2x.jpg]

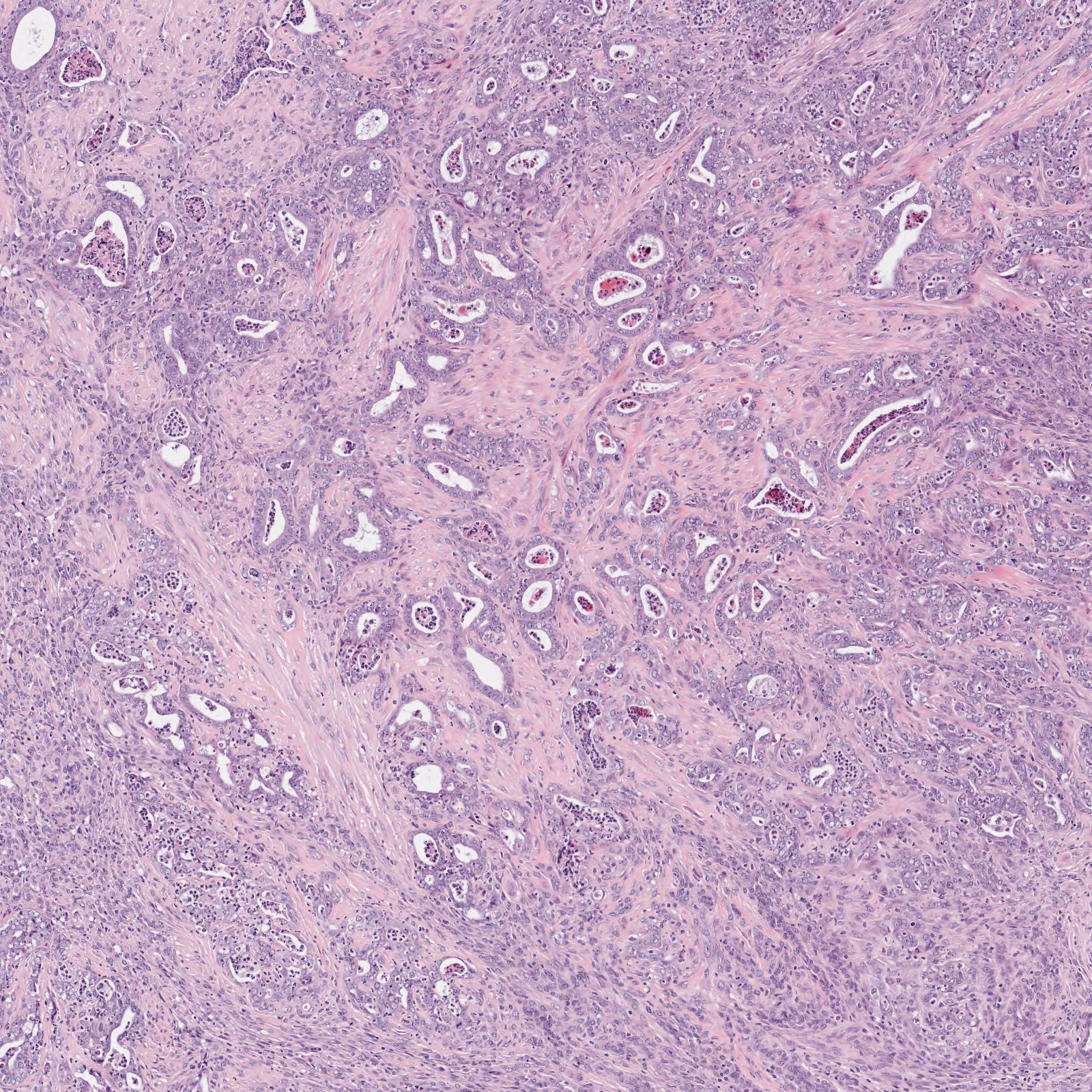

Supplement: Supplementary file 15 — Source Data for Figure 2 [file EMMM-15-e17094-s003.zip › EMM-2022-17094_source_data_figure_2/figure_2C/survival/ptendel_fbxw7mut_tbnw_1.2g_pten_r482q_@survival_he_10x.jpg]

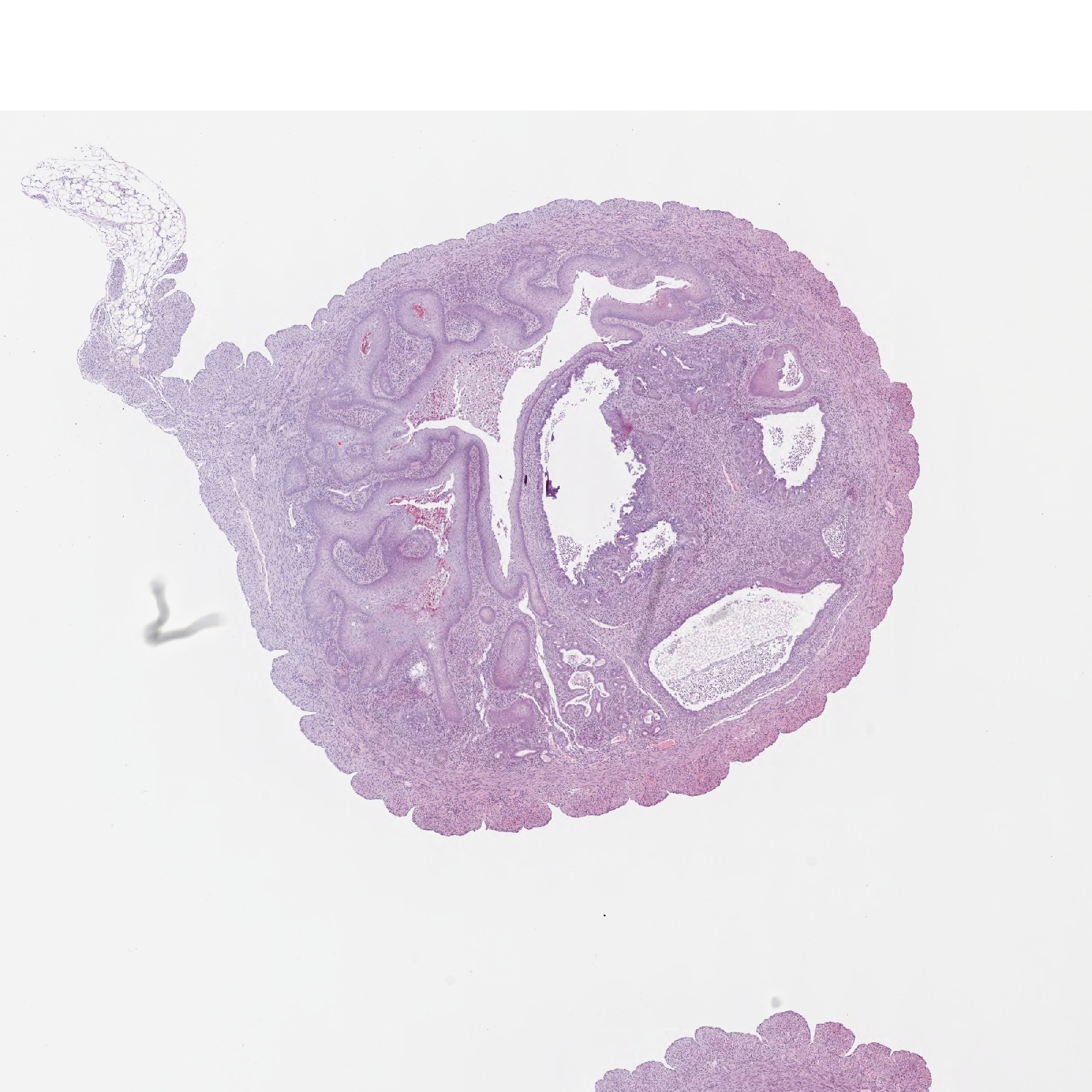

Supplement: Supplementary file 15 — Source Data for Figure 2 [file EMMM-15-e17094-s003.zip › EMM-2022-17094_source_data_figure_2/figure_2C/survival/ptendel_tbnw_1.2i_pten_@survival_he_2x.jpg]

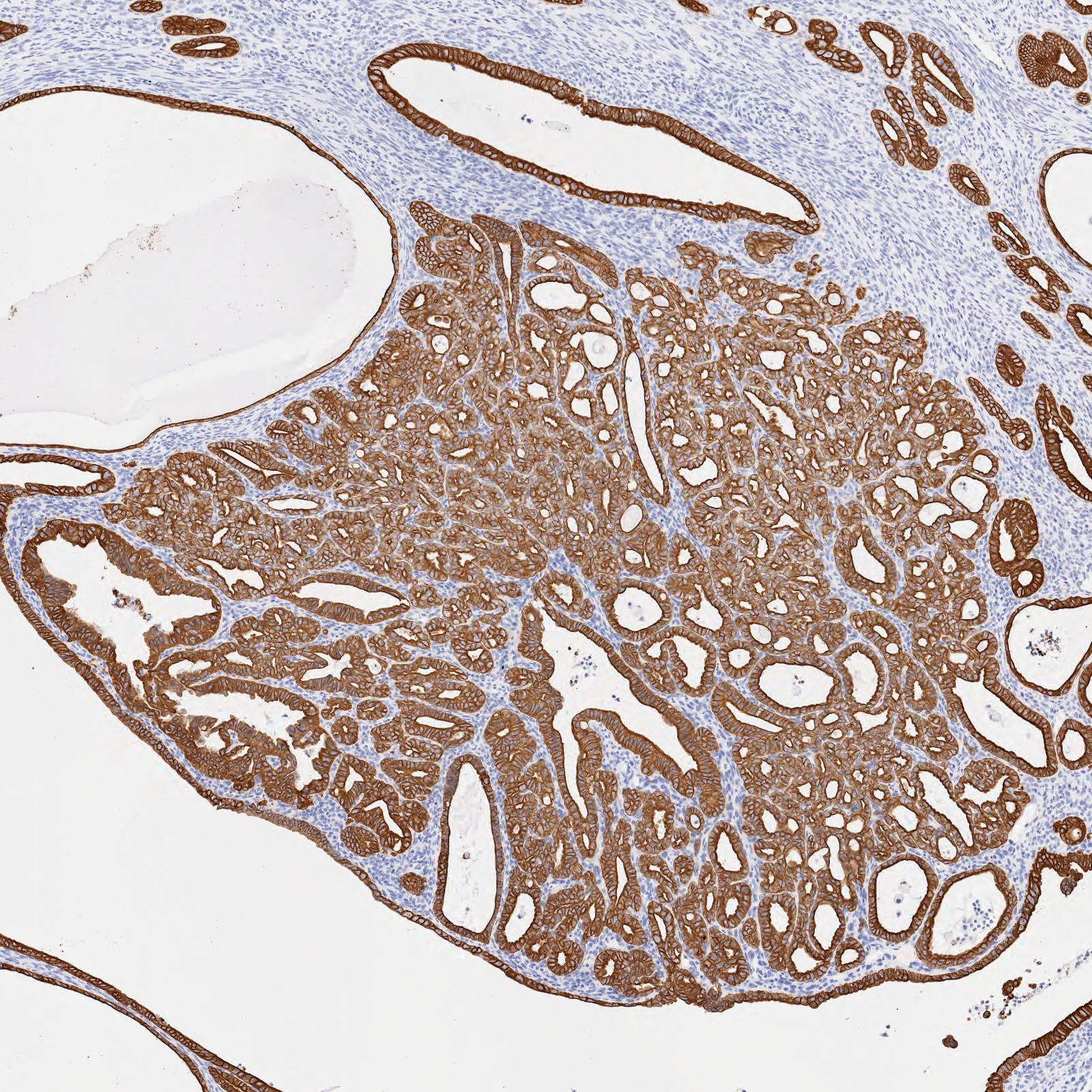

Supplement: Supplementary file 15 — Source Data for Figure 2 [file EMMM-15-e17094-s003.zip › EMM-2022-17094_source_data_figure_2/figure_2C/survival/trp53mut_tprp_1.3d_r172h_@survival_ck_10x.jpg]

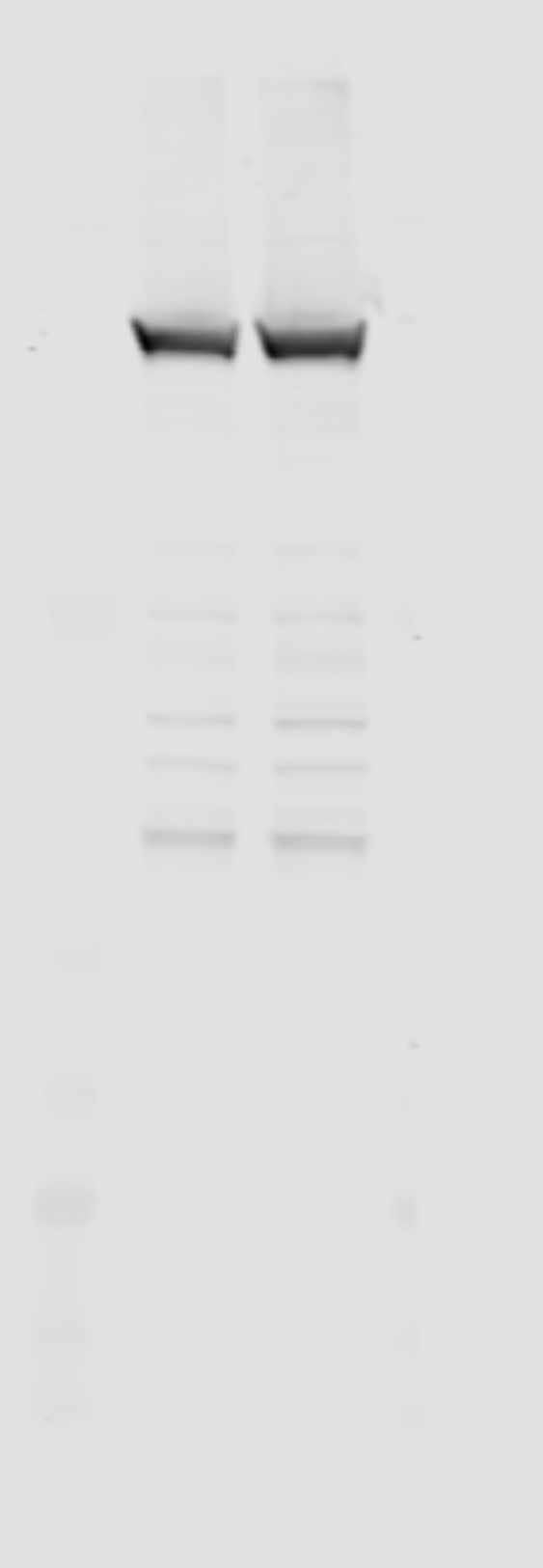

Supplement: Supplementary file 18 — Source Data for Figure 5 [file EMMM-15-e17094-s001.zip › EMM-2022-17094_source_data_figure_5/figure_5D/Myc-tag Input.tif]

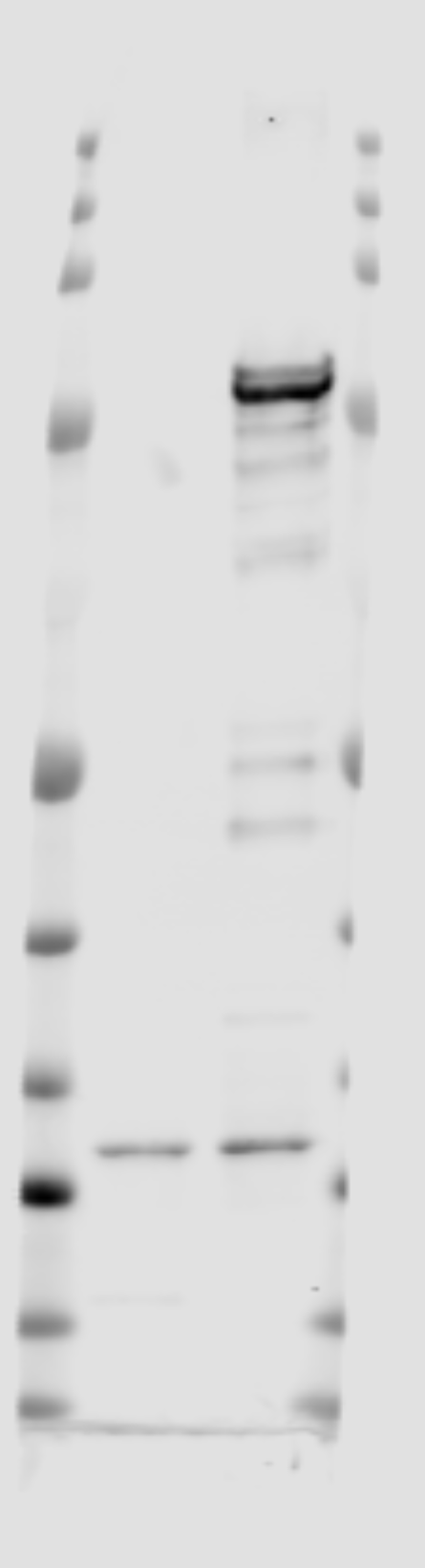

Supplement: Supplementary file 18 — Source Data for Figure 5 [file EMMM-15-e17094-s001.zip › EMM-2022-17094_source_data_figure_5/figure_5D/V5-tag IP.tif]

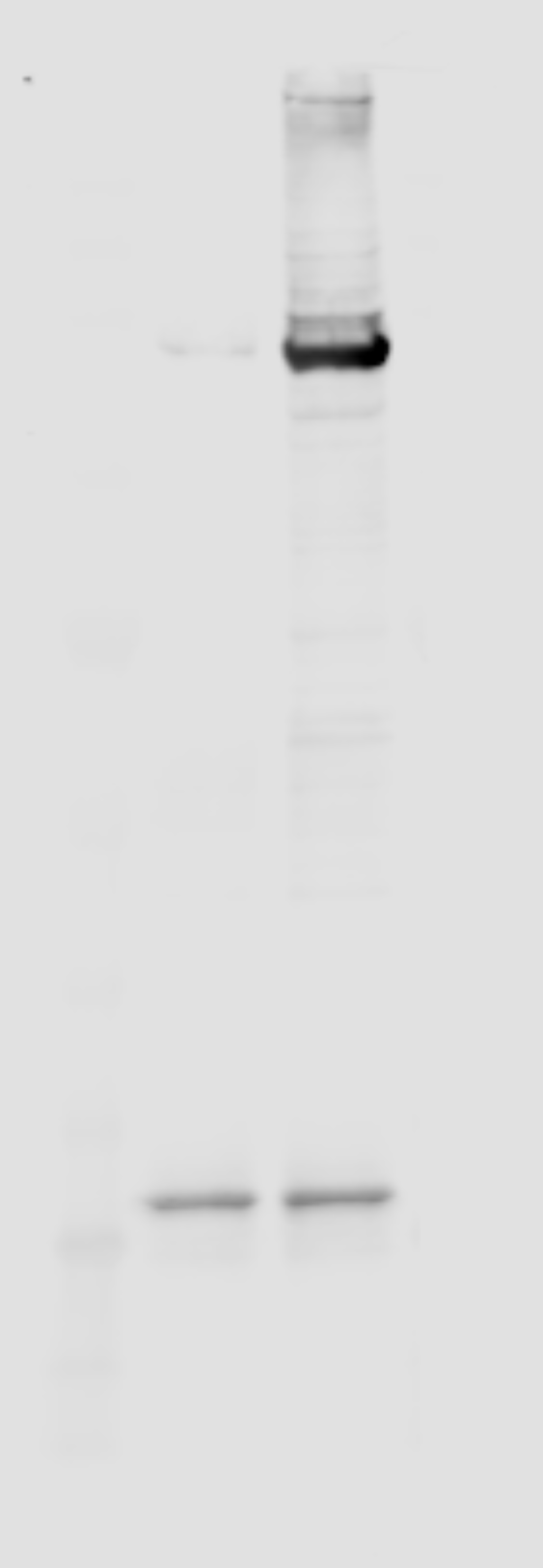

Supplement: Supplementary file 18 — Source Data for Figure 5 [file EMMM-15-e17094-s001.zip › EMM-2022-17094_source_data_figure_5/figure_5D/Myc-tag IP.tif]

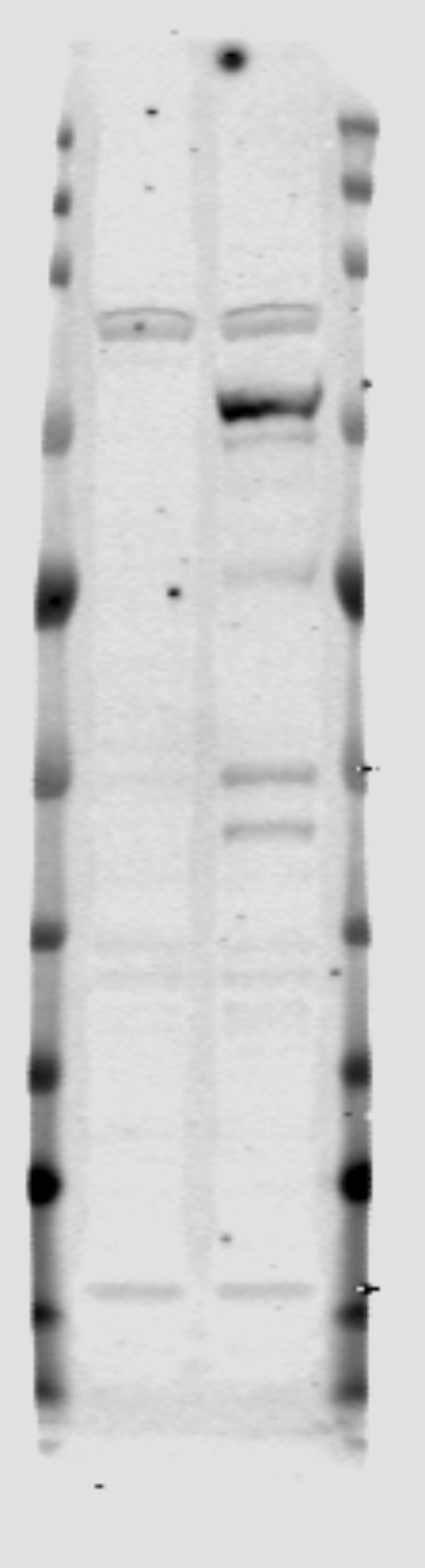

Supplement: Supplementary file 18 — Source Data for Figure 5 [file EMMM-15-e17094-s001.zip › EMM-2022-17094_source_data_figure_5/figure_5D/V5-tag Input.tif]

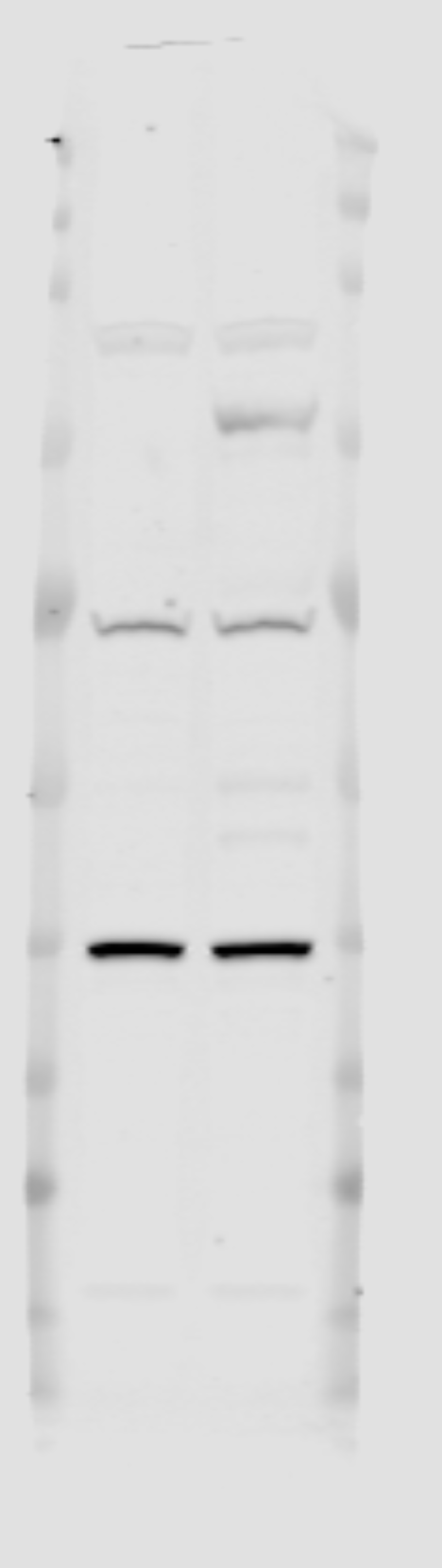

Supplement: Supplementary file 18 — Source Data for Figure 5 [file EMMM-15-e17094-s001.zip › EMM-2022-17094_source_data_figure_5/figure_5D/GAPDH Input.tif]

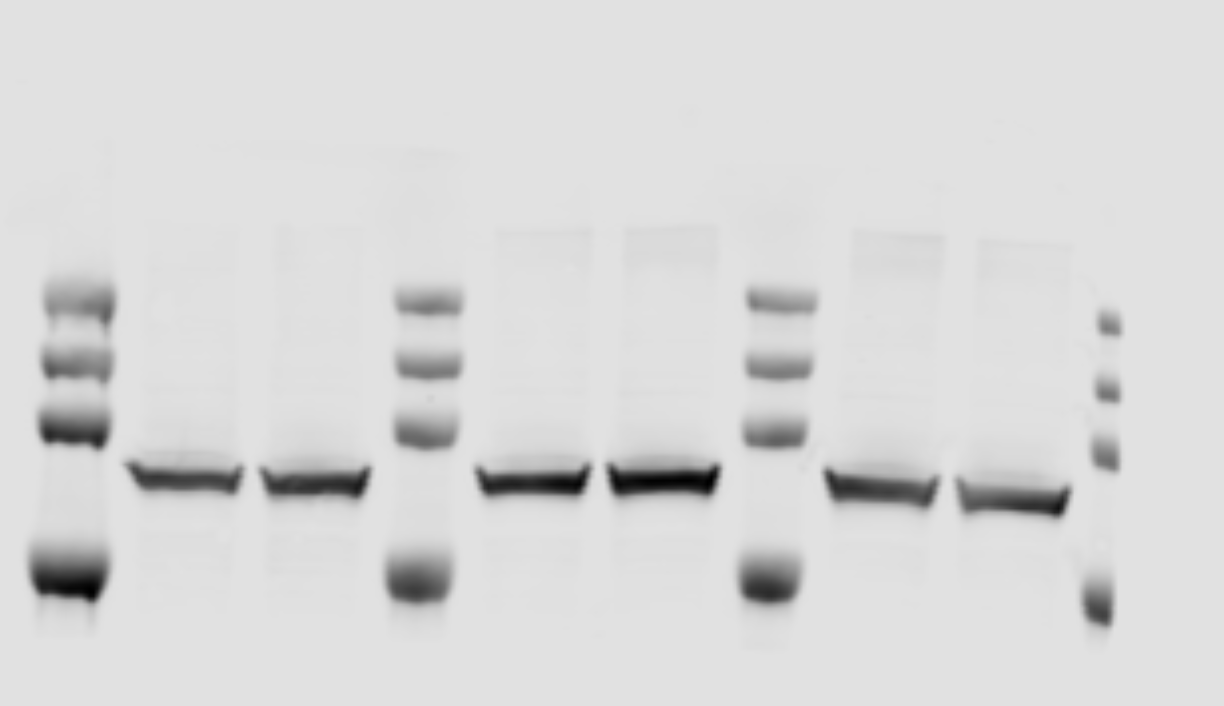

Supplement: Supplementary file 18 — Source Data for Figure 5 [file EMMM-15-e17094-s001.zip › EMM-2022-17094_source_data_figure_5/figure_5C/Myc-tag Input.tif]

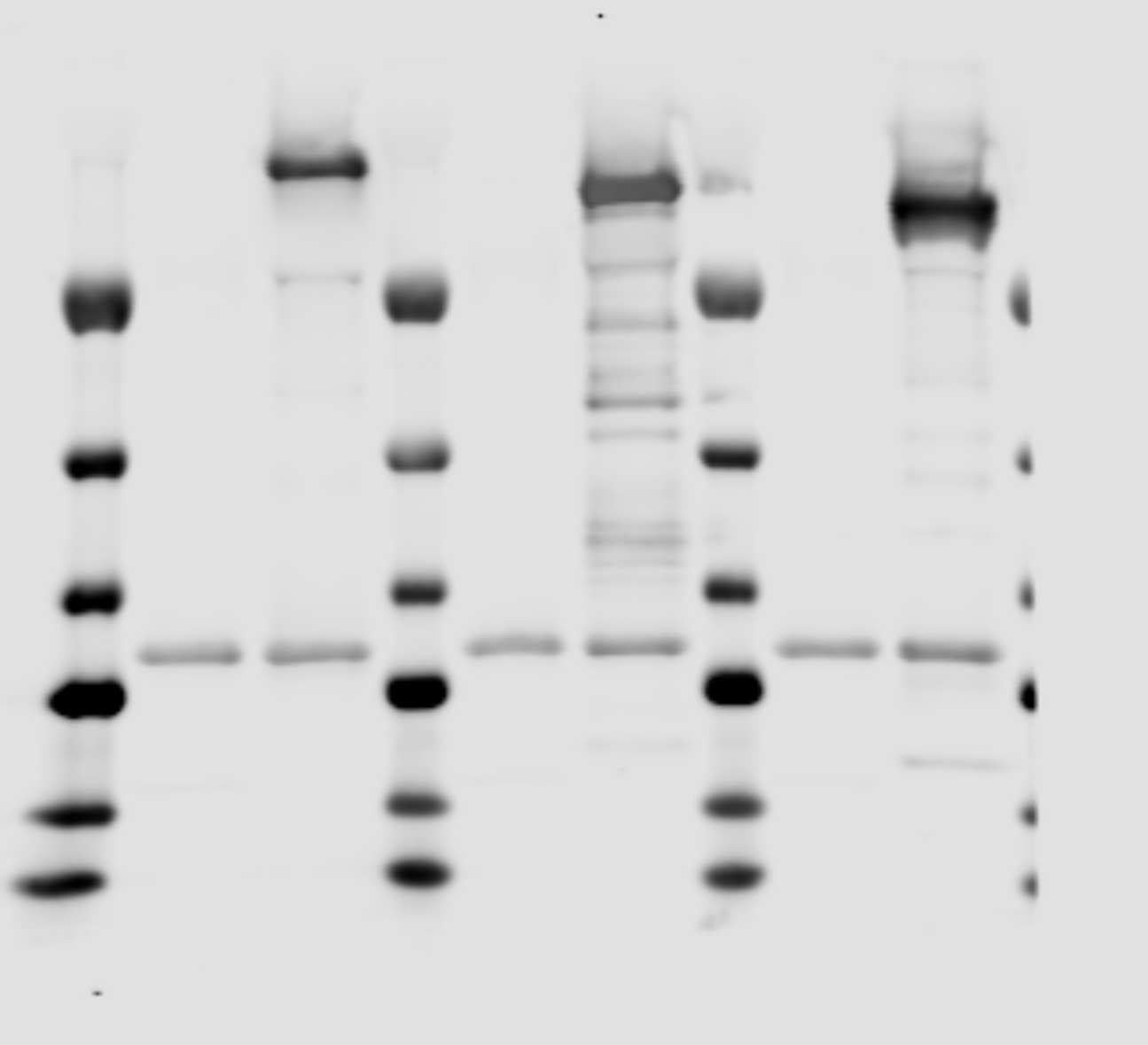

Supplement: Supplementary file 18 — Source Data for Figure 5 [file EMMM-15-e17094-s001.zip › EMM-2022-17094_source_data_figure_5/figure_5C/V5-tag IP.tif]

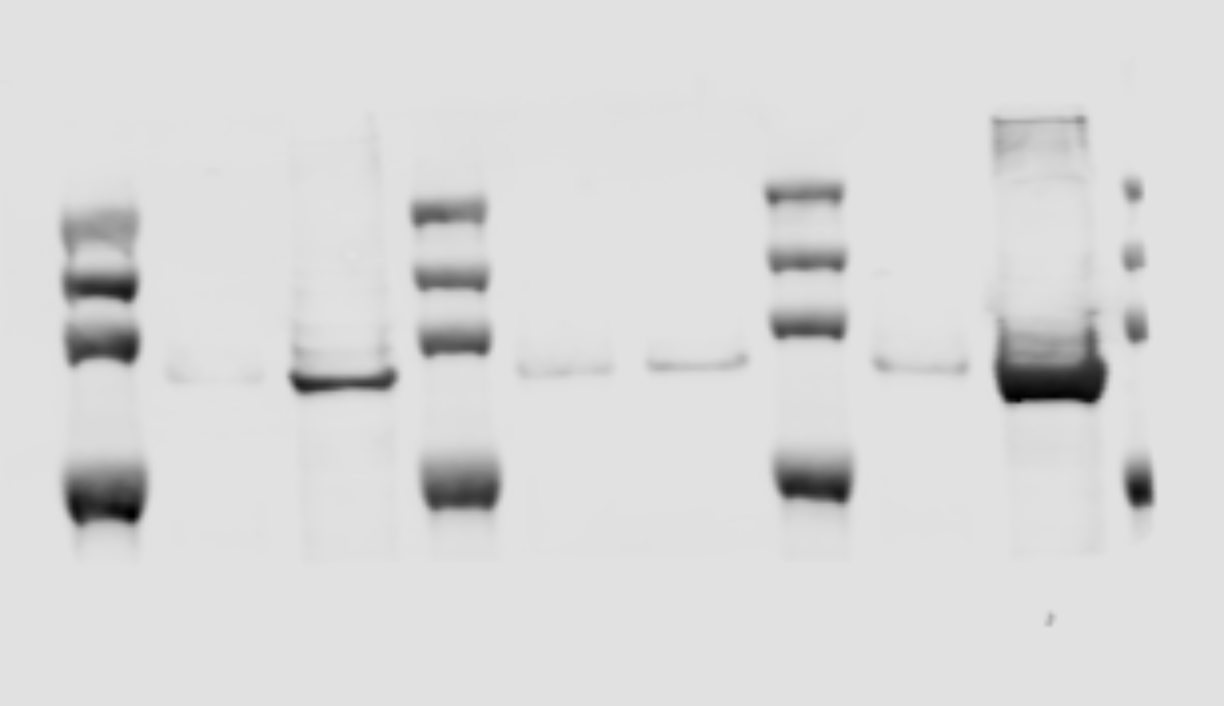

Supplement: Supplementary file 18 — Source Data for Figure 5 [file EMMM-15-e17094-s001.zip › EMM-2022-17094_source_data_figure_5/figure_5C/Myc-tag IP.tif]

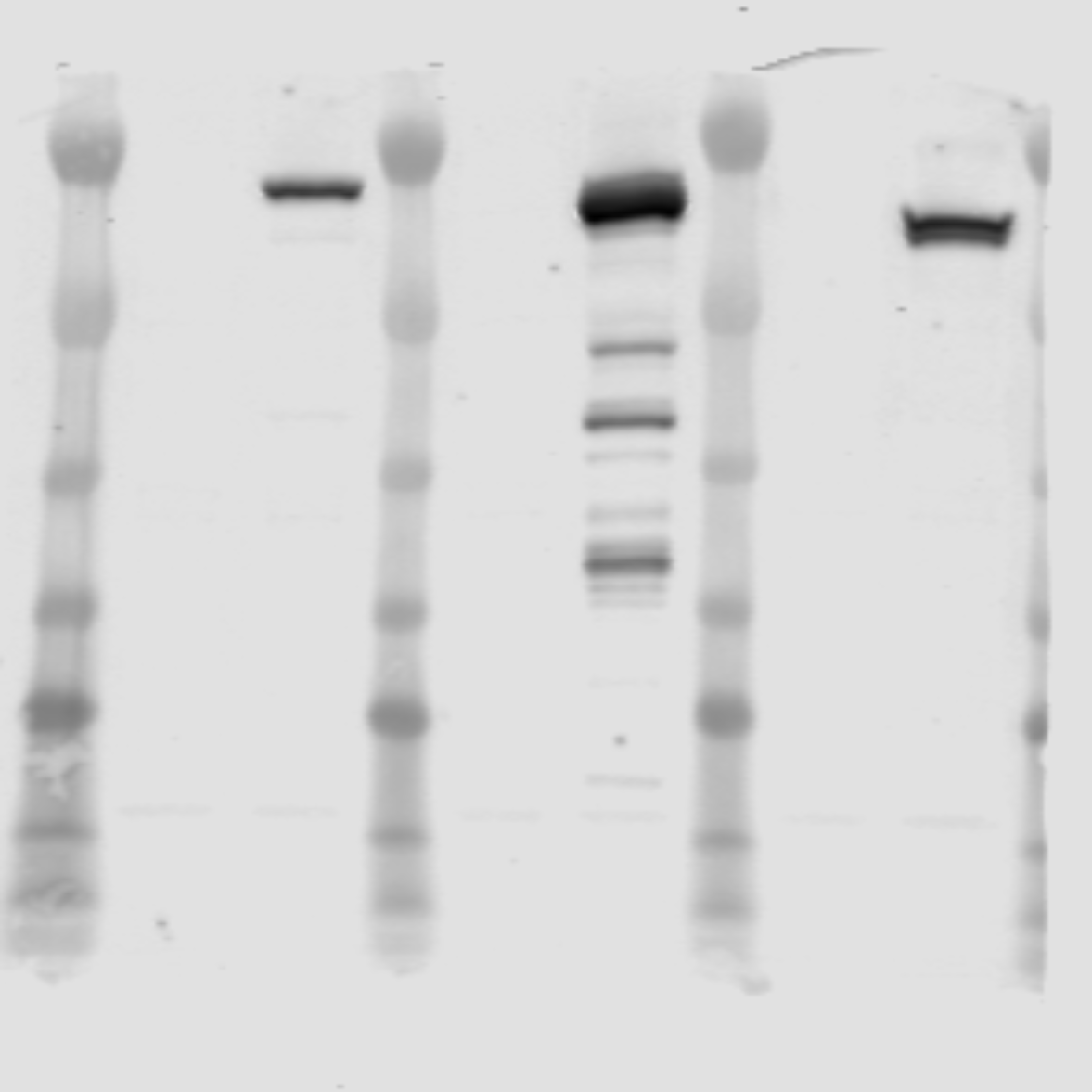

Supplement: Supplementary file 18 — Source Data for Figure 5 [file EMMM-15-e17094-s001.zip › EMM-2022-17094_source_data_figure_5/figure_5C/V5-tag Input.tif]

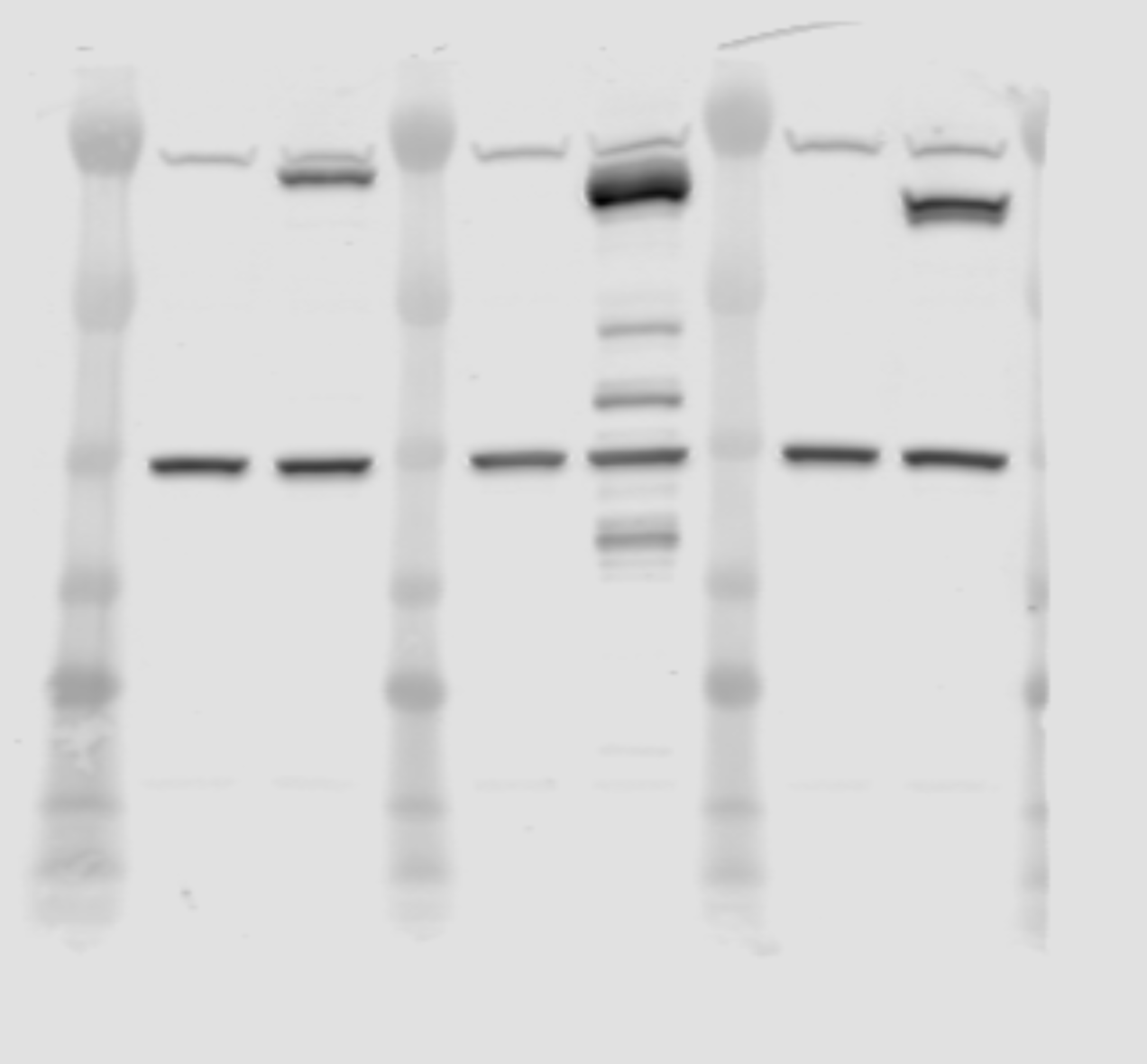

Supplement: Supplementary file 18 — Source Data for Figure 5 [file EMMM-15-e17094-s001.zip › EMM-2022-17094_source_data_figure_5/figure_5C/GAPDH Input.tif]

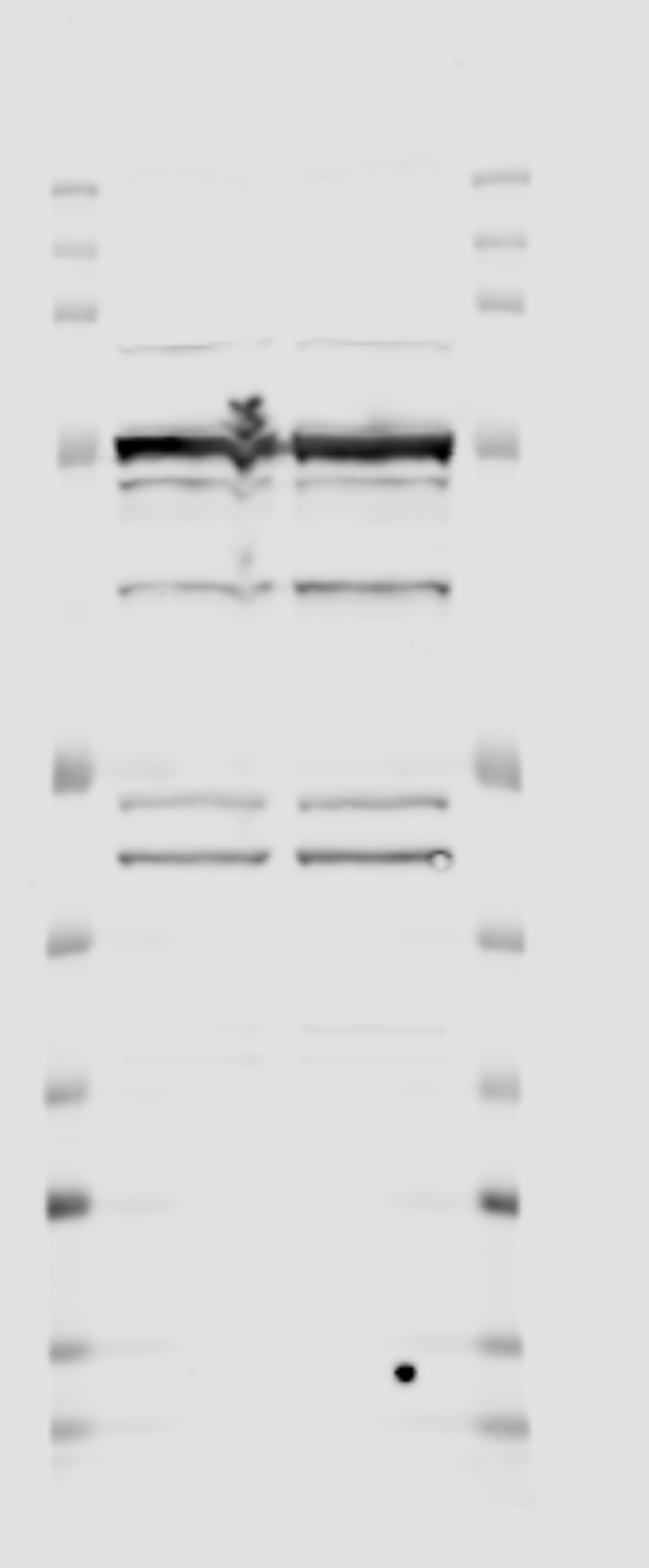

Supplement: Supplementary file 18 — Source Data for Figure 5 [file EMMM-15-e17094-s001.zip › EMM-2022-17094_source_data_figure_5/figure_5B/Myc-tag Input.tif]

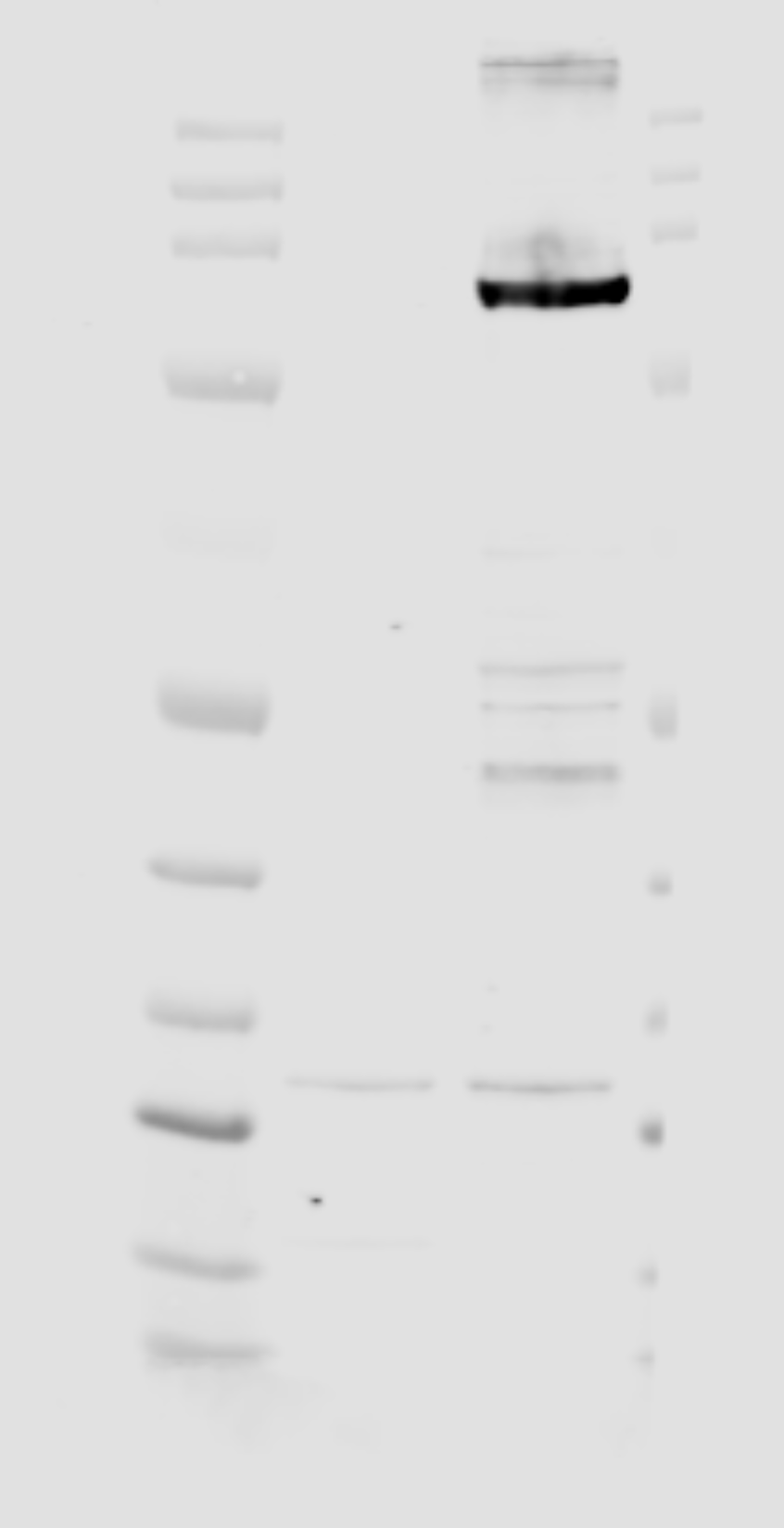

Supplement: Supplementary file 18 — Source Data for Figure 5 [file EMMM-15-e17094-s001.zip › EMM-2022-17094_source_data_figure_5/figure_5B/V5-tag IP.tif]

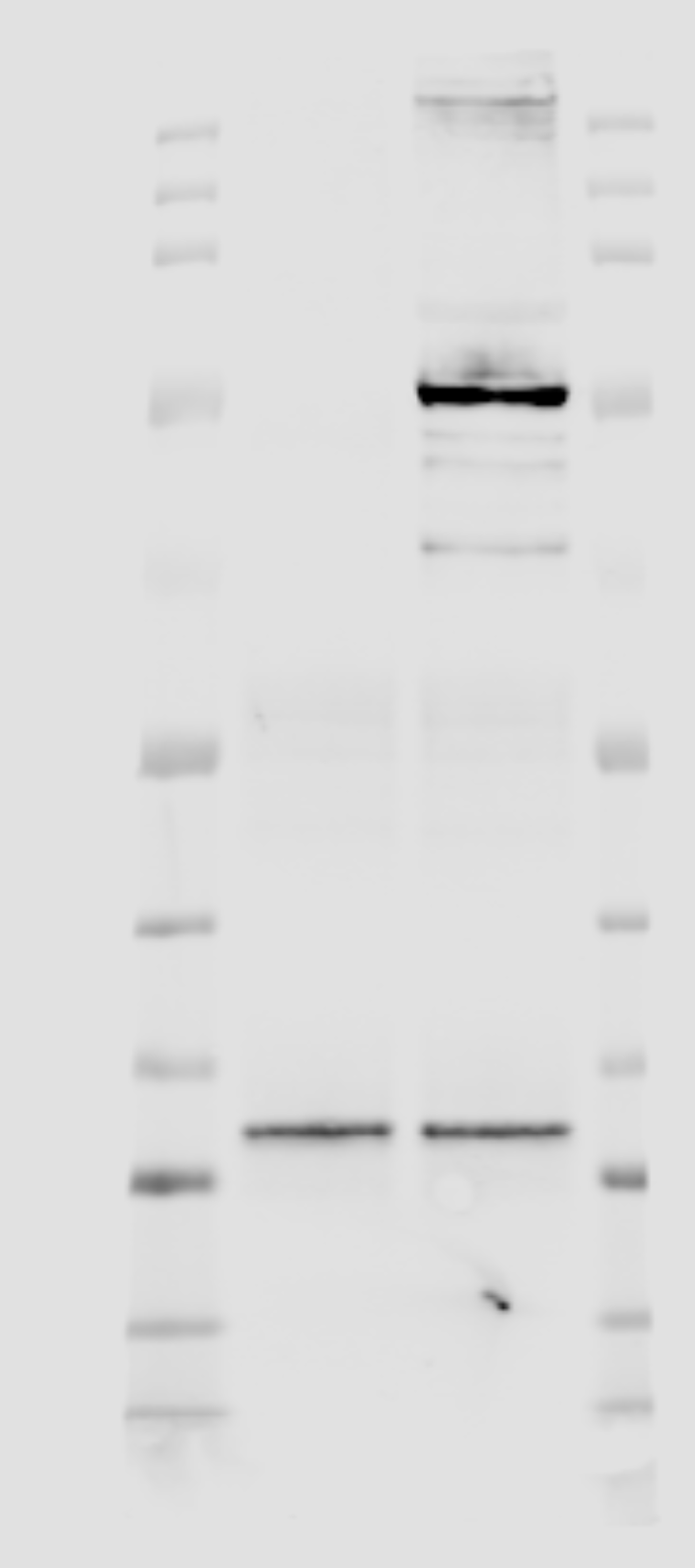

Supplement: Supplementary file 18 — Source Data for Figure 5 [file EMMM-15-e17094-s001.zip › EMM-2022-17094_source_data_figure_5/figure_5B/Myc-tag IP.tif]

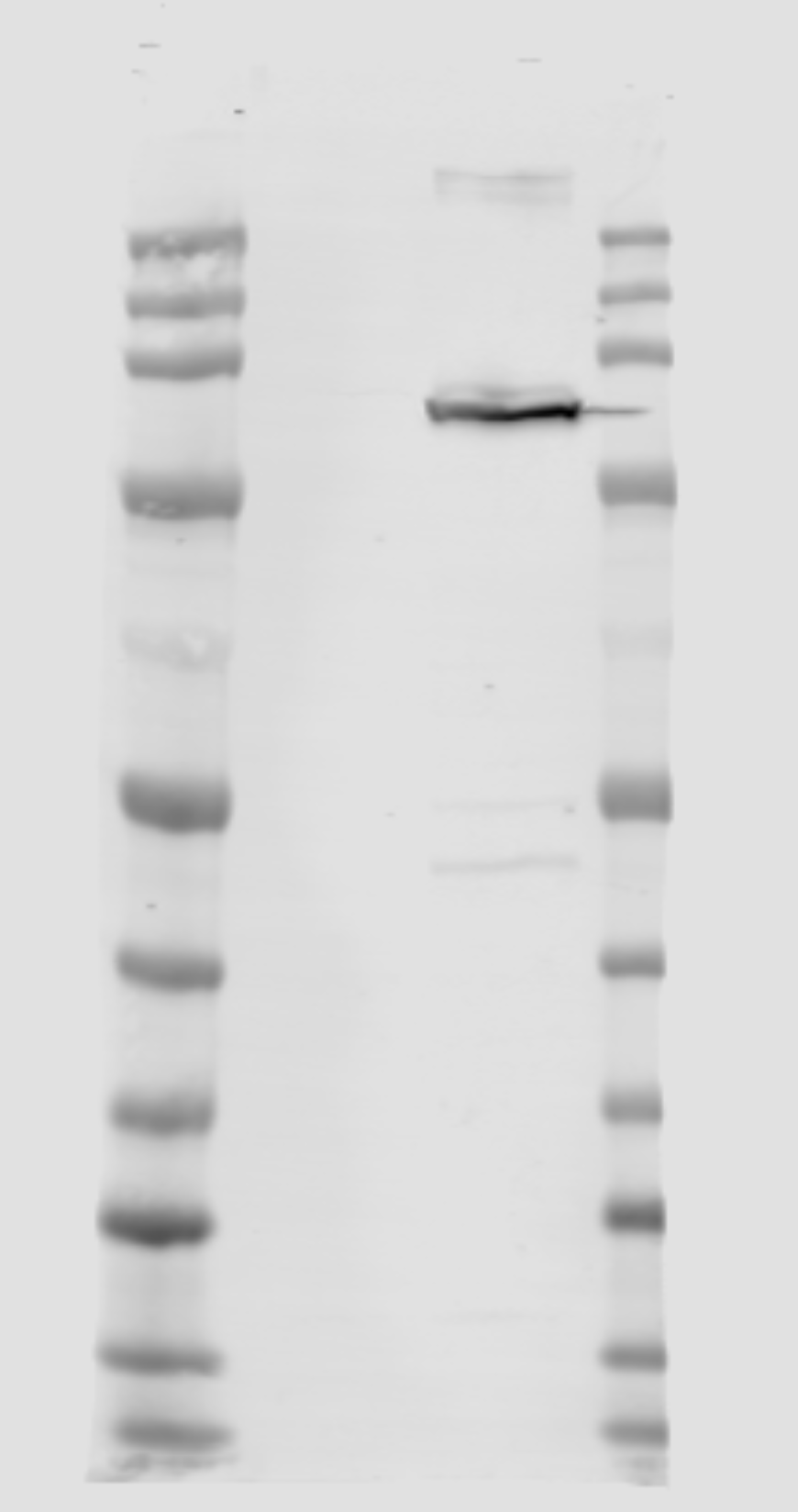

Supplement: Supplementary file 18 — Source Data for Figure 5 [file EMMM-15-e17094-s001.zip › EMM-2022-17094_source_data_figure_5/figure_5B/V5-tag Input.tif]

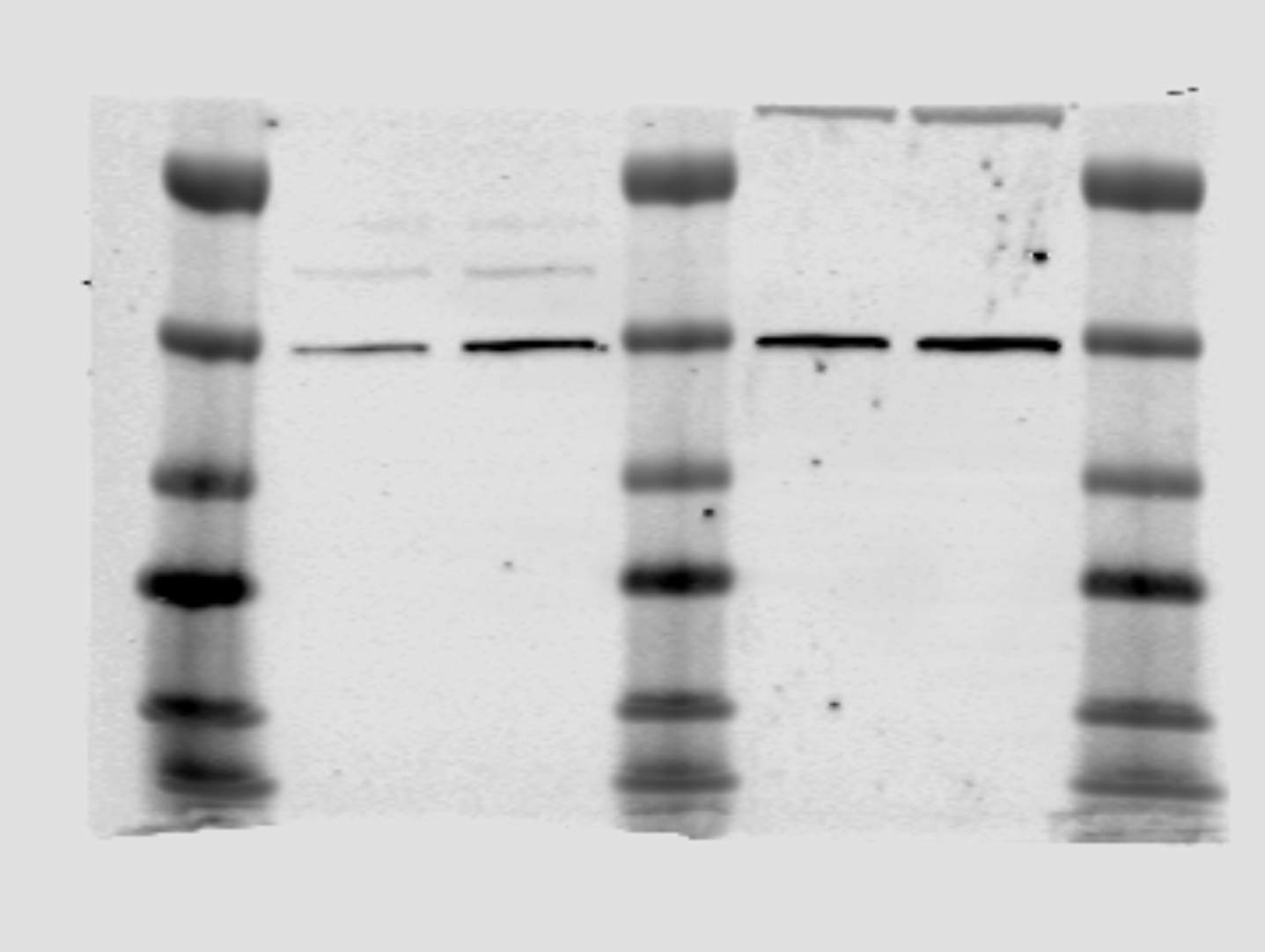

Supplement: Supplementary file 18 — Source Data for Figure 5 [file EMMM-15-e17094-s001.zip › EMM-2022-17094_source_data_figure_5/figure_5B/GAPDH Input.tif]

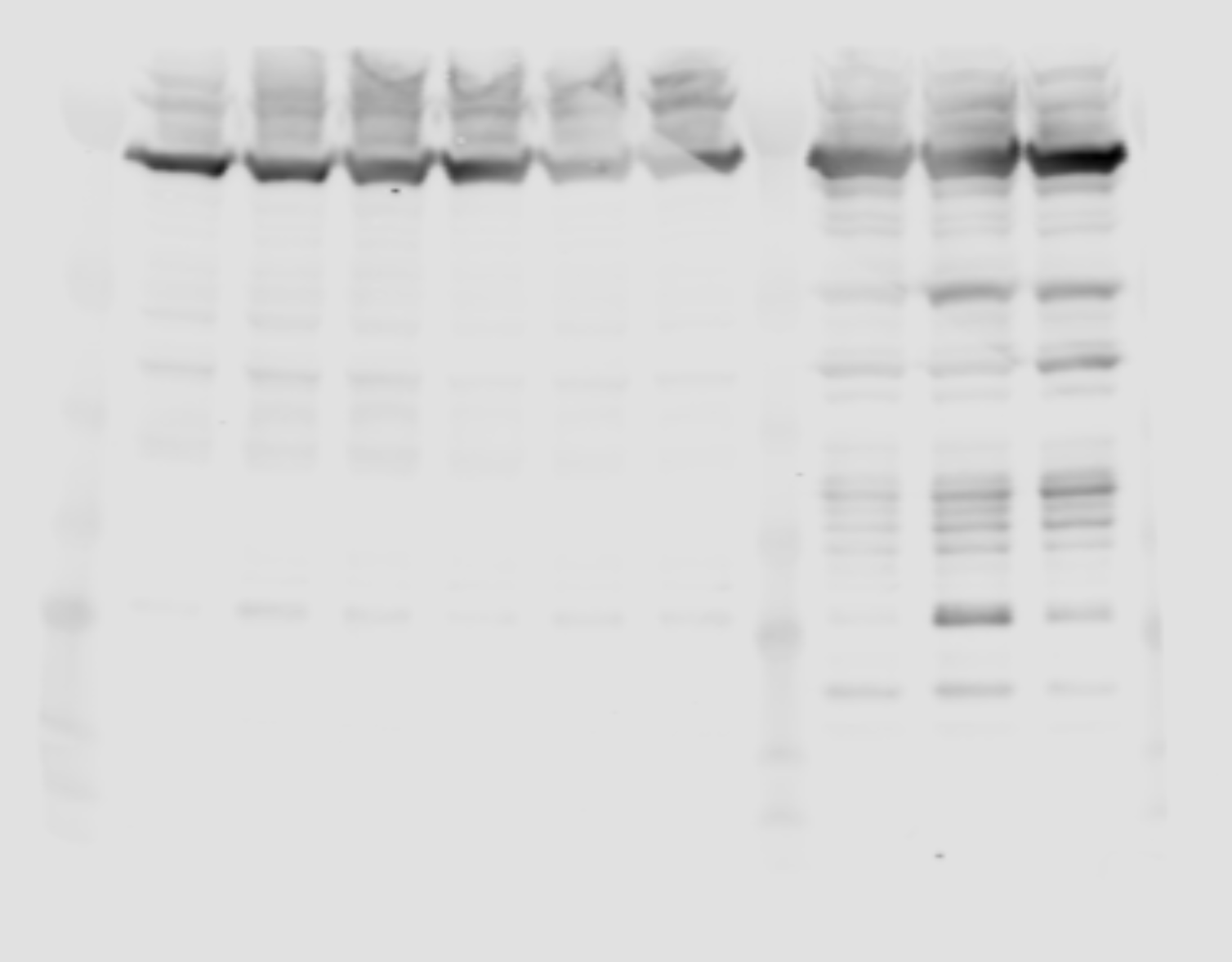

Supplement: Supplementary file 18 — Source Data for Figure 5 [file EMMM-15-e17094-s001.zip › EMM-2022-17094_source_data_figure_5/figure_5E/Myc-tag Input.tif]

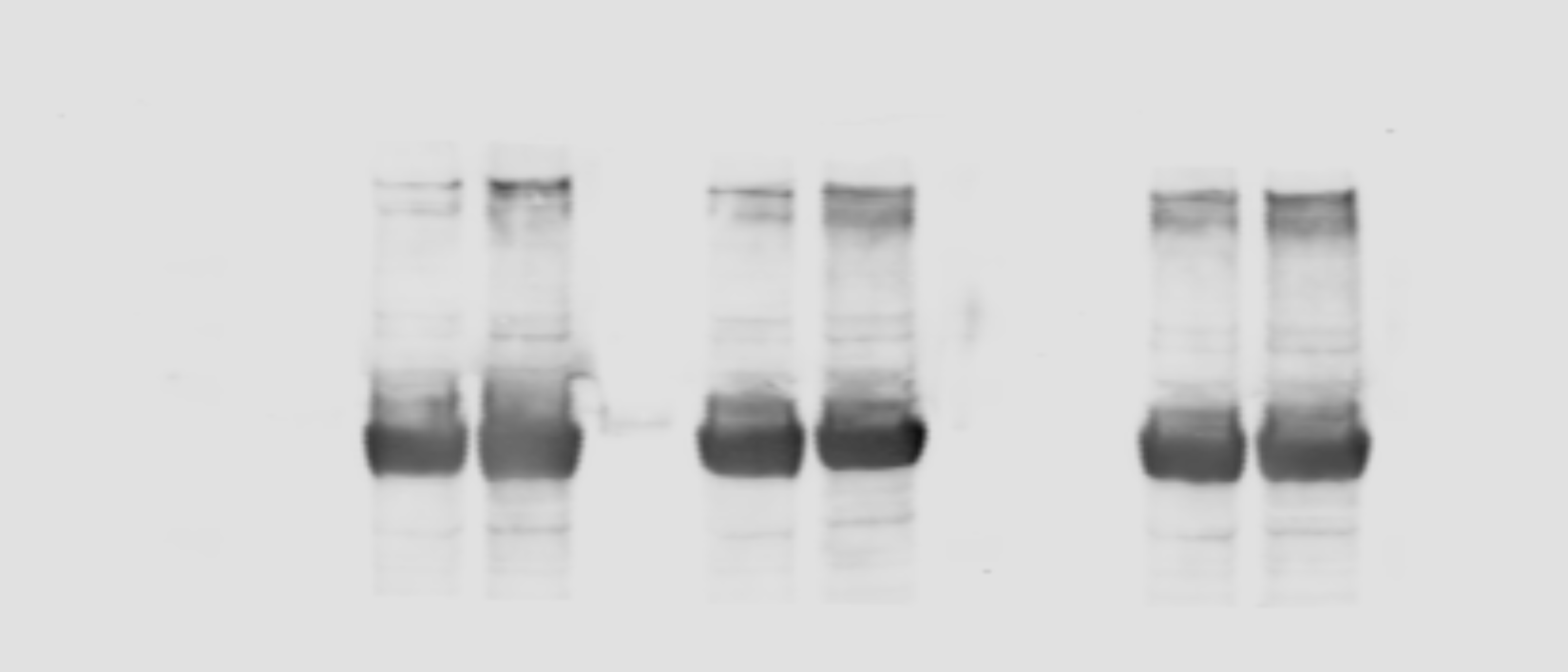

Supplement: Supplementary file 18 — Source Data for Figure 5 [file EMMM-15-e17094-s001.zip › EMM-2022-17094_source_data_figure_5/figure_5E/V5-tag IP.tif]

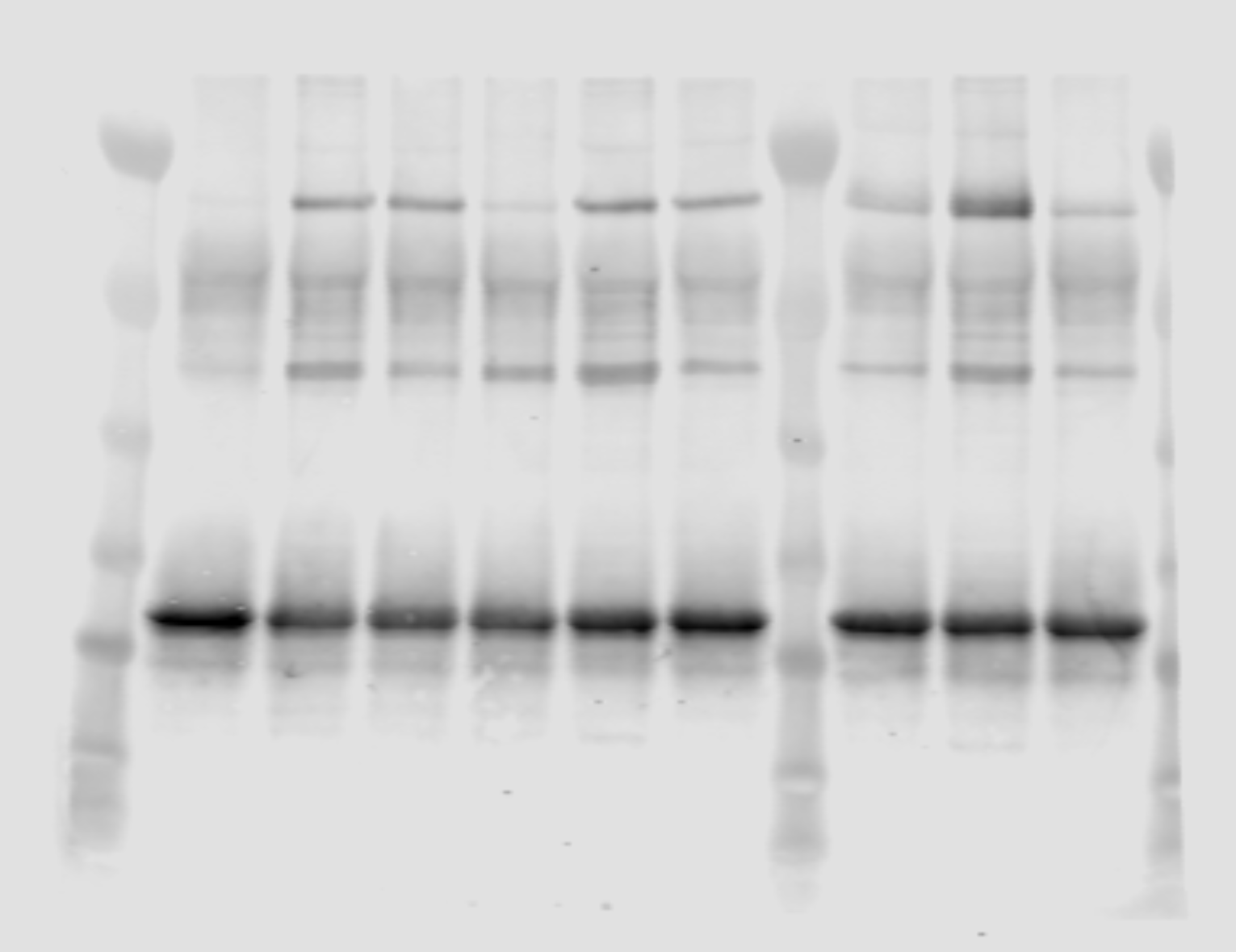

Supplement: Supplementary file 18 — Source Data for Figure 5 [file EMMM-15-e17094-s001.zip › EMM-2022-17094_source_data_figure_5/figure_5E/Myc-tag IP.tif]

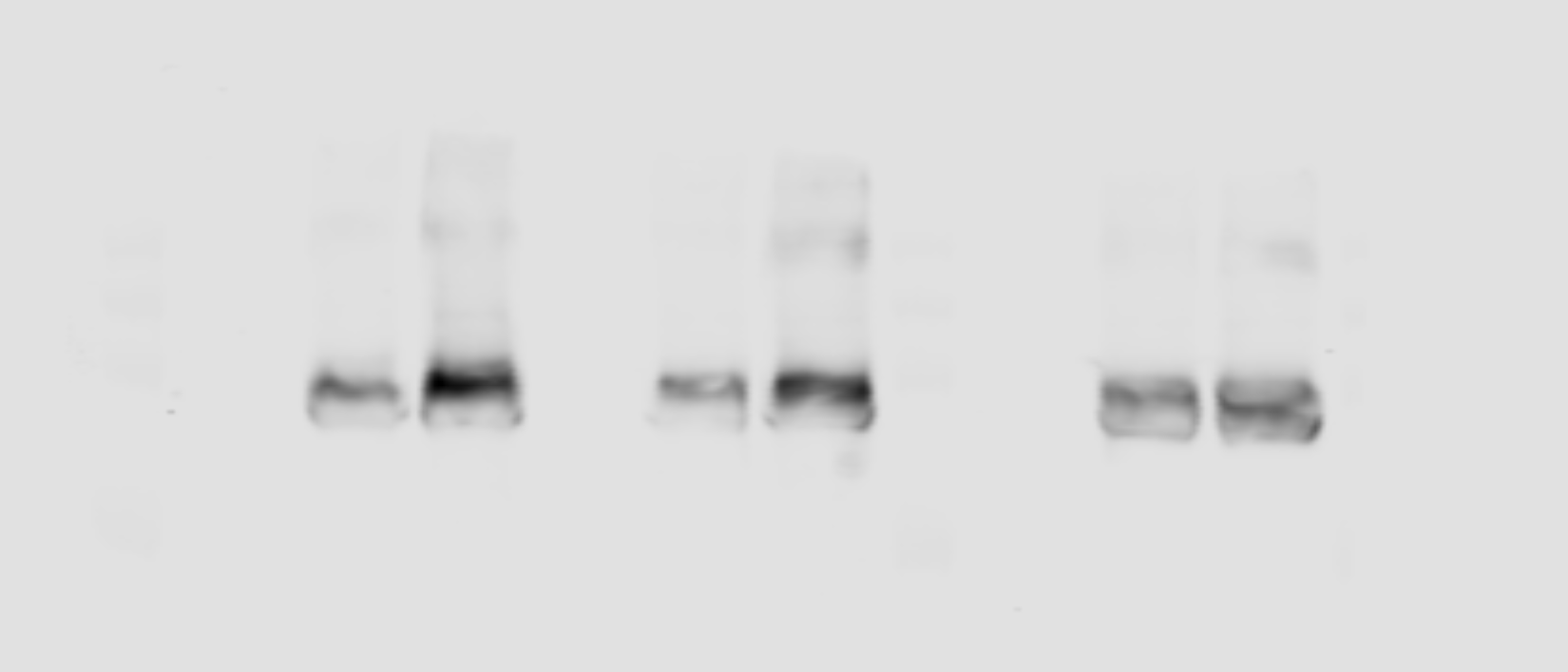

Supplement: Supplementary file 18 — Source Data for Figure 5 [file EMMM-15-e17094-s001.zip › EMM-2022-17094_source_data_figure_5/figure_5E/V5-tag Input.tif]

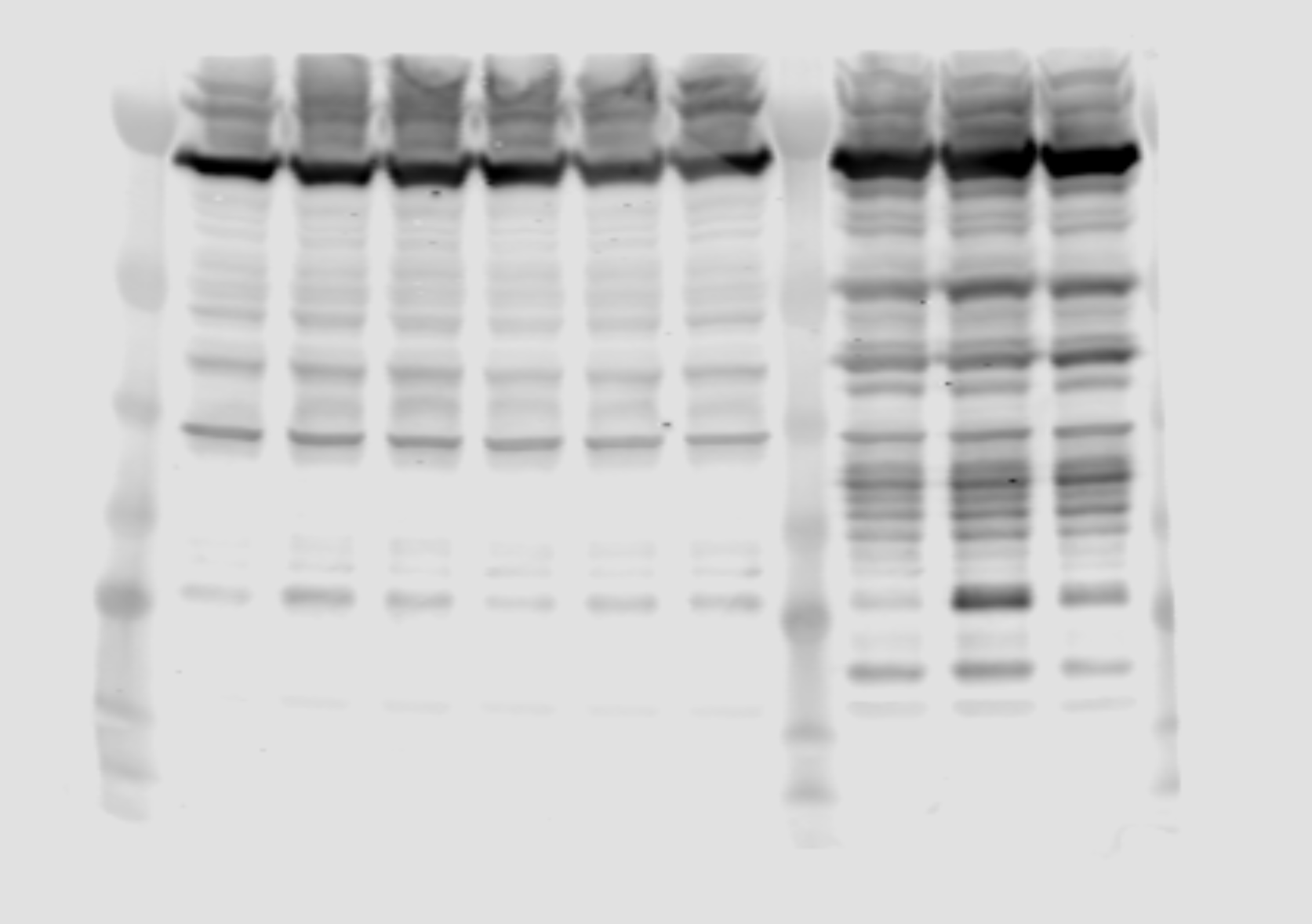

Supplement: Supplementary file 18 — Source Data for Figure 5 [file EMMM-15-e17094-s001.zip › EMM-2022-17094_source_data_figure_5/figure_5E/GAPDH Input.tif]

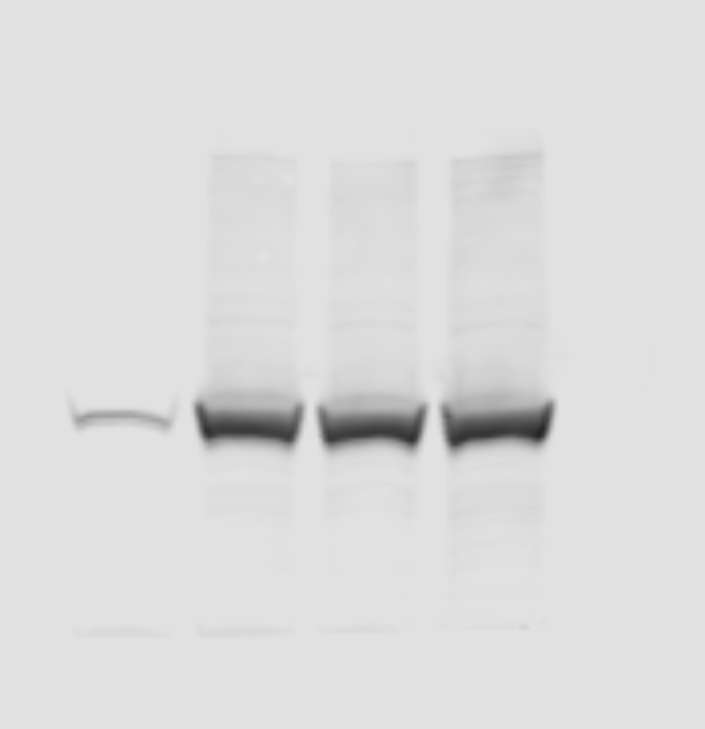

Supplement: Supplementary file 18 — Source Data for Figure 5 [file EMMM-15-e17094-s001.zip › EMM-2022-17094_source_data_figure_5/figure_5G/Myc-tag Input.tif]

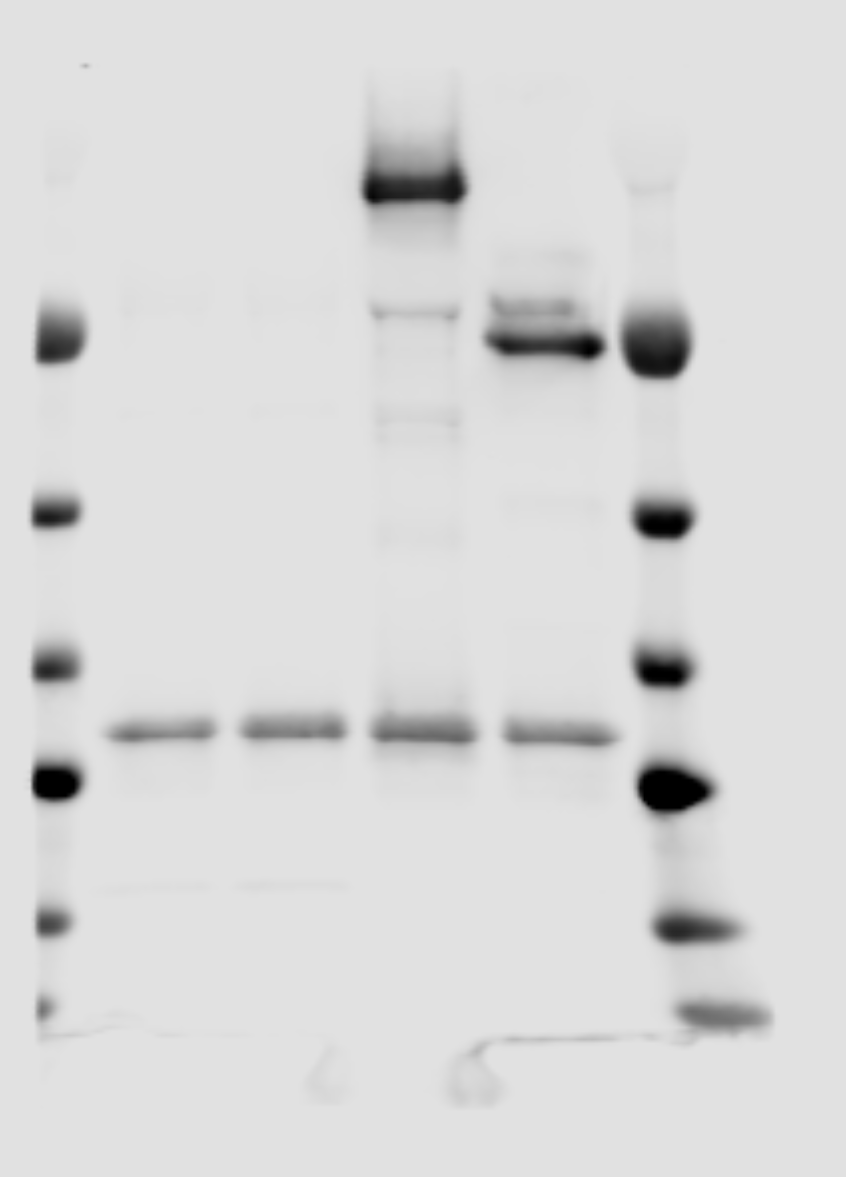

Supplement: Supplementary file 18 — Source Data for Figure 5 [file EMMM-15-e17094-s001.zip › EMM-2022-17094_source_data_figure_5/figure_5G/V5-tag IP.tif]

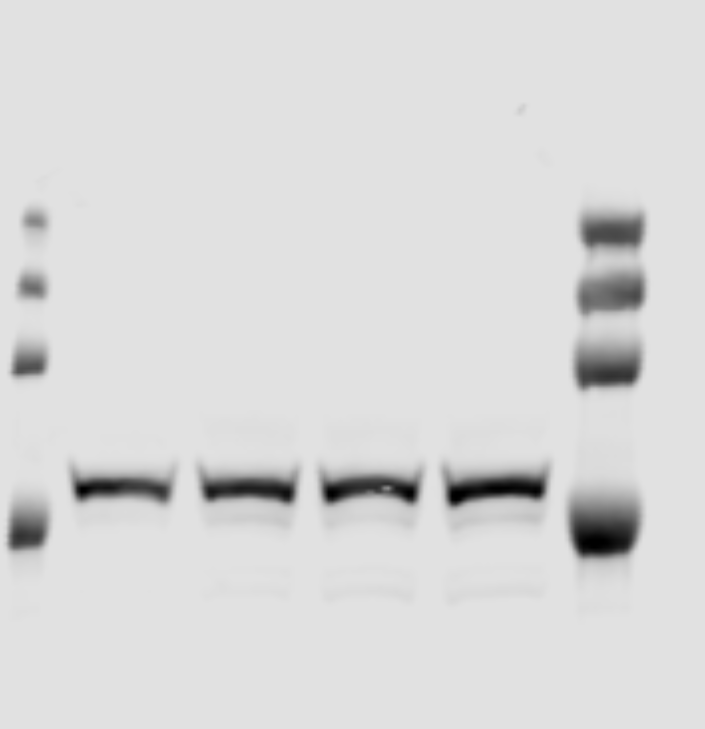

Supplement: Supplementary file 18 — Source Data for Figure 5 [file EMMM-15-e17094-s001.zip › EMM-2022-17094_source_data_figure_5/figure_5G/Beta-catenin Input.tif]
