# Supplementary figures and images for: Functional analysis reveals driver cooperativity and novel mechanisms in endometrial carcinogenesis (part 2 of 2)
Source: EMBO Mol Med. 2023 Aug 17;15(10):e17094. doi: 10.15252/emmm.202217094 (PMC10565641; doi:10.15252/emmm.202217094)

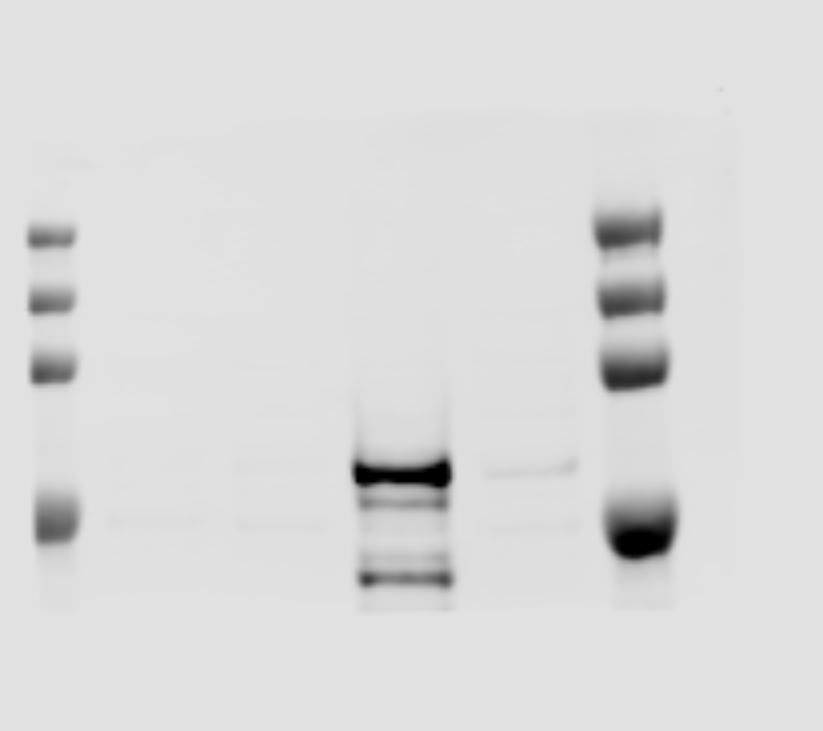

Supplement: Supplementary file 18 — Source Data for Figure 5 [file EMMM-15-e17094-s001.zip › EMM-2022-17094_source_data_figure_5/figure_5G/Beta-catenin IP.tif]

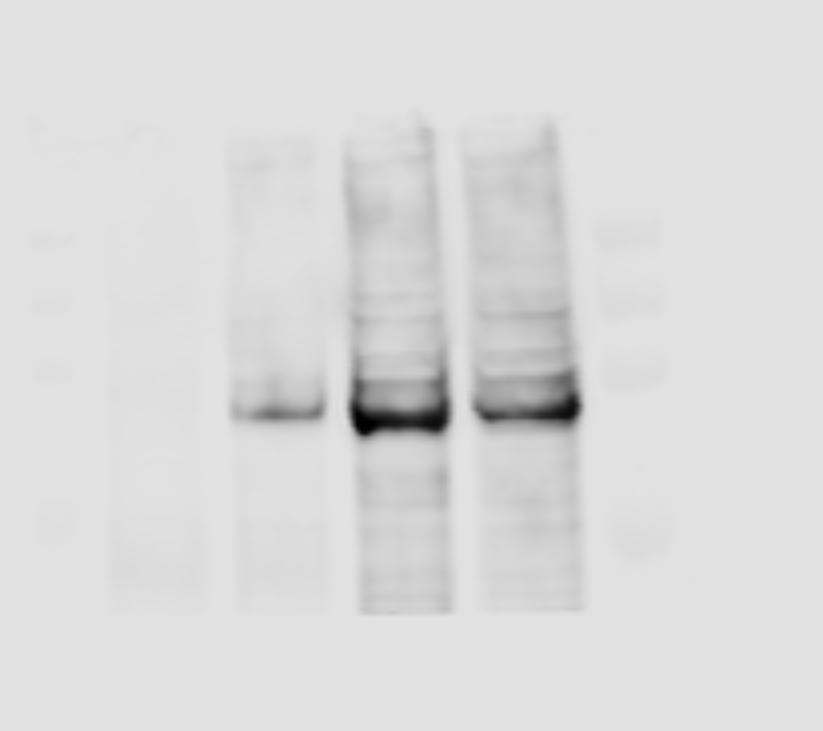

Supplement: Supplementary file 18 — Source Data for Figure 5 [file EMMM-15-e17094-s001.zip › EMM-2022-17094_source_data_figure_5/figure_5G/Myc-tag IP.tif]

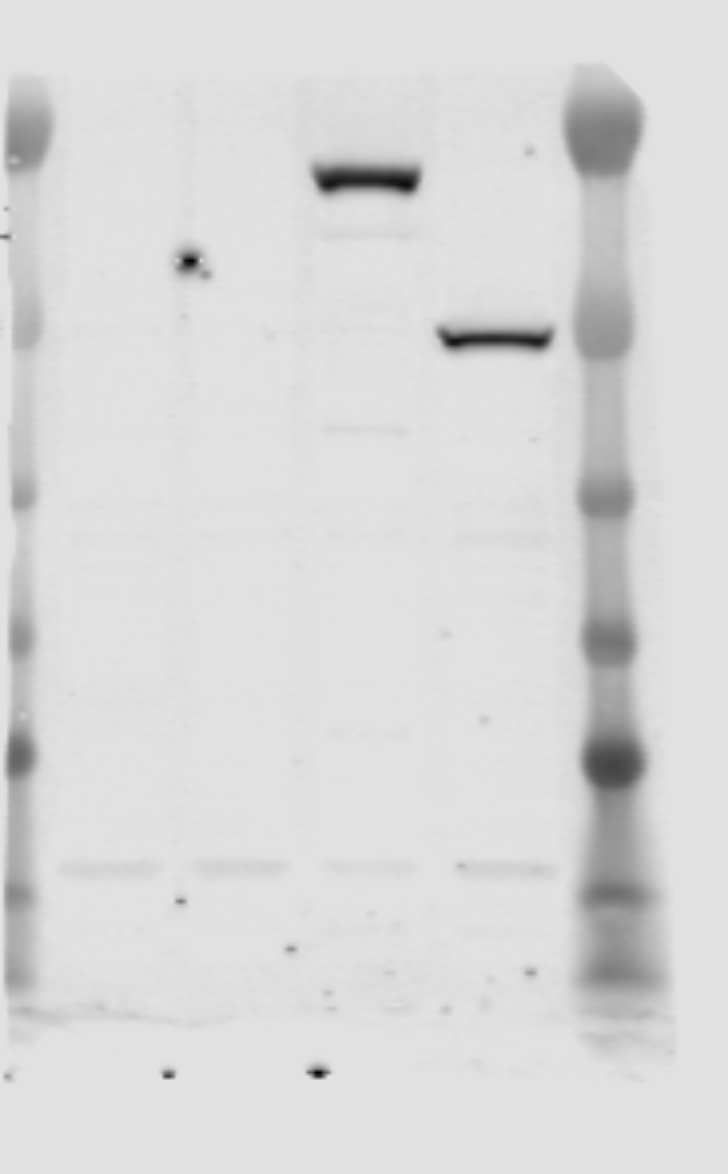

Supplement: Supplementary file 18 — Source Data for Figure 5 [file EMMM-15-e17094-s001.zip › EMM-2022-17094_source_data_figure_5/figure_5G/V5-tag Input.tif]

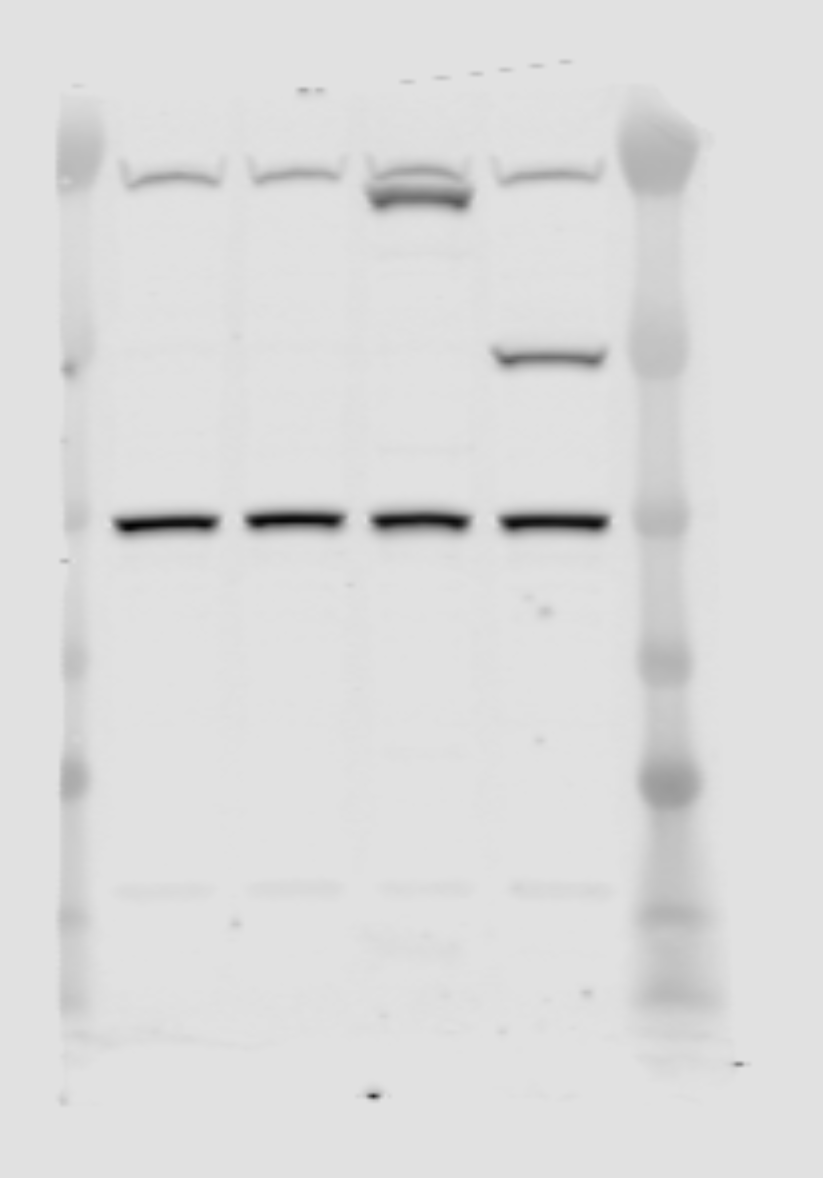

Supplement: Supplementary file 18 — Source Data for Figure 5 [file EMMM-15-e17094-s001.zip › EMM-2022-17094_source_data_figure_5/figure_5G/GAPDH Input.tif]

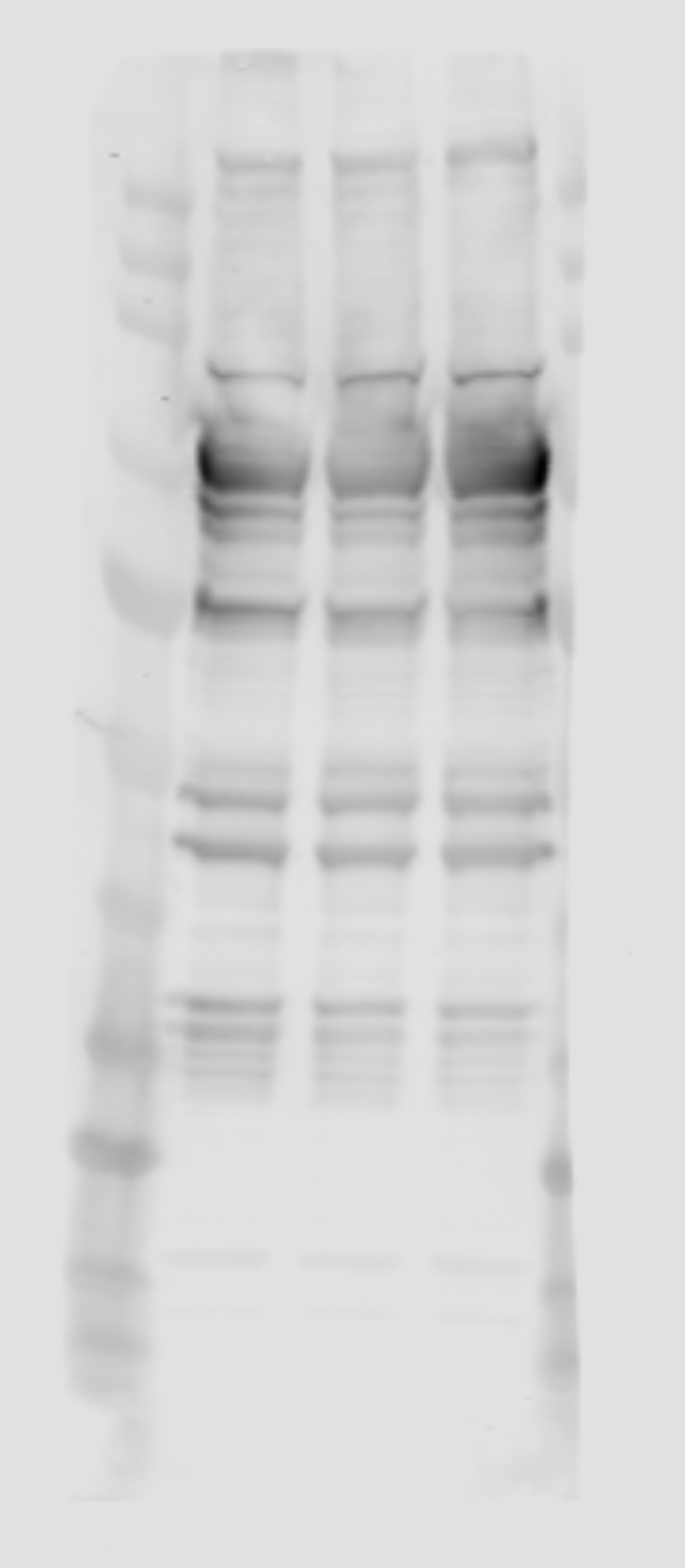

Supplement: Supplementary file 18 — Source Data for Figure 5 [file EMMM-15-e17094-s001.zip › EMM-2022-17094_source_data_figure_5/figure_5F/Myc-tag Input.tif]

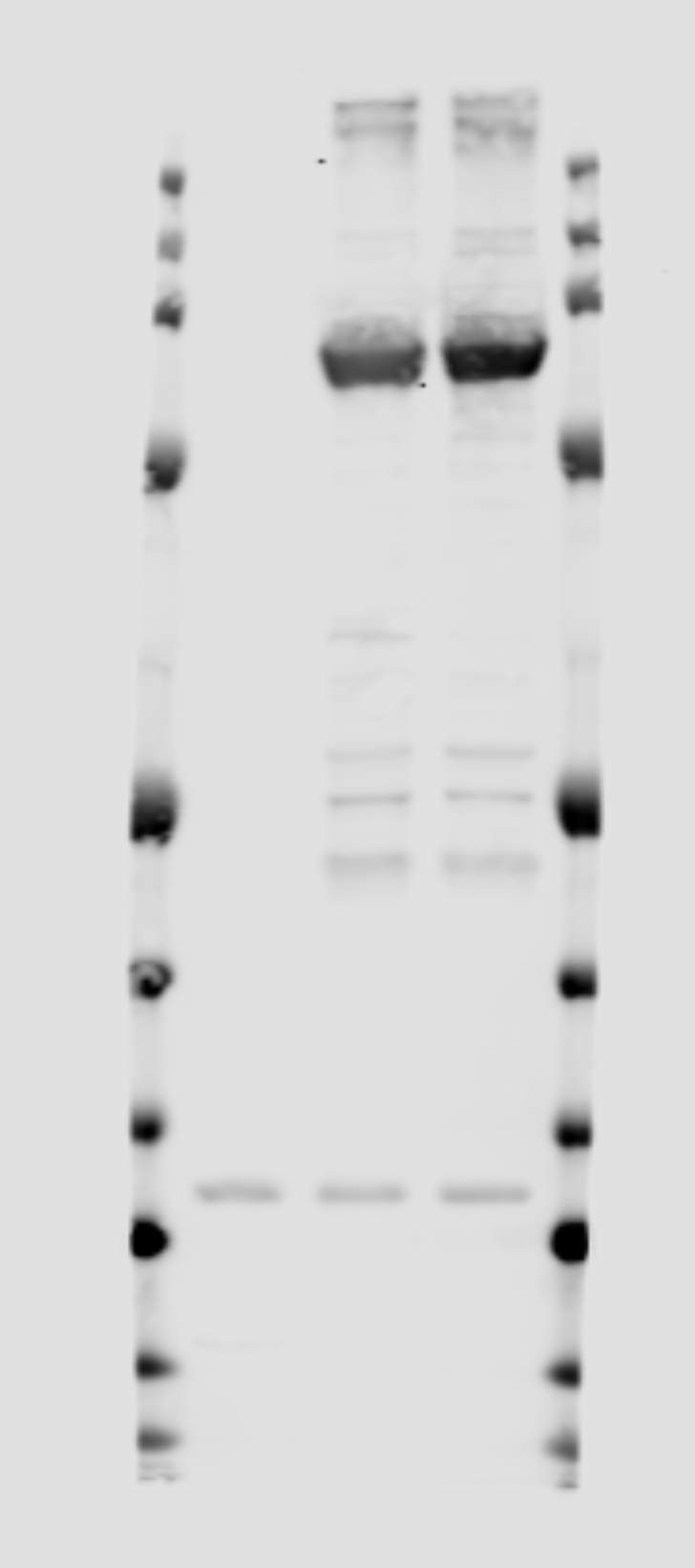

Supplement: Supplementary file 18 — Source Data for Figure 5 [file EMMM-15-e17094-s001.zip › EMM-2022-17094_source_data_figure_5/figure_5F/V5-tag IP.tif]

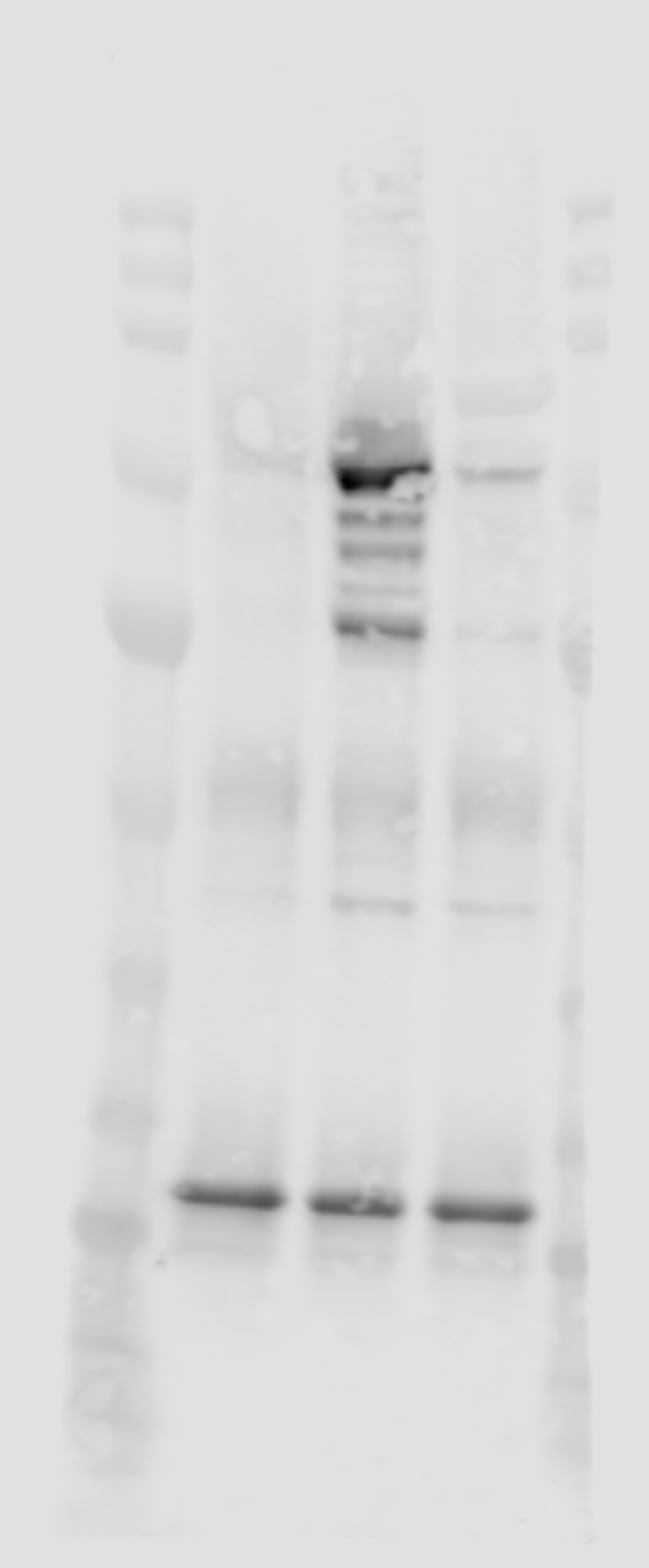

Supplement: Supplementary file 18 — Source Data for Figure 5 [file EMMM-15-e17094-s001.zip › EMM-2022-17094_source_data_figure_5/figure_5F/Myc-tag IP.tif]

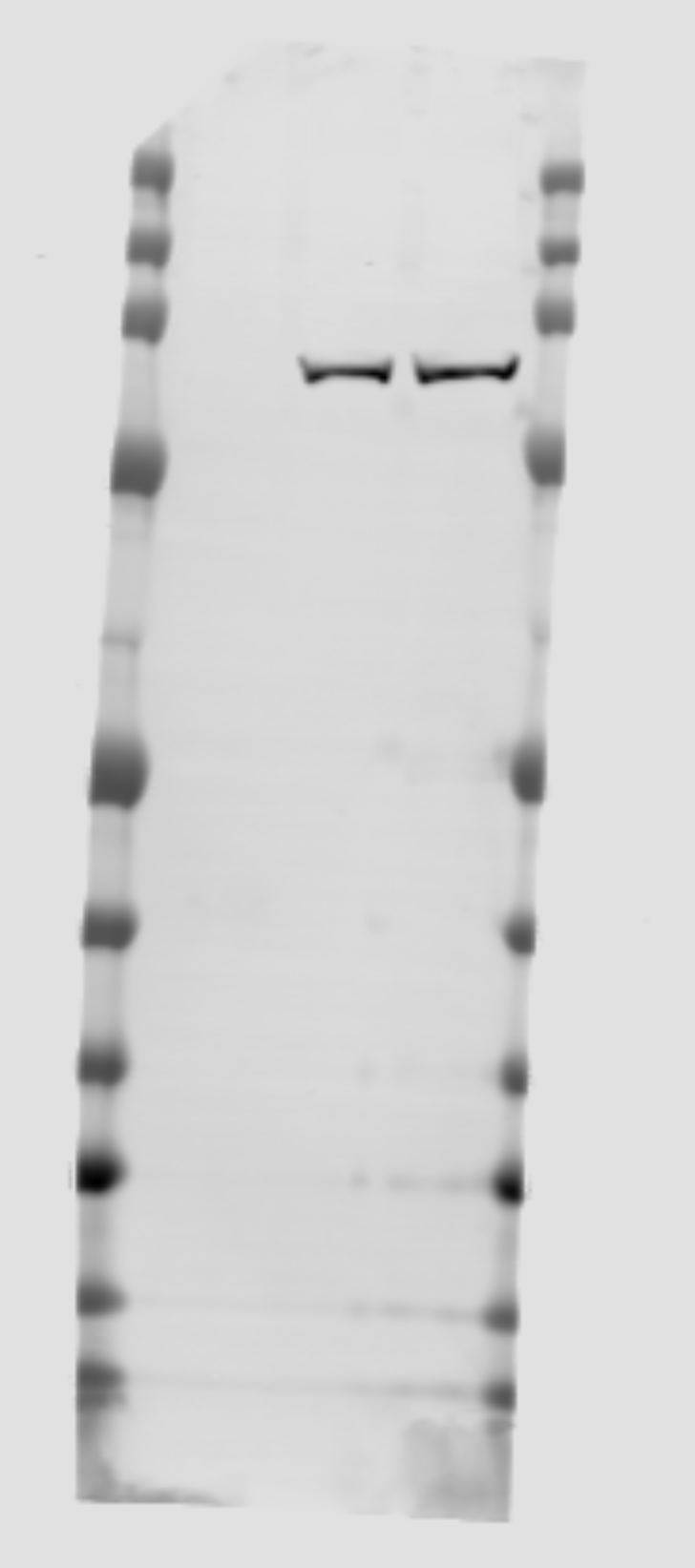

Supplement: Supplementary file 18 — Source Data for Figure 5 [file EMMM-15-e17094-s001.zip › EMM-2022-17094_source_data_figure_5/figure_5F/V5-tag Input.tif]

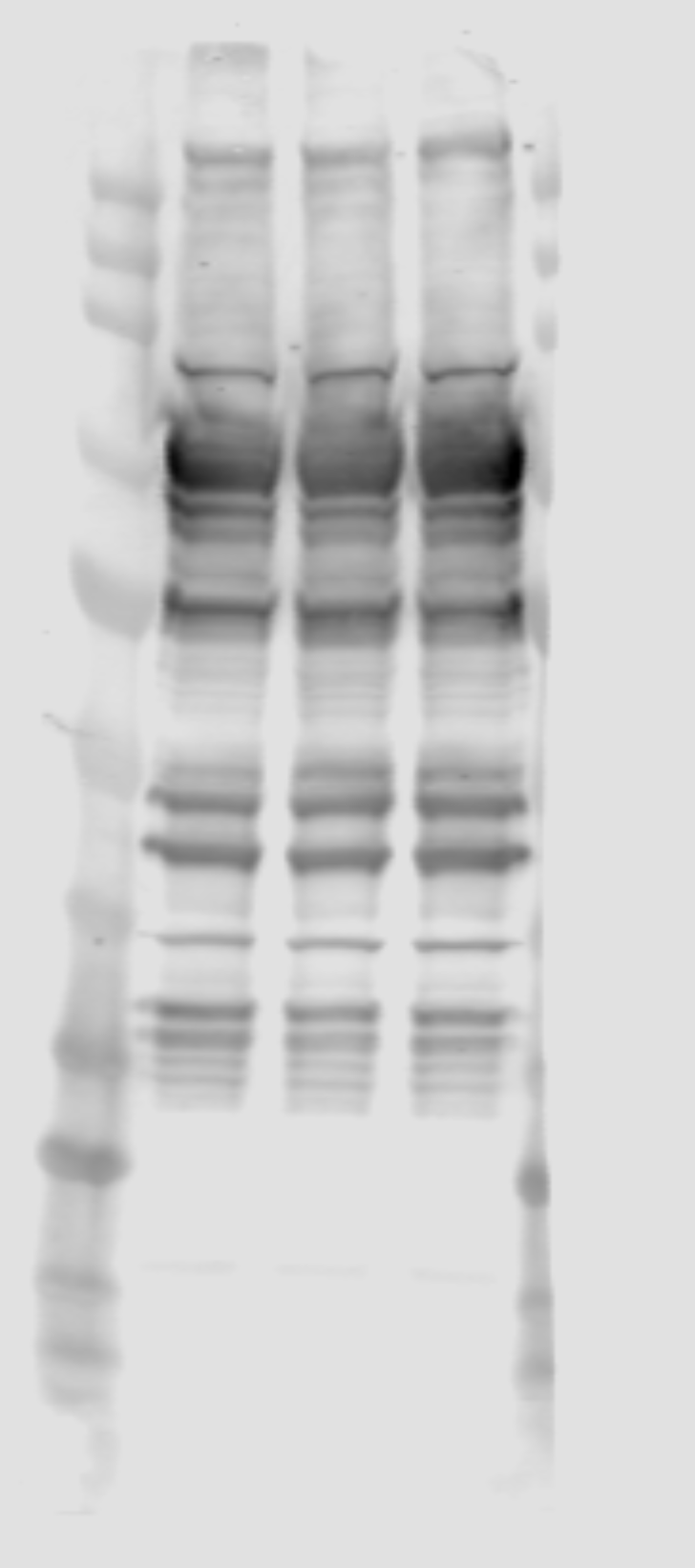

Supplement: Supplementary file 18 — Source Data for Figure 5 [file EMMM-15-e17094-s001.zip › EMM-2022-17094_source_data_figure_5/figure_5F/GAPDH Input.tif]

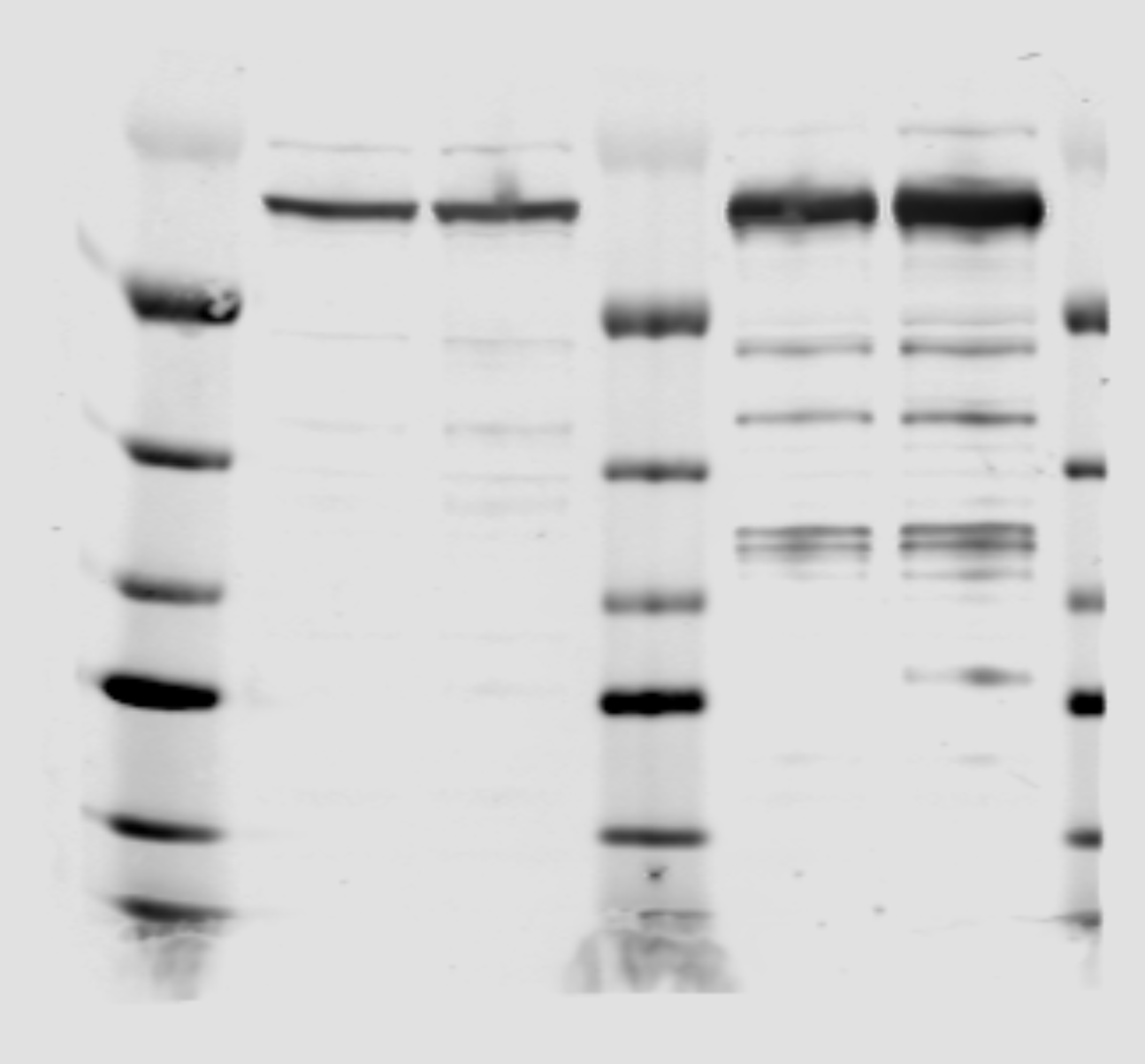

Supplement: Supplementary file 18 — Source Data for Figure 5 [file EMMM-15-e17094-s001.zip › EMM-2022-17094_source_data_figure_5/figure_5A/Myc-tag Input.tif]

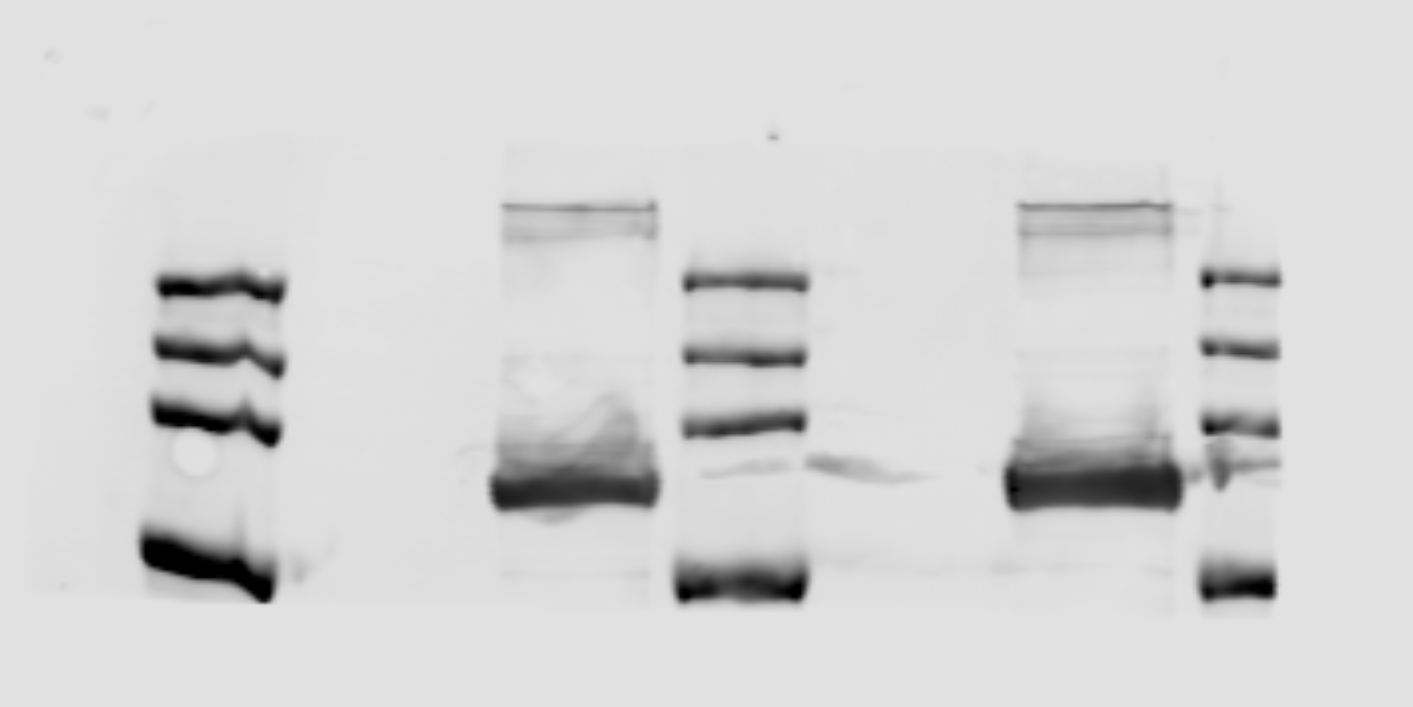

Supplement: Supplementary file 18 — Source Data for Figure 5 [file EMMM-15-e17094-s001.zip › EMM-2022-17094_source_data_figure_5/figure_5A/V5-tag IP.tif]

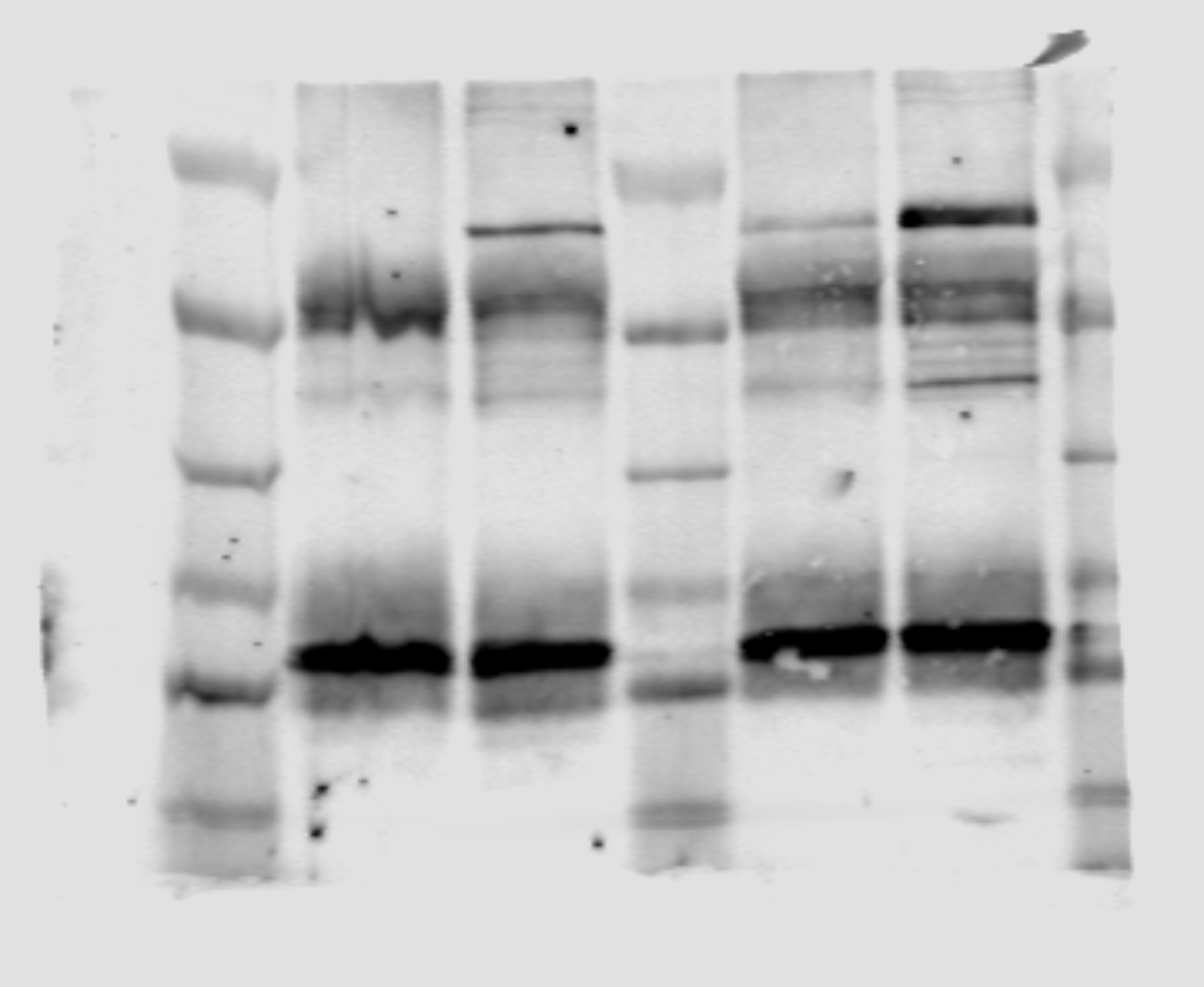

Supplement: Supplementary file 18 — Source Data for Figure 5 [file EMMM-15-e17094-s001.zip › EMM-2022-17094_source_data_figure_5/figure_5A/Myc-tag IP.tif]

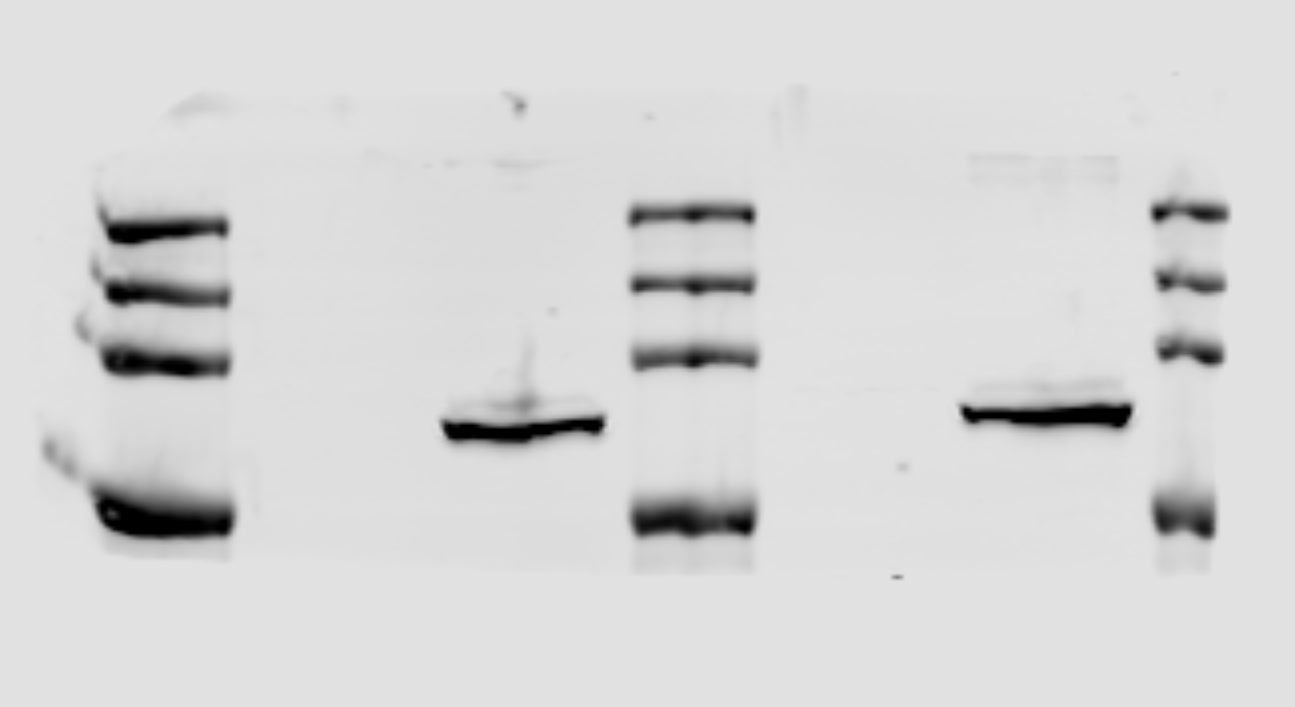

Supplement: Supplementary file 18 — Source Data for Figure 5 [file EMMM-15-e17094-s001.zip › EMM-2022-17094_source_data_figure_5/figure_5A/V5-tag Input.tif]

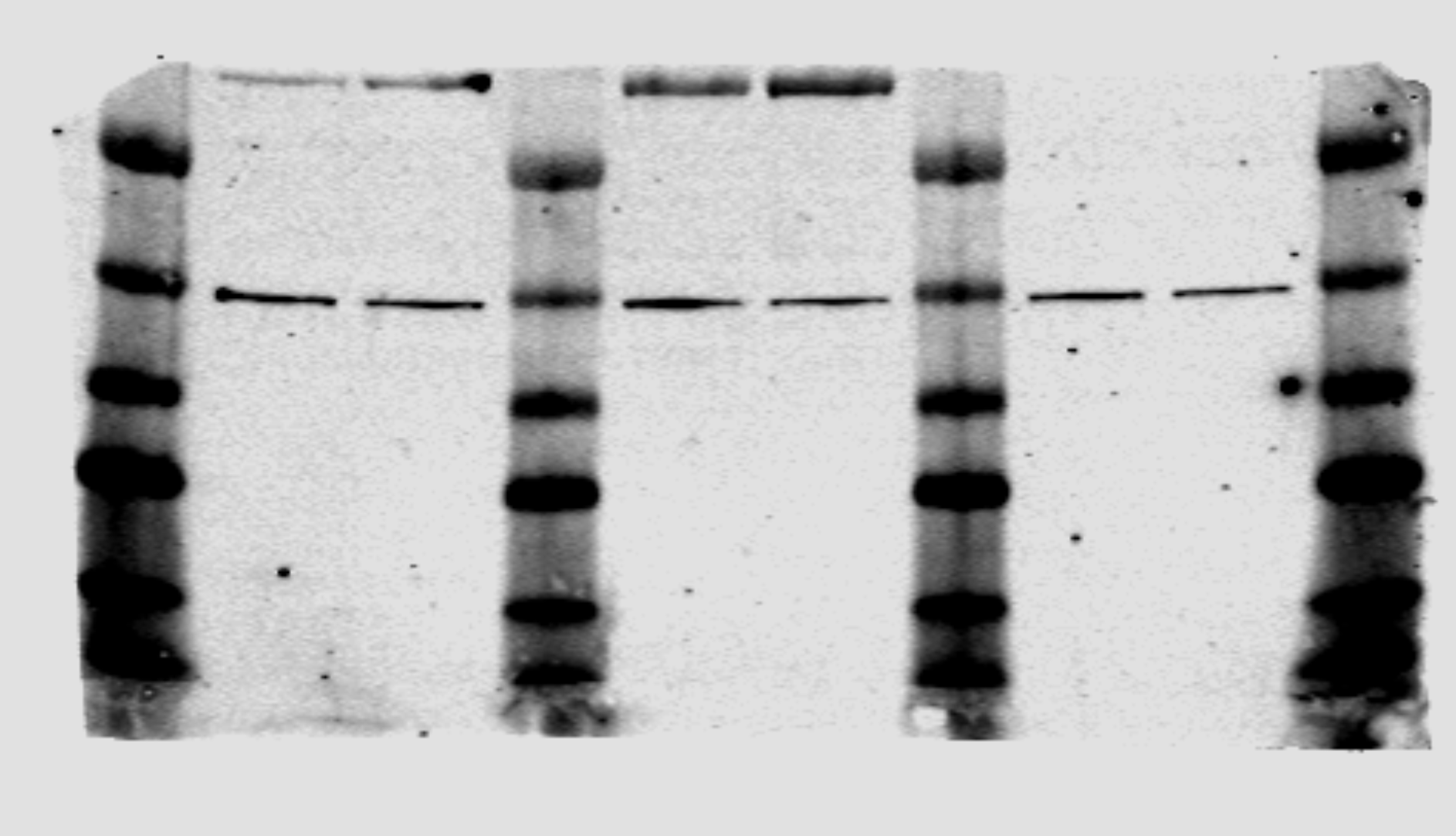

Supplement: Supplementary file 18 — Source Data for Figure 5 [file EMMM-15-e17094-s001.zip › EMM-2022-17094_source_data_figure_5/figure_5A/GAPDH Input.tif]

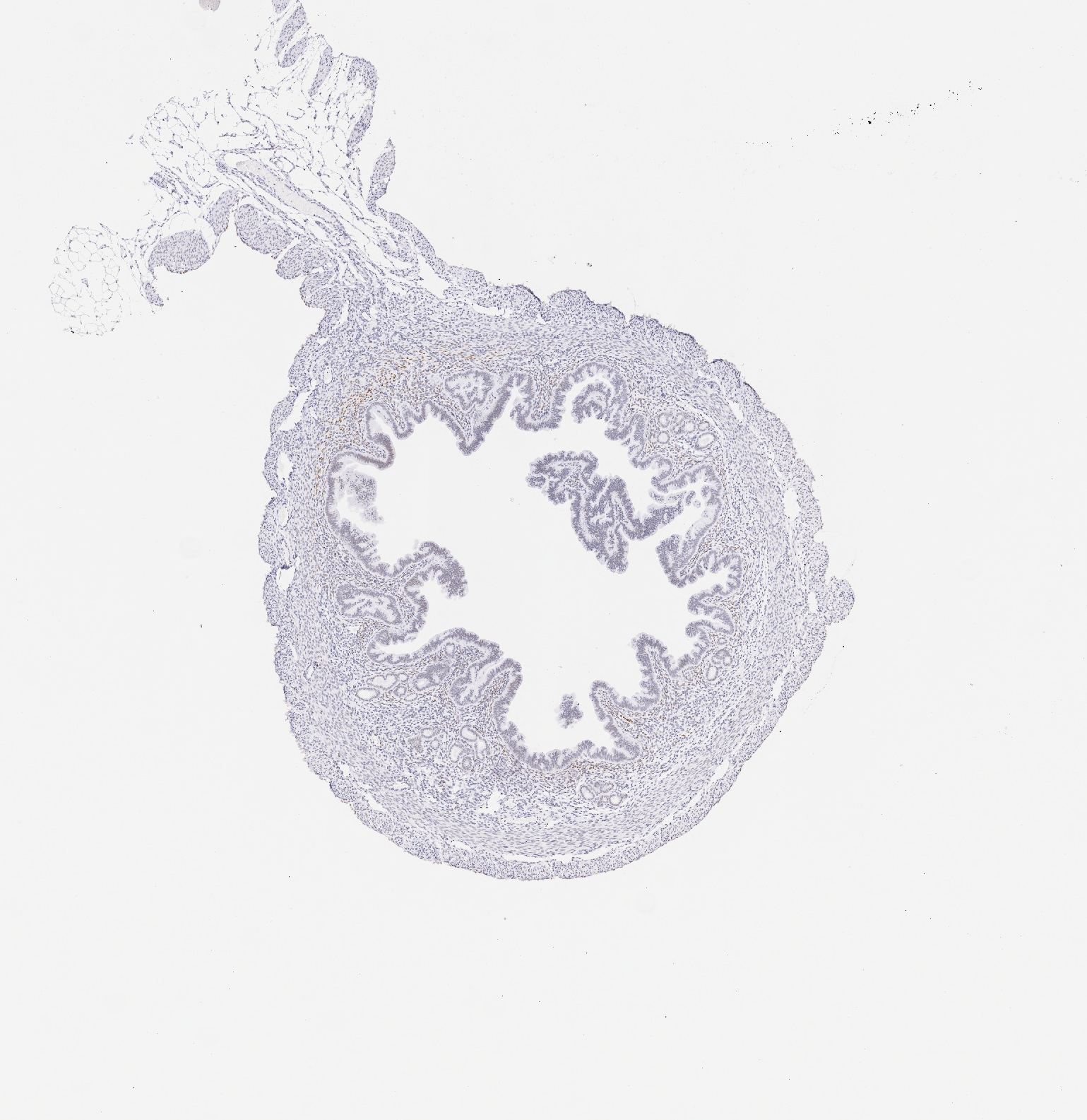

Supplement: Supplementary file 19 — Source Data for Figure 6 [file EMMM-15-e17094-s004.zip › EMM-2022-17094_source_data_figure_6/figure_6B/tbpw 1.1k r482q lef1 4x.jpg]

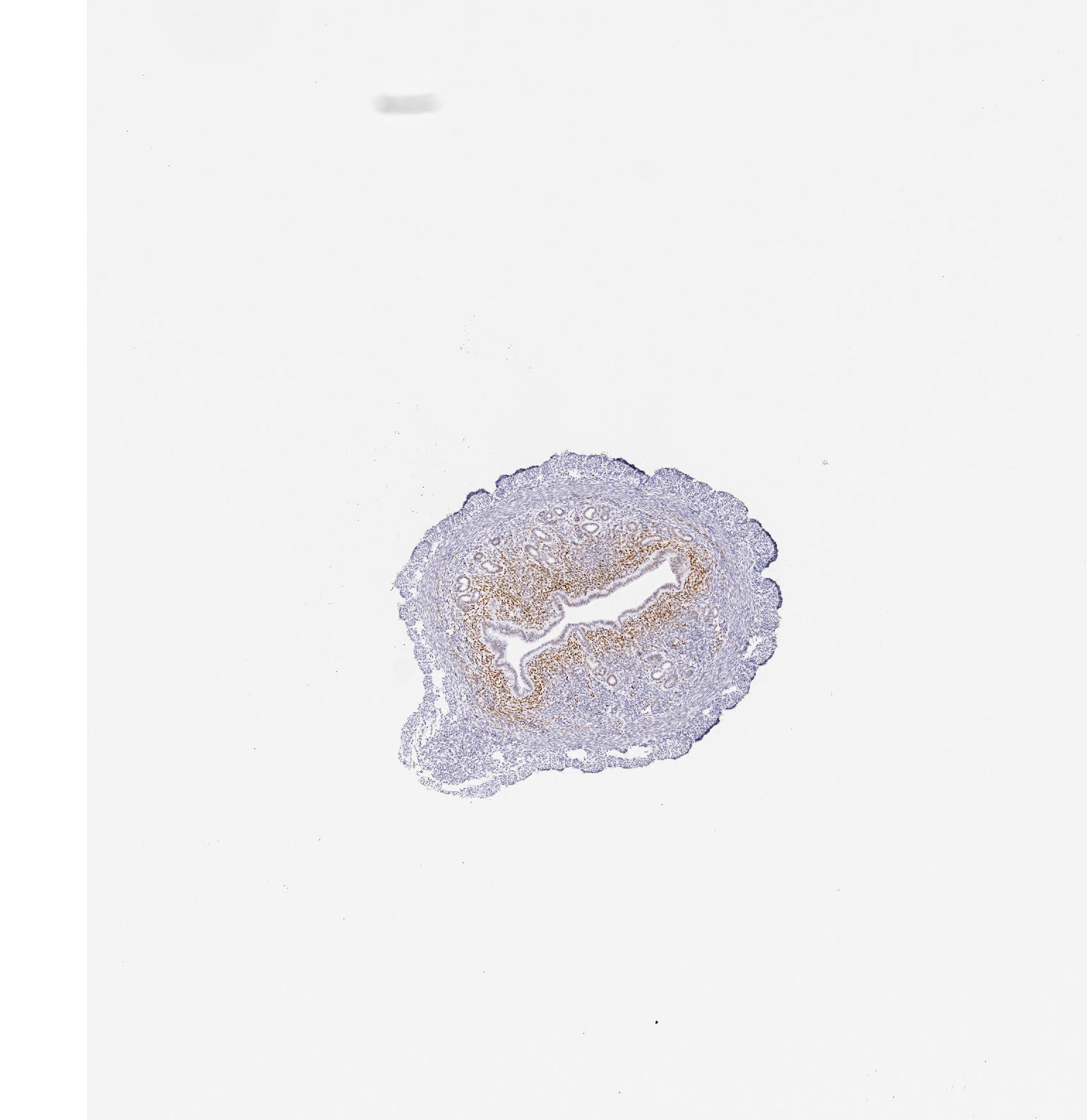

Supplement: Supplementary file 19 — Source Data for Figure 6 [file EMMM-15-e17094-s004.zip › EMM-2022-17094_source_data_figure_6/figure_6B/tbow 13.3d trp53 lef1 4x.jpg]

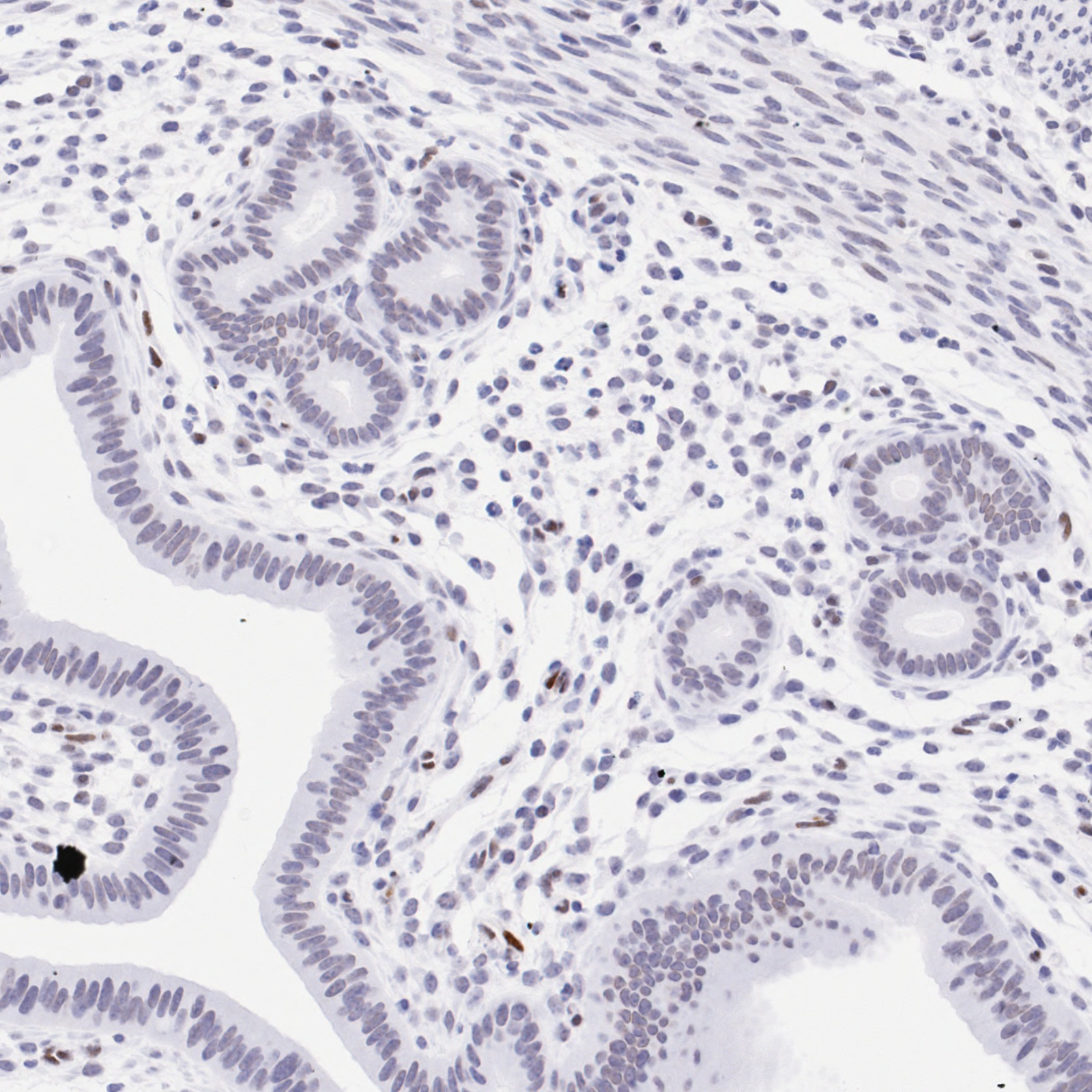

Supplement: Supplementary file 19 — Source Data for Figure 6 [file EMMM-15-e17094-s004.zip › EMM-2022-17094_source_data_figure_6/figure_6B/tprp 16.3f r172h lef1 40x.jpg]

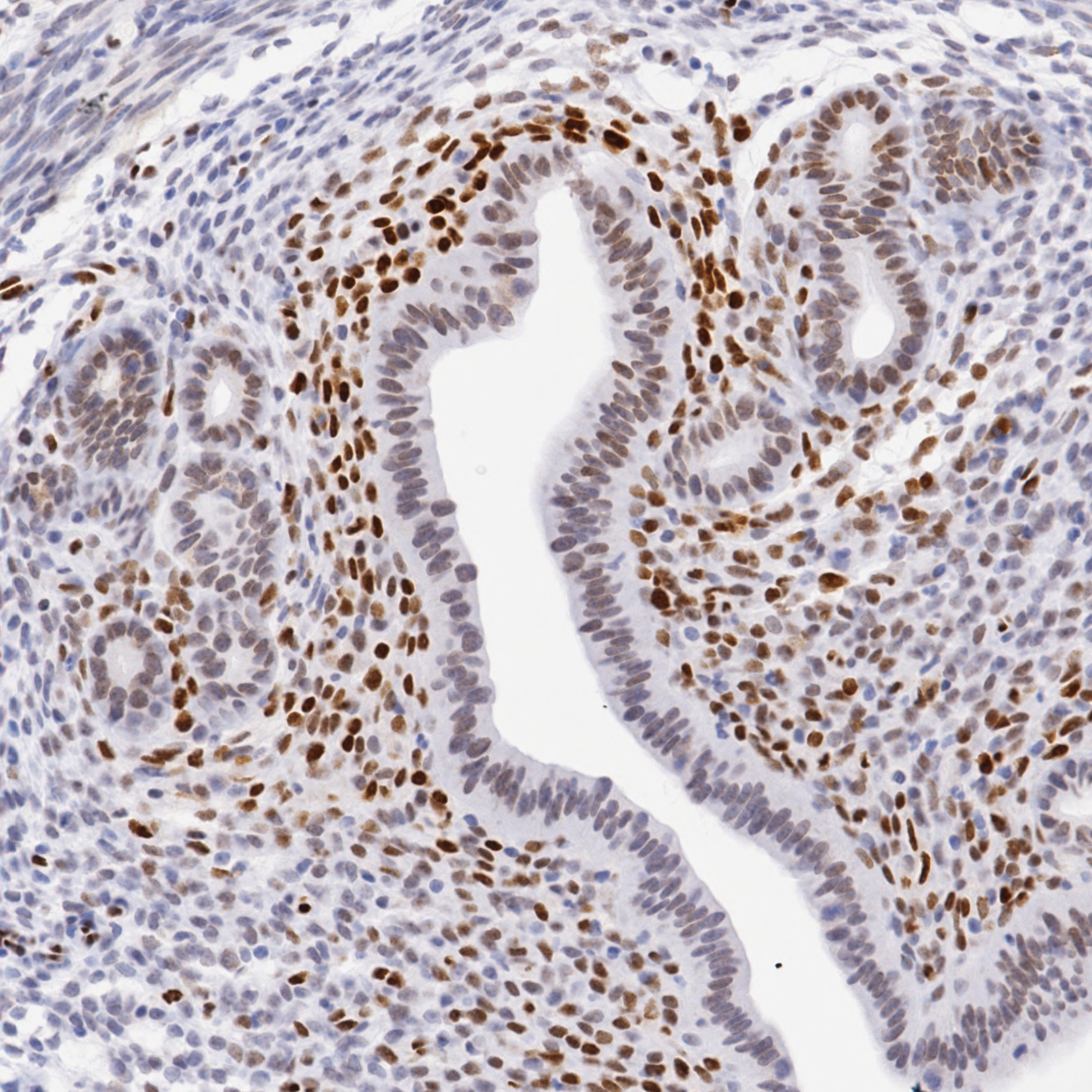

Supplement: Supplementary file 19 — Source Data for Figure 6 [file EMMM-15-e17094-s004.zip › EMM-2022-17094_source_data_figure_6/figure_6B/tprp 16.3i r172hr482q lef1 40x.jpg]

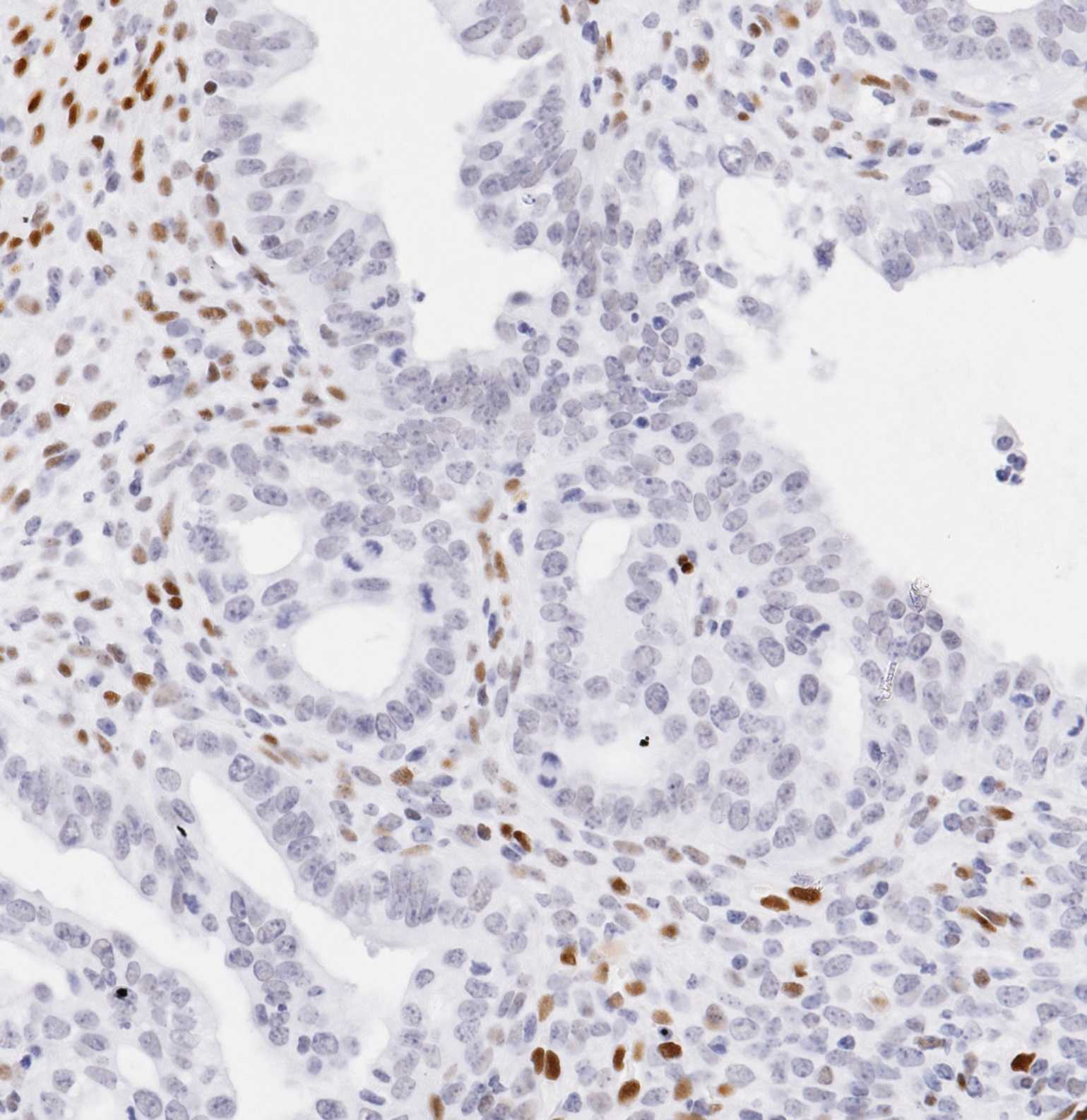

Supplement: Supplementary file 19 — Source Data for Figure 6 [file EMMM-15-e17094-s004.zip › EMM-2022-17094_source_data_figure_6/figure_6B/tbnw 10.1f pten lef1 40x.jpg]

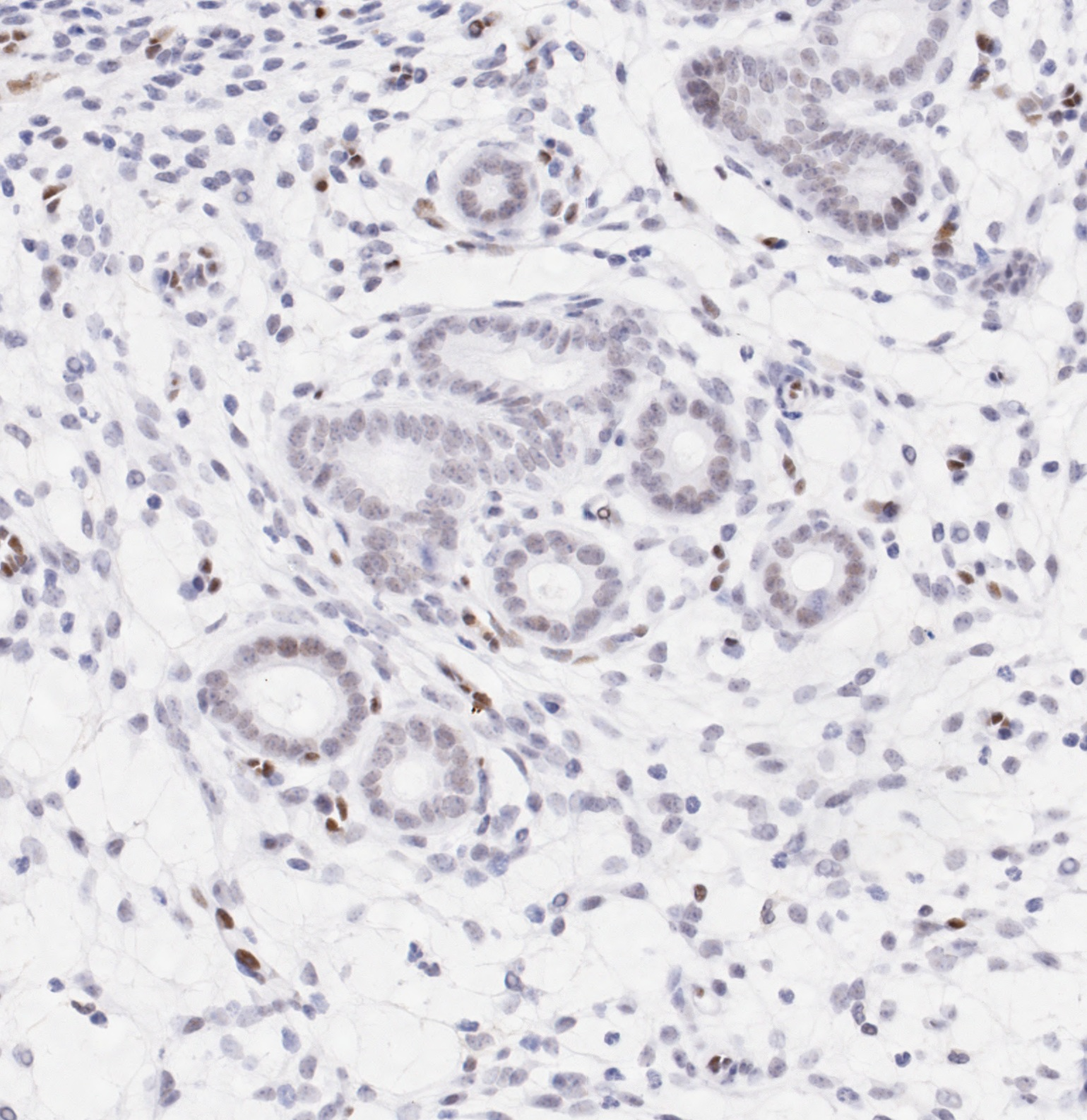

Supplement: Supplementary file 19 — Source Data for Figure 6 [file EMMM-15-e17094-s004.zip › EMM-2022-17094_source_data_figure_6/figure_6B/tbp1 1.1j wt lef1 40x.jpg]

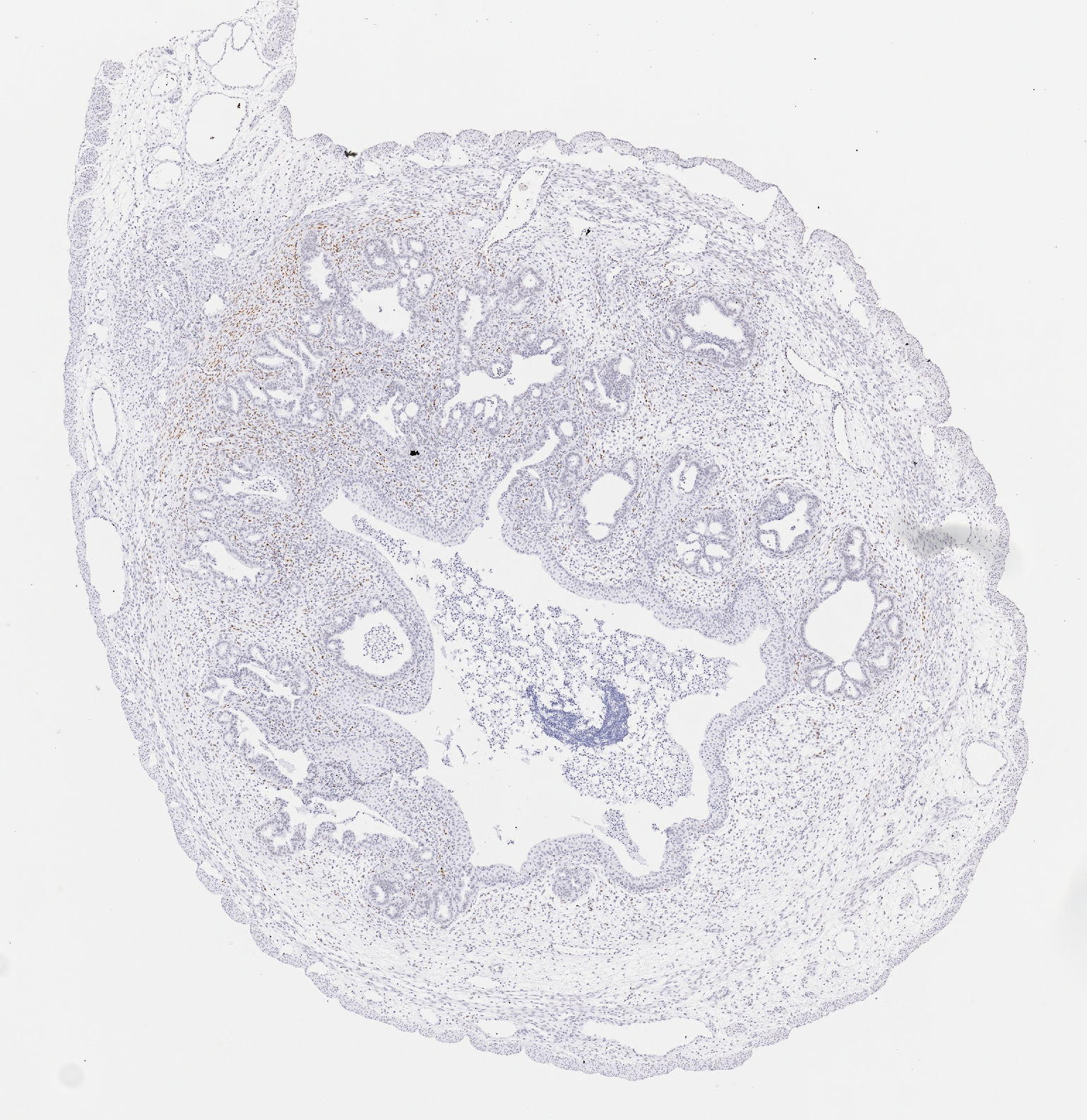

Supplement: Supplementary file 19 — Source Data for Figure 6 [file EMMM-15-e17094-s004.zip › EMM-2022-17094_source_data_figure_6/figure_6B/tbnw 10.1f pten lef1 4x.jpg]

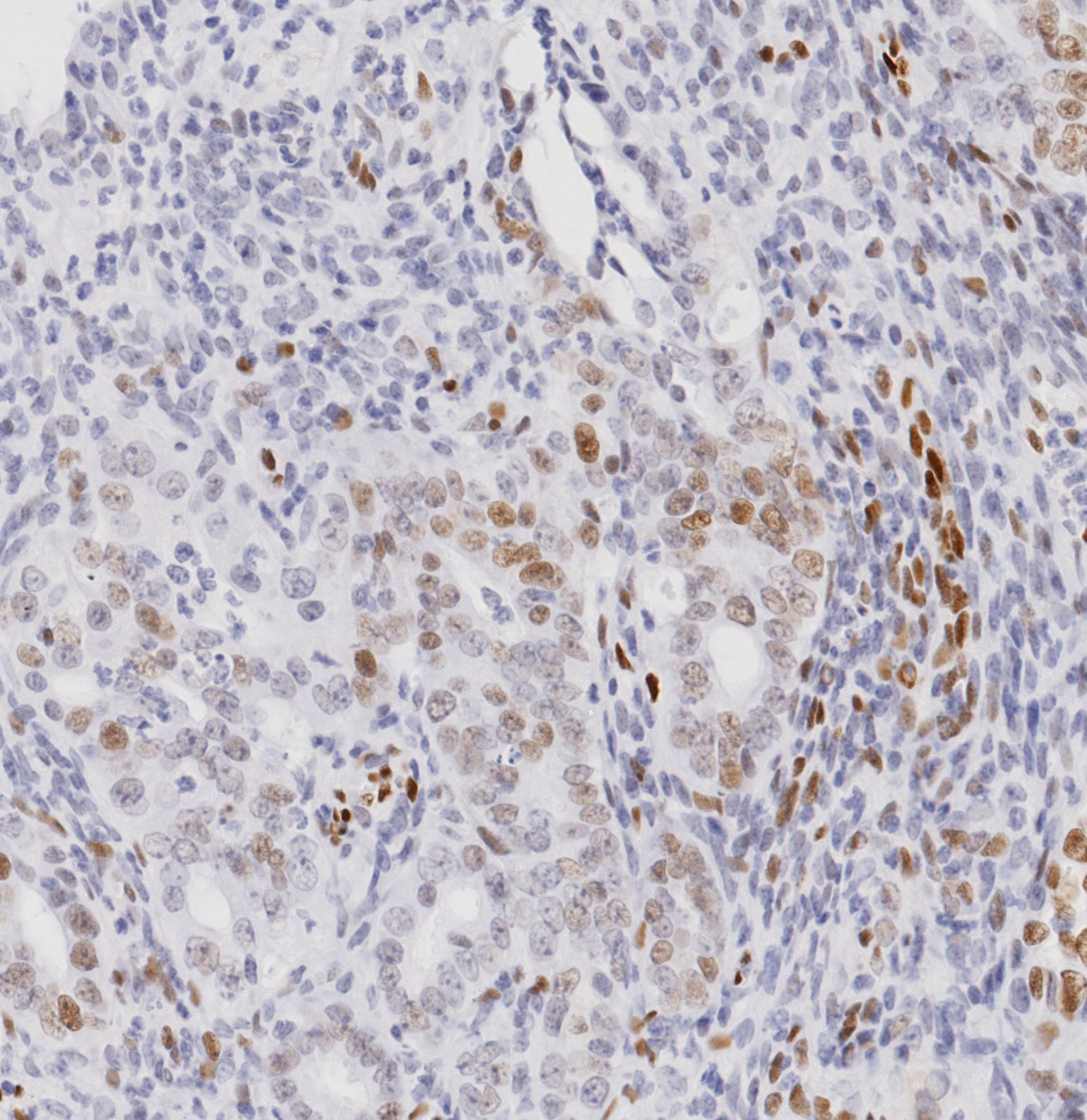

Supplement: Supplementary file 19 — Source Data for Figure 6 [file EMMM-15-e17094-s004.zip › EMM-2022-17094_source_data_figure_6/figure_6B/tbnw 10.1h ptenr482q_lef1_40x.jpg]

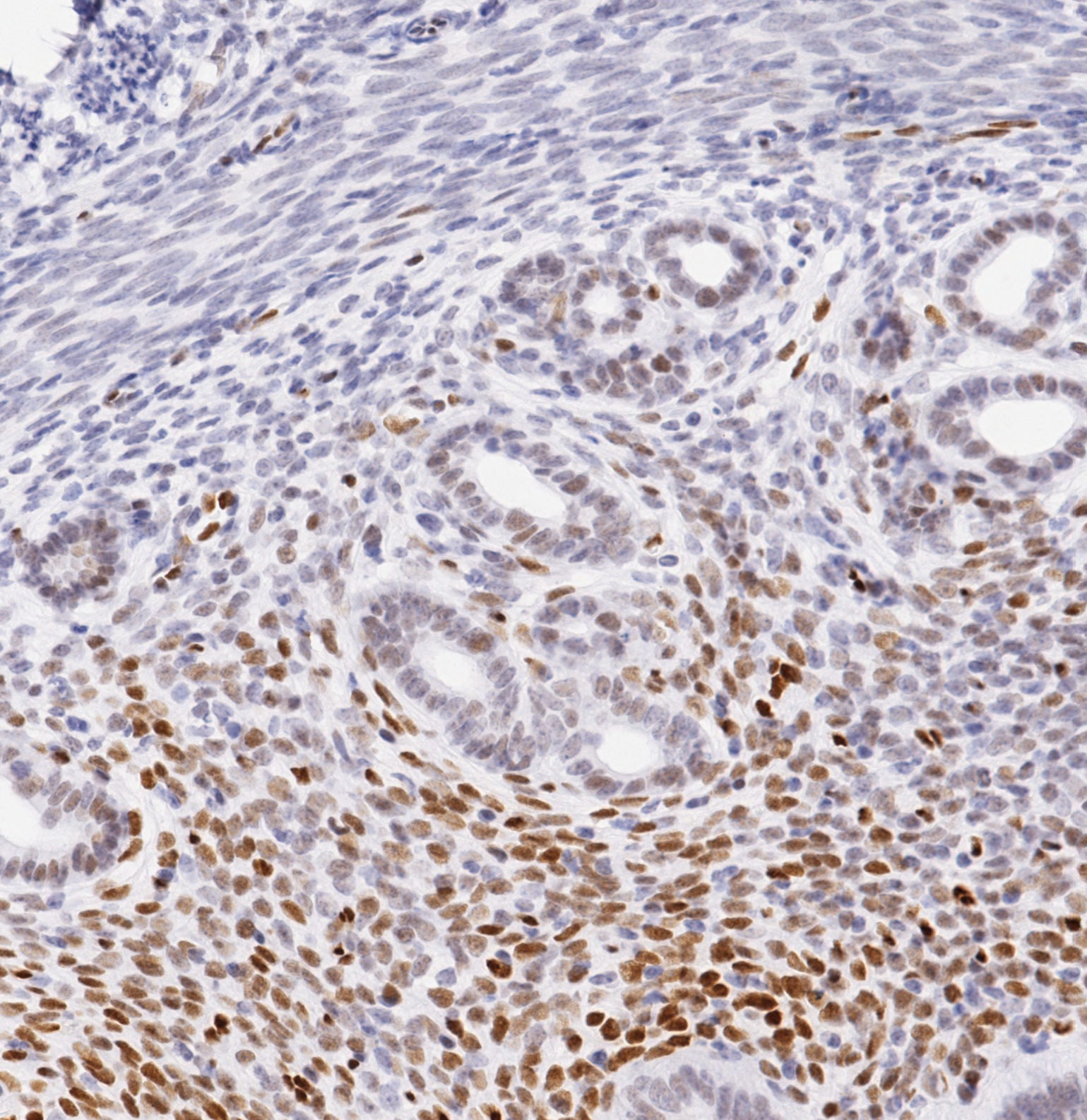

Supplement: Supplementary file 19 — Source Data for Figure 6 [file EMMM-15-e17094-s004.zip › EMM-2022-17094_source_data_figure_6/figure_6B/tbow 13.3d trp53 lef1 40x.jpg]

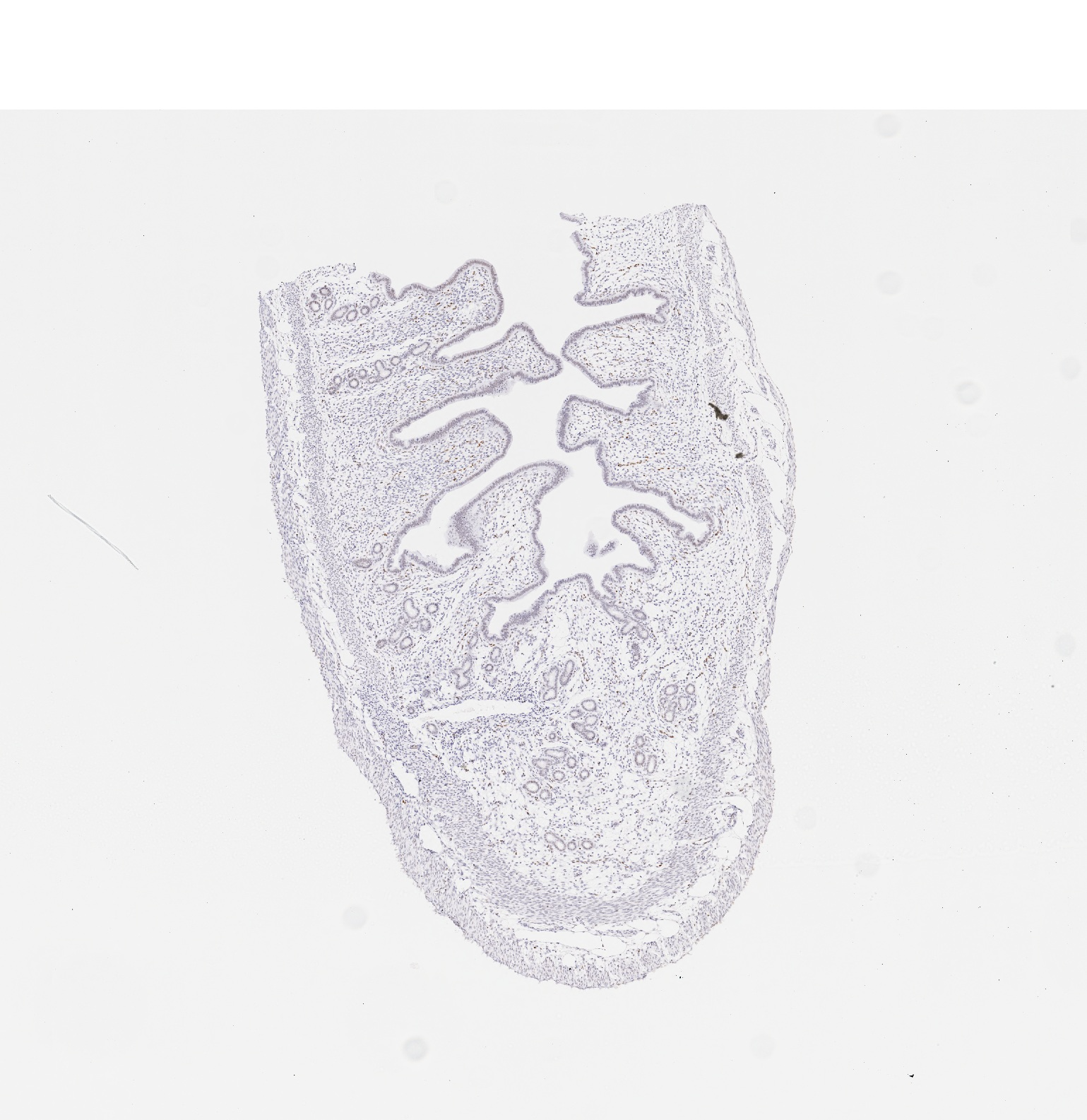

Supplement: Supplementary file 19 — Source Data for Figure 6 [file EMMM-15-e17094-s004.zip › EMM-2022-17094_source_data_figure_6/figure_6B/tbp1 1.1j wt lef1 4x.jpg]

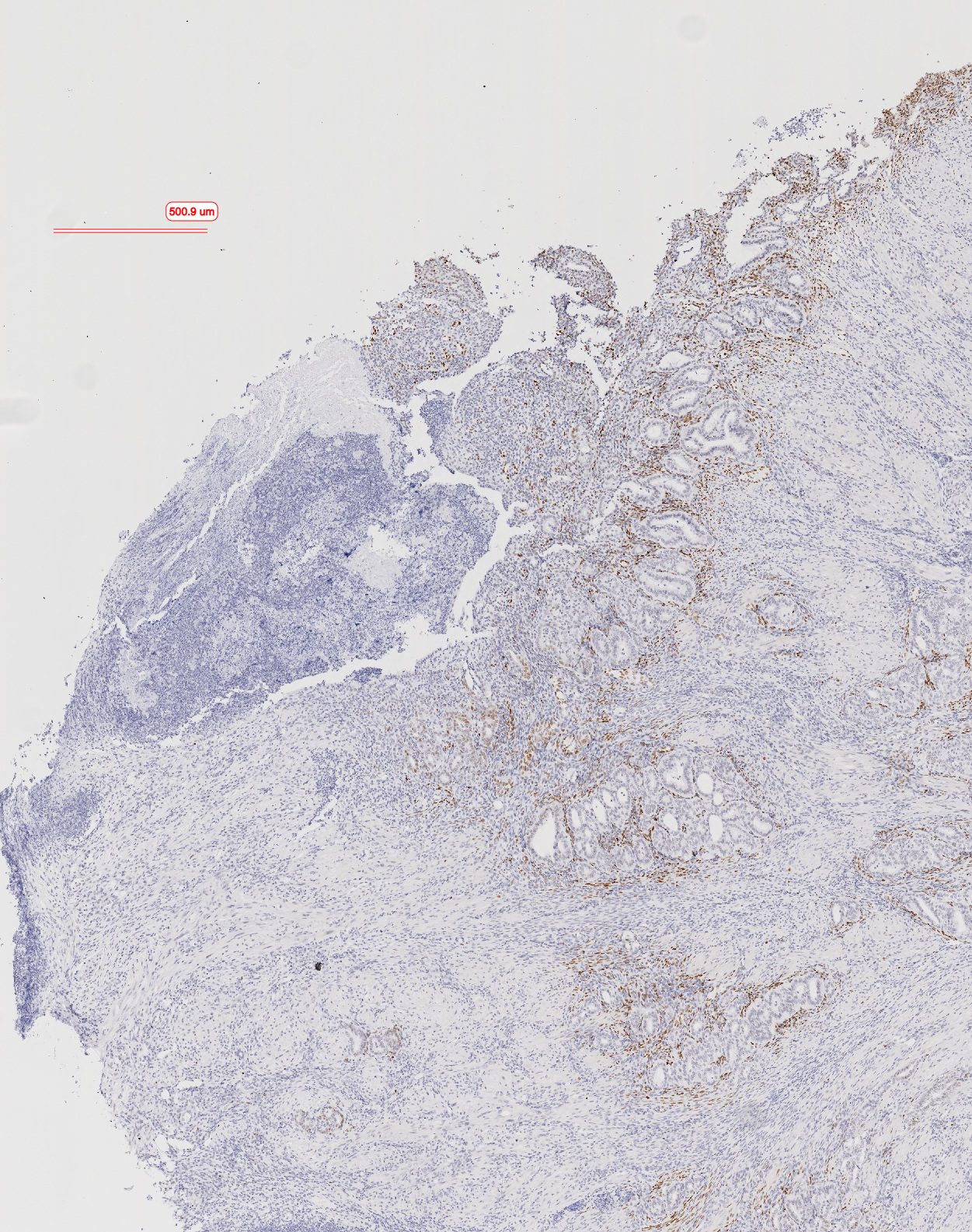

Supplement: Supplementary file 19 — Source Data for Figure 6 [file EMMM-15-e17094-s004.zip › EMM-2022-17094_source_data_figure_6/figure_6B/tbnw 10.1h ptenr482q_lef1_4x.jpg]

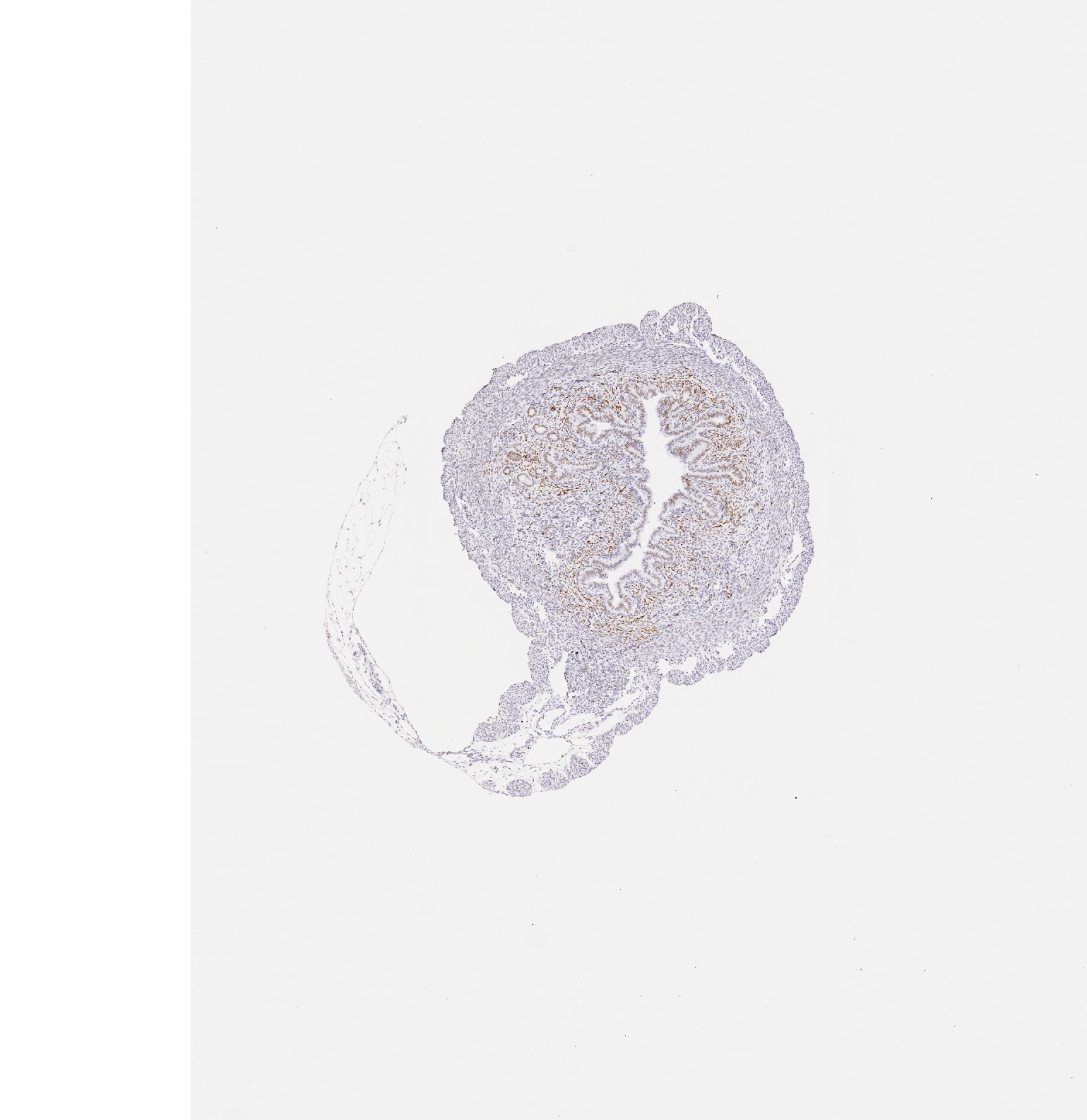

Supplement: Supplementary file 19 — Source Data for Figure 6 [file EMMM-15-e17094-s004.zip › EMM-2022-17094_source_data_figure_6/figure_6B/tbow 13.3f trp53r 482q lef1 4x.jpg]

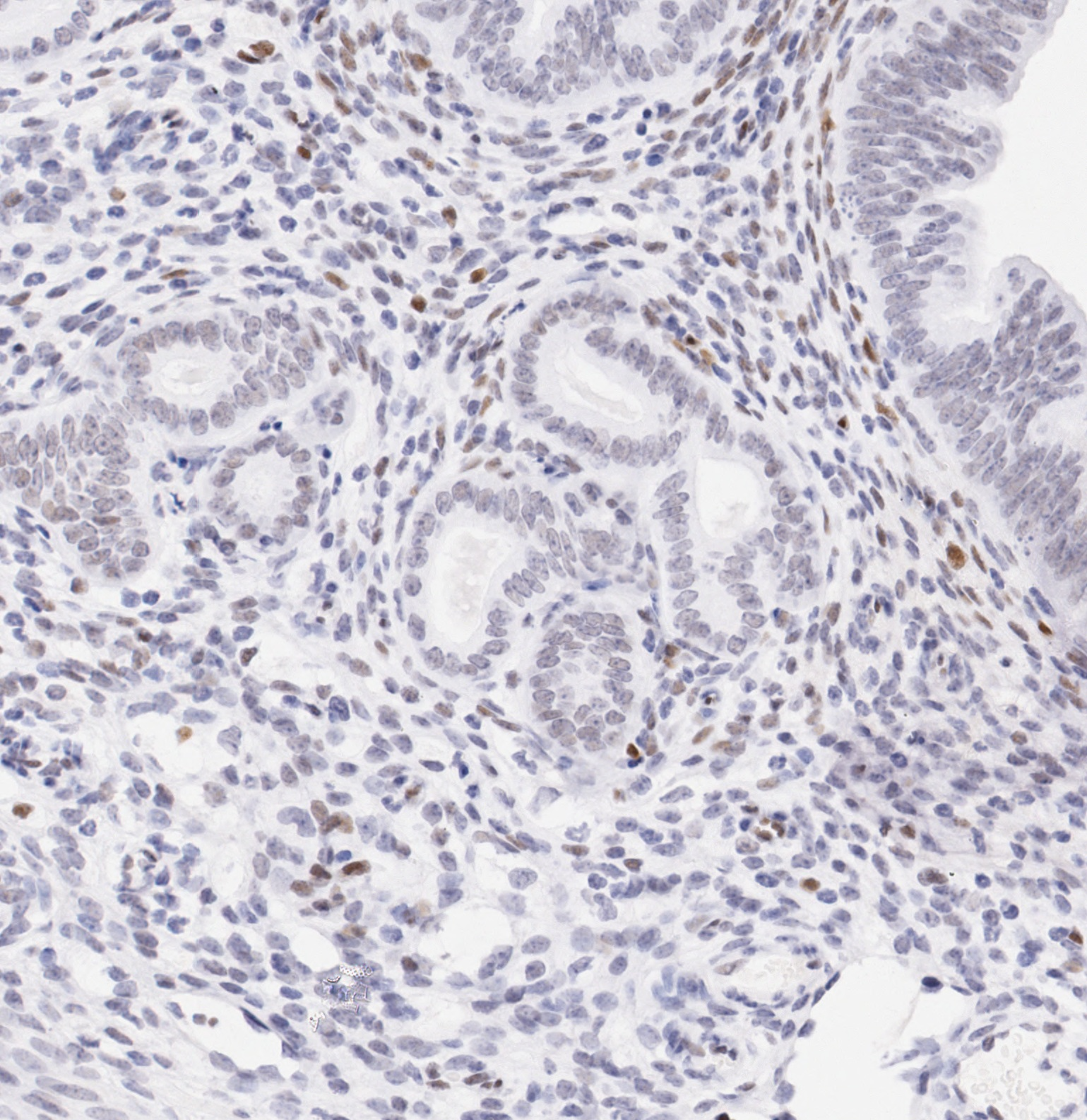

Supplement: Supplementary file 19 — Source Data for Figure 6 [file EMMM-15-e17094-s004.zip › EMM-2022-17094_source_data_figure_6/figure_6B/tbpw 1.1k r482q lef1 40x.jpg]

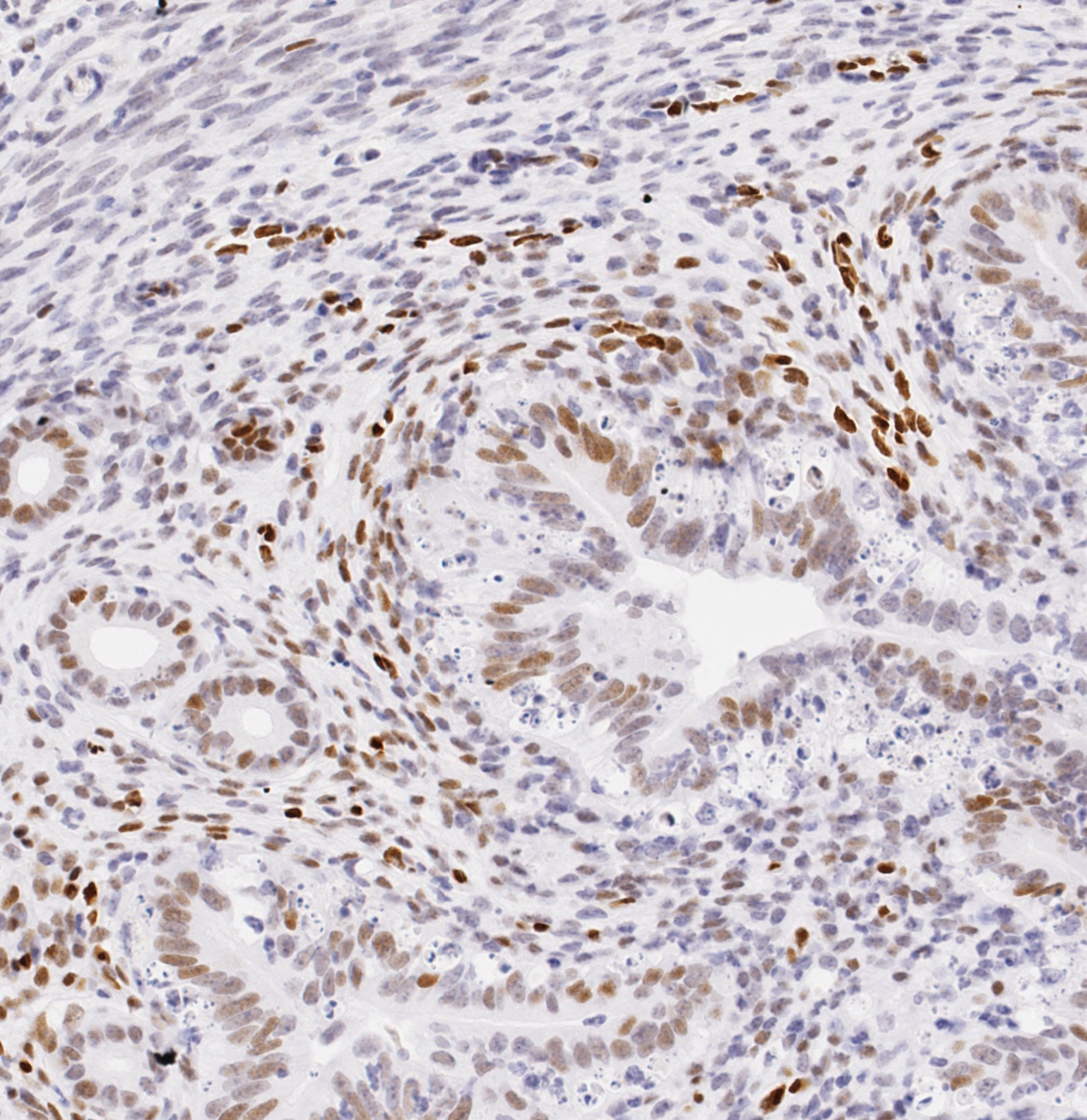

Supplement: Supplementary file 19 — Source Data for Figure 6 [file EMMM-15-e17094-s004.zip › EMM-2022-17094_source_data_figure_6/figure_6B/tbow 13.3f trp53 r482q lef1 40x.jpg]

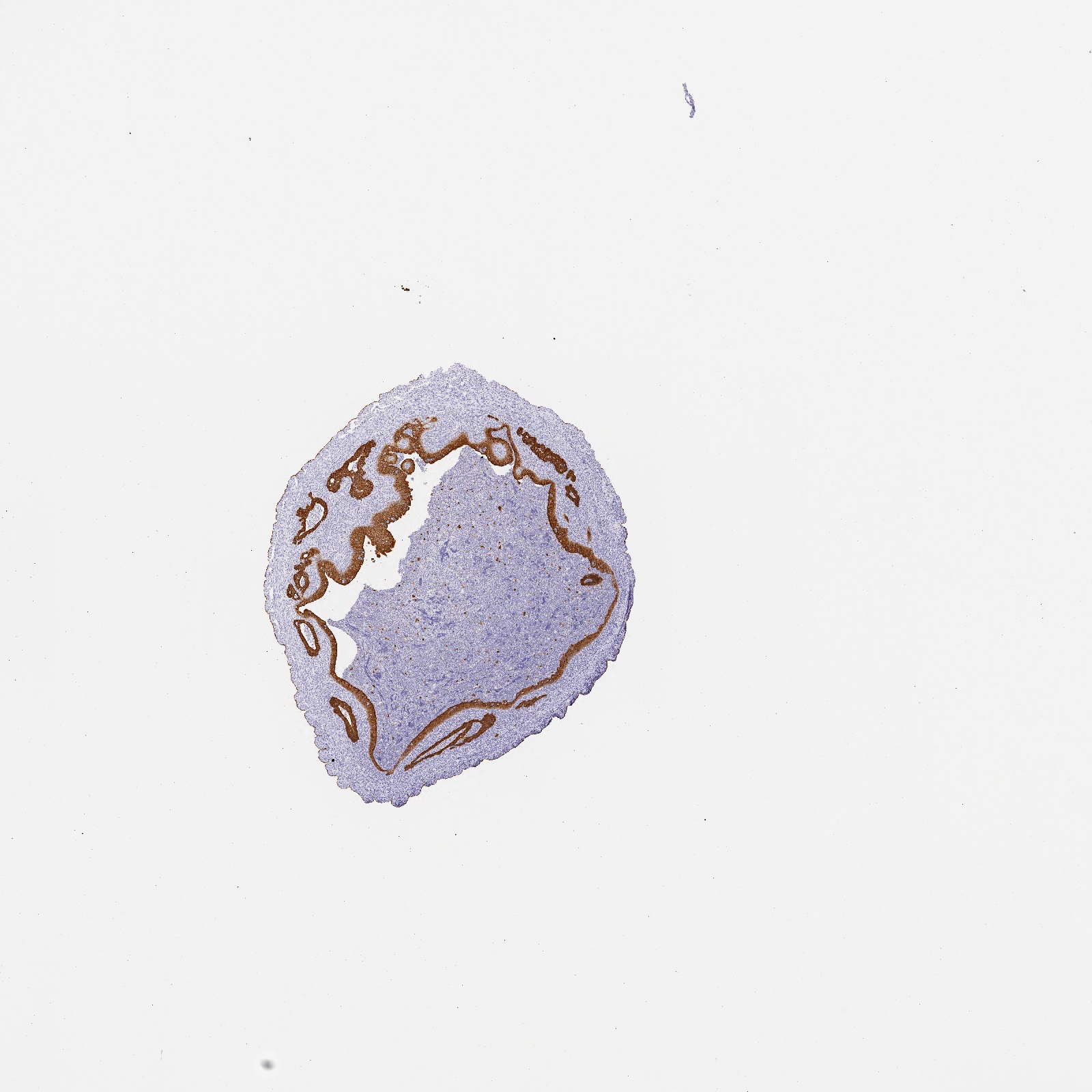

Supplement: Supplementary file 19 — Source Data for Figure 6 [file EMMM-15-e17094-s004.zip › EMM-2022-17094_source_data_figure_6/figure_6D/ptendel_ck8 @2x.jpg]

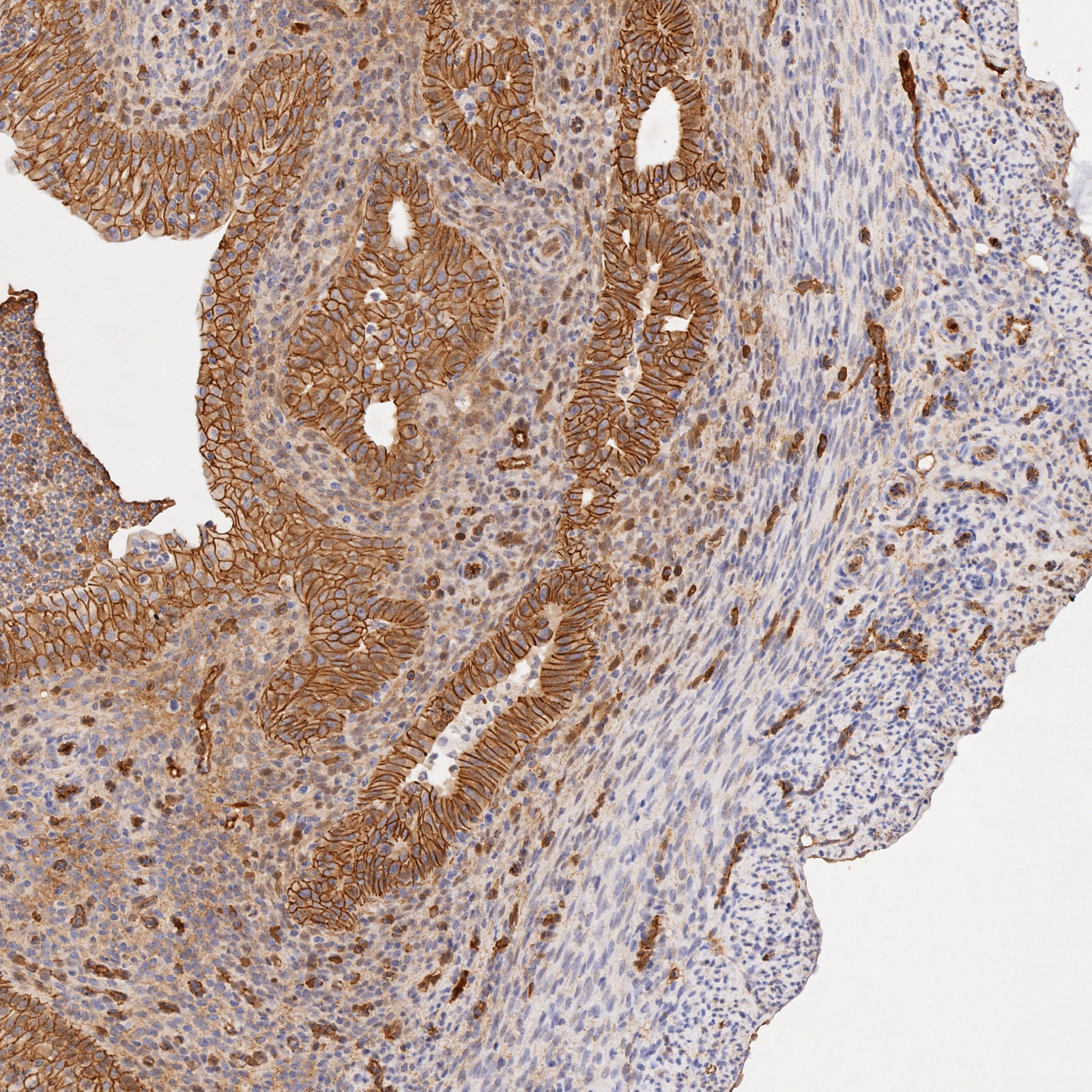

Supplement: Supplementary file 19 — Source Data for Figure 6 [file EMMM-15-e17094-s004.zip › EMM-2022-17094_source_data_figure_6/figure_6D/ptendel_bcat in lef1 neg glands @20x.png]

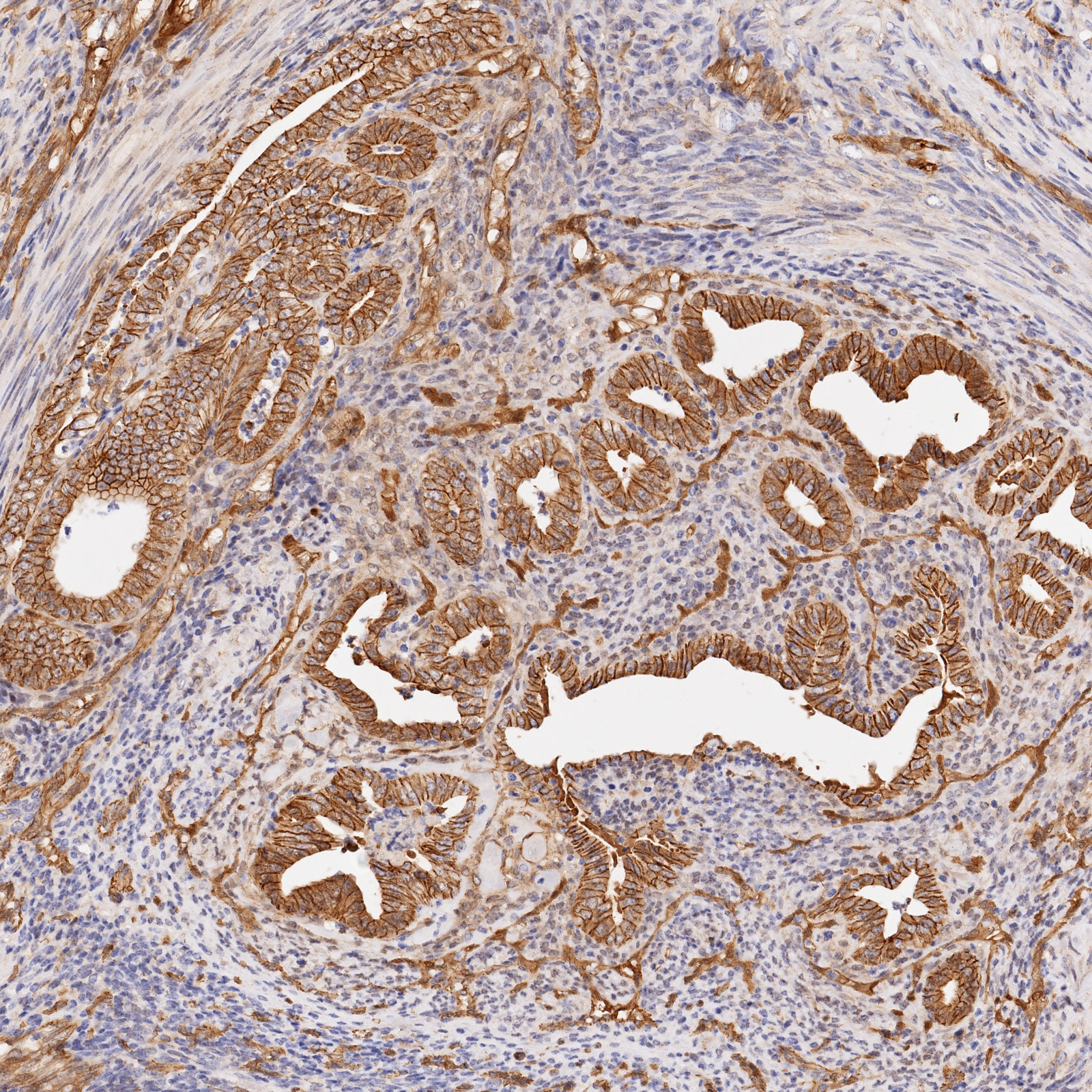

Supplement: Supplementary file 19 — Source Data for Figure 6 [file EMMM-15-e17094-s004.zip › EMM-2022-17094_source_data_figure_6/figure_6D/ptendel_fbxw7mut_bcat in lef1 pos region @20x.jpg]

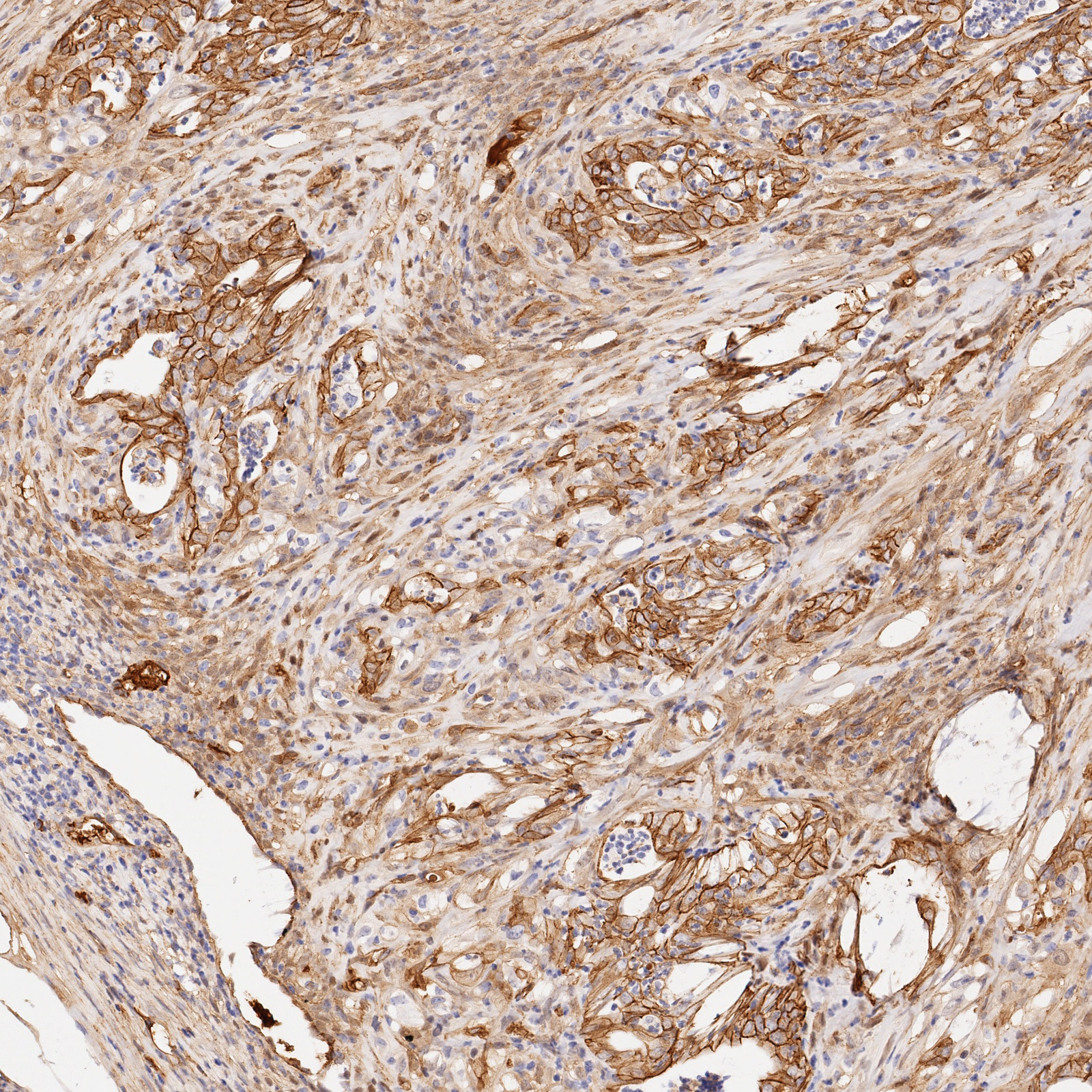

Supplement: Supplementary file 19 — Source Data for Figure 6 [file EMMM-15-e17094-s004.zip › EMM-2022-17094_source_data_figure_6/figure_6D/ptendel_fbxw7mut_bcat in lef1 neg region @20x.jpg]

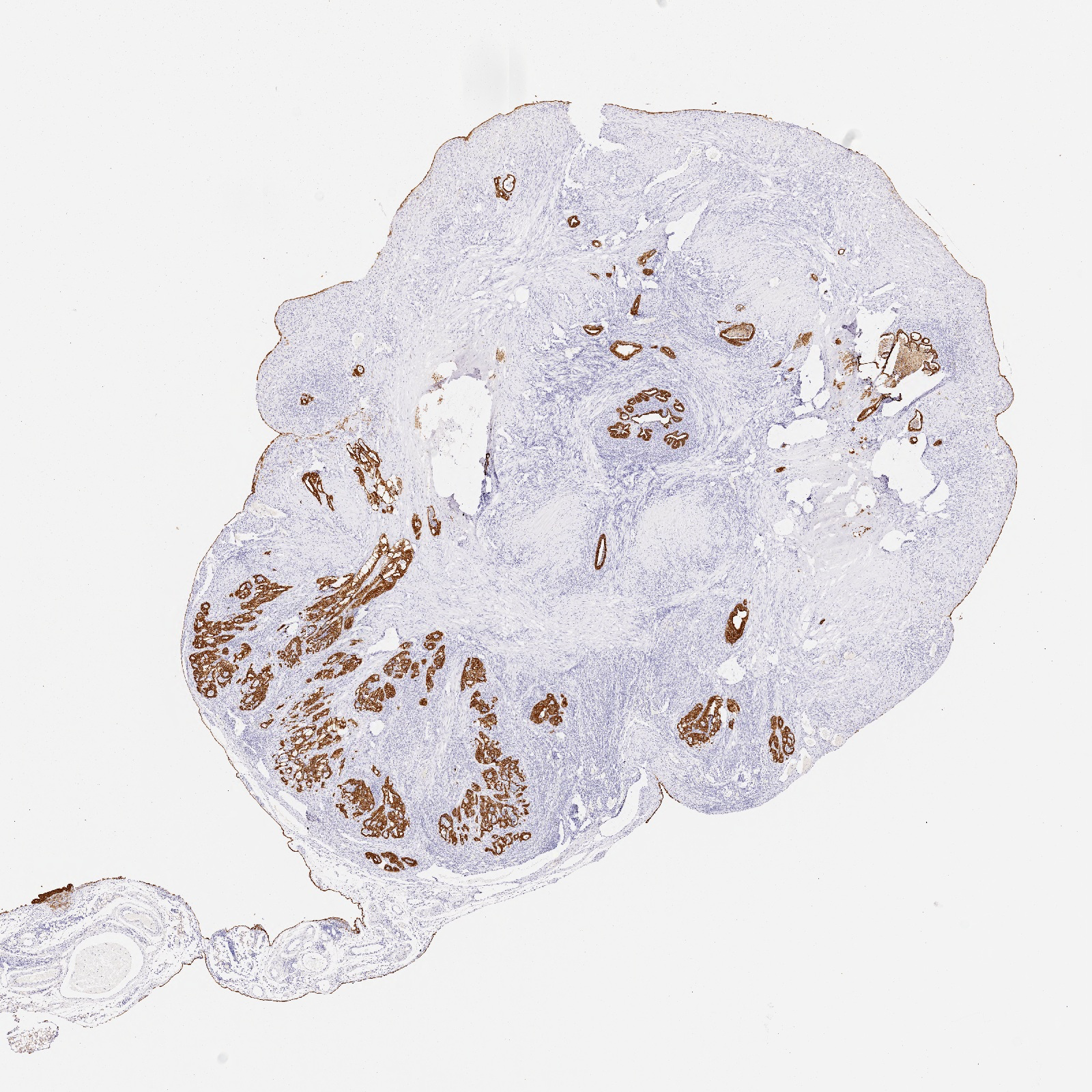

Supplement: Supplementary file 19 — Source Data for Figure 6 [file EMMM-15-e17094-s004.zip › EMM-2022-17094_source_data_figure_6/figure_6D/ptendel_fbxw7mut_ck8 @2x.jpg]

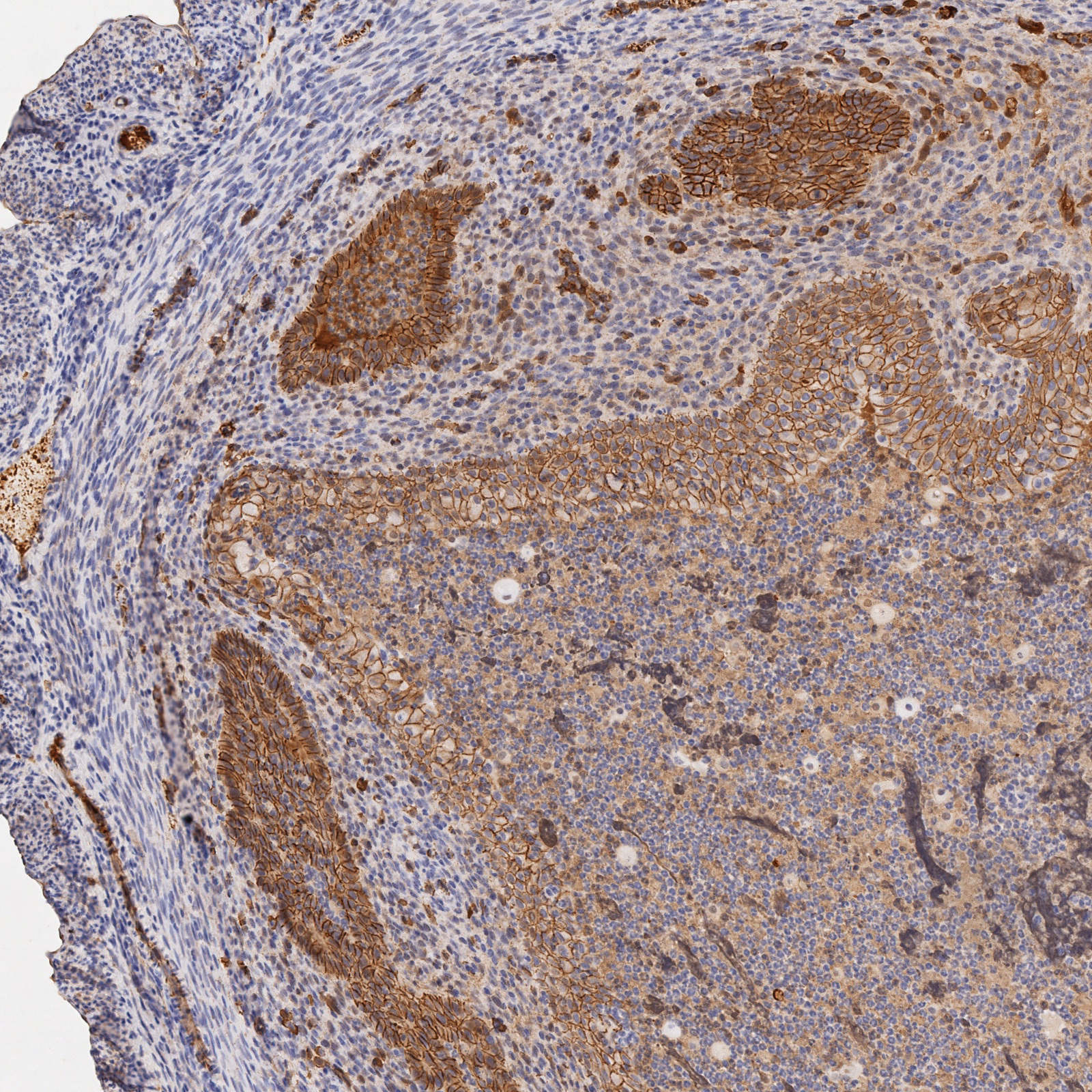

Supplement: Supplementary file 19 — Source Data for Figure 6 [file EMMM-15-e17094-s004.zip › EMM-2022-17094_source_data_figure_6/figure_6D/ptendel_bcat in lef1 pos glands @20x.png]

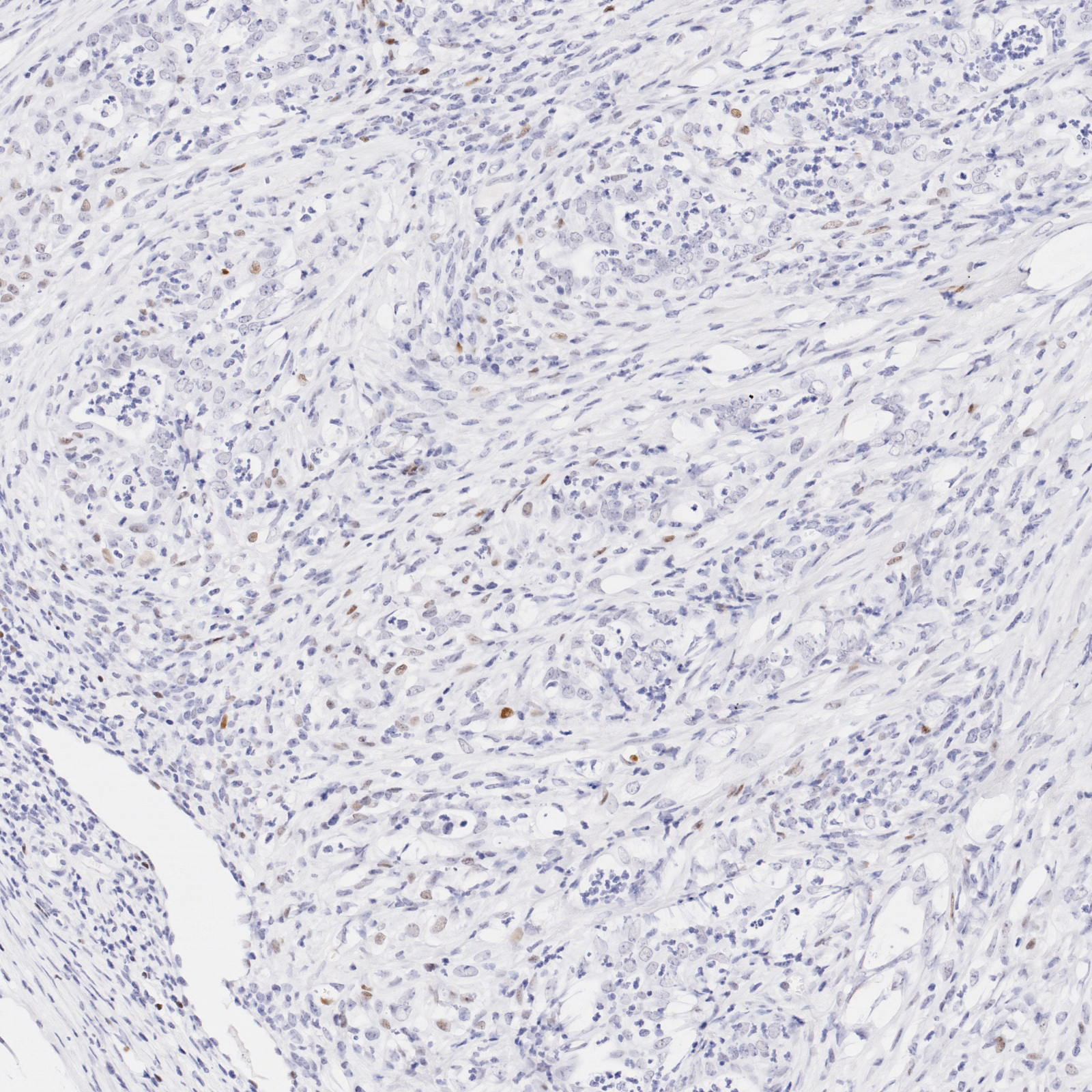

Supplement: Supplementary file 19 — Source Data for Figure 6 [file EMMM-15-e17094-s004.zip › EMM-2022-17094_source_data_figure_6/figure_6D/ptendel_fbxw7mut_lef1 in lef1 neg region @20x.jpg]

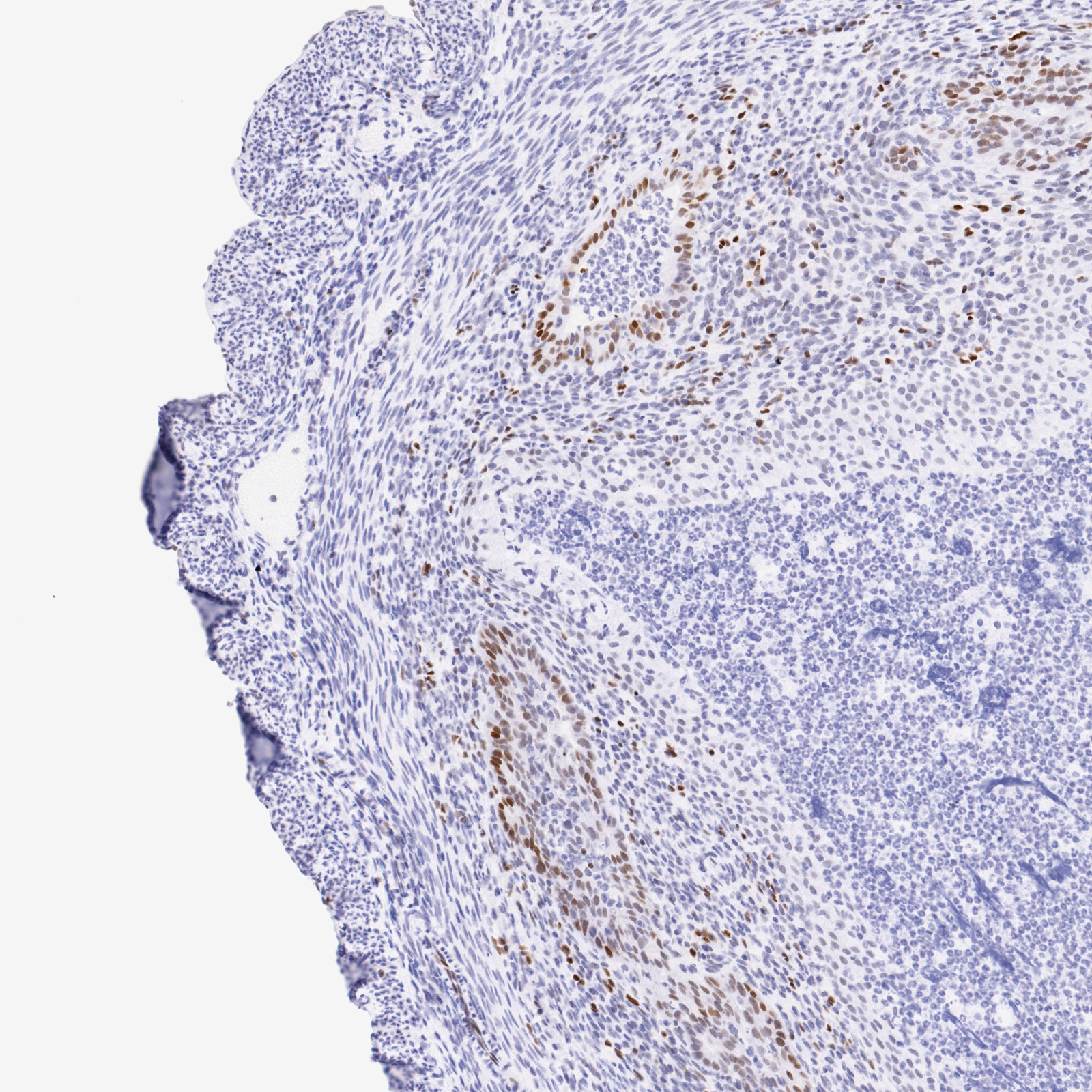

Supplement: Supplementary file 19 — Source Data for Figure 6 [file EMMM-15-e17094-s004.zip › EMM-2022-17094_source_data_figure_6/figure_6D/ptendel_lef1 in lef1 pos glands @20x.jpg]

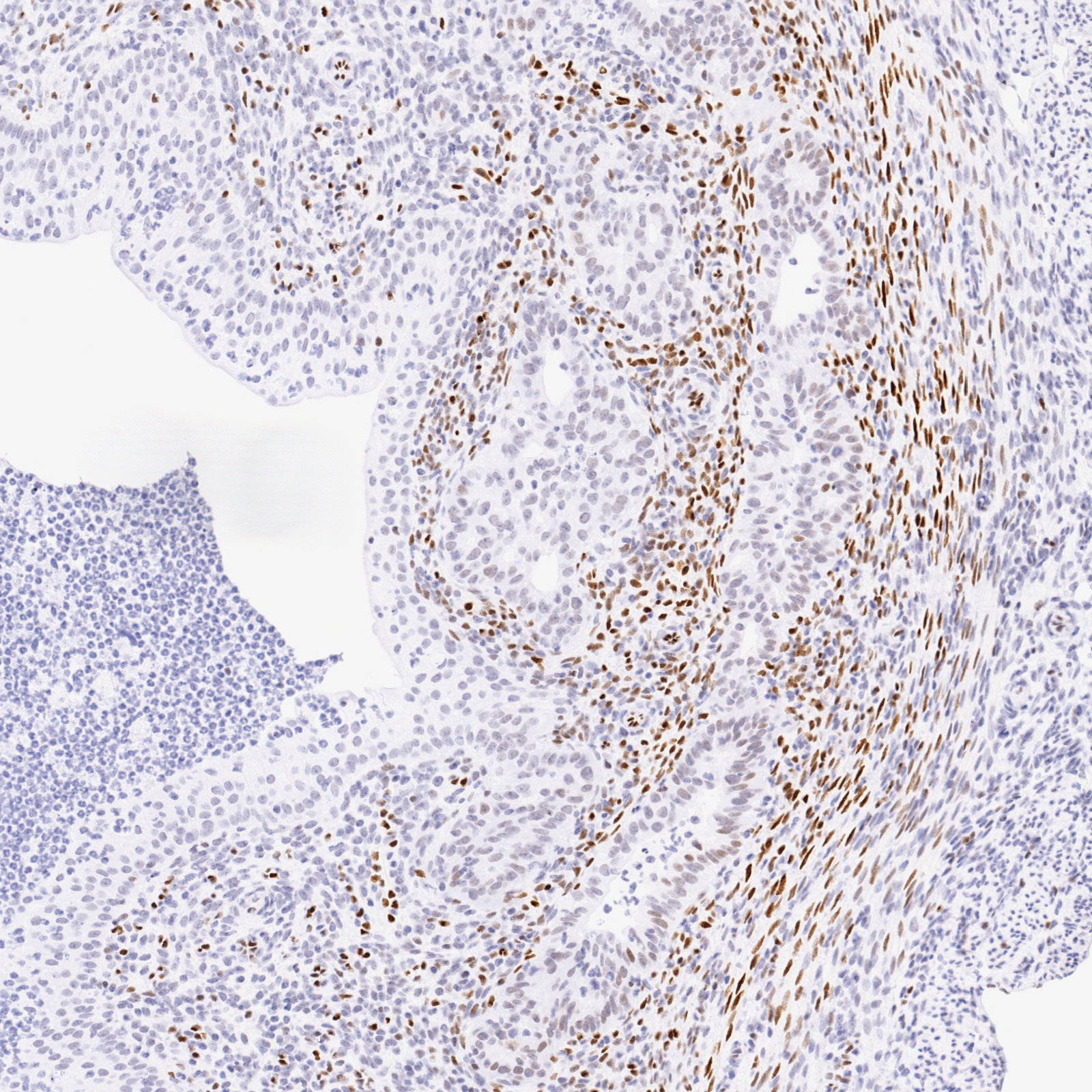

Supplement: Supplementary file 19 — Source Data for Figure 6 [file EMMM-15-e17094-s004.zip › EMM-2022-17094_source_data_figure_6/figure_6D/ptendel_lef1 in lef1 neg glands @20x.jpg]

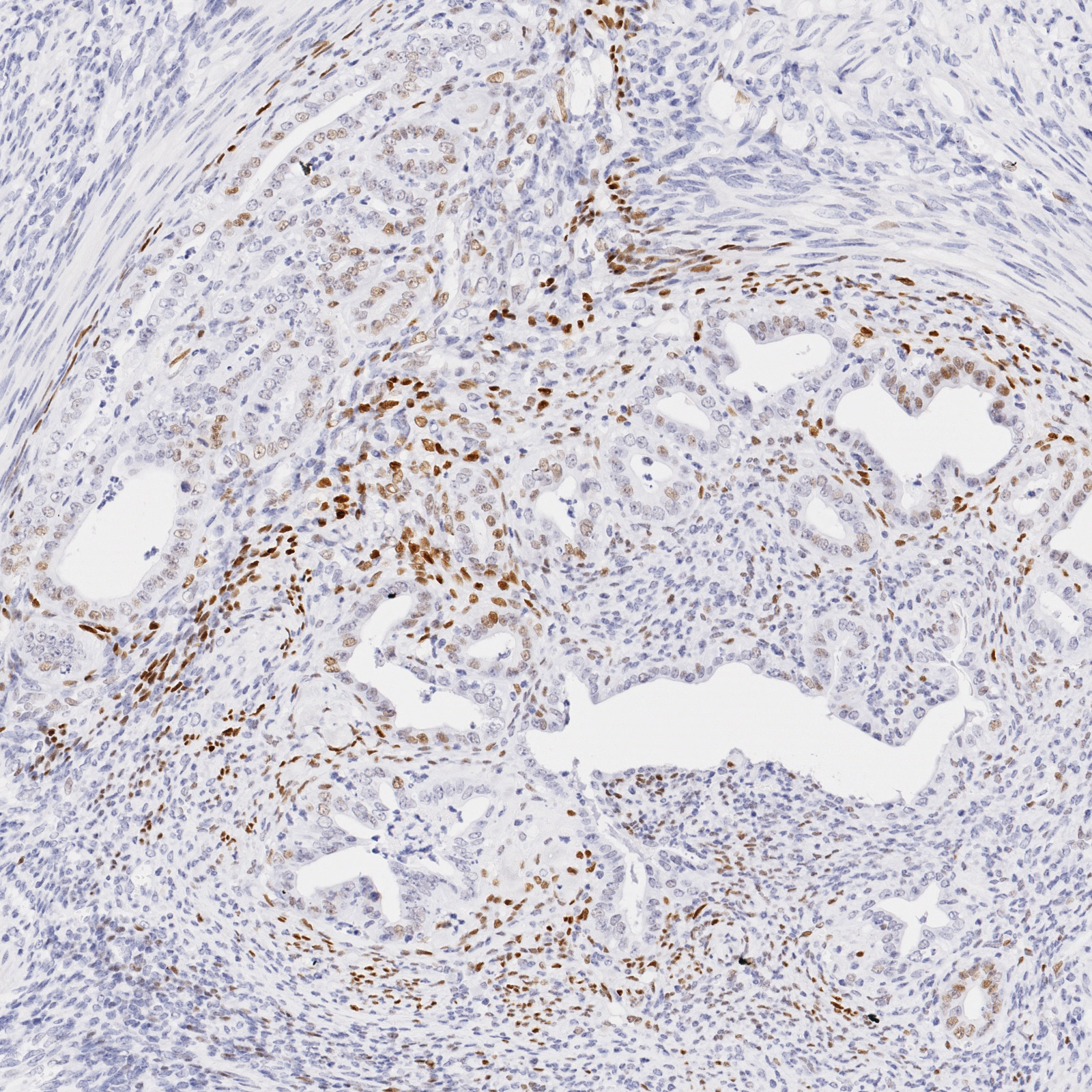

Supplement: Supplementary file 19 — Source Data for Figure 6 [file EMMM-15-e17094-s004.zip › EMM-2022-17094_source_data_figure_6/figure_6D/ptendel_fbxw7mut_lef1 in lef1 pos region @20x.jpg]
