# Supplementary material for: Functional analysis reveals driver cooperativity and novel mechanisms in endometrial carcinogenesis
Source: EMBO Mol Med. 2023 Aug 17;15(10):e17094. doi: 10.15252/emmm.202217094 (PMC10565641; doi:10.15252/emmm.202217094)
Supplement: Supplementary file 20 [file EMMM-15-e17094-s017.pdf]

EMBO Molecular Medicine

Functional analysis reveals driver cooperativity and novel mechanisms in endometrial carcinogenesis

Matthew Brown, Alicia Leon, Katarzyna Kedzierska, Charlotte Moore, Hayley Belnoue-Davis, Susanne Flach, John Lydon, Francesco deMayo, Annabelle Lewis, Tjalling Bosse, Ian Tomlinson, and David Church
DOI: 10.15252/emmm.202217094

Corresponding author: David Church (david.church@well.ox.ac.uk)

Review Timeline:

Submission Date:	24th Oct 22
Editorial Decision:	23rd Nov 22
Revision Received:	13th May 23
Editorial Decision:	9th Jun 23
Revision Received:	1st Aug 23
Accepted:	2nd Aug 23

Editor: Lise Roth

Transaction Report:

(Note: With the exception of the correction of typographical or spelling errors that could be a source of ambiguity, letters and reports are not edited. Depending on transfer agreements, referee reports obtained elsewhere may or may not be included in this compilation. Referee reports are anonymous unless the Referee chooses to sign their reports.)

23rd Nov 2022

Dear Dr. Church,

Thank you for the submission of your manuscript to EMBO Molecular Medicine. We have now received feedback from the three reviewers who agreed to evaluate your manuscript. As you will see from the reports below, the referees acknowledge the interest of the study and are overall supporting publication of your work pending appropriate revisions.

Addressing the reviewers' concerns in full will be necessary for further considering the manuscript in our journal, and acceptance of the manuscript will entail a second round of review. EMBO Molecular Medicine encourages a single round of revision only and therefore, acceptance or rejection of the manuscript will depend on the completeness of your responses included in the next, final version of the manuscript. For this reason, and to save you from any frustrations in the end, I would strongly advise against returning an incomplete revision.

If you would like to discuss further the points raised by the referee, I am available to do so via email or video. Let me know if you are interested in this option.

Revised manuscripts should be submitted within three months of a request for revision; they will otherwise be treated as new submissions, except under exceptional circumstances in which a short extension is obtained from the editor.

When submitting your revised manuscript, please carefully review the instructions that follow below. We perform an initial quality control of all revised manuscripts before re-review; failure to include requested items will delay the evaluation of your revision.

We require:

1) A .docx formatted version of the manuscript text (including legends for main figures, EV figures and tables). Please make sure that the changes are highlighted to be clearly visible.

2) Individual production quality figure files as .eps, .tif, .jpg (one file per figure). For guidance, download the 'Figure Guide PDF' (https://www.embopress.org/page/journal/17574684/authorguide#figureformat).

3) At EMBO Press we ask authors to provide source data for the main and EV figures. Our source data coordinator will contact you to discuss which figure panels we would need source data for and will also provide you with helpful tips on how to upload and organize the files.
Numerical data should be provided as individual .xls or .csv files (including a tab describing the data). For blots or microscopy, uncropped images should be submitted (using a zip archive if multiple images need to be supplied for one panel). Additional information on source data and instruction on how to label the files are available at
.

4) A .docx formatted letter INCLUDING the reviewers' reports and your detailed point-by-point responses to their comments. As part of the EMBO Press transparent editorial process, the point-by-point response is part of the Review Process File (RPF), which will be published alongside your paper.

5) A complete author checklist, which you can download from our author guidelines (https://www.embopress.org/page/journal/17574684/authorguide#submissionofrevisions). Please insert information in the checklist that is also reflected in the manuscript. The completed author checklist will also be part of the RPF.

6) It is mandatory to include a 'Data Availability' section after the Materials and Methods. Before submitting your revision, primary datasets produced in this study need to be deposited in an appropriate public database, and the accession numbers and database listed under 'Data Availability'. Please remember to provide a reviewer password if the datasets are not yet public (see https://www.embopress.org/page/journal/17574684/authorguide#dataavailability).
Note that the Data Availability Section is restricted to new primary data that are part of this study.

7) For data quantification: please specify the name of the statistical test used to generate error bars and P values, the number (n) of independent experiments (specify technical or biological replicates) underlying each data point and the test used to calculate p-values in each figure legend. The figure legends should contain a basic description of n, P and the test applied. Graphs must include a description of the bars and the error bars (s.d., s.e.m.). Please provide exact p values.

8) Our journal encourages inclusion of *data citations in the reference list* to directly cite datasets that were re-used and obtained from public databases. Data citations in the article text are distinct from normal bibliographical citations and should directly link to the database records from which the data can be accessed. In the main text, data citations are formatted as follows: "Data ref: Smith et al, 2001" or "Data ref: NCBI Sequence Read Archive PRJNA342805, 2017". In the Reference list,

data citations must be labeled with "[DATASET]". A data reference must provide the database name, accession number/identifiers and a resolvable link to the landing page from which the data can be accessed at the end of the reference. Further instructions are available at .

9) We replaced Supplementary Information with Expanded View (EV) Figures and Tables that are collapsible/expandable online. A maximum of 5 EV Figures can be typeset. EV Figures should be cited as 'Figure EV1, Figure EV2" etc... in the text and their respective legends should be included in the main text after the legends of regular figures.
- For the figures that you do NOT wish to display as Expanded View figures, they should be bundled together with their legends in a single PDF file called *Appendix*, which should start with a short Table of Content. Appendix figures should be referred to in the main text as: "Appendix Figure S1, Appendix Figure S2" etc.
- Additional Tables/Datasets should be labeled and referred to as Table EV1, Dataset EV1, etc. Legends have to be provided in a separate tab in case of .xls files. Alternatively, the legend can be supplied as a separate text file (README) and zipped together with the Table/Dataset file.
See detailed instructions here:

10) The paper explained: EMBO Molecular Medicine articles are accompanied by a summary of the articles to emphasize the major findings in the paper and their medical implications for the non-specialist reader. Please provide a draft summary of your article highlighting
- the medical issue you are addressing,
- the results obtained and
- their clinical impact.
This may be edited to ensure that readers understand the significance and context of the research. Please refer to any of our published articles for an example.

11) For more information: There is space at the end of each article to list relevant web links for further consultation by our readers. Could you identify some relevant ones and provide such information as well? Some examples are patient associations, relevant databases, OMIM/proteins/genes links, author's websites, etc...

12) Author contributions: CRediT has replaced the traditional author contributions section because it offers a systematic machine readable author contributions format that allows for more effective research assessment. Please remove the Authors Contributions from the manuscript and use the free text boxes beneath each contributing author's name in our system to add specific details on the author's contribution. More information is available in our guide to authors.

13) Disclosure statement and competing interests: We updated our journal's competing interests policy in January 2022 and request authors to consider both actual and perceived competing interests. Please review the policy https://www.embopress.org/competing-interests and update your competing interests if necessary.

14) Every published paper now includes a 'Synopsis' to further enhance discoverability. Synopses are displayed on the journal webpage and are freely accessible to all readers. They include a short stand first (maximum of 300 characters, including space) as well as 2-5 one-sentences bullet points that summarizes the paper. Please write the bullet points to summarize the key NEW findings. They should be designed to be complementary to the abstract - i.e. not repeat the same text. We encourage inclusion of key acronyms and quantitative information (maximum of 30 words / bullet point). Please use the passive voice. Please attach these in a separate file or send them by email, we will incorporate them accordingly.

Please also suggest a striking image or visual abstract to illustrate your article as a PNG file 550 px wide x 300-600 px high.

15) As part of the EMBO Publications transparent editorial process initiative (see our Editorial at http://embomolmed.embopress.org/content/2/9/329), EMBO Molecular Medicine will publish online a Review Process File (RPF) to accompany accepted manuscripts.
In the event of acceptance, this file will be published in conjunction with your paper and will include the anonymous referee reports, your point-by-point response and all pertinent correspondence relating to the manuscript. Let us know whether you agree with the publication of the RPF and as here, if you want to remove or not any figures from it prior to publication.
Please note that the Authors checklist will be published at the end of the RPF.

EMBO Molecular Medicine has a "scooping protection" policy, whereby similar findings that are published by others during review or revision are not a criterion for rejection. Should you decide to submit a revised version, I do ask that you get in touch after three months if you have not completed it, to update us on the status.

I look forward to receiving your revised manuscript.

Yours sincerely,

Lise Roth

Lise Roth, PhD
Senior Editor
EMBO Molecular Medicine

Please note: When submitting your revision you will be prompted to enter your funding and payment information. This will allow Wiley to send you a quote for the article processing charge (APC) in case of acceptance. This quote takes into account any reduction or fee waivers that you may be eligible for. Authors do not need to pay any fees before their manuscript is accepted and transferred to the publisher.

EMBO Press participates in many Publish and Read agreements that allow authors to publish Open Access with reduced/no publication charges. Check your eligibility: https://authorservices.wiley.com/author-resources/Journal-Authors/open-access/affiliation-policies-payments/index.html

***** Reviewer's comments *****

Referee #1 (Remarks for Author):

Reviewer comments

The GEMM described in this study are novel and the results are interesting and important. However, several important points require further clarification in the manuscript.

Specific comments
1. There are conflicting definitions of "high-risk EC" used throughout the manuscript, as follows:

• Abstract: "High risk endometrial cancer has poor prognosis"
• Introduction: "poor-risk group comprises 10-20% of tumours, and includes cases of serous, clear-cell and undifferentiated histology, and de-differentiated carcinosarcomas; metaplastic tumours derived from epithelial progenitors."
• Results: "focusing on high-risk tumours (TCGA copy number high UCEC subgroup and carcinosarcomas)"
These variable definitions are at odds with one another, which will be confusing to both experts and non-experts alike. For example, high-grade endometrioid tumors are not included in the histologic definition used in the introduction even though they are included in the TCGA copy number high UCEC subgroup and thus as included in the definition used in the results. Conversely, clear-cell and undifferentiated histologies are included in the definition used in the introduction but these subtypes were not included in the TCGA-UCEC cohort as thus not included in the definition used in the results.
2. Page-6: The first paragraph of the results section should clarify that in the TCGA copy number high UCEC subgroup and in the TCGA carcinosarcomas, PTEN mutations and FBXW7 mutations rarely co-exist; in fact, in the TCGA copy number high UCEC subgroup they are mutually exclusive (q=0.036). This information can be retrieved using the cBioPortal.
3. The rationale for choosing the R172H as the conditional knockin mutant of murine Trp53 should be provided since the corresponding human mutation (TP53-R175H) is rare in the TCGA copy number high UCEC subgroup, (constituting only 7 of 146 (4.8%) TP53 mutations in this subgroup), and is also rare in human TCGA carcinosarcomas and in endometrioid ECs in TCGA. Three other TP53 residues are mutated more frequently in human EC (S241, R248, and R273) so the rationale for knocking in the murine mutation that corresponds to human TP53-T175H is not obvious.

Referee #2 (Remarks for Author):

In the manuscript the authors have used various genetically modified mouse models with uterine specific gene modifications to define the role of mutations in Fbxw7, Pten and Tp53. In addition to the histopatology analyses the authors used various methods to dissect out the mechanisms behind the phenotypes appeared. It is of special importance that the point mutations used mimic those described to take place in the corresponding human tumors. Also various in silico methods were elegantly used to fill the gaps of the experimental analyses. The data generated with appropriate number of replicates with various genetic modifications and is technically solid. The results, especially, showed that the that Fbxw7 point mutation does not cause endometrial cancer by its own, but accelerates carcinogenesis caused by Pten loss or Trp53 missense mutation. Of the possible

down-stream signaling systems the authors analyzed the role of LEF1/Wnt-signalling, with a solid hypothesis.

As such the manuscripts is a significant contribution with extensive data towards understanding the tumorigenic pathway leading to endometrial carcinogenesis.

Minor comments include:
The genotyping data looks convincing in Fig S2, the information in Fig 1c is not clear.

The RNA expression data provided is a useful information source for others in the field, and should be placed in a public repository.

The table with the RNA expression data should have a headline showing the groups compared. This ease the use of the data files.

The reason for the difference in the outcome between Trp-del and Trep-fl/fl could be better explained.

Referee #3 (Comments on Novelty/Model System for Author):

There was a lack of human sample analysis to correlate the data. More data is necessary for the FWBX7 conclusions.

Referee #3 (Remarks for Author):

The manuscript by Brown et al. seeks to understand the genetic composition of high-risk endometrial cancer. The approach is to examine genetically engineered mouse models to investigate alterations in Fbxw7, pten, and p53. Mutant p53 was a "strong" driver of endometrial carcinogenesis. Mutation in Fbxw7 was not a driver but enhanced tumor progression in pten or p53-altered animals. The study switches focus on Fbxw7 to validate LEF and TCFL12 as a target of Fbxw7. Overall the study was well-controlled and clearly articulated.

There are some suggestions that the authors might consider.

1. The work correlates genetic alterations with carcinoma formation in mice. It would be suggested that the author consider testing if the same components would cause transformation in human endometrial cells.
2. While GEMMs are valuable models, the authors should consider examining human patient samples. This is particularly important with data regarding murine p53, and human p53 have some overlapping genes but also have a significant number of gene targets that don't overlap.
3. The data in Figure 5 supports interactions between Fbxw7 and LEF1 and the lower levels of TCFL2 through exogenous expression. The author should consider the examination of endogenous interactions and demonstration that Fbxw7 regulates the pathway.
4. Figure 1f and g conclude that loss of p53 increased PTEN. The author should quantitate these data. The pten gene is a transcriptional target of p53, and it would be concluded that one allele of p53 is active.

Minor

1. The author should provide more information regarding the Error bars presented in the figures. It was unclear if this was a standard error or standard deviation.
2. It was not evident what the cut-off for gene expression was to be considered significant.
3. The author should be careful in concluding p53 levels and gene sets (page 9). Gene ratios don't consider that these gene sets overlap with other transcription factors. For example, p53 can upregulate p21, but the TGFb1 pathway and others may also. The authors should consider re-evaluating this section and the conclusions.
4. The author should make the manuscript clear that this is a GEMM study throughout the manuscript which may provide partial insight into human neoplastic development. These studies will be used for artificial intelligence, which may not support outcomes in human neoplastic development without supportive data.

****** Reviewer's comments ******

Referee #1 (Remarks for Author):

Reviewer comments

The GEMM described in this study are novel and the results are interesting and important. However, several important points require further clarification in the manuscript.

Specific comments
1. There are conflicting definitions of "high-risk EC" used throughout the manuscript, as follows:
• Abstract: "High risk endometrial cancer has poor prognosis"
• Introduction: "poor-risk group comprises 10-20% of tumours, and includes cases of serous, clear-cell and undifferentiated histology, and de-differentiated carcinosarcomas; metaplastic tumours derived from epithelial progenitors."
• Results: "focusing on high-risk tumours (TCGA copy number high UCEC subgroup and carcinosarcomas)"
These variable definitions are at odds with one another, which will be confusing to both experts and non-experts alike. For example, high-grade endometrioid tumors are not included in the histologic definition used in the introduction even though they are included in the TCGA copy number high UCEC subgroup and thus as included in the definition used in the results. Conversely, clear-cell and undifferentiated histologies are included in the definition used in the introduction but these subtypes were not included in the TCGA-UCEC cohort as thus not included in the definition used in the results.

We are grateful to the reviewer for his/her positive evaluation of our draft manuscript and for highlighting these inconsistencies. We have sought to clarify this in our revised version as follows:

Introduction, p4, lines 5-9. "While most ~~cases~~ are low grade endometrioid tumours of early stage with favourable prognosis, the 10-20% of cases classified as high-risk ~~those of non-endometrioid histotype~~ have considerably worse outcomes[2-4]. This ~~poor risk~~ group ~~comprises 10-20% of tumours, and~~ includes high grade (grade 3) endometrioid tumours cases of non-endometrioid histologies including serous, clear-cell and undifferentiated histology, and de-differentiated carcinosarcomas; metaplastic tumours derived from epithelial progenitors[5]."

Results, p6, lines 5-10: We first examined *FBXW7*, *TP53* and *PTEN* mutation frequency and type relative to other IntOgen EC drivers[11] in the TCGA uterine corpus endometrial cancer (UCEC)[6] and carcinosarcoma (UCS)[21] cohorts, focusing on the ~~high-risk tumours (TCGA~~ copy number high UCEC subgroup and carcinosarcomas – hereafter referred to as high risk cases – in view of their exaggerated morbidity and mortality[3] (note that this definition excludes clear cell and undifferentiated tumours, which have poor prognosis but were excluded from the TCGA analyses).

2. Page-6: The first paragraph of the results section should clarify that in the TCGA copy number high UCEC subgroup and in the TCGA carcinosarcomas, PTEN mutations and FBXW7 mutations rarely co-exist; in fact, in the TCGA copy number high UCEC subgroup they are mutually exclusive (q=0.036). This information can be retrieved using the cBioPortal.

This is an important point which we are happy to add. For consistency, we have tested mutual exclusivity of oncogenic driver mutations among the 219 high risk tumours (copy no high UCEC and UCS) analysed elsewhere in our manuscript and now provide this in the first section of the Results, p6, lines 21-23:

Oncogenic mutations in *FBXW7* in this cohort of high risk endometrial cancers tended to mutual exclusivity with those in *TP53* (OR=0.53, *P*=0.12) and particularly *PTEN* (OR=0.32, *P*=0.067).

3. The rationale for choosing the R172H as the conditional knockin mutant of murine Trp53 should be provided since the corresponding human mutation (TP53-R175H) is rare in the TCGA copy number high UCEC subgroup, (constituting only 7 of 146 (4.8%) TP53 mutations in this subgroup), and is also rare in human TCGA carcinosarcomas and in endometrioid ECs in TCGA. Three other TP53 residues are mutated more frequently in human EC (S241, R248, and R273) so the rationale for knocking in the murine mutation that corresponds to human TP53-T175H is not obvious.

This is another important point. The mouse R172H (human R175H) allele was used for pragmatic reasons as it was readily available at the time the experiment was initiated. While we plan to examine the consequences of the mouse R270H allele in a future experiment, this is beyond the scope of the current study. We have added this as a study limitation in the Discussion as follows p14, lines 7-9:

Similarly, the *Trp53* R172H allele used in this study was chosen for pragmatic reasons rather than its prevalence in high-risk endometrial cancer and it will be of interest to examine the effects of orthologues of more common mutations such as *TP53* R273H.

****

Referee #2 (Remarks for Author):

In the manuscript the authors have used various genetically modified mouse models with uterine specific gene modifications to define the role of mutations in Fbxw7, Pten and Tp53. In addition to the histopatology analyses the authors used various methods to dissect out the mechanisms behind the phenotypes appeared. It is of special importance that the point mutations used mimic those described to take place in the corresponding human tumors. Also various in silico methods were elegantly used to fill the gaps of the experimental analyses. The data generated with appropriate number of replicates with various genetic modifications and is technically solid. The results, especially, showed that the that Fbxw7 point mutation does not cause endometrial cancer by its own, but accelerates carcinogenesis caused by Pten loss or Trp53 missense mutation. Of the possible down-stream signaling systems the authors analyzed the role of LEF1/Wnt-signalling, with a solid hypothesis.

As such the manuscripts is a significant contribution with extensive data towards understanding the tumorigenic pathway leading to endometrial carcinogenesis.

We are grateful to the reviewer for the positive comments on our study.

Minor comments include:
The genotyping data looks convincing in Fig S2, the information in Fig 1c is not clear.

We agree. As Figure S2 contains all the information included in Figure 1C, and these data are not essential to show in the main manuscript, we have removed panel C from the revised Figure 1 and now present Figure S2 alone.

The RNA expression data provided is a useful information source for others in the field, and should be placed in a public repository.

Microarray data have been uploaded to GEO under accession number GSE232356 and will be made freely available at the time of publication. Reviewer access is granted as follows:

To review GEO accession GSE232356:
Go to https://www.ncbi.nlm.nih.gov/geo/query/acc.cgi?acc=GSE232356
Enter token udajoammjvodved into the box

The table with the RNA expression data should have a headline showing the groups compared. This ease the use of the data files.

This is a good suggestion and we have added headers providing this information to each worksheet.

The reason for the difference in the outcome between Trp-del and Trep-fl/fl could be better explained.

On re-reading we agree that this section could be clearer. In fact, despite the greater frequency of uterine tumours in *Trp53*^{mut} mice compared to *Trp53*^{del} mice, survival of these two groups was not significantly different (*P*=0.30, log rank test). To clarify both this fact, and the difference in tumour phenotype between these genotypes, we have added statistical comparison of their survival to Figure 2a and revised the manuscript text as follows in the Results, p7 lines 27-34 & p8 lines 1-4:

"In contrast, *Trp53*^{del} mice showed no evidence of endometrial neoplasia at early timepoints, but instead developed external tumours requiring sacrifice from 24 weeks age, most of which were found to be soft tissue sarcomas on pathologist review (**Figure 2c-e)**. One *Trp53*^{del} female culled for an external tumour at 57 weeks age was found to also have an endometrial carcinoma on pathological review, and another two *Trp53*^{del} mice culled for external tumours at 51 and 68 weeks age had uterine sarcomas (**Figure 2a)**. While some *Trp53*^{mut} females also required sacrifice for external tumours, an appreciable fraction were culled for abdominal distension or genital bleeding, found to be secondary to uterine tumours on necropsy. Although survival of *Trp53*^{mut} mice was not significantly different to that of *Trp53*^{del} animals, and pathology review revealed ~~showed~~ similar frequency of extrauterine tumours (predominantly sarcomas) to *Trp53*^{del} mice (**Figure 2a,d,e)**, the frequency of endometrial carcinoma in *Trp53*^{mut} mice was far greater, with 8 of 11 (72.7%) ~~mice~~ developing epithelial malignancies during the study (*P*=3e-04 vs *Trp53*^{del}, Fisher exact test) (**Figure 2a-c**)."

****

Referee #3 (Comments on Novelty/Model System for Author):

There was a lack of human sample analysis to correlate the data. More data is necessary for the FWBX7 conclusions.

We concur that human data defining the role of *FBXW7* missense mutation in tumour initiation and promotion are highly valuable.

With respect to causative human data, we would like to highlight that in our study we validated the enrichment of the LEF1 signature identified in our GEMM tumours using gene expression data from human isogenic HCT116 cells into which the FBXW7 R505C missense hotspot mutation had been introduced. Indeed, this analysis revealed that the LEF1 signature was the most enriched in FBXW7 missense-mutant cells. We also performed the co-immunoprecipitation experiments using human HEK293T cells in which we confirmed reduced interaction of LEF1 and TCF7L2 with FBXW7 mutated and hotspot residues within the WD40 repeats. At the suggestion of this reviewer, the highly experienced staff at our transgenic core facility have spent several months attempting to engineer immortalized human endometrial cells to carry the *FBXW7* hotspot mutation equivalent to that tested in our GEMM, however this has proven technically unfeasible as detailed further below and in the attached Annexe.

In addition to the above, and to further confirm the relevance of our study to human disease, we have also added two important correlative analyses to the revised main and supplementary text:

1. Gene set enrichment analysis of high-risk human endometrial cancers from the TCGA UCEC and UCS cohorts, which demonstrates significant enrichment of the LEF1 gene signature in tumours carrying FBXW7 missense mutations.

2. Immunohistochemical staining for LEF1 in high grade human endometrial cancers of p53 mutant and no specific molecular profile (NSMP) subtypes with and without FBXW7 missense mutation. This analysis demonstrates that strong nuclear LEF1 staining is common in tumours with both mutant and wild-type *FBXW7,* consistent with an oncogenic role and alternative mechanisms of activation in *FBXW7* wild-type samples.

Further details of both these analyses are provided below. We believe that these additional data substantially strengthen our conclusions, and we hope address the reviewer's concerns regarding human relevance.

Referee #3 (Remarks for Author):

The manuscript by Brown et al. seeks to understand the genetic composition of high-risk endometrial cancer. The approach is to examine genetically engineered mouse models to investigate alterations in Fbxw7, pten, and p53. Mutant p53 was a "strong" driver of endometrial carcinogenesis. Mutation in Fbxw7 was not a driver but enhanced tumor progression in pten or p53-altered animals. The study switches focus on Fbxw7 to validate LEF and TCFL12 as a target of Fbxw7. Overall the study was well-controlled and clearly articulated.

We are grateful to the reviewer for these positive comments on our study.

There are some suggestions that the authors might consider.

1. The work correlates genetic alterations with carcinoma formation in mice. It would be suggested that the author consider testing if the same components would cause transformation in human endometrial cells.

This is an interesting suggestion. We have experience with human hEM3 cells, which were derived from normal endometrium (PMID: 34376780) and kindly shared with our laboratory by their creator. Like other immortalized human endometrial cell lines 12-Z, 49-Z, 108-Z and 11-Z , these were immortalized by SV40-TAg, meaning that they lack functional p53 and are arguably an imperfect model for analysis of alterations impacting on, and impacted by the p53 pathway as those we test in our study are. This caveat nothwithstanding, it is certainly of interest to define the effects of *FBXW7* missense mutation (± *PTEN* loss) in normal human endometrial cells and we have spent much time and effort trying do this in hEM3 cells over the last few months. This work has been performed by senior staff in our transgenic core facility, who have extensive experience in successful CRISPR-Cas9 gene editing of human and mouse cells. Full details of this experiment are provided in the attached Annexe below and can be summarised as follows:

Our core facility designed a guide RNA to target the *FBXW7* genomic sequence close to codon 479, and a single-stranded oligodeoxynucleotide (ssODN) to introduce the *FBXW7* R479Q mutation equivalent to that used in our GEMM. The ssODN contained other silent mutations to enable screening of cells for successful targeting by restriction digest. Guide RNA and ssODN were introduced into early passage hEM3 cells by electroporation. After 48 hours, the cells were split into three 96 well plates and expanded for 25 days before each well in each plate was further split into two across two 96 well plates – one for genotyping and one for maintenance. Restriction digest of cell pools from each well of each genotyping plate demonstrated recombination products of successful ssODN integration in 8 of 288 wells ie 2.8% targeting efficiency – the expected frequency for HDR targeting. Cells from these 8 wells were pooled and FACS sorted for monoclonal expansion. Unfortunately, these single cells failed to proliferate and generate clones, even after 4 weeks of incubation. Given that the core facility has successfully performed HDR gene editing of

multiple cell types and multiple targets, we hypothesise that the failure of the targeted immortalized endometrial cells to grow is a consequence of their modest proliferation rate and robustness (vs cancer cells or fetal cells often used for gene targeting) and the functional consequences of FBXW7 mutation. Regrettably we have therefore been unable to test the postulate suggested above. Reference to this experiment has been added to the Results, p11, lines 23-26:

"Similarly, our attempts to define the effects of *FBXW7* mutation and PTEN loss in normal human endometrial cells proved unsuccessful, as hEM3 cells targeted with *FBXW7* mutation failed to expand from single cell colonies."

And its absence added to the Discussion section as a limitation of the current study p14, lines 12-14:

"Finally, our attempts to introduce *FBXW7* missense mutation into immortalised human endometrial cells were unsuccessful, and it will be of interest to develop methods to permit this in cells or organoids in the future."

2. While GEMMs are valuable models, the authors should consider examining human patient samples.

This is also a good point, and we agree that inclusion of human data would strengthen our study. We have added two independent analyses to provide this:

1. Gene set enrichment analysis of high-risk human endometrial cancers from the TCGA UCEC and UCS cohorts. This demonstrates that the LEF1 signature is one of the most enriched gene signatures in *FBXW7* missense-mutant tumours, consistent with a role of LEF1 signalling in the pathogenesis of human endometrial cancer driven by *FBXW7* mutation. These results are shown in the revised Figure 3b alongside the corresponding data from the GEMM endometrial tumours and the human isogenic FBXW7 mutant cells, with corresponding data provided in the revised Table S7. They are also presented in the revised Results as follows p9, lines 23-26:

We sought to confirm the association of *FBXW7* mutation with LEF1 signalling in human samples. GSEA of high-risk endometrial cancers from TCGA (CN high UCEC and UCS) revealed enrichment of LEF1 signalling in cases with *FBXW7* missense driver mutation (**Figure 3b, Table S7**).

2. Semiquantitative immunohistochemical of high grade human endometrial cancers of p53 mutant and no specific molecular profile (NSMP) subtypes with and without *FBXW7* missense mutation. This analysis demonstrates that strong nuclear LEF1 staining is common in tumours with both mutant and wild-type *FBXW7*. This is consistent with its nuclear localisation in endometrial cancers from *FBXW7* wild-type GEMM as shown in the revised Figure S12, and previous data from human and mouse tumours (eg this recent preprint which we cite). Representative images of LEF1 IHC together with its quantification have been added as Figure S11 and are reported in the Results as follows p11, lines 13-18:

"Interestingly, analysis of high-grade human endometrial cancers of no specific molecular profile (NSMP) and p53 mutant subtypes and murine endometrial cancers from $Pten^{del}$ and $Trp53^{mut}$ GEMM ~~mice~~ at experimental endpoint revealed near-universal epithelial LEF1/Lef1 expression, irrespective of *FBXW7*/*Fbxw7* mutation (**Figure S11, S12**), suggesting that *FBXW7*/*Fbxw7* wild-type tumours may employ alternative mechanisms of Lef1 upregulation, as has been suggested by earlier studies of mouse and human tumours[27,35]."

While these data are consistent with an oncogenic role for LEF1 in endometrial cancer, the inability to quantitate LEF1 protein by IHC precludes comparison of LEF1 pathway activity by *FBXW7* status, which is better captured by the GSEA above. Furthermore, as human endometrial cancers carry alterations in

multiple genes other than *FBXW7* which impact on the Wnt pathway and thus LEF1 signalling (selected examples include *CTNNB1*, *SOX17* and *APC*) there are many potential explanations for nuclear LEF1 localisation in *FBXW7* wild-type cancers. Defining this is beyond the scope of this study, but would certainly be of interest in the future.

This is particularly important with data regarding murine p53, and human p53 have some overlapping genes but also have a significant number of gene targets that don't overlap.

Regarding the specific comment on differences in the gene targets of murine and human p53, we note that the GSEA we show was derived from *Fbxw7*^{R482Q}, and *Pten*^{del}, *Fbxw7*^{R482Q} mice rather than *Tp53*^{del} or *Tp53*^{del}, *Fbxw7*^{R482Q} mice. However, the point regarding the difference in p53 targets is well made and well taken, as indeed p53 gene sets were not among the most enriched with *FBXW7* mutation in either the human endometrial cancers or the human isogenic HCT116 cells, in contrast to the mouse tumours. Whether this relates to differences in p53 regulated genes between mouse and human, or to the frequent *TP53* mutations in high-risk human endometrial cancers and other alterations impacting on the p53 pathway in highly mutated HCT116 cells is impossible to know. We have highlighted and speculated on this discordance together with an additional comment on MYC pathway enrichment in the Results as follows p9, line 31 – p10, line 1:

"Interestingly, while as in the GEMM tumours *FBXW7* mutation was associated with enrichment of MYC signalling in the human endometrial cancers, this was not the case in the colon cancer cells, and dysregulation of p53 gene sets was less evident in both, possibly owing to different p53 targets in mouse and human, or mutations in *TP53* and other genes in its pathway in the human samples (**Figure 3a-c**)."

3. The data in Figure 5 supports interactions between Fbxw7 and LEF1 and the lower levels of TCFL2 through exogenous expression. The author should consider the examination of endogenous interactions and demonstration that Fbxw7 regulates the pathway.

We agree that while exogenous expression of tagged proteins remains arguably the most popular method to demonstrate protein-protein interaction, it runs risk of false positives. Indeed, we sought to minimise the risk of these by pulldown of tagged FBXW7 and reciprocal pulldown of LEF1 and TCF7L2. Unfortunately, the lack of reliable antibodies against FBXW7 precluded co-immunoprecipitation of endogenous protein, and technical limitations prevented examination of FBXW7 interactions by FRET-FLIM, despite great effort and expert technical guidance by our institute core imaging facility. We are happy to mention this as a limitation of our study if requested.

Regarding the final comment on demonstration that FBXW7 regulates the (LEF1) pathway, we would like to highlight that evidence in support of this can be drawn from the GSEA on the human isogenic HCT116 cells, where LEF1 signalling is the most enriched gene set in those carrying FBXW7 missense WD40 mutation.

4. Figure 1f and g conclude that loss of p53 increased PTEN. The author should quantitate these data. The pten gene is a transcriptional target of p53, and it would be concluded that one allele of p53 is active.

We have performed densitometric quantification of protein levels in the immunoblots shown in the previous Figure 1f (Figure 1e in the revised manuscript) as requested, and have added the values (and SDs) to the figure together with the results of statistical comparison between groups. Indeed, as the reviewer suspects this confirms our previous suggestion of increased Pten protein levels in mice with uterine *Trp53* deletion (1.3 fold increase ± 0.10; P=0.029 Mann-Whitney test).

The observation above that *Pten* is a transcriptomic target of p53 is interesting, as complementary analysis of uteri by qPCR at the same 8 week timepoint demonstrates no significant increase in *Pten* mRNA in either *Trp53*^{del} or *Trp53*^{del}, *Fbxw7*^{mut} samples (1.09 and (1.1 fold increase respectively, P=0.15 and P=0.22, Mann-Whitney test, n=5 samples each group). Given this result, and the fact that PTEN is regulated at the

transcriptional level by multiple factors other than p53, including c-Jun, NOTCH, PPARγ, ATF2 and MYC (PMID: 24054978) it is not clear to us at least that these data alone indicate monoallelic recombination of Trp53 – particularly as this is inconsistent with the substantial decrease in Trp53 mRNA in *Trp53*^{del} uteri compared to wild-type uteri at 4 weeks (14% of wild-type expression) and 8 weeks (6% of wild-type expression) as shown in Figure 1e (revised Figure 1d).

Indeed, it seems plausible that this upregulation could be post-transcriptional, potentially due to one of the many factors known to mediate this (PMID: 21045203). While certainly of interest, further exploration of the mechanism(s) underpinning this is beyond the scope of the current study.

Minor

1. The author should provide more information regarding the Error bars presented in the figures. It was unclear if this was a standard error or standard deviation.

The whiskers in the plots in Fig 1e, 3c and 3d are actually the minimum and maximum values (boxes show mean with IQR). We have clarified this in the figure legends in the revised manuscript, but we are also happy to revise the figure to show SD or SEM if requested.

2. It was not evident what the cut-off for gene expression was to be considered significant.

We used a two sided alpha of <0.05 or an FDR or <0.05 depending on the analysis as noted in Methods, Statistical analysis – p20, lines 6-7.

3. The author should be careful in concluding p53 levels and gene sets (page 9). Gene ratios don't consider that these gene sets overlap with other transcription factors. For example, p53 can upregulate p21, but the TGFb1 pathway and others may also. The authors should consider re-evaluating this section and the conclusions.

This is a good point. The enrichment for the p53 gene set in the *Fbxw7*-mutant GEMM uteri is mentioned as a point of interest, rather than a finding central to conclusions of our study. Nevertheless, we have revised the sentence in the Results, p9, lines 12-16 to:

"These included those corresponding to epithelial-mesenchymal transition (EMT) (the most enriched hallmark gene set), dys~~up~~regulation of p53 signalling (consistent with the increased p53 protein on immunostaining, although we note that enrichment of p53 gene sets is not specific for alterations in p53 itself), signalling by known FBXW7 substrate Myc, and multiple other oncogenic cellular signalling pathways (**Figure 3a**)."

4. The author should make the manuscript clear that this is a GEMM study throughout the manuscript which may provide partial insight into human neoplastic development. These studies will be used for artificial intelligence, which may not support outcomes in human neoplastic development without supportive data.

We are happy to clarify this if it was not clear in the previous version.

In addition to the current abstract, which explicitly notes that we used GEMM for functional analysis (sentence 3), we have now added explicit mention of this in the synopsis (both bullet points and blurb) so this will be immediately obvious to any reader.

Given that at the helpful suggestion of this reviewer we have now added human correlative data, we are would prefer not to refer to this study as an exclusively GEMM study.

We hope this addresses this concern but would be happy to make additional alterations if required. .

Reviewer #3 Additional comment communicated by email 16 Dec 2022

"I would strongly suggest that the authors repeat key data [on] one of the endometrial immortalized cell lines 12-Z, 49-Z, 108-Z and 11-Z. IF they choose not to perform experiments in the human cell lines, I am not sure they are addressing human disease in this study."

We sincerely hope this comment has been addressed in our response to Reviewer #3 Major comment #1 above.

ANNEXE

REPORT FROM WHG TRANSGENIC CORE FACILITY ON GENE TARGETING IN IMMORTALIZED HUMAN NORMAL ENDOMETRIAL CELLS

PHALGUNI RATH

03 MAY 2023

Summary

To generate the R479Q (G->A) SNP, we designed a single guide RNA (FBXW7-CRISPOR A) targeting the genomic sequence very close to the nucleotides encoding the Arginine residue. An ssODN harbouring the desired G->A SNP for homology directed repair was designed. Silent mutations were introduced to inhibit retargeting after successful HDR. The nucleotide conversion after successful integration, creats a BamHI restriction site in close proximity of the sgRNA target region. PCR primers were designed to amplify 717 bases surrounding the SNP site and BamHI restriction digestion of the PCR products was used for genotyping.

The site-specific Cas9 nuclease as a ribonucleoprotein complex with FBXW7 Cr-A sgRNA and the ssODN were electroporated into early passage cells. For initial characterization, 20 cells were seeded in each well of a 96 well plate and BamHI RE digestion based genotyping of individual wells lead to the identification of putative HDR pools. The positive 20 cell pools were FACS sorted to seed one single cell in each well of a 96 well plate. The cells didn’t survive monoclonal expansion.

Strategy for mutagenesis

A CRISPR/Cas9 nuclease is comprised of two components – a protein component, Cas9 which is the nuclease and an RNA molecule which represents a hybrid of two RNA species, the tracrRNA and the crRNA. The first 20 nucleotides of the crRNA sequence defines the specificity of the nuclease which is achieved by complementary base pairing with the target sequence in genomic DNA. The 20 nucleotide genomic target site is known as the Protospacer and requires the presence of a 3' NGG motif, known as the Protospacer Adjacent Motif (PAM) for cleavage. Target sites for CRISPR/Cas9 mediated mutagenesis thus can be described as X_{20}XGG where X is potentially any nucleotide. The components of the CRISPR/Cas9 system are summarized in Figure 1.

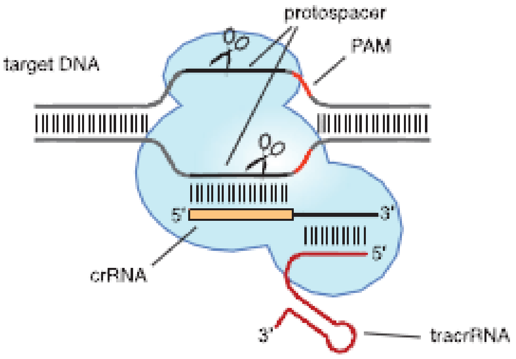

Figure 1. CRISPR/Cas9 system. The Cas9 protein is shown in blue which complexes with two RNA cofactors, a tracrRNA and a crRNA the first 20 nucleotides of which define the cleavage specificity of the nuclease.

For simplicity, frequently the tracrRNA and the crRNA are linked together by a small hairpin to create a single guide RNA, known as sgRNA which can be easily generated in the laboratory by in vitro transcription.

The site-specific nuclease can be used to introduce a double strand break (DSB) into the genome which is known to be highly recombinogenic. The DSB is either repaired by non-homologous end joining (NHEJ) which effectively stitches the broken ends back together or an alternative homology directed repair (HDR) pathway is used to repair the lesion using homologous sequences as a template (Figure 2). NHEJ can lead to the introduction of indel mutations at the site of the DSB which can be mutagenic and lead to disruption of a protein coding open-reading-frame (ORF). Where two CRISPR/Cas9 nucleases are used and they introduce two DSB *in cis*, the intervening sequence is frequently deleted, providing a robust strategy for a Knock-out.

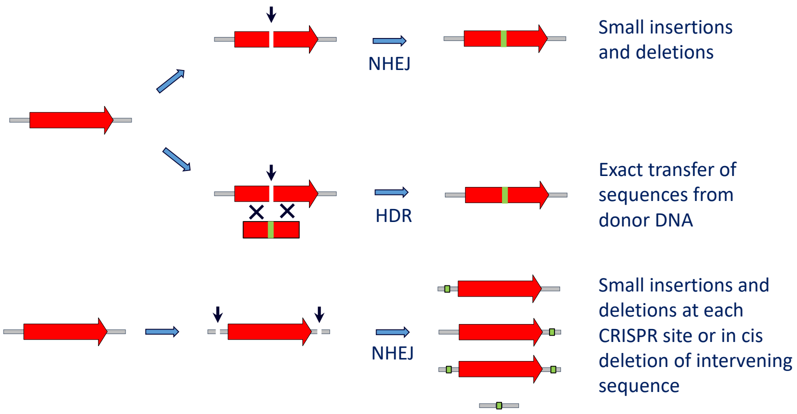

Figure 2. (overleaf) Nuclease mediated genome engineering. A DSB lesion introduced by a site specific nuclease can be processed by either NHEJ which can lead to the disruption of a target sequence by indels, or HDR where a homologous template is used to copy specific changes into the genome. When two Nucleases are used *in cis*, each

introduced double strand break, can be repaired with the insertion of an indel, or the deletion of the intervening sequence can result.

Selection of gRNA sequence for introduction of the G to A SNP in the FBXW7 gene

The aim of the experiment was to generate R479Q (G->A) SNP in the FBXW7 gene, and a strategy involving CRISPR/Cas9 site specific nucleases along with a repair ssODN was used. The repair of the induced double strand break could result in the insertion of the supplied ssODN sequence, harbouring the desired point mutation in the target gene. Figure 3 shows the genomic structure of the FBXW7 gene.

Putative target sequence for a CRISPR/Cas9 nuclease was designed using the CRISPOR sgRNA design tool (http://crispor.tefor.net). The guide RNA sequence was selected on the basis of being relatively genome unique with minimal off-target sites found across the genome (Table 1).

Table 1 – Selected guide and predicted off-targets

	sgRNA Sequence + PAM sequence	Off targets
FBXW7-CRISPOR A	GTTCTCGAGATGCCACTCTT AGG	0 - 0 - 1 - 7 - 75*<br>0 - 0 - 0 - 4 - 2

* For each number of mismatches, the number of off-targets is indicated. Example: 0 - 0 - 1 - 7 - 75 means 0 off-target with 0 mismatches, 0 off-targets with 1 mismatch, 1 off-targets with 2 mismatches, etc. Off-targets are considered if they are flanked by one of the motifs NGG, NAG or NGA.

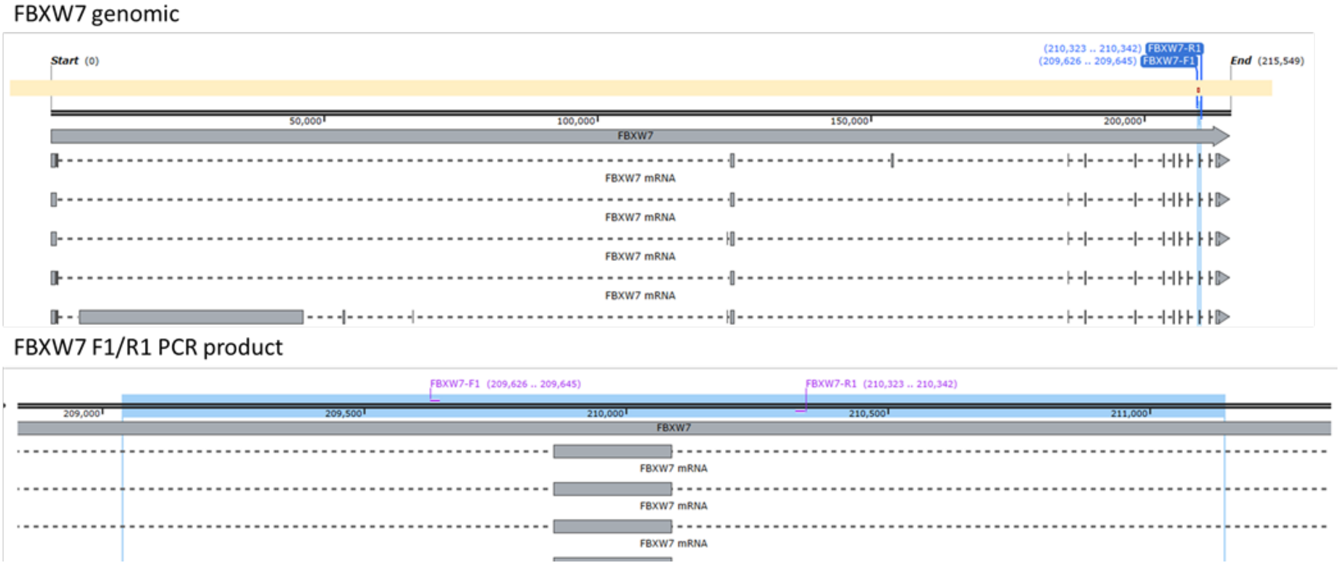

Figure 3. Genomic structure and position of selected CRISPR/Cas9 target site. The position of the target stie for mutagenesis is highlighted in blue. The position of PCR primers used for genotyping is highlighted in pink.

The ssODN was designed by changing the target nucleotide (G to A). Further silent mutations were included to deplete the PAM and introduce a BamHI RE site (Figure 4). G**enotyping for successful HDR was performed using BamHI estriction digestion of the PCR products.**

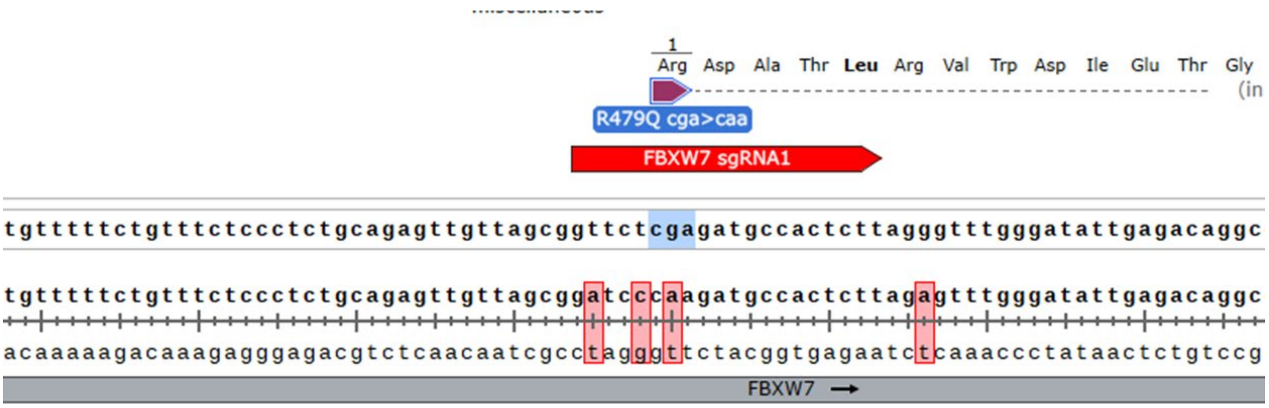

Figure 4. Segment of the ssODN used for homology directed repair depicting the G to A modification.

Design and testing of CRISPR/Cas9 reagents

The selected guide RNA was purchased from Synthego as synthetic RNA molecules. PCR primers were designed to amplify genomic regions surrounding the CRISPR target sites (Tables 2 and 3).

Table 2. PCR primers used for genotyping

FBXW7-F1	5'- CAGGTCAGGGAACCAAGCAT -3'
FBXW7-R1	5'- GAGCCCCAAAGTGTCAGGTT -3'

Table 3. PCR conditions

Immolase PCR (Bioline- 21047)	
Buffer (10X)	2 µl
Q solution	4 µl
$MgCl_2$ (50mM)	0.6 µl
dNTP mix (8mM)	0.5 µl
Forward Primer (10 µM)	0.5 µl
Reverse Primer (10 µM)	0.5 µl
Immolase Taq (5U/µl)	0.3 µl
DNA	1 µl (100ng)
Water	10.6 µl
Total	20 µl

PCR Conditions	Temperature	Time	
Initial Denaturation	98^0C	10 minutes	
Denaturation	95^0C	15s	35 cycles
Annealing	58^0C	20s	
Extension	72^0C	10s	
Final extension	72^0C	2 minutes	

Mutagenesis
The synthetic guide RNAs (Synthego) were complexed with protein Cas9 (HiFi Cas9 V3, IDT) to prepare the RNP mix (table 4).

Table 4. Preparation of the RNP complex

Buffer R	4.4 µl
sgRNA (FBXW7-CrA)	1 µl
Cas9	0.6 µl
Total	6 µl

The sgRNA and Cas9 mix was incubated at room temperature for 15 minutes. 0.5 µl ssODN (100uM) was added to RNP mix. The cells were dissociated using Trypsin and resuspended in 5ml PBS. Cells were counted using the automated cell counter. 10^5 cells were electroporated using the Neon electroporation system at **1200 volts, 30ms and 2 pulses**. The cells were seeded on one well of a 48 well plate for recovery. After 48 hours of, the cells were dissociated and approximately 20 single cells were seeded in each well of a 96 well plate. A small pool of cells were collected for genomic DNA preparation and the rest of the cells were frozen as a back up.

The mutagenesis was assayed by BamHI digestion of the FBXW7 F1/R1 PCR products from the pool of cells (Figure-5).

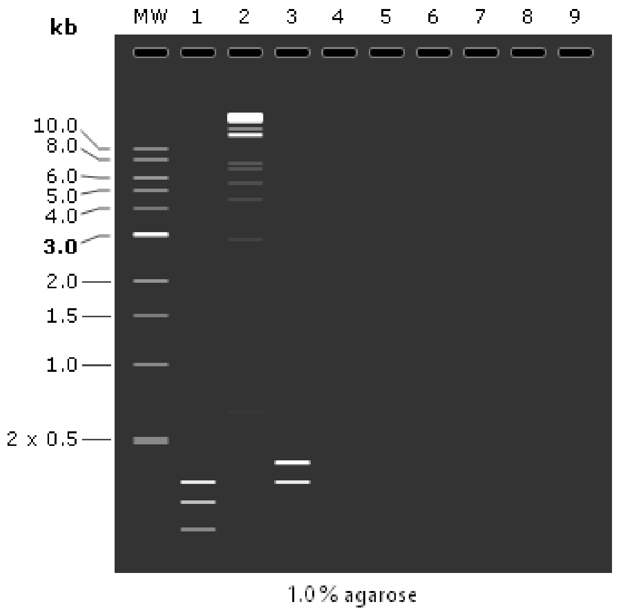

Figure 5. Analysis of the electroporated pool for possible HDR. Lane 1- BamHI digestion of the edited PCR product. The digested bands are 321bp, 247bp and 149bp . Lane 3- BamHI digestion of the wild type PCR product. The digested bands are 396bp and 321bp.

Genotyping 20 cell pools in 96 well plate

Approximately 20 cells were seeded in each well of a 96 well plate and grown for 25 days before splitting on to 2X96 well plate for genotyping and maintenance. The plate for genotyping was lysed for genomic DNA and PCR to amplify the target region was performed following the protocol mentioned in Table-3. 5ul of the PCR products were digested using BmrI (Table 5). The digested products were run on a 2% agarose gel.

Table 5. BmrI RE digestion

Cut Smart Buffer	3 µl
BmrI	0.3 µl
PCR product	5 µl
NFW	21.7 µl
Total	30 µl

Seven pools (C2, C6, C10, C27, C31, C45 and C53) showed expected digestion products, indicative of successful integration of the ssODN (Figure 6). Cells from the positive pools were pooled together and seeded in ultra low density for monoclonal expansion.

An example of the expected digestion pattern which was observed in few pools from the 96 well plate. These are 20 cell pools, therefore mosaic digestion was expected.

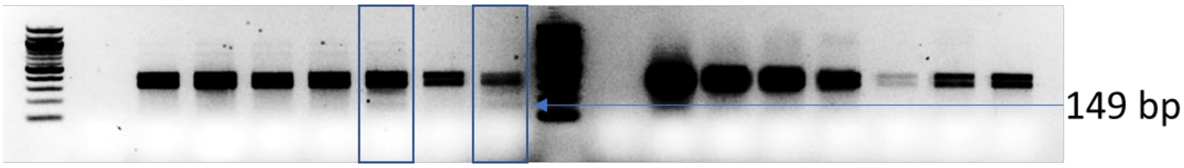

Figure 6. Primary screen. BamHI digestion of FBXW7-F1/R1 PCR products from individual wells. Eight pools from 3X 96 well plates showed digestion.

Sanger sequencing of the PCR product confirmed traces of homology directed repair.

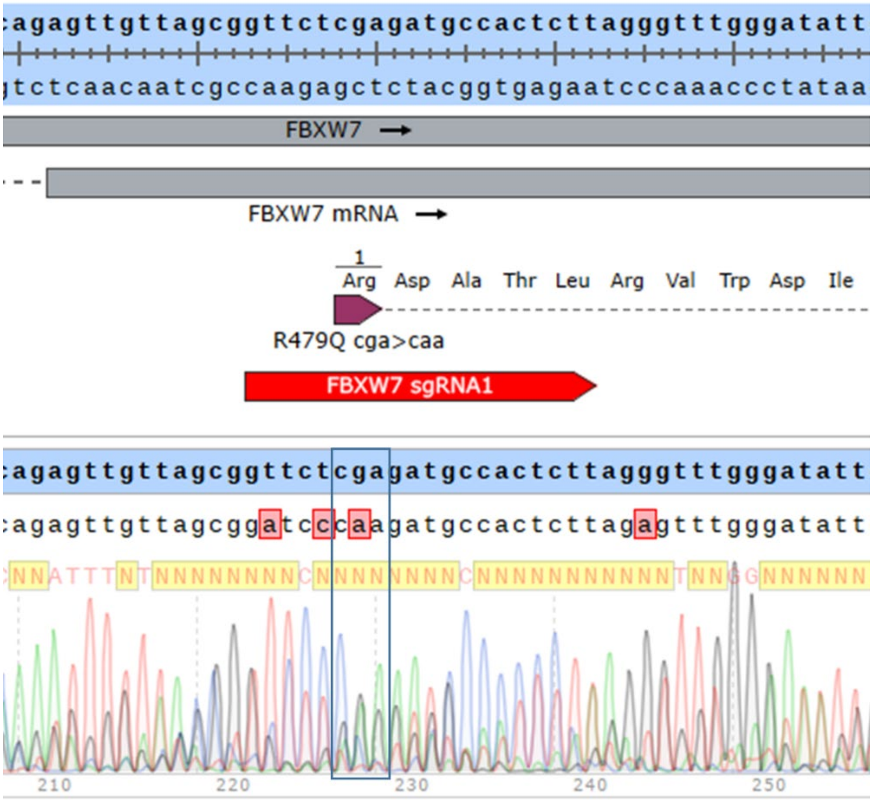

Other genotyping gels

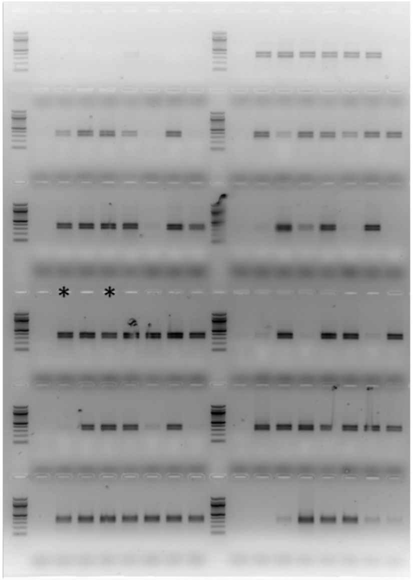

28.02.23

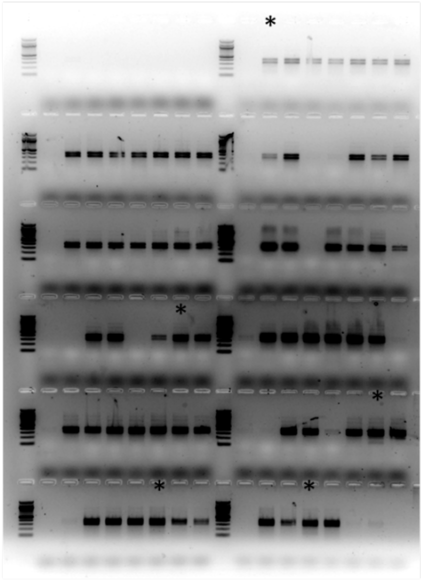

01.03.23

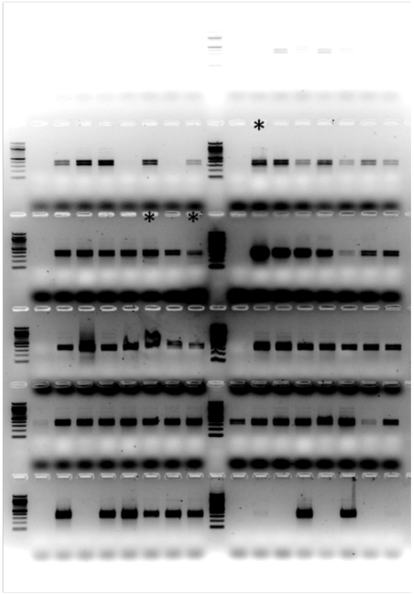

01.03.23

Cells from the eight positive wells wells were pooled together and FACS sorted for monoclonal expansion. Unfortunately, the single cells failed proliferate and to generate clones.

9th Jun 2023

Dear Dr. Church,

Thank you for the submission of your revised manuscript to EMBO Molecular Medicine. We have now received the reports from referees #1 and #3 who re-reviewed your manuscript. As you will see, both referees are supportive of publication, and we will therefore be able to accept your manuscript once the following points will be addressed:

1/ Referees' comments: please address the remaining minor concerns from referee #1.

2/ Main manuscript text:
- Please address the queries from our data editors in track changes mode (see the related file "data edited manuscript"). Accept all previous changes, and only keep in track changes mode any new modification.
- Please provide up to 5 keywords.
- Kindly remove "Research support" from page 2 and provide this information in the "Acknowledgements" section. The information provided in the manuscript needs to be identical to the one provided in the submission system (currently missing from the system: the Oxford NIHR Comprehensive Biomedical Research Centre (BRC), the Munich Clinician Scientist Programme (Verein zur Förderung von Wissenschaft und Forschung an der Medizinischen Fakultät der LMU München e.V.), MR/P000738/1, 203141/Z/16/Z).
- Materials and methods:
o Human cancers: please add a statement that the experiments conformed to the principles set out in the WMA Declaration of Helsinki and the Department of Health and Human Services Belmont Report.
o Statistics: please include a statement about randomization and inclusion/exclusion criteria (and correct the checklist accordingly).
- Data Availability Section: Thank you for providing a link with reviewer token. Please note that the data must be public before acceptance of the manuscript. Please also remove the sentence "other data are available from the ... conflict of interest".
- Author contributions: CRediT has replaced the traditional author contributions section because it offers a systematic machine-readable author contributions format that allows for more effective research assessment. Please remove the Authors Contributions from the manuscript and use the free text boxes beneath each contributing author's name in our system to add specific details on the author's contribution. More information is available in our guide to authors.
- Please rename conflict of interest: "Disclosure statement and competing interests". (We updated our journal's competing interests policy in January 2022 and request authors to consider both actual and perceived competing interests. Please review the policy https://www.embopress.org/competing-interests and update your competing interests if necessary.)
- References: please list the references in alphabetical order, with 10 authors before et al, and remove the DOIs.

3/ Figures and Appendix:
- Please provide exact p values, not a range, in all figures or in their legends, (currently missing in figure 1d).
- Figure 1e: in the legend, you indicated "image is representative of duplicate technical replicates". Do I understand correctly that the Western Blots have been performed twice from the same samples? How were the SD obtained? Please note that the use of statistical tests needs to be justified, and that with two values it is usually preferred to present both values.
- Appendix: please correct the nomenclature to "Appendix Figure S1", "Appendix Figure S2", etc. and correct the callouts accordingly in the manuscript. Please note that you have the possibility to make some of the Appendix figures "Expanded View Figures" that are collapsible/expandable online. A maximum of 5 EV Figures can be typeset. EV Figures should be cited as 'Figure EV1, Figure EV2" etc... in the text and their respective legends should be included in the main text after the legends of regular figures.
- The Suppl. Tables S1-S11 in Excel file should be renamed to Dataset EV1-EV11 and uploaded as Dataset; the references to these tables in the manuscript should be updated accordingly.
- References are missing in the manuscript for Table S11, Figure 6d, and for individual panels in the appendix figures.

4/ At EMBO Press we ask authors to provide source data for the main figures. From previous email exchanges, I understand you did not receive the email from our source data coordinator Hannah, but please see the checklist attached (Source Data Checklist) listing the figure panels for which source data are needed. Please contact us or Hannah (h.sonntag@sourcedata.embo.org) if you have any question.

5/ Synopsis:
I slightly edited your text to fit our format and style, please let me know if you agree with the following or amend as you see fit:
The consequences of common endometrial driver mutations were studied in mice, and found to exert different functional effects alone and in combination.
• FBXW7 mutation requires additional alterations to exert oncogenic effect in endometria
• Wnt pathway effectors LEF1 and TCF7L2 are novel FBXW7 substrates and potential therapeutic targets in FBXW7-mutant endometrial cancer
Thank you for providing a synopsis picture, please resize it to a png/jpeg/tiff file 550 px wide x 300-600 px high.

6/ As part of the EMBO Publications transparent editorial process initiative (see our Editorial at http://embomolmed.embopress.org/content/2/9/329), EMBO Molecular Medicine will publish online a Review Process File (RPF) to accompany accepted manuscripts.
This file will be published in conjunction with your paper and will include the anonymous referee reports, your point-by-point response and all pertinent correspondence relating to the manuscript. Let us know whether you agree with the publication of the RPF and as here, if you want to remove or not any figures from it prior to publication.
Please note that the Authors checklist will be published at the end of the RPF.

I look forward to receiving your revised manuscript.

Yours sincerely,

Lise Roth

Lise Roth, PhD
Senior Editor
EMBO Molecular Medicine

The system will prompt you to fill in your funding and payment information. This will allow Wiley to send you a quote for the article processing charge (APC) in case of acceptance. This quote takes into account any reduction or fee waivers that you may be eligible for. Authors do not need to pay any fees before their manuscript is accepted and transferred to our publisher.

***** Reviewer's comments *****

Referee #1 (Remarks for Author):

Specific comment-1:
The authors' response reads: "While most are low grade endometrioid tumours of early stage with favourable prognosis, the 10-20% of cases classified as high-risk have considerably worse outcomes2-4. This group includes high grade (grade 3) endometrioid tumours, cases of non-endometrioid histologies including serous, clear-cell and undifferentiated histology, and de-differentiated carcinosarcomas; metaplastic tumours derived from epithelial progenitors5."
Reviewer comment on the revised text: Please further clarify whether the "10-20% of cases classified as high-risk" refers to 10-20% of all EC cases, or to 10-20% of endometrioid cases.

Specific comment-2:
Original comment: "The first paragraph of the results section should clarify that in the TCGA copy number high UCEC subgroup and in the TCGA carcinosarcomas, PTEN mutations and FBXW7 mutations rarely co-exist; in fact, in the TCGA copy number high UCEC subgroup they are mutually exclusive (q=0.036). This information can be retrieved using the cBioPortal"
The authors' response: "This is an important point which we are happy to add. For consistency, we have tested mutual exclusivity of oncogenic driver mutations among the 219 high risk tumours (copy no high UCEC and UCS) analysed elsewhere in our manuscript and now provide this in the first section of the Results, p6, lines 21-23: Oncogenic mutations in FBXW7 in this cohort of high risk endometrial cancers tended to mutual exclusivity with those in TP53 (OR=0.53, P=0.12) and particularly PTEN (OR=0.32, P=0.067). "
Reviewer comment on the revised text: Because the UCEC and UCS tumors are moelcualrly distinct, the data on mutual exclusivity should calculated and presented separately for each cohort. In addition to the p-values, the q-values must be provided to account to multiple testing of > 21,000 protein genes sequenced. Odds ratios, p-values and q-values can easily be retrieved from the cBIOportal for each of these cohorts.

Specific comment-3:
Original comment: The rationale for choosing the R172H as the conditional knockin mutant of murine Trp53 should be provided since the corresponding human mutation (TP53-R175H) is rare in the TCGA copy number high UCEC subgroup, (constituting only 7 of 146 (4.8%) TP53 mutations in this subgroup), and is also rare in human TCGA carcinosarcomas and in endometrioid ECs in TCGA. Three other TP53 residues are mutated more frequently in human EC (S241, R248, and R273) so the rationale

for knocking in the murine mutation that corresponds to human TP53-T175H is not obvious.
The authors' response: This is another important point. The mouse R172H (human R175H) allele was used for pragmatic reasons as it was readily available at the time the experiment was initiated. While we plan to examine the consequences of the mouse R270H allele in a future experiment, this is beyond the scope of the current study. We have added this as a study limitation in the Discussion as follows p14, lines 7-9: "Similarly, the Trp53 R172H allele used in this study was chosen for pragmatic reasons rather than its prevalence in high-risk endometrial cancer and it will be of interest to examine the effects of orthologues of more common mutations such as TP53 R273H."
Reviewer comment on the revised text: The revised sentence needs to be further modified to include that facts that the human R175H mutation (equivalent to the mouse R172H mutation) is a rare among TP53 mutations in the UCEC and UCS cohorts.

Referee #3 (Remarks for Author):

The authors have addressed my concerns.

***** Reviewer's comments *****

Referee #1 (Remarks for Author):

Specific comment-1:
The authors' response reads: "While most are low grade endometrioid tumours of early stage with favourable prognosis, the 10-20% of cases classified as high-risk have considerably worse outcomes2-4. This group includes high grade (grade 3) endometrioid tumours, cases of non-endometrioid histologies including serous, clear-cell and undifferentiated histology, and de-differentiated carcinosarcomas; metaplastic tumours derived from epithelial progenitors5."
Reviewer comment on the revised text: Please further clarify whether the "10-20% of cases classified as high-risk" refers to 10-20% of all EC cases, or to 10-20% of endometrioid cases.

> We have changed the text to read "…10-20% of ECs…" to clarify that the denominator is all endometrial cancers rather than endometrioid cancers only.

Specific comment-2:
Original comment: "The first paragraph of the results section should clarify that in the TCGA copy number high UCEC subgroup and in the TCGA carcinosarcomas, PTEN mutations and FBXW7 mutations rarely co-exist; in fact, in the TCGA copy number high UCEC subgroup they are mutually exclusive (q=0.036). This information can be retrieved using the cBioPortal"
The authors' response: "This is an important point which we are happy to add. For consistency, we have tested mutual exclusivity of oncogenic driver mutations among the 219 high risk tumours (copy no high UCEC and UCS) analysed elsewhere in our manuscript and now provide this in the first section of the Results, p6, lines 21-23: Oncogenic mutations in FBXW7 in this cohort of high risk endometrial cancers tended to mutual exclusivity with those in TP53 (OR=0.53, P=0.12) and particularly PTEN (OR=0.32, P=0.067). "
Reviewer comment on the revised text: Because the UCEC and UCS tumors are moelcualrly distinct, the data on mutual exclusivity should calculated and presented separately for each cohort. In addition to the p-values, the q-values must be provided to account to multiple testing of > 21,000 protein genes sequenced. Odds ratios, p-values and q-values can easily be retrieved from the cBIOportal for each of these cohorts.

> We would respectfully contest the statement that UCEC and UCS tumours should be analysed separately. Carcinosarcomas were reclassified as endometrial carcinomas rather than mixed epithelial and mesenchymal malignancies in the latest (2020) WHO classification, as sequencing studies have demonstrated the mesenchymal component represents transdifferentiation from carcinoma during their development. This is further evidenced by the observation that carcinosarcomas may be classified molecularly into any of the four molecular groups defined by TCGA and codifed in the latest WHO classification. Therefore our strong preference is to analyse UCEC and UCS together in accordance with most recent pathological consensus.

On the question of multiple testing, we agree that an agnostic search for mutual exclusivity across all >21,000 genes would indeed have to use q values to adjust for molecular testing. However, this is not what we are doing. Rather we are only testing exclusivity of FBXW7 mutations with mutations in *PTEN* and *TP53* (two tests). To clarify that P values are unadjusted we have explicitly noted this in the Results; as neither meet the threshold for statistical significance the reader can infer that this will remain the case after adjustment for two tests. (please note that we have highlighted the tendency to mutual exclusivity in the text to avoid an over-reliance on P values in interpretation).

Specific comment-3:
Original comment: The rationale for choosing the R172H as the conditional knockin mutant of murine Trp53 should be provided since the corresponding human mutation (TP53-R175H) is rare in the TCGA copy number high UCEC subgroup, (constituting only 7 of 146 (4.8%) TP53 mutations in this subgroup), and is also rare in human TCGA carcinosarcomas and in endometrioid ECs in TCGA. Three other TP53 residues are mutated more frequently in human EC (S241, R248, and R273) so the rationale for knocking in the murine mutation that corresponds to human TP53-T175H is not obvious.
The authors' response: This is another important point. The mouse R172H (human R175H) allele was used for pragmatic reasons as it was readily available at the time the experiment was initiated. While we plan to examine the consequences of the mouse R270H allele in a future experiment, this is beyond the scope of the current study. We have added this as a study limitation in the Discussion as follows p14, lines 7-9: "Similarly, the Trp53 R172H allele used in this study was chosen for pragmatic reasons rather than its prevalence in high-risk endometrial cancer and it will be of interest to examine the effects of orthologues of more common mutations such as TP53 R273H."
Reviewer comment on the revised text: The revised sentence needs to be further modified to include that facts that the human R175H mutation (equivalent to the mouse R172H mutation) is a rare among TP53 mutations in the UCEC and UCS cohorts.

> We are happy to change this to clarify and have added the exact frequency of mutations at both codons to the text:

Similarly, the *Trp53* R172H allele used in this study was chosen for pragmatic reasons rather than its prevalence in high-risk endometrial cancer (6% of oncogenic *TP53* point mutations in high risk EC in the TCGA studies) and it will be of interest to examine the effects of orthologues of more common mutations such as *TP53* R273H (14% of oncogenic *TP53* point mutations in TCGA) .

Referee #3 (Remarks for Author):

The authors have addressed my concerns.

> We thank the reviewer for his/her comments and are glad we have addressed them.

2nd Aug 2023

Dear Dr. Church,

Thank you for sending the revised files. I am pleased to inform you that your manuscript is accepted for publication and is now being sent to our publisher to be included in the next available issue of EMBO Molecular Medicine!

Please read below for additional IMPORTANT information regarding your article, its publication and the production process.

Congratulations on your interesting work!

With kind regards,

Lise Roth

Lise Roth, Ph.D
Senior Editor
EMBO Molecular Medicine

Follow us on Twitter @EmboMolMed
Sign up for eTOCs at embopress.org/alertsfeeds

*** *** *** IMPORTANT INFORMATION *** *** ***

SPEED OF PUBLICATION
The journal aims for rapid publication of papers, using using the advance online publication "Early View" to expedite the process: A properly copy-edited and formatted version will be published as "Early View" after the proofs have been corrected. Please help the Editors and publisher avoid delays by providing e-mail address(es), telephone and fax numbers at which author(s) can be contacted.

Should you be planning a Press Release on your article, please get in contact with embomolmed@wiley.com as early as possible, in order to coordinate publication and release dates.

LICENSE AND PAYMENT:

All articles published in EMBO Molecular Medicine are fully open access: immediately and freely available to read, download and share.

EMBO Molecular Medicine charges an article processing charge (APC) to cover the publication costs. You, as the corresponding author for this manuscript, should have already received a quote with the article processing fee separately. Please let us know in case this quote has not been received.

Once your article is at Wiley for editorial production you will receive an email from Wiley's Author Services system, which will ask you to log in and will present you with the publication license form for completion. Within the same system the publication fee can be paid by credit card, an invoice, pro forma invoice or purchase order can be requested.

Payment of the publication charge and the signed Open Access Agreement form must be received before the article can be published online.

PROOFS

You will receive the proofs by e-mail approximately 2 weeks after all relevant files have been sent o our Production Office. Please return them within 48 hours and if there should be any problems, please contact the production office at embopressproduction@wiley.com.

Please inform us if there is likely to be any difficulty in reaching you at the above address at that time. Failure to meet our deadlines may result in a delay of publication.

All further communications concerning your paper proofs should quote reference number EMM-2022-17094-V3 and be directed to the production office at embopressproduction@wiley.com.

EMBO Press Author Checklist

Corresponding Author Name: David Church	
Journal Submitted to: EMBO molecular medicine	
Manuscript Number: EMM-2022-17094	

USEFUL LINKS FOR COMPLETING THIS FORM
The EMBO Journal - Author Guidelines
EMBO Reports - Author Guidelines
Molecular Systems Biology - Author Guidelines
EMBO Molecular Medicine - Author Guidelines

Reporting Checklist for Life Science Articles (updated January
This checklist is adapted from Materials Design Analysis Reporting (MDAR) Checklist for Authors. MDAR establishes a minimum set of requirements in transparent reporting in the life sciences (see Statement of Task: 10.31222/osf.io/9sm4x). Please follow the journal's guidelines in preparing your
Please note that a copy of this checklist will be published alongside your article.

Abridged guidelines for figures
1. Data
The data shown in figures should satisfy the following conditions:
- the data were obtained and processed according to the field's best practice and are presented to reflect the results of the experiments in an accurate and unbiased manner.
- ideally, figure panels should include only measurements that are directly comparable to each other and obtained with the same assay.
- plots include clearly labeled error bars for independent experiments and sample sizes. Unless justified, error bars should not be shown for technical
- if n<5, the individual data points from each experiment should be plotted. Any statistical test employed should be justified.
- Source Data should be included to report the data underlying figures according to the guidelines set out in the authorship guidelines on Data

2. Captions
Each figure caption should contain the following information, for each panel where they are relevant:
- a specification of the experimental system investigated (eg cell line, species name).
- the assay(s) and method(s) used to carry out the reported observations and measurements.
- an explicit mention of the biological and chemical entity(ies) that are being measured.
- an explicit mention of the biological and chemical entity(ies) that are altered/varied/perturbed in a controlled manner.
- the exact sample size (n) for each experimental group/condition, given as a number, not a range;
- a description of the sample collection allowing the reader to understand whether the samples represent technical or biological replicates (including how many animals, litters, cultures, etc.).
- a statement of how many times the experiment shown was independently replicated in the laboratory.
- definitions of statistical methods and measures:
  - common tests, such as t-test (please specify whether paired vs. unpaired), simple χ2 tests, Wilcoxon and Mann-Whitney tests, can be unambiguously identified by name only, but more complex techniques should be described in the methods section;
  - are tests one-sided or two-sided?
  - are there adjustments for multiple comparisons?
  - exact statistical test results, e.g., P values = x but not P values < x;
  - definition of 'center values' as median or average;
  - definition of error bars as s.d. or s.e.m.

Please complete ALL of the questions below.
Select "Not Applicable" only when the requested information is not relevant for your study.

Materials

Newly Created Materials	Information included in the manuscript?	In which section is the information available? (Reagents and Tools Table, Materials and Methods, Figures, Data Availability Section)
New materials and reagents need to be available; do any restrictions apply?	Not Applicable	

Antibodies	Information included in the manuscript?	In which section is the information available? (Reagents and Tools Table, Materials and Methods, Figures, Data Availability Section)
For **antibodies** provide the following information:<br>- Commercial antibodies: RRID (if possible) or supplier name, catalogue number and or/clone number<br>- Non-commercial: RRID or citation	Yes	Table S11

DNA and RNA sequences	Information included in the manuscript?	In which section is the information available? (Reagents and Tools Table, Materials and Methods, Figures, Data Availability Section)
**Short novel DNA or RNA including primers, probes**: provide the sequences.	Yes	Table S10

Cell materials	Information included in the manuscript?	In which section is the information available? (Reagents and Tools Table, Materials and Methods, Figures, Data Availability Section)
**Cell lines:** Provide species information, strain. Provide accession number in repository **OR** supplier name, catalog number, clone number, and/**OR** RRID.	Yes	Manuscript Methods
**Primary cultures:** Provide species, strain, sex of origin, genetic modification status.	Not Applicable	
Report if the cell lines were recently **authenticated** (e.g., by STR profiling) and tested for mycoplasma contamination.	Yes	Manuscript Methods

Experimental animals	Information included in the manuscript?	In which section is the information available? (Reagents and Tools Table, Materials and Methods, Figures, Data Availability Section)
**Laboratory animals or Model organisms:** Provide species, strain, sex, age, genetic modification status. Provide accession number in repository **OR** supplier name, catalog number, clone number, **OR** RRID.	Yes	Manuscript Methods, Mice
**Animal observed in or captured from the field:** Provide species, sex, and age where possible.	Not Applicable	
Please detail **housing and husbandry conditions**.	Yes	Manuscript Methods, Mice

Plants and microbes	Information included in the manuscript?	In which section is the information available? (Reagents and Tools Table, Materials and Methods, Figures, Data Availability Section)
**Plants:** provide species and strain, ecotype and cultivar where relevant, unique accession number if available, and source (including location for collected wild specimens).	Not Applicable	
**Microbes:** provide species and strain, unique accession number if available, and source.	Not Applicable	

Human research participants	Information included in the manuscript?	In which section is the information available? (Reagents and Tools Table, Materials and Methods, Figures, Data Availability Section)
If collected and within the bounds of privacy constraints report on age, sex and gender or ethnicity for all study participants.	Not Applicable	

Core facilities	Information included in the manuscript?	In which section is the information available? (Reagents and Tools Table, Materials and Methods, Figures, Data Availability Section)
If your work benefited from core facilities, was their service mentioned in the acknowledgments section?	Yes	Acknowledgements

Design

Study protocol	Information included in the manuscript?	In which section is the information available? (Reagents and Tools Table, Materials and Methods, Figures, Data Availability Section)
If study protocol has been **pre-registered, provide DOI in the manuscript**. For clinical trials, provide the trial registration number **OR** cite DOI.	Not Applicable	
Report the **clinical trial registration number** (at ClinicalTrials.gov or equivalent), where applicable.	Not Applicable	

Laboratory protocol	Information included in the manuscript?	In which section is the information available? (Reagents and Tools Table, Materials and Methods, Figures, Data Availability Section)
Provide DOI OR other citation details if **external detailed step-by-step protocols** are available.	Not Applicable	

Experimental study design and statistics	Information included in the manuscript?	In which section is the information available? (Reagents and Tools Table, Materials and Methods, Figures, Data Availability Section)
Include a statement about **sample size** estimate even if no statistical methods were used.	Yes	Manuscript Methods
Were any steps taken to minimize the effects of subjective bias when allocating animals/samples to treatment (e.g. **randomization procedure**)? If yes, have they been described?	Yes	Materials and Methods
Include a statement about **blinding** even if no blinding was done.	Yes	Manuscript Methods and main text
Describe **inclusion/exclusion criteria** if samples or animals were excluded from the analysis. Were the criteria pre-established? If sample or data points were omitted from analysis, report if this was due to attrition or intentional exclusion and provide justification.	Yes	Materials and Methods
For every figure, are **statistical tests** justified as appropriate? Do the data meet the assumptions of the tests (e.g., normal distribution)? Describe any methods used to assess it. Is there an estimate of variation within each group of data? Is the variance similar between the groups that are being statistically compared?	Yes	Detailed in figures - non parametric tests used given non-linearity of data

Sample definition and in-laboratory replication	Information included in the manuscript?	In which section is the information available? (Reagents and Tools Table, Materials and Methods, Figures, Data Availability Section)
In the figure legends: state number of times the experiment was **replicated** in laboratory.	Yes	Figure legends
In the figure legends: define whether data describe **technical or biological replicates**.	Yes	Figure legends

Ethics

Ethics	Information included in the manuscript?	In which section is the information available? (Reagents and Tools Table, Materials and Methods, Figures, Data Availability Section)
Studies involving **human participants**: State details of **authority granting ethics approval** (IRB or equivalent committee(s), provide reference number for approval.	Yes	Manuscript Methods
Studies involving **human participants**: Include a statement confirming that **informed consent** was obtained from all subjects and that the experiments conformed to the principles set out in the WMA Declaration of Helsinki and the Department of Health and Human Services Belmont Report.	Yes	Manuscript Methods
Studies involving **human participants: For** publication of **patient photos**, include a statement confirming that consent to publish was obtained.	Not Applicable	
Studies involving experimental **animals**: State details of **authority granting ethics approval** (IRB or equivalent committee(s), provide reference number for approval. Include a statement of compliance with ethical regulations.	Yes	Manuscript Methods
Studies involving **specimen and field samples:** State if relevant **permits** obtained, provide details of authority approving study; if none were required, explain why.	Not Applicable	

Dual Use Research of Concern (DURC)	Information included in the manuscript?	In which section is the information available? (Reagents and Tools Table, Materials and Methods, Figures, Data Availability Section)
Could your study fall under dual use research restrictions? Please check biosecurity documents and list of **select agents and toxins** (CDC): https://www.selectagents.gov/sat/list.htm	Not Applicable	
If you used a select agent, is the security level of the lab appropriate and reported in the manuscript?	Not Applicable	
If a study is subject to dual use research of concern regulations, is the name of the **authority granting approval and reference number** for the regulatory approval provided in the manuscript?	Not Applicable	

Reporting

The MDAR framework recommends adoption of discipline-specific guidelines, established and endorsed through community initiatives. Journals have their own policy about requiring specific guidelines and recommendations to complement MDAR.

Adherence to community standards	Information included in the manuscript?	In which section is the information available? (Reagents and Tools Table, Materials and Methods, Figures, Data Availability Section)
State if relevant guidelines or checklists (e.g., **ICMJE, MIBBI, ARRIVE, PRISMA**) have been followed or provided.	Not Applicable	
For **tumor marker prognostic studies,** we recommend that you follow the **REMARK** reporting guidelines (see link list at top right). See author guidelines, under 'Reporting Guidelines'. Please confirm you have followed these guidelines.	Not Applicable	
For **phase II and III randomized controlled trials**, please refer to the **CONSORT** flow diagram (see link list at top right) and submit the CONSORT checklist (see link list at top right) with your submission. See author guidelines, under 'Reporting Guidelines'. Please confirm you have submitted this list.	Not Applicable	

Data Availability

Data availability	Information included in the manuscript?	In which section is the information available? (Reagents and Tools Table, Materials and Methods, Figures, Data Availability Section)
Have **primary datasets** been deposited according to the journal's guidelines (see 'Data Deposition' section) and the respective accession numbers provided in the Data Availability Section?	Yes	Data availability
Were **human clinical and genomic datasets** deposited in a public access-controlled repository in accordance to ethical obligations to the patients and to the applicable consent agreement?	Not Applicable	
Are **computational models** that are central and integral to a study available without restrictions in a machine-readable form? Were the relevant accession numbers or links provided?	Not Applicable	
If publicly available data were reused, provide the respective **data citations in the reference list**.	Yes	References
